# Synthesis of Thiophenes from Pyridines Using Elemental Sulfur

**DOI:** 10.1002/anie.202512321

**Published:** 2025-07-25

**Authors:** Zi Liu, Michael F. Greaney

**Affiliations:** ^1^ School of Chemistry The University of Manchester Oxford Road Manchester M13 9PL UK

**Keywords:** Pyridine, Sulfur, Thiophene, skeletal edit

## Abstract

We describe a skeletal editing of pyridines to afford thiophenes through a formal [4 + 1] reaction using elemental sulfur. 2‐Arylpyridines are converted to ring‐opened aza‐triene Zincke ketone structures, followed by simple treatment with sulfur to give 2‐aroylthiophene products directly. The amphiphilic character of octasulfur enables smooth reaction with the Zincke dienamine, affording the cyclized products under mild and neutral conditions. We illustrate this new disconnection with a synthesis of the anti‐inflammatory drug suprofen from a pyridine starting material.

## Graphical abstract

Thiophenes can be disconnected to pyridines through a novel formal [4 + 1] treatment of Zincke ketones with elemental sulfur. In situ ring‐opening of the π‐deficient heteroarene gives an extended diene, which undergoes simple reaction with octasulfur to give the π‐excessive thiophene.

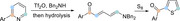

Methods for interconverting heteroarene rings have long been recognized as powerful transformations, enabling the exchange of structures having very different chemical reactivity (Scheme 1a).^[^ ^1^ ^]^ Recent advances in skeletal‐editing^[^ ^2^ ^]^ have dramatically enhanced the efficiency and usability of these transmutations, enabling far wider scope of application.^[^ ^3^, ^4^, ^5^, ^6^, ^7^, ^8^, ^9^, ^10^, ^11^, ^12^, ^13^ ^]^ These thought‐provoking disconnections show how heteroarene ring systems, often having orthogonal chemistries (e.g., π‐deficient / π‐excessive) can be interconverted in nonobvious ways. The resultant scaffold‐hopping transformations have widespread applications in synthesis, e.g., in medicinal chemistry, where late‐stage edits can effect deep‐seated structural and / or electronic change to a drug candidate series.^[^ ^14^ ^]^ In complex molecule synthesis, multistep routes can exploit the favorable properties of one heteroarene (e.g., stability, ease of derivatization), with a subsequent scaffold switch affording the target heterocycle structure.^[^ ^15^, ^16^ ^]^

**Scheme 1:**
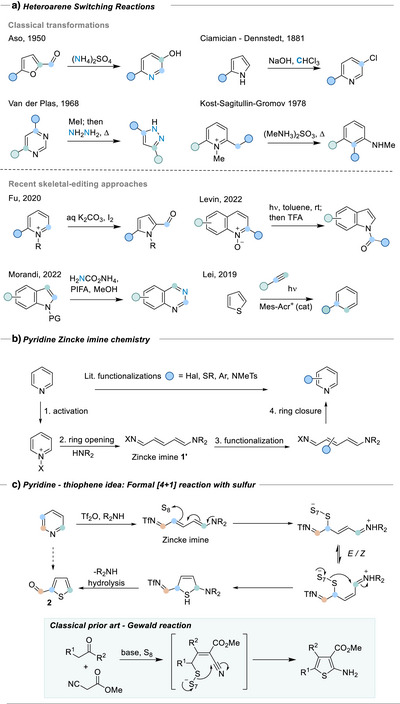
a) Heteroarene‐switching reactions.^[^ ^1^, ^2^, ^3^, ^12^ ^]^ b) Pyridine functionalization via ring‐opening to Zincke intermediates. c) Proposed pyridine to thiophene skeletal edit.

Pyridines have featured prominently in recent skeletal‐editing studies,^[^ ^6^, ^10^, ^11^, ^13^, ^17^, ^18^, ^19^, ^20^, ^21^, ^22^, ^23^, ^24^, ^25^, ^26^, ^27^, ^28^ ^]^ being key structures in drug design and biologically active natural products. In particular, the addition of nucleophile ring‐opening ring‐closing mechanisms (ANRORC) distinct to pyridines create possibilities for new functionalizations and conversions into different ring structures.^[^ ^29^ ^]^ Scheme 1b shows a Zincke reaction, the archetypal ANRORC process, whereby an activated pyridine undergoes ring opening with an amine to give an extended Zincke imine **1′**.^[^ ^30^ ^]^ A number of functionalizations then become possible, which are very difficult to achieve directly on the native pyridine. The venerable Zincke reaction has undergone a revival in the recent literature,^[^ ^31^ ^]^ both as a vehicle for pyridine functionalization (via ring‐closing back to the pyridine),^[^ ^32^, ^33^, ^34^, ^35^ ^]^ and as a skeletal editing strategy to convert pyridines to other arenes and heteroaromatics.^[^ ^17^, ^18^, ^19^, ^20^, ^23^, ^25^, ^28^ ^]^

Our recent work on pyridine switching to benzene rings via ANRORC led us to consider alternative pathways to different heterocycles.^[^ ^20^ ^]^ The transmutation of pyridines into 5‐membered π‐excessive heteroarenes represents a powerful skeletal edit that links two important, but very different, classes of heterocycle. The pyridine–pyrrole conversion is precedented in a number of classical photochemical rearrangements,^[^ ^36^, ^37^ ^]^ whereby the N atom is retained from the starting material.

Recently, Fu and co‐workers described a polar Zincke method for this transformation on alkyl‐pyridiniums, using hydroxide as a nucleophile in the presence of iodine.^[^ ^38^, ^39^ ^]^ Conversion of pyridines into furans and thiophenes, on the other hand, has not been described in the literature.

We were interested in exploring this skeletal edit in the sulfur series, with the idea of using elemental sulfur as an amphiphilic reagent to access thiophenes. Thiophenes are privileged drug motifs present in many valuable medicines, in addition to being widely explored in organic materials for their opto‐electronic properties.^[^ ^40^ ^]^ Sulfur, existing as cyclic octasulfur monomers, has versatile reactivity in organic chemistry, whereby it can undergo ring opening with a carbon nucleophile, followed by extrusion of S_7_ (or fragments thereof) to create a nucleophilic thiol group on the organic substrate.^[^ ^41^ ^]^ This thiol can then react further in ring‐closing events. A well‐known exemplification of this process is the classical Gewald reaction, for synthesizing aminothiophenes from ketones and cyanoacetates.^[^ ^42^ ^]^ We wondered if Zincke imines could undergo a similar transformation, via their inherent enamine reactivity, to give the 2‐carboxythiophene products **2** (Scheme 1c) and create a thiophene‐pyridine disconnection. Zincke imines have a richly explored [4 + 2] chemistry to make 6‐membered rings,^[^ ^43^ ^]^ but formal [4 + 1] processes have not been described to the best of our knowledge.

Our initial trials of reacting sulfur with the Zincke imine **1′a** derived from 2‐phenylpyridine, dibenzylamine, and triflic anhydride (Tf_2_O), were disappointing, with little reactivity being observed under a variety of conditions. Reasoning that the neutral triflyl Zincke imine **1′a** might be insufficiently nucleophilic to react with sulfur, we turned to the Zincke ketone **1a**, available through simple hydrolysis, and were pleased to observe successful reaction, albeit in low yield. Thiophene **2a** was formed in 15% yield by treating **1a** with sulfur in acetonitrile (MeCN) at 60 °C overnight. Encouraged by this promising result, further evaluation of various solvents demonstrated that solvent choice plays a crucial role. The reaction was completely suppressed when toluene (Tol), ethyl acetate (EA), or tetrahydrofuran (THF) was used as the reaction solvent (Table 1, entries 2–4).

**Table 1:** Reaction optimization. Conditions: Zincke ketone **1a** (0.2 mmol), sulfur (x equiv.), solvent (2 mL), T °C, overnight, under air.

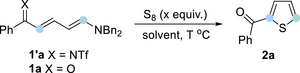				
Entry	Equiv of S_8_	T (°C)	Solvent	Yield (%)^a)^
1	3.0	60	MeCN	15
2	3.0	60	Tol	0
3	3.0	60	EA	0
4	3.0	60	THF	0
5	3.0	60	DMF	49
6	3.0	40	DMF	5
7	3.0	80	DMF	55
8	1.2	60	DMF	36
9	5.0	60	DMF	43
10^b)^	3.0	60	DMF	59

^a)^ Isolated yield.

^b)^ Under N_2_.

In contrast, conducting the reaction in N,N‐dimethylformamide (DMF) notably improved the yield of thiophene product **2a** to 49% (Table 1, entry 5). We subsequently modified other reaction parameters, including reaction temperature and sulfur loading, but these modifications did not result in any significant enhancement in reaction efficiency (Table 1, entries 6–9). Further control experiments revealed that performing the reaction under a nitrogen atmosphere boosted the yield of **2a** to 59% (Table 1, entry 10). Upon establishing these optimal reaction conditions, we set out to explore the substrate scope and limitations of this formal [4 + 1] annulation by examining a range of Zincke ketones (Scheme 2). Generally, a broad range of 2‐phenyl‐substituted pyridines, either with electron‐donating or electron‐withdrawing groups were well‐tolerated, furnishing the corresponding thiophene derivatives (**2b‐2j**) in moderate to good yields.

**Scheme 2:**
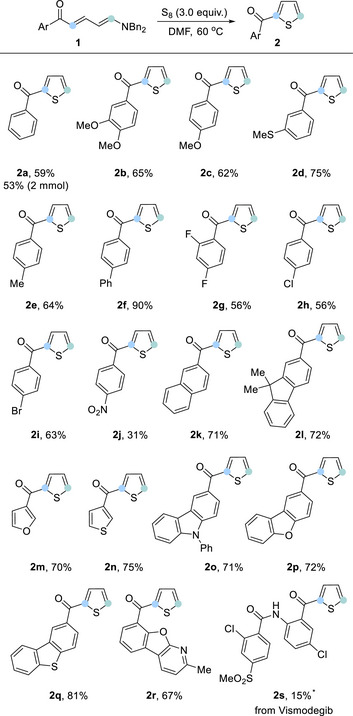
Substrate scope. Standard conditions: Zincke ketone (0.2 mmol), sulfur (3.0 equiv), DMF (0.1 M), 60 °C, overnight, under N_2_. ^*^ Dimethylsulfoxide (DMSO) as solvent.

Electron‐rich Zincke ketones (**1b**‐**1e**) were converted into 2‐carboxythiophenes more efficiently than their electron‐deficient counterparts (**1g**‐**1j**). Also, the substitution patterns on the aryl ring, including *para*‐, *meta*‐, and *ortho*‐ substitution, did not interfere with reaction efficiency. Halogen‐substituted substrates, **1h** and **1i**, were compatible, providing valuable handles for further derivatization. In particular, the introduction of a large conjugated system on 2‐arylpyridine moiety significantly raised the reaction yields (**2f**, **2k**, **2l**). 2‐Heteroaryl substituted pyridines were also studied to prepare the corresponding Zincke ketone, engaging in this annulation and offering the desired thiophene compounds, including the unsymmetrically linked bis‐thiophene **2n**, in 70–81% yields (**2m**‐**2r**). The approach could be further extended to the skeletal editing of a drug molecule, Vismodegib, thereby offering a late‐stage pyridine–thiophene switch for scaffold investigation. In terms of limitations, the reaction did not proceed with 4‐substituted pyridines, with difficulties encountered in preparing pure Zincke ketones and aldehydes.

We could further expand the scope of thiophene formation through additional functionalization of the Zincke ketone, using a Pd‐catalyzed arylation we have recently reported.^[^ ^32^ ^]^ Treatment of **1a** with an aryl iodide under Heck conditions, followed by formal [4 + 1] annulation with sulfur, gave the 2,3‐disubstituted thiophenes (**2t**–**2w**) (Scheme 3). The 2,3‐substitution pattern supports the proposed sequence of bond formations set out in Scheme 1c for thiophene formation (see Supporting Information for an alternative mechanism).

**Scheme 3:**
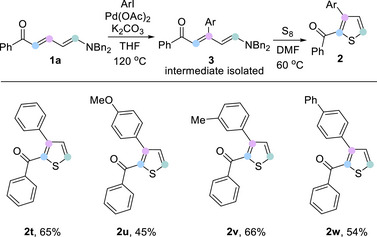
Substrate scope of disubstituted Zincke ketone. Standard conditions: Zincke ketone (0.2 mmol), sulfur (3.0 equiv), DMF (0.1 M), 60 °C, overnight, under N_2_.

To display the practicality of the developed procedure, we carried out a scale‐up reaction (2.0 mmol) by taking Zincke ketone **1a** derived from 2‐phenylpyridine as a model substrate, affording the 2‐carboxythiophene product **2a** in 53% yield. Furthermore, a series of post‐synthetic functionalizations of 2‐acylthiophene product were performed to highlight the synthetic utility of this methodology (Scheme 4a). Treatment of **2a** with Lawesson's reagent resulted in quantitative conversion of the ketone into the corresponding thione **4a**. Selective reduction of the carbonyl group of **2a** using NaBH_4_ and AlCl_3_ yielded the methylene derivative **4b**. Meanwhile, the ketone **2a** underwent a Wittig reaction to provide the terminal alkene **4c** in 86% yield. Finally, a Pd‐catalyzed decarboxylative arylation^[^ ^44^ ^]^ of **2a** with 2,4‐dimethoxybenzoic acid in DMSO / DME (DME = 1,2‐dimethoxylethane) led to 2,5‐disubstituted thiophene **4d** in 65% yield. To further streamline the process, we developed a telescoped one‐pot sequence for the synthesis of compound **2** (Scheme 4b) from starting pyridines. Zincke imine formation and hydrolysis was carried out in ethyl acetate, followed by concentration and a solvent switch to DMF for the sulfur treatment. The one‐pot approach was demonstrated for four different 2‐arylpyridines, affording the thiophenes **2a**, **2k**, **2l**, and **2n** directly in good overall yields for the three‐step process.

**Scheme 4:**
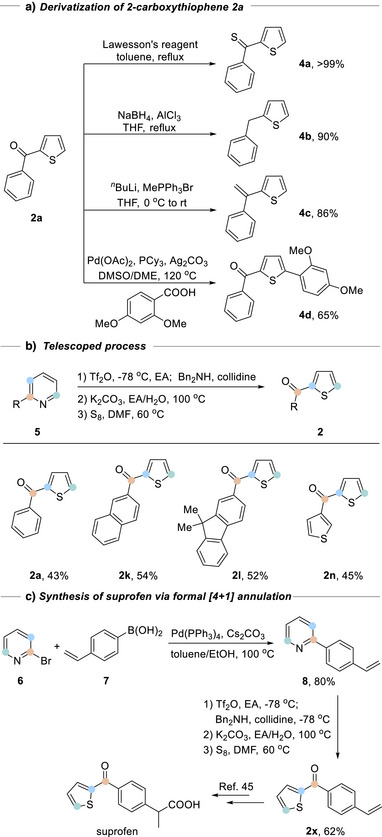
Synthetic application.

We could apply this pyridine–thiophene transformation to the formal synthesis of suprofen, an anti‐inflammatory drug. As shown in Scheme 4c, 2‐bromopyridine could be arylated via Suzuki cross‐coupling to supply **8**, which is then transformed in a one‐pot reaction to furnish **2x**, an important precursor in the synthesis of suprofen,^[^ ^45^ ^]^ in 62% overall yield.

In summary, we have developed a novel skeletal‐editing method for the synthesis of thiophene derivatives via formal [4 + 1] annulation of Zincke ketones with elemental sulfur. The methods display broad substrate scope, tolerating a wide range of functional groups and substitution patterns, and have been applied in the modification and synthesis of drug structures.

## Conflict of Interests

The authors declare no conflict of interest.

## Supporting information



Supporting Information

## Data Availability

The data that support the findings of this study are available from the corresponding author upon reasonable request.
