# Supplementary material for: Synthesis of Thiophenes from Pyridines Using Elemental Sulfur
Source: Angew Chem Int Ed Engl. 2025 Jul 25;64(37):e202512321. doi: 10.1002/anie.202512321 (PMC12416476; doi:10.1002/anie.202512321)
Supplement: Supplementary file 1 — Supporting Information [file ANIE-64-e202512321-s001.pdf]

# Synthesis of Thiophenes from Pyridines Using Elemental Sulfur

## Supporting Information

Zi Liu and Michael F. Greaney*

Department of Chemistry, University of Manchester, Oxford Rd, Manchester M13 9PL, United Kingdom

Table of Contents

1. General methods S2
2. General procedures S2
3. Compound characterization S3
4. Mechanism S24
5. References S24
6. NMR spectra S26

## 1. General methods

Nuclear magnetic resonance spectra were acquired on 400 MHz (Bruker AVIII HD 400, Bruker AVIII 400). All $^{1}H$ NMR spectra are reported in parts per million (ppm) and were measured relative to the signals at 7.26 ppm ($CHCl_3$). All $^{13}C$ NMR spectra were reported in ppm relative to residual $CHCl_3$ (77.16 ppm) and were obtained with 1H-decoupling. All $^{19}F$ chemical shifts were unadjusted from raw data. Data for $^{1}H$ NMR are described as following: chemical shift (δ in ppm), multiplicity (s, singlet; d, doublet; t, triplet; q, quartet; quin, quintet; m, multiplet; br, broad signal), coupling constant (Hz), integration.

High resolution mass spectra were obtained on ThermoFisher Scientific Q-ExactiveTM, Thermo Scientific Exactive plus EMR and Agilent 6530 Q-TOF instruments, using either electrospray ionisation (ESI), atmospheric-pressure chemical ionisation (APCI) as ionisation methods in the positive and negative mode.

Flash column chromatography was carried out by using re-used 10g, 25g or 50g Biotage® Snap Ultra or Biotage Sfär Silica cartridges on a Biotage Isolera Four automated column, using 35-70 µm, 60 Å silica gel for chromatography from ThermoFisher Scientific® or 40-63 µm 60 Å silica gel from Sigma-Aldrich.

Melting points (mp) were recorded on a Griffin melting point apparatus to the nearest degree and are uncorrected.

All air and/or moisture sensitive reactions were performed under an atmosphere of dry nitrogen using anhydrous solvents. All solvents for air sensitive reactions were degassed by bubbling $N_2$ on the Schlenk line. All commercially available reagents and solvents were used as received without further purification. 5-(Dibenzylamino)-3-(4-methoxyphenyl)-1-phenylpenta-2,4-dien-1-one was prepared according to a known procedure.[1]

## 2. General procedures

**General procedure A:**

To an oven-dried microwave vial charged with a stir bar were added boronic acid (1.2 equiv.), $Pd(PPh_3)_4$ (2 mol%) and $K_2CO_3$ (2.0 equiv.) and the vial was sealed. The air inside was replaced with $N_2$ by three vacuum/$N_2$ cycles. Then toluene/$H_2O$ (10/1, 0.2 M), 2-bromopyridine (1.0 equiv.) were added into the vial and the mixture was stirred at 100 °C overnight. After cooling to room temperature, the mixture was diluted with EtOAc, washed with brine, extracted with EtOAc (x3). The combined organic phases were dried over $Na_2SO_4$ and concentrated *in vacuo*. The residue was purified by flash column chromatography to give the corresponding 2-substituted pyridine compounds.

**General procedure B:**

An oven-dried microwave vial was charged with pyridine (if it is solid) (1.0 mmol, 1.0 equiv.), sealed and placed on the Schlenk line to vacuum and refill with nitrogen three times. Then pyridine (if it is liquid) (1.0 mmol, 1.0 equiv.), EtOAc (0.1 M) were added under a flow of nitrogen. The reaction solution was cooled to -78 °C and $Tf_2O$ (1.0 equiv.) was added dropwise. After the addition was completed, the reaction was kept at -78 °C for 30 min and then a solution of dibenzylamine (1.2 equiv.) in EtOAc (1.0 M) was added slowly followed by collidine (1.0 equiv.). The reaction mixture was stirred at -78 °C for an additional 30 min and then allowed to warm to room temperature and stirred for 30 min. The reaction was washed with water three times and the organic phase was concentrated. The residue was dissolved in EtOAc/$H_2O$ (10/1, 0.1 M) and $K_2CO_3$ (2.0 equiv.) was added into the vial. The mixture was stirred at 100 °C overnight, then cooled to room temperature, washed with brine and extracted with EtOAc (x3). The combine organic layers were dried over $Na_2SO_4$ and concentrated *in vacuo* and the product was purified by flash column chromatography on silica gel.

**General procedure C:**

A mixture of Zincke ketone (0.2 mmol, 1.0 equiv.) and $S_8$ (0.6 mmol, 19.2 mg, 3.0 equiv.) were added into an oven-dried microwave vial. Dry DMF (2 mL) was added under nitrogen and the reaction mixture was stirred at 60 °C overnight. After cooling to room temperature, the reaction was diluted with EtOAc and washed with water (x3). The organic phase was dried over $Na_2SO_4$, filtered and concentrated *in vacuo*. The product was isolated by using flash column chromatography.

## 3. Compound characterization

**2-(3,4-dimethoxyphenyl)pyridine**

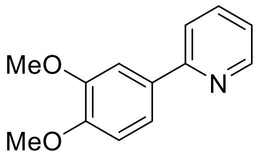


Prepared according to general procedure **A** (5.0 mmol scale) by using (3,4-dimethoxyphenyl)boronic acid to yield the titled compound as white solid (920 mg, 86%).

**$^1$H NMR** (400 MHz, $CDCl_3$) δ 8.66 – 8.65 (m, 1H), 7.74 – 7.66 (m, 3H), 7.51 (dd, *J* = 8.3, 2.1 Hz, 1H), 7.18 (ddd, *J* = 6.7, 4.8, 1.8 Hz, 1H), 6.95 (d, *J* = 8.3 Hz, 1H), 4.00 (s, 3H), 3.94 (s, 3H).

**$^{13}$C NMR** (100 MHz, $CDCl_3$) δ 157.2, 150.1, 149.6, 149.4, 136.8, 132.5, 121.7, 120.1, 119.5, 111.2, 110.1, 56.1.

The data are consistent with those already published.[2]

**2-(3-(methylthio)phenyl)pyridine**

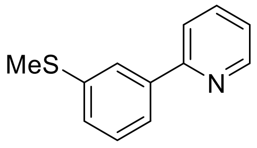


Prepared according to general procedure **A** (2.0 mmol scale) by using (3-(methylthio)phenyl)boronic acid to yield the titled compound as colourless oil (354 mg, 86%).

**$^1$H NMR** (400 MHz, $CDCl_3$) δ 8.70 (ddd, *J* = 4.8, 1.8, 1.0 Hz, 1H), 7.92 (t, *J* = 1.8 Hz, 1H), 7.80 – 7.68 (m, 3H), 7.39 (t, *J* = 7.8 Hz, 1H), 7.31 (ddd, *J* = 7.9, 2.0, 1.1 Hz, 1H), 7.28 – 7.22 (m, 1H), 2.56 (s, 3H).

**$^{13}$C NMR** (100 MHz, $CDCl_3$) δ 157.0, 149.7, 140.0, 139.4, 137.1, 129.3, 127.2, 125.1, 123.8, 122.5, 120.9, 16.0.

The data are consistent with those already published.[3]

**2-(4-methoxyphenyl)pyridine**

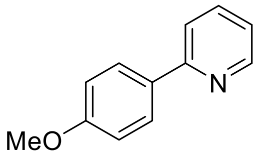


Prepared according to general procedure **A** (5.0 mmol scale) by using (4-methoxyphenyl)boronic acid to yield the titled compound as white solid (735 mg, 79%).

**$^1$H NMR** (400 MHz, $CDCl_3$) δ 8.65 (ddd, *J* = 4.8, 1.8, 1.0 Hz, 1H), 7.97 – 7.93 (m, 2H), 7.73 – 7.65 (m, 2H), 7.17 (ddd, *J* = 7.1, 4.8, 1.5 Hz, 1H), 7.02 – 6.98 (m, 2H), 3.86 (s, 3H).

**$^{13}$C NMR** (100 MHz, $CDCl_3$) δ 160.6, 157.2, 149.7, 136.8, 132.2, 128.3, 121.5, 120.0, 114.2, 55.5.

The data are consistent with those already published.[2]

**2-([1,1'-biphenyl]-4-yl)pyridine**

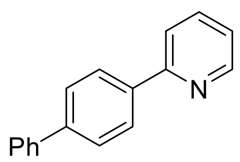

Prepared according to general procedure **A** (5.0 mmol scale) by using [1,1'-biphenyl]-4-ylboronic acid to yield the titled compound as white solid (1.0 g, 90%).

**$^1$H NMR** (400 MHz, $CDCl_3$) δ 8.74 – 8.72 (m, 1H), 8.11 – 8.08 (m, 2H), 7.80 – 7.72 (m, 4H), 7.69 – 7.66 (m, 2H), 7.50 – 7.46 (m, 2H), 7.40 – 7.36 (m, 1H), 7.24 (ddd, *J* = 6.8, 5.0, 2.3 Hz, 1H).

**$^{13}$C NMR** (100 MHz, $CDCl_3$) δ 157.1, 149.8, 141.8, 140.7, 138.4, 136.9, 128.9, 127.6, 127.6, 127.4, 127.2, 122.2, 120.6.

The data are consistent with those already published.[2]

**2-(4-chlorophenyl)pyridine**

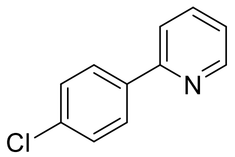

Prepared according to general procedure **A** (5.0 mmol scale) by using (4-chlorophenyl)boronic acid to yield the titled compound as white solid (451 mg, 48%).

**$^1$H NMR** (400 MHz, $CDCl_3$) δ 8.68 (ddd, *J* = 4.8, 1.8, 1.0 Hz, 1H), 7.95 – 7.92 (m, 2H), 7.77 – 7.73 (m, 1H), 7.69 (dt, *J* = 8.0, 1.2 Hz, 1H), 7.46 – 7.42 (m, 2H), 7.24 (ddd, *J* = 7.3, 4.8, 1.3 Hz, 1H).

**$^{13}$C NMR** (100 MHz, $CDCl_3$) δ 156.3, 149.9, 137.9, 137.0, 135.2, 129.0, 128.3, 122.5, 120.5.

The data are consistent with those already published.[2]

**2-(4-nitrophenyl) pyridine**

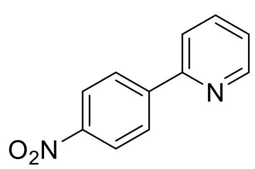

Prepared according to general procedure **A** (5.0 mmol scale) by using (4-nitrophenyl)boronic acid to yield the titled compound as white solid (716 mg, 72%).

**$^1$H NMR** (400 MHz, $CDCl_3$) δ 8.74 (d, *J* = 4.9 Hz, 1H), 8.31 (d, *J* = 8.8 Hz, 2H), 8.17 (d, *J* = 8.9 Hz, 2H), 7.85 – 7.76 (m, 2H), 7.34 (ddd, *J* = 6.7, 4.8, 2.2 Hz, 1H).

**$^{13}$C NMR** (100 MHz, $CDCl_3$) δ 155.0, 150.3, 148.3, 145.4, 137.3, 127.8, 124.1, 123.7, 121.3.

The data are consistent with those already published.[4]

**2-(naphthalen-2-yl)pyridine**

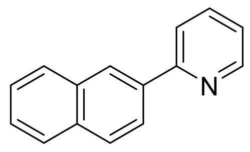

Prepared according to general procedure **A** (5.0 mmol scale) by using naphthalen-2-ylboronic acid to yield the titled compound as white solid (959 mg, 94%).

**$^1$H NMR** (400 MHz, $CDCl_3$) δ 8.76 (d, *J* = 4.9 Hz, 1H), 8.49 (s, 1H), 8.15 (dd, *J* = 8.5, 1.8 Hz, 1H), 7.9f8 – 7.94 (m, 2H), 7.90 – 7.86 (m, 2H), 7.80 (td, *J* = 7.7, 1.9 Hz, 1H), 7.51 (dd, *J* = 6.3, 3.3 Hz, 2H), 7.28 – 7.25 (m, 1H).

**$^{13}$C NMR** (100 MHz, $CDCl_3$) δ 157.5, 150.0, 137.0, 136.8, 133.8, 133.7, 128.9, 128.6, 127.8, 126.7, 126.5, 126.4, 124.7, 122.3, 121.0.

The data are consistent with those already published.[2]

**2-(9,9-dimethyl-9H-fluoren-2-yl)pyridine**

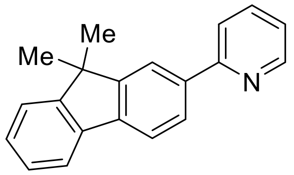


Prepared according to general procedure **A** (5.0 mmol scale) by using (9,9-dimethyl-9H-fluoren-2-yl)boronic acid to yield the titled compound as white solid (1.2 g, 89%).

**$^1$H NMR** (400 MHz, $CDCl_3$) δ 8.78 (ddd, *J* = 4.8, 1.8, 0.9 Hz, 1H), 8.22 (dd, *J* = 1.7, 0.7 Hz, 1H), 7.99 (dd, *J* = 7.9, 1.7 Hz, 1H), 7.87 – 7.77 (m, 3H), 7.76 – 7.70 (m, 1H), 7.53 – 7.47 (m, 1H), 7.43 – 7.34 (m, 2H), 7.22 (ddd, *J* = 7.3, 4.8, 1.2 Hz, 1H), 1.61 (s, 6H).

**$^{13}$C NMR** (100 MHz, $CDCl_3$) δ 157.7, 154.2, 154.2, 149.6, 140.1, 138.7, 138.5, 136.7, 127.6, 127.1, 126.0, 122.7, 121.9, 121.2, 120.6, 120.3, 120.2, 47.0, 27.2.

The data are consistent with those already published.[5]

**2-(furan-3-yl)pyridine**

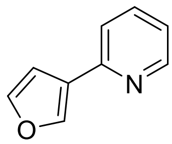


Prepared according to general procedure **A** (5.0 mmol scale) by using furan-3-ylboronic acid to yield the titled compound as colourless oil (219 mg, 30%).

**$^1$H NMR** (400 MHz, $CDCl_3$) δ 8.59 (ddd, *J* = 4.9, 1.8, 1.0 Hz, 1H), 8.02 (dd, *J* = 1.6, 0.8 Hz, 1H), 7.67 (td, *J* = 7.7, 1.8 Hz, 1H), 7.50 (t, *J* = 1.7 Hz, 1H), 7.45 (dt, *J* = 7.9, 1.1 Hz, 1H), 7.14 (ddd, *J* = 7.5, 4.9, 1.2 Hz, 1H), 6.90 (dd, *J* = 1.9, 0.9 Hz, 1H).

**$^{13}$C NMR** (100 MHz, $CDCl_3$) δ 152.0, 149.8, 144.0, 141.3, 136.7, 127.2, 121.8, 120.2, 108.7.

The data are consistent with those already published.[6]

**2-(thiophen-3-yl)pyridine**

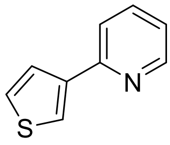


Prepared according to general procedure **A** (5.0 mmol scale) by using thiophen-3-ylboronic acid to yield the titled compound as colourless oil (627 mg, 78%).

**$^1$H NMR** (400 MHz, $CDCl_3$) δ 8.62 – 8.60 (m, 1H), 7.90 (dd, *J* = 3.0, 1.3 Hz, 1H), 7.69 – 7.64 (m, 2H), 7.59 (dt, *J* = 7.9, 1.2 Hz, 1H), 7.38 (dd, *J* = 5.0, 3.0 Hz, 1H), 7.14 (ddd, *J* = 7.4, 4.8, 1.3 Hz, 1H).

**$^{13}$C NMR** (100 MHz, $CDCl_3$) δ 153.5, 149.6, 142.2, 136.8, 126.4, 126.2, 123.6, 121.9, 120.3.

The data are consistent with those already published.[7]

**9-phenyl-3-(pyridin-2-yl)-9H-carbazole**

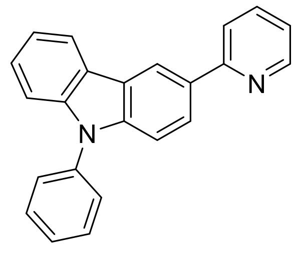


Prepared according to general procedure **A** (5.0 mmol scale) by using (9-phenyl-9H-carbazol-3-yl)boronic acid to yield the titled compound as white solid (1.5 g, 91%).

**$^1$H NMR** (400 MHz, $CDCl_3$) δ 8.83 (d, *J* = 1.7 Hz, 1H), 8.74 (ddd, *J* = 4.8, 1.9, 1.0 Hz, 1H), 8.24 (d, *J* = 7.7 Hz, 1H), 8.07 (dd, *J* = 8.6, 1.8 Hz, 1H), 7.86 (dt, *J* = 8.1, 1.1 Hz, 1H), 7.78 (td, *J* = 7.7, 1.9 Hz, 1H), 7.65 – 7.59 (m, 4H), 7.49 (tt, *J* = 8.6, 1.2 Hz, 2H), 7.43 (dd, *J* = 3.5, 1.0 Hz, 2H), 7.32 (ddd, *J* = 8.0, 4.6, 3.6 Hz, 1H), 7.22 (ddd, *J* = 7.4, 4.8, 1.1 Hz, 1H).

**$^{13}$C NMR** (100 MHz, $CDCl_3$) δ 158.3, 149.8, 141.6, 137.7, 136.9, 131.7, 130.1, 127.7, 127.2, 126.3, 125.2, 124.0, 123.8, 121.5, 120.7, 120.5, 120.4, 119.2, 110.09, 110.06.

The data are consistent with those already published.[8]

**2-(dibenzo[b,d]furan-2-yl)pyridine**

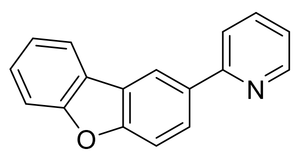

Prepared according to general procedure **A** (5.0 mmol scale) by using dibenzo[b,d]furan-2-ylboronic acid to yield the titled compound as white solid (728 mg, 59%).

**$^1$H NMR** (400 MHz, $CDCl_3$) δ 8.74 (d, *J* = 5.1 Hz, 1H), 8.64 (d, *J* = 1.9 Hz, 1H), 8.09 (dd, *J* = 8.6, 1.9 Hz, 1H), 8.03 (d, *J* = 8.0 Hz, 1H), 7.83 (d, *J* = 7.9 Hz, 1H), 7.78 (td, *J* = 7.5, 1.8 Hz, 1H), 7.65 (d, *J* = 8.6 Hz, 1H), 7.59 (d, *J* = 8.2 Hz, 1H), 7.48 (td, *J* = 7.8, 1.3 Hz, 1H), 7.37 (td, *J* = 7.4, 1.0 Hz, 1H), 7.27 – 7.24 (m, 1H).

**$^{13}$C NMR** (100 MHz, $CDCl_3$) δ 157.5, 157.0, 156.9, 149.7, 137.1, 134.5, 127.5, 126.4, 125.0, 124.4, 123.1, 122.0, 121.1, 120.7, 119.6, 111.9.

The data are consistent with those already published.[9]

**2-(dibenzo[b,d]thiophen-2-yl)pyridine**

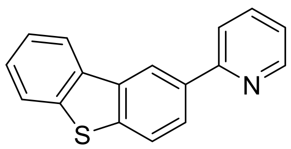

Prepared according to general procedure **A** (5.0 mmol scale) by using dibenzo[b,d]thiophen-2-ylboronic acid to yield the titled compound as white solid (569 mg, 73%).

**$^1$H NMR** (400 MHz, $CDCl_3$) δ 8.84 (d, *J* = 1.8 Hz, 1H), 8.76 (ddd, *J* = 4.9, 1.8, 1.0 Hz, 1H), 8.31 – 8.27 (m, 1H), 8.08 (dd, *J* = 8.4, 1.8 Hz, 1H), 7.94 (d, *J* = 8.4 Hz, 1H), 7.88 – 7.84 (m, 2H), 7.80 (td, *J* = 7.7, 1.8 Hz, 1H), 7.51 – 7.46 (m, 2H), 7.29 – 7.25 (m, 1H).

**$^{13}$C NMR** (100 MHz, $CDCl_3$) δ 157.3, 149.7, 140.5, 140.0, 137.2, 136.3, 135.9, 135.7, 127.1, 125.6, 124.7, 123.1, 123.0, 122.3, 122.1, 120.8, 120.2.

The data are consistent with those already published.[10]

**2-(4-vinylphenyl)pyridine**

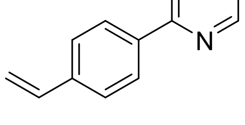

The 2-(4-vinylphenyl)pyridine was prepared on a 0.5 mmol scale according to the previously reported procedure.[11] The titled compound was obtained as colourless oil (72.4 mg, 80%).

**$^1$H NMR** (400 MHz, $CDCl_3$) δ 8.69 (d, *J* = 4.7 Hz, 1H), 7.98 (d, *J* = 8.4 Hz, 2H), 7.77 – 7.72 (m, 2H), 7.52 (d, *J* = 8.2 Hz, 2H), 7.22 (ddd, *J* = 5.5, 4.8, 3.0 Hz, 1H), 6.77 (dd, *J* = 17.6, 10.9 Hz, 1H), 5.83 (dd, *J* = 17.6, 0.9 Hz, 1H), 5.31 (dd, *J* = 10.9, 0.9 Hz, 1H).

**$^{13}$C NMR** (100 MHz, $CDCl_3$) δ 149.8, 138.8, 138.3, 136.9, 136.5, 127.2, 126.8, 122.2, 120.5, 114.6.

The data are consistent with those already published.[11]

**5-(dibenzylamino)-1-phenylpenta-2,4-dien-1-one**

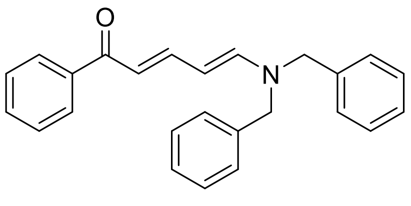

Prepared according to general procedure **B** by using 2-phenylpyridine to yield the titled compound as yellow solid (286 mg, 81%).

**$^1$H NMR** (400 MHz, $CDCl_3$) δ 7.94 – 7.91 (m, 2H), 7.70 (dd, *J* = 14.3, 11.6 Hz, 1H), 7.49 – 7.30 (m, 9H), 7.23 – 7.19 (m, 4H), 7.14 (d, *J* = 12.7 Hz, 1H), 6.62 (d, *J* = 14.3 Hz, 1H), 5.57 (t, *J* = 12.2 Hz, 1H), 4.35 (s, 4H).

**$^{13}$C NMR** (100 MHz, $CDCl_3$) δ 189.6, 152.3, 148.9, 139.9, 136.0, 131.4, 128.9, 128.2, 127.94, 127.92, 127.5, 113.7, 98.9.

**HRMS (+APCI)**: calcd for $C_{25}H_{24}NO$ $[M+H]^+$ 354,1852, found 354,1852.

The data are consistent with those already published.[1]

**5-(dibenzylamino)-1-(3,4-dimethoxyphenyl)penta-2,4-dien-1-one**

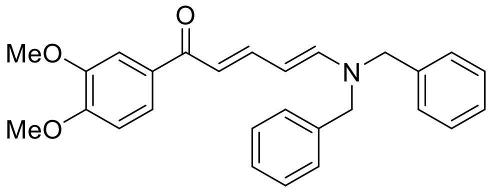


Prepared according to general procedure **B** by using 2-(3,4-dimethoxyphenyl)pyridine to yield the titled compound as yellow oil (352 mg, 85%).

**$^1$H NMR** (400 MHz, $CDCl_3$) δ 7.71 (dd, *J* = 14.2, 11.6 Hz, 1H), 7.58 – 7.54 (m, 2H), 7.37 – 7.28 (m, 6H), 7.20 – 7.12 (m, 5H), 6.85 (d, *J* = 8.4 Hz, 1H), 6.66 (d, *J* = 14.2 Hz, 1H), 5.55 (t, *J* = 12.2 Hz, 1H), 4.33 (s, 4H), 3.91 (s, 3H), 3.90 (s, 3H).

**$^{13}$C NMR** (100 MHz, $CDCl_3$) δ 187.7, 151.9, 151.9, 148.6, 147.9, 136.0, 132.6, 128.8, 127.8, 127.3, 121.7, 112.9, 110.5, 109.8, 98.7, 55.9, 55.8.

**HRMS (+APCI)**: calcd for $C_{27}H_{28}NO_3$ $[M+H]^+$ 414.2064, found 414.2061.

**5-(dibenzylamino)-1-(4-methoxyphenyl)penta-2,4-dien-1-one**

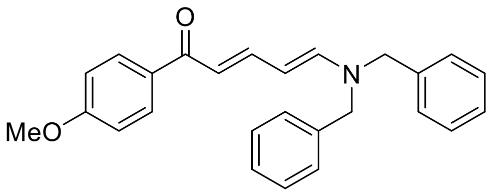


Prepared according to general procedure **B** by using 2-(4-methoxyphenyl)pyridine to yield the titled compound as yellow solid (337 mg, 88%).

**$^1$H NMR** (400 MHz, $CDCl_3$) δ 7.94 – 7.91ff (m, 2H), 7.65 (dd, *J* = 14.2, 11.6 Hz, 1H), 7.38 – 7.29 (m, 6H), 7.22 – 7.17 (m, 4H), 7.12 (d, *J* = 12.8 Hz, 1H), 6.92 – 6.89 (m, 2H), 6.62 (d, *J* = 14.2 Hz, 1H), 5.54 (t, *J* = 12.2 Hz, 1H), 4.35 (s, 4H), 3.85 (s, 3H).

**$^{13}$C NMR** (100 MHz, $CDCl_3$) δ 188.3, 162.5, 151.9, 147.9, 136.2, 132.7, 130.2, 129.0, 128.0, 127.6, 113.6, 113.5, 98.9, 55.5.

**HRMS (+APCI)**: calcd for $C_{26}H_{26}NO_2$ $[M+H]^+$ 384.1958, found 384.1949.

**mp:** 90-94 °C.

**5-(dibenzylamino)-1-(3-(methylthio)phenyl)penta-2,4-dien-1-one**

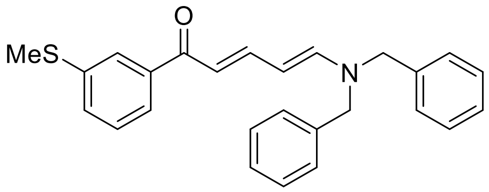


Prepared according to general procedure **B** by using 2-(3-(methylthio)phenyl)pyridine to yield the titled compound as yellow solid (332 mg, 83%).

**$^1$H NMR** (400 MHz, $CDCl_3$) δ 7.79 (t, *J* = 1.8 Hz, 1H), 7.70 – 7.63 (m, 2H), 7.39 – 7.29 (m, 8H), 7.20 – 7.13 (m, 5H), 6.56 (d, *J* = 14.2 Hz, 1H), 5.56 (t, *J* = 12.2 Hz, 1H), 4.36 (s, 4H), 2.51 (s, 3H).

**$^{13}$C NMR** (100 MHz, $CDCl_3$) δ 189.3, 152.6, 149.2, 140.6, 139.0, 136.0, 129.5, 129.1, 128.7, 128.1, 127.6, 125.8, 124.8, 113.6, 99.0, 15.9.

**HRMS (+APCI)**: calcd for $C_{26}H_{26}NOS$ $[M+H]^+$ 400.1730, found 400.1723.

**mp:** 80-82 °C.

**5-(dibenzylamino)-1-(p-tolyl)penta-2,4-dien-1-one**

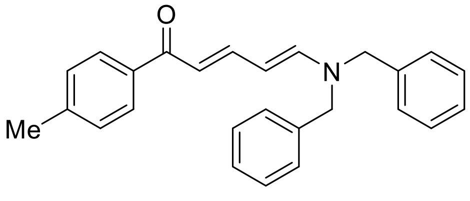


Prepared according to general procedure **B** by using 2-(p-tolyl)pyridine to yield the titled compound as yellow solid (359 mg, 98%).

**$^1$H NMR** (400 MHz, $CDCl_3$) δ 7.83 (d, *J* = 8.2 Hz, 2H), 7.67 (dd, *J* = 14.3, 11.6 Hz, 1H), 7.39 – 7.30 (m, 6H), 7.23 – 7.18 (m, 6H), 7.13 (d, *J* = 12.7 Hz, 1H), 6.62 (d, *J* = 14.3 Hz, 1H), 5.55 (dd, *J* = 12.7, 11.6 Hz, 1H), 4.35 (s, 4H), 2.39 (s, 3H).

**$^{13}$C NMR** (100 MHz, $CDCl_3$) δ 189.4, 152.1, 148.4, 142.0, 137.2, 136.1, 129.0, 129.0, 128.2, 128.0, 127.5, 113.8, 98.9, 21.7.

**HRMS (+APCI)**: calcd for $C_{26}H_{26}NO$ $[M+H]^+$ 368.2009, found 368.2015.

**mp:** 89-94 °C.

**1-([1,1'-biphenyl]-4-yl)-5-(dibenzylamino)penta-2,4-dien-1-one**

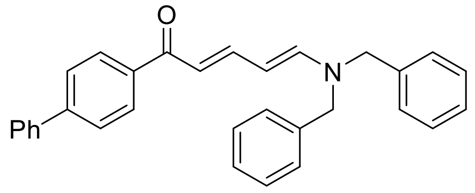


Prepared according to general procedure **B** by using 2-([1,1'-biphenyl]-4-yl)pyridine to yield the titled compound as greenish solid (284 mg, 66%).

**$^1$H NMR** (400 MHz, $CDCl_3$) δ 8.00 (d, *J* = 8.4 Hz, 2H), 7.70 (dd, *J* = 14.3, 11.6 Hz, 1H), 7.66 – 7.61 (m, 4H), 7.46 (t, *J* = 7.5 Hz, 2H), 7.39 – 7.30 (m, 7H), 7.21 – 7.19 (m, 4H), 7.16 (d, *J* = 12.7 Hz, 1H), 6.67 (d, *J* = 14.2 Hz, 1H), 5.58 (t, *J* = 12.2 Hz, 1H), 4.37 (s, 4H).

**$^{13}$C NMR** (100 MHz, $CDCl_3$) δ 189.2, 152.4, 148.8, 144.2, 140.5, 138.7, 136.1, 129.1, 129.0, 128.7, 128.1, 127.9, 127.6, 127.4, 127.1, 113.8, 99.0.

**HRMS (+APCI)**: calcd for $C_{31}H_{28}NO$ $[M+H]^+$ 430.2165, found 430.2159.

**mp:** 126-128 °C.

**5-(dibenzylamino)-1-(2,4-difluorophenyl)penta-2,4-dien-1-one**

Prepared according to general procedure **B** by using 2-(2,4-difluorophenyl)pyridine to yield the titled compound as yellow solid (253 mg, 65%).

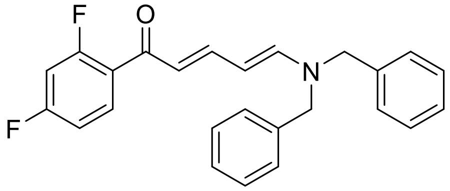

**$^{1}$H NMR** (400 MHz, $CDCl_3$) δ 7.78 (td, *J* = 8.5, 6.7 Hz, 1H), 7.59 (ddd, *J* = 14.0, 11.7, 1.9 Hz, 1H), 7.38 – 7.30 (m, 6H), 7.19 – 7.12 (m, 5H), 6.90 (td, *J* = 8.2, 2.5 Hz, 1H), 6.81 (ddd, *J* = 11.1, 8.9, 2.4 Hz, 1H), 6.42 (dd, *J* = 14.3, 2.9 Hz, 1H), 5.55 (t, *J* = 12.2 Hz, 1H), 4.35 (s, 4H).

**$^{19}$F NMR** (376 MHz, $CDCl_3$) δ -106.00 – -106.09 (m), -107.14 – -107.22 (m).

**$^{13}$C NMR** (100 MHz, $CDCl_3$) δ 186.4 (d, 31.8 Hz), 164.5 (dd, 263.2, 11.8 Hz), 161.3 (dd, 264.8, 12.2 Hz), 152.9, 149.7, 135.9, 132.5 (dd, 10.1, 4.8 Hz), 129.1, 128.1, 127.6, 125.3 (dd, 14.2, 3.8 Hz), 117.3 (d, 6.7 Hz), 111.7 (dd, 21.1, 3.7 Hz), 104.4 (dd, 27.6, 25.2 Hz), 99.0.

**HRMS (+APCI)**: calcd for $C_{25}H_{22}NOF_2$ $[M+H]^+$ 390.1664, found 390.1663.

**mp:** 86-88 °C.

**1-(4-chlorophenyl)-5-(dibenzylamino)penta-2,4-dien-1-one**

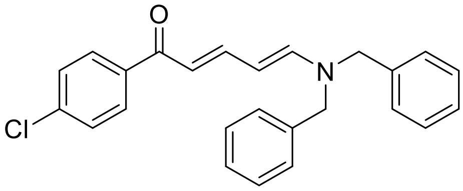

Prepared according to general procedure **B** by using 2-(4-chlorophenyl)pyridine to yield the titled compound as yellow solid (252 mg, 65%).

**$^{1}$H NMR** (400 MHz, $CDCl_3$) δ 7.85 (d, *J* = 8.5 Hz, 2H), 7.67 (dd, *J* = 14.2, 11.7 Hz, 1H), 7.39 – 7.32 (m, 8H), 7.20 – 7.14 (m, 5H), 6.55 (d, *J* = 14.2 Hz, 1H), 5.55 (t, *J* = 12.2 Hz, 1H), 4.36 (s, 4H).

**$^{13}$C NMR** (100 MHz, $CDCl_3$) δ 188.3, 152.7, 149.4, 138.3, 137.6, 136.0, 129.5, 129.1, 128.6, 128.1, 127.6, 113.1, 99.0.

**HRMS (+APCI)**: calcd for $C_{25}H_{23}NOCl$ $[M+H]^+$ 388.1463, found 388.1464.

**mp:** 98-100 °C

**1-(4-bromophenyl)-5-(dibenzylamino)penta-2,4-dien-1-one**

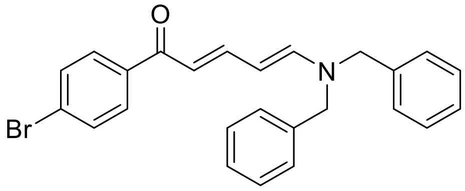

Prepared according to general procedure **B** by using 2-(4-bromophenyl)pyridine to yield the titled compound as yellow solid (331 mg, 77%).

**$^{1}$H NMR** (400 MHz, $CDCl_3$) δ 7.77 (d, *J* = 8.5 Hz, 2H), 7.67 (dd, *J* = 14.1, 11.7 Hz, 1H), 7.54 (d, *J* = 8.6 Hz, 2H), 7.39 – 7.31 (m, 6H), 7.20 – 7.15 (m, 5H), 6.54 (d, *J* = 14.2 Hz, 1H), 5.55 (t, *J* = 12.2 Hz, 1H), 4.36 (s, 4H).

**$^{13}$C NMR** (100 MHz, $CDCl_3$) δ 188.4, 152.8, 149.5, 138.7, 135.9, 131.5, 129.7, 129.1, 128.1, 127.6, 126.2, 113.0, 99.0.

**HRMS (+APCI)**: calcd for $C_{25}H_{23}NOBr$ $[M+H]^+$ 432.0958, found 432.0956.

**mp:** 120-124 °C.

**5-(dibenzylamino)-1-(4-nitrophenyl)penta-2,4-dien-1-one**

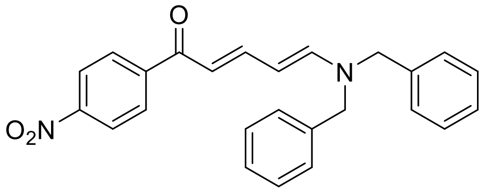

Prepared according to general procedure **B** by using 2-(4-nitrophenyl)pyridine to yield the titled compound as dark red solid (159 mg, 40%).

**$^{1}$H NMR** (400 MHz, $CDCl_3$) δ 8.27 – 8.23 (m, 2H), 8.03 – 8.00 (m, 2H), 7.71 (dd, *J* = 14.1, 11.8 Hz, 1H), 7.40 – 7.31 (m, 7H), 7.23 – 7.18 (m, 5H), 6.54 (d, *J* = 14.1 Hz, 1H), 5.60 (t, *J* = 12.2 Hz, 1H), 4.39 (s, 4H).

**$^{13}$C NMR** (100 MHz, $CDCl_3$) δ 187.5, 153.8, 150.9, 149.4, 145.4, 135.6, 129.2, 128.9, 128.7, 128.3, 127.6, 123.6, 112.8, 99.3.

**HRMS (+APCI)**: calcd for $C_{25}H_{23}N_2O_3$ $[M+H]^+$ 399.1703, found 399.1696.

**mp:** 92-94 °C.

**5-(dibenzylamino)-1-(naphthalen-2-yl)penta-2,4-dien-1-one**

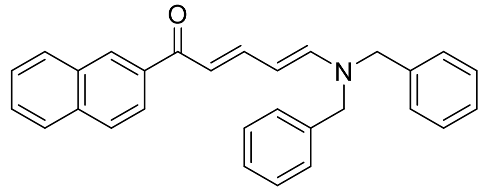

Prepared according to general procedure **B** by using 2-(naphthalen-2-yl)pyridine to yield the titled compound as yellow solid (343 mg, 85%).

**$^{1}$H NMR** (400 MHz, $CDCl_3$) δ 8.43 (s, 1H), 8.04 (dd, *J* = 8.6, 1.7 Hz, 1H), 7.93 (dd, *J* = 7.6, 1.8 Hz, 1H), 7.88 – 7.84 (m, 2H), 7.74 (dd, *J* = 14.2, 11.6 Hz, 1H), 7.56 – 7.49 (m, 2H), 7.40 – 7.30 (m, 6H), 7.22 – 7.15 (m, 5H), 6.78 (d, *J* = 14.2 Hz, 1H), 5.61 (t, *J* = 12.2 Hz, 1H), 4.37 (s, 4H)

**$^{13}$C NMR** (100 MHz, $CDCl_3$) δ 189.6, 152.4, 148.9, 137.3, 136.1, 135.0, 132.8, 129.4, 129.1, 128.8, 128.08, 128.05, 127.8, 127.6, 127.6, 126.4, 124.9, 113.8, 99.0.

**HRMS (+APCI)**: calcd for $C_{29}H_{26}NO$ $[M+H]^+$ 404.2009, found 404.2000.

**mp:** 82-84 °C.

**5-(dibenzylamino)-1-(9,9-dimethyl-9H-fluoren-2-yl)penta-2,4-dien-1-one**

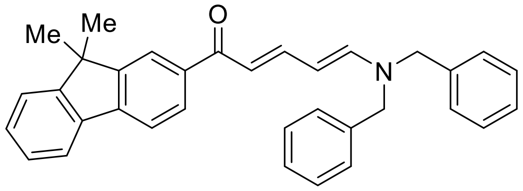

Prepared according to general procedure **B** by using 2-(9,9-dimethyl-9H-fluoren-2-yl)pyridine to yield the titled compound as orange solid (400 mg, 85%).

**$^{1}$H NMR** (400 MHz, $CDCl_3$) δ 8.06 (d, *J* = 1.5 Hz, 1H), 7.95 (dd, *J* = 7.9, 1.6 Hz, 1H), 7.77 – 7.71 (m, 3H), 7.47 – 7.45 (m, 1H), 7.40 – 7.33 (m, 8H), 7.22 – 7.18 (m, 4H), 7.17 (d, *J* = 12.7 Hz, 1H), 6.73 (d, *J* = 14.2 Hz, 1H), 5.60 (t, *J* = 12.2 Hz, 1H), 4.37 (s, 4H), 1.52 (s, 6H).

**$^{13}$C NMR** (100 MHz, $CDCl_3$) δ 189.4, 154.8, 153.7, 152.2, 148.5, 142.6, 138.9, 138.5, 136.1, 129.0, 128.5, 128.3, 128.1, 128.0, 127.6, 127.2, 122.8, 122.5, 120.8, 119.6, 114.0, 99.0, 47.1, 27.1.

**HRMS (+APCI)**: calcd for $C_{34}H_{32}NO$ $[M+H]^+$ 470.2478, found 470.2483.

**mp:** 84-88 °C.

**5-(dibenzylamino)-1-(furan-3-yl)penta-2,4-dien-1-one**

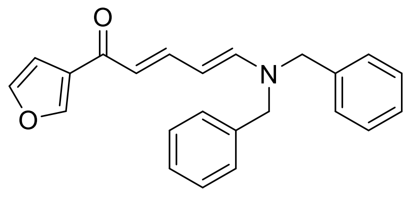

Prepared according to general procedure **B** by using 2-(furan-3-yl)pyridine to yield the titled compound as brown solid (205 mg, 60%).

**$^{1}$H NMR** (400 MHz, $CDCl_3$) δ 7.95 (s, 1H), 7.63 (dd, *J* = 14.3, 11.7 Hz, 1H), 7.40 – 7.29 (m, 7H), 7.20 – 7.18 (m, 4H), 7.12 (d, *J* = 12.8 Hz, 1H), 6.80 (d, *J* = 1.9 Hz, 1H), 6.27 (d, *J* = 14.2 Hz, 1H), 5.48 (t, *J* = 12.2 Hz, 1H), 4.35 (s, 4H).

**$^{13}$C NMR** (100 MHz, $CDCl_3$) δ 183.8, 152.2, 147.4, 145.8, 143.6, 136.1, 129.2, 129.0, 128.0, 127.5, 114.8, 109.4, 98.4.

**HRMS (+APCI)**: calcd for $C_{23}H_{22}NO_2$ $[M+H]^+$ 344.1645, found 344.1645.

**mp:** 92-94 °C.

**5-(dibenzylamino)-1-(thiophen-3-yl)penta-2,4-dien-1-one**

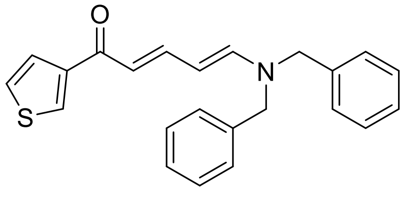

Prepared according to general procedure **B** by using 2-(thiophen-3-yl)pyridine to yield the titled compound as yellow oil (262 mg, 73%).

**$^{1}$H NMR** (400 MHz, $CDCl_3$) δ 7.94 (dd, *J* = 3.0, 1.2 Hz, 1H), 7.65 (dd, *J* = 14.2, 11.7 Hz, 1H), 7.56 (dd, *J* = 5.1, 1.2 Hz, 1H), 7.39 – 7.30 (m, 6H), 7.28 – 7.26 (m, 1H), 7.20 – 7.18 (m, 4H), 7.13 (d, *J* = 12.8 Hz, 1H), 6.48 (d, *J* = 14.2 Hz, 1H), 5.53 (t, *J* = 12.2 Hz, 1H), 4.35 (s, 4H).

**$^{13}$C NMR** (100 MHz, $CDCl_3$) δ 183.7, 152.2, 148.1, 144.5, 136.1, 129.7, 129.0, 128.0, 127.6, 125.7, 114.5, 98.7.

**HRMS (+APCI)**: calcd for $C_{23}H_{22}NOS$ $[M+H]^+$ 360,1417, found 360.1416.

**5-(dibenzylamino)-1-(9-phenyl-9*H*-carbazol-3-yl)penta-2,4-dien-1-one**

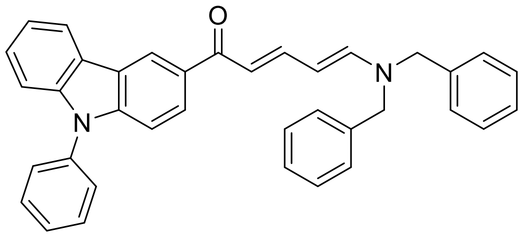

Prepared according to general procedure **B** by using 9-phenyl-3-(pyridin-2-yl)-9H-carbazole to yield the titled compound as yellow solid (400 mg, 77%).

**$^{1}$H NMR** (400 MHz, $CDCl_3$) δ 8.81 (d, *J* = 1.7 Hz, 1H), 8.20 (d, *J* = 7.4 Hz, 1H), 8.08 (dd, *J* = 8.7, 1.7 Hz, 1H), 7.76 (dd, *J* = 14.2, 11.6 Hz, 1H), 7.62 (t, *J* = 7.7 Hz, 2H), 7.57 – 7.55 (m, 2H), 7.51 – 7.47 (m, 1H), 7.45 – 7.30 (m, 10H), 7.23 – 7.21 (m, 4H), 7.16 (d, *J* = 12.8 Hz, 1H), 6.87 (d, *J* = 14.2 Hz, 1H), 5.63 (t, *J* = 12.2 Hz, 1H), 4.37 (s, 4H).

**$^{13}$C NMR** (100 MHz, $CDCl_3$) δ 189.1, 151.8, 147.8, 143.0, 141.7, 137.3, 136.3, 132.1, 130.1, 129.0, 128.0, 127.9, 127.6, 127.2, 126.7, 126.4, 123.8, 123.2, 121.3, 120.7, 114.0, 110.2, 109.3, 99.0.

**HRMS (+APCI)**: calcd for $C_{37}H_{31}N_2O$ $[M+H]^+$ 519.2431, found 519.2437.

**mp:** 140-145 °C.

**1-(dibenzo[*b,d*]furan-2-yl)-5-(dibenzylamino)penta-2,4-dien-1-one**

Prepared according to general procedure **B** by using 2-(dibenzo[b,d]furan-2-yl)pyridine to yield the titled compound as yellow solid (324 mg, 73%).

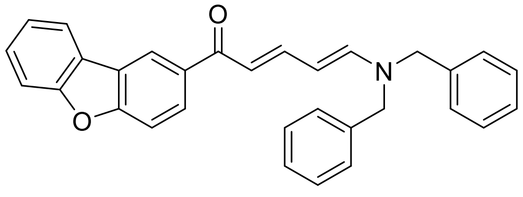

**$^1$H NMR** (400 MHz, $CDCl_3$) δ 8.58 (d, *J* = 1.8 Hz, 1H), 8.09 (dd, *J* = 8.6, 1.9 Hz, 1H), 7.99 (d, *J* = 7.0 Hz, 1H), 7.74 (dd, *J* = 14.2, 11.7 Hz, 1H), 7.57 (dd, *J* = 8.4, 4.4 Hz, 2H), 7.48 (td, *J* = 7.8, 1.4 Hz, 1H), 7.40 – 7.30 (m, 7H), 7.22 – 7.16 (m, 5H), 6.76 (d, *J* = 14.2 Hz, 1H), 5.61 (t, *J* = 12.2 Hz, 1H), 4.38 (s, 4H).

**$^{13}$C NMR** (100 MHz, $CDCl_3$) δ 188.9, 158.3, 156.9, 152.4, 148.8, 136.1, 135.2, 129.1, 128.1, 127.8, 127.6, 127.6, 124.4, 124.3, 123.2, 121.2, 121.1, 113.7, 111.9, 111.3, 99.0.

**HRMS (+APCI)**: calcd for $C_{31}H_{26}NO_2$ $[M+H]^+$ 444.1958, found 444.1959.

**mp:** 118-122 °C.

**1-(dibenzo[*b,d*]thiophen-2-yl)-5-(dibenzylamino)penta-2,4-dien-1-one**

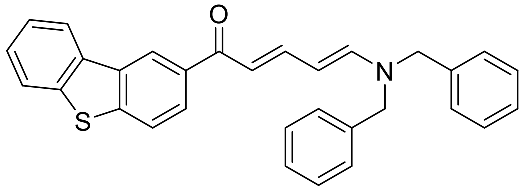

Prepared according to general procedure **B** by using 2-(dibenzo[b,d]thiophen-2-yl)pyridine to yield the titled compound as yellow solid (322 mg, 70%).

**$^1$H NMR** (400 MHz, $CDCl_3$) δ 8.75 (d, *J* = 1.6 Hz, 1H), 8.26 – 8.22 (m, 1H), 8.04 (dd, *J* = 8.4, 1.7 Hz, 1H), 7.88 – 7.83 (m, 2H), 7.77 (dd, *J* = 14.2, 11.7 Hz, 1H), 7.50 – 7.45 (m, 2H), 7.40 – 7.30 (m, 6H), 7.22 – 7.16 (m, 5H), 6.78 (d, *J* = 14.2 Hz, 1H), 5.62 (t, *J* = 12.2 Hz, 1H), 4.37 (s, 4H).

**$^{13}$C NMR** (100 MHz, $CDCl_3$) δ 189.1, 152.5, 149.0, 142.9, 139.8, 136.5, 136.1, 135.7, 135.6, 129.1, 128.1, 127.6, 127.1, 126.4, 124.8, 123.0, 122.5, 122.0, 121.5, 113.6, 99.1.

**HRMS (+APCI)**: calcd for $C_{31}H_{26}NOS$ $[M+H]^+$ 460.1730, found 460.1718.

**mp:** 131-136 °C.

**2-chloro-N-(4-chloro-3-(5-(dibenzylamino)penta-2,4-dienoyl)phenyl)-4-(methylsulfonyl)benzamide**

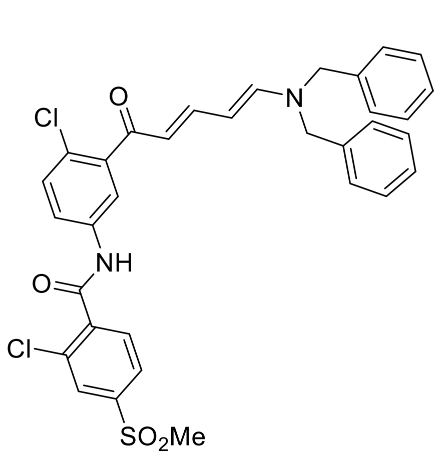

2-chloro-N-(4-chloro-3-(pyridin-2-yl)phenyl)-4-(methylsulfonyl)benzamide (0.4 mmol, 168 mg, 1.0 equiv.) was added into an oven-dried microwave vial (20 mL) and the vial was subjected to three cycles of vacuum/$N_2$ backfill. Then anhydrous EtOAc was introduced and the resulting mixture was stirred at 60 °C for 10 min until full dissolution of the starting material. Then the reaction was cooled -78 °C and $Tf_2O$ (1.0 equiv.) was added dropwise. The reaction was stirred at -78 °C for 30 min, followed by addition of dibenzylamine (1.0 equiv., 1.0 M in EtOAc) and collidine (1.0 equiv.). The reaction was stirred at -78 °C for a further 30 min. After removing the cooling bath, the reaction continued stirring at room temperature for 30 min. The reaction was washed with water three times and the organic phase was concentrated. The residue was dissolved in EtOAc/$H_2O$ (10/1, 0.1 M) and $K_2CO_3$ (2.0 equiv.) was added into the vial. The mixture was stirred at 100 °C overnight, then cooled to room temperature, washed with brine and extracted with EtOAc (x3). The combine organic layers were dried over $Na_2SO_4$ and concentrated

*in vacuo* and the product was purified by flash column chromatography on silica gel. The desired product was obtained as yellow solid (82 mg, 33%).

**$^1$H NMR** (400 MHz, $CDCl_3$) δ 9.51 (s, 1H), 7.95 (d, *J* = 1.7 Hz, 1H), 7.91 (dd, *J* = 8.7, 2.6 Hz, 1H), 7.83 (dd, *J* = 8.0, 1.7 Hz, 1H), 7.76 (d, *J* = 8.0 Hz, 1H), 7.38 – 7.29 (m, 8H), 7.18 – 7.15 (m, 4H), 7.09 (dd, *J* = 13.1, 9.2 Hz, 2H), 5.98 (d, *J* = 14.5 Hz, 1H), 5.48 (t, *J* = 12.2 Hz, 1H), 4.35 (s, 4H), 3.03 (s, 3H).

**$^{13}$C NMR** (100 MHz, $CDCl_3$) δ 164.0, 154.0, 142.8, 140.9, 140.7, 136.5, 135.6, 132.8, 130.7, 130.6, 129.2, 129.1, 128.2, 127.6, 126.4, 125.9, 122.6, 120.9, 117.3, 99.0, 44.6.

**HRMS (+APCI)**: calcd for $C_{33}H_{29}N_2O_4Cl_2S$ $[M+H]^+$ 619.1220, found 619.1204.

**mp:** 96-99 °C.

**5-(dibenzylamino)-1-(2-methylbenzofuro[2,3-*b*]pyridin-8-yl)penta-2,4-dien-1-one**

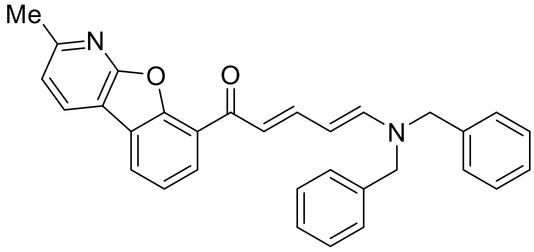


Prepared according to general procedure **B** by using 2-methyl-8-(pyridin-2-yl)benzofuro[2,3-b]pyridine to yield the titled compound as yellow solid (276 mg, 60%).

**$^1$H NMR** (400 MHz, $CDCl_3$) δ 8.10 – 8.00 (m, 2H), 7.90 (dd, *J* = 7.5, 1.4 Hz, 1H), 7.76 (dd, *J* = 14.2, 11.7 Hz, 1H), 7.36 – 7.24 (m, 7H), 7.19 (d, *J* = 3.2 Hz, 1H), 7.16 – 7.08 (m, 6H), 5.68 (t, *J* = 12.2 Hz, 1H), 4.30 (s, 4H), 2.61 (s, 3H).

**$^{13}$C NMR** (100 MHz, $CDCl_3$) δ 186.0, 163.1, 156.7, 153.0, 152.3, 149.4, 136.0, 130.0, 129.12, 129.05, 128.0, 127.7, 125.6, 123.8, 123.6, 123.4, 119.2, 117.4, 113.5, 99.8, 24.7.

**HRMS (+APCI)**: calcd for $C_{31}H_{27}N_2O_2$ $[M+H]^+$ 459.2067, found 459.2065.

**mp:** 116-120 °C.

**5-(dibenzylamino)-1,3-diphenylpenta-2,4-dien-1-one**

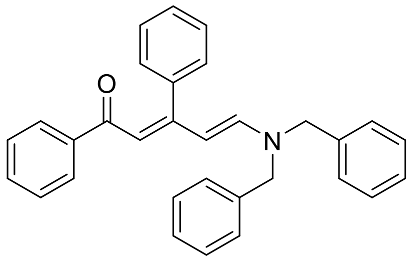


5-(Dibenzylamino)-1,3-diphenylpenta-2,4-dien-1-one was prepared on a 0.5 mmol according to the previously reported procedure.[1] The titled compound was obtained as orange gum (124 mg, 58%).

**$^1$H NMR** (400 MHz, $CDCl_3$) δ 7.94 – 7.92 (m, 2H), 7.89 (d, *J* = 13.5 Hz, 1H), 7.46 – 7.28 (m, 15H), 7.26 – 7.19 (m, 3H), 6.98 (d, *J* = 13.5 Hz, 1H), 6.31 (s, 1H), 4.35 (s, 4H).

**$^{13}$C NMR** (100 MHz, $CDCl_3$) δ 189.8, 160.9, 153.8, 142.3, 141.7, 136.2, 131.0, 129.2, 128.9, 128.3, 128.2, 128.1, 127.9, 127.7, 111.4, 99.6.

**HRMS (+APCI)**: calcd for $C_{31}H_{28}NO$ $[M+H]^+$ 430.2165, found 430.2159.

**5-(dibenzylamino)-1-phenyl-3-(*m*-tolyl)penta-2,4-dien-1-one**

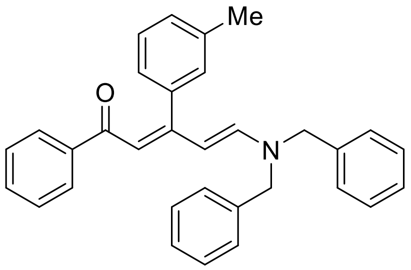


5-(Dibenzylamino)-1-phenyl-3-(*m*-tolyl)penta-2,4-dien-1-one was prepared on a 0.5 mmol according to the previously reported procedure.[1] The titled compound was obtained as orange gum (113 mg, 51%).

**$^1$H NMR** (400 MHz, $CDCl_3$) δ 7.93 (dt, *J* = 6.7, 1.6 Hz, 2H), 7.87 (d, *J* = 13.5 Hz, 1H), 7.46 – 7.19 (m, 17H), 6.99 (d, *J* = 13.5 Hz, 1H), 6.30 (s, 1H), 4.35 (s, 4H), 2.40 (s, 3H).

**$^{13}$C NMR** (100 MHz, $CDCl_3$) δ 189.8, 161.1, 153.9, 142.2, 141.8, 137.9, 131.0, 129.8, 128.9, 128.9, 128.2, 128.0, 127.9, 127.7, 126.4, 111.2, 99.6, 21.6.

**HRMS (+APCI)**: calcd for $C_{32}H_{30}NO$ $[M+H]^+$ 444.2322, found 444.2314.

**3-([1,1'-biphenyl]-4-yl)-5-(dibenzylamino)-1-phenylpenta-2,4-dien-1-one**

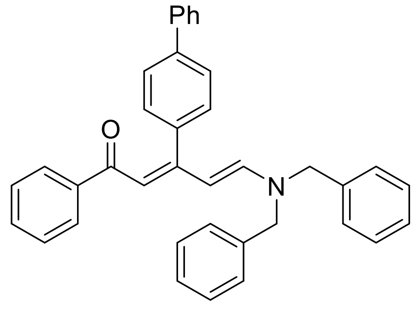


3-([1,1'-Biphenyl]-4-yl)-5-(dibenzylamino)-1-phenylpenta-2,4-dien-1-one was prepared on a 0.5 mmol according to the previously reported procedure.[1] The titled compound was obtained as orange gum (131 mg, 52%).

**$^1$H NMR** (400 MHz, $CDCl_3$) δ 7.95 – 7.93 (m, 2H), 7.89 (d, *J* = 13.5 Hz, 1H), 7.66 – 7.62 (m, 4H), 7.52 – 7.49 (m, 3H), 7.48 – 7.45 (m, 2H), 7.43 – 7.29 (m, 10H), 7.22 – 7.21 (m, 3H), 7.04 (d, *J* = 13.5 Hz, 1H), 6.35 (s, 1H), 4.37 (s, 4H).

**$^{13}$C NMR** (100 MHz, $CDCl_3$) δ 189.8, 160.5, 153.8, 141.8, 141.3, 141.1, 140.7, 136.2, 131.1, 129.7, 129.00, 128.95, 128.3, 127.97, 127.95, 127.8, 127.6, 127.2, 127.0, 111.3, 99.6.

**HRMS (+APCI)**: calcd for $C_{37}H_{32}NO$ $[M+H]^+$ 506.2478, found 506.2474.

**phenyl(thiophen-2-yl)methanone**

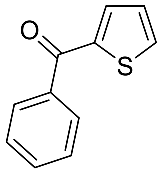


Prepared according to general procedure **C,** the titled compound was obtained as light yellow solid (22.2 mg, 59%).

2 mmol scale reaction:

A 50 mL oven-dried flask was charged with 5-(dibenzylamino)-1-phenylpenta-2,4-dien-1-one (2.0 mmol, 1.0 equiv.), $S_8$ (6.0 mmol, 3.0 equiv.) and sealed. The vial was evacuated and backfilled with $N_2$ three times. Then anhydrous DMF (20 mL, 0.1 M) was added and the reaction mixture was stirred at 60 °C overnight. After cooling to room temperature, the resulting mixture was diluted with EtOAc and washed with water (x3). The organic layer was dried over $Na_2SO_4$, filtered and concentrated *in vacuo*. The crude product was purified by flash column chromatography to deliver the corresponding product as light yellow solid (199 mg, 53% yield).

Telescoped process:

An oven-dried microwave vial was sealed and placed on the Schlenk line to vacuum and refill with nitrogen three times. Then 2-phenylpyridine (0.2 mmol, 1.0 equiv.), EtOAc (2 mL, 0.1 M) was added under a flow of nitrogen. The reaction solution was cooled to -78 °C and $Tf_2O$ (1.0 equiv.) was added dropwise. After the

addition was completed, the reaction was kept -78 °C for 30 min and then a solution of dibenzylamine (1.2 equiv.) in EtOAc (1.0 M) was added slowly followed by collidine (1.0 equiv.). The reaction mixture was stirred at -78 °C for an additional 30 min and then allowed to warm to room temperature and stirred for 30 min. Then $K_2CO_3$ (2,0 equiv.) and $H_2O$ (0.2 mL) were added into the vial and the mixture was stirred at 100 °C overnight. Then EtOAc and $H_2O$ were removed under reduced pressure. $S_8$ (0.6 mmol, 3.0 equiv.) was added into the microwave vial. Dry DMF (2 mL) was added under nitrogen and the reaction mixture was stirred at 60 °C overnight. After cooling to room temperature, the reaction was diluted with EtOAc and washed with water (x3). The organic phase was dried over $Na_2SO_4$, filtered and concentrated *in vacuo*. The product was isolated as light yellow solid (16.2 mg, 43%) by using flash column chromatography.

**$^1$H NMR** (400 MHz, $CDCl_3$) δ 7.88 – 7.85 (m, 2H), 7.71 (dd, *J* = 4.9, 1.1 Hz, 1H), 7.64 (dd, *J* = 3.8, 1.1 Hz, 1H), 7.61 – 7.56 (m, 1H), 7.49 (t, *J* = 7.5 Hz, 2H), 7.15 (dd, *J* = 5.0, 3.8 Hz, 1H).

**$^{13}$C NMR** (100 MHz, $CDCl_3$) δ 188.3, 143.7, 138.2, 134.9, 134.3, 132.4, 129.2, 128.5, 128.1.

**HRMS (+ESI)**: calcd for $C_{11}H_8OSNa$ $[M+Na]^+$ 211.0188, found 211.0184.

**mp:** 46-48 °C.

The data are consistent with those already published.[12]

**(3,4-dimethoxyphenyl)(thiophen-2-yl)methanone**

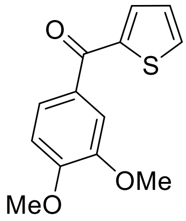


Prepared according to general procedure **C,** the titled compound was obtained as light yellow solid (32.2 mg, 65%).

**$^1$H NMR** (400 MHz, $CDCl_3$) δ 7.70 – 7.66 (m, 2H), 7.56 (dd, *J* = 8.3, 2.0 Hz, 1H), 7.47 (d, *J* = 2.0 Hz, 1H), 7.16 (dd, *J* = 5.0, 3.8 Hz, 1H), 6.93 (d, *J* = 8.4 Hz, 1H), 3.97 (s, 3H), 3.95 (s, 3H).

**$^{13}$C NMR** (100 MHz, $CDCl_3$) δ 186.9, 153.0, 149.1, 143.8, 134.2, 133.6, 130.9, 127.9, 124.1, 111.9, 110.1, 56.2, 56.2.

**HRMS (+ESI)**: calcd for $C_{13}H_{12}O_3SNa$ $[M+Na]^+$ 271.0399, found 271.0391.

**mp:** 66-68 °C.

**(4-methoxyphenyl)(thiophen-2-yl)methanone**

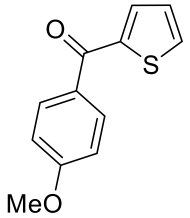


Prepared according to general procedure **C,** the titled compound was obtained as light yellow solid (27.0 mg, 62%).

**$^1$H NMR** (400 MHz, $CDCl_3$) δ 7.92 – 7.88 (m, 2H), 7.68 (dd, *J* = 4.9, 1.1 Hz, 1H), 7.64 (dd, *J* = 3.8, 1.2 Hz, 1H), 7.15 (dd, *J* = 5.0, 3.8 Hz, 1H), 7.00 – 6.96 (m, 2H), 3.89 (s, 3H).

**$^{13}$C NMR** (100 MHz, $CDCl_3$) δ 187.0, 163.2, 144.0, 134.1, 133.6, 131.7, 130.8, 127.9, 113.8, 55.6.

**HRMS (+APCI)**: calcd for $C_{12}H_{11}O_2S$ $[M+H]^+$ 219.0474, found 219.0468.

**mp:** 58-60 °C.

**(3-(methylthio)phenyl)(thiophen-2-yl)methanone**

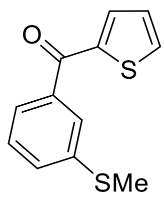

Prepared according to general procedure **C,** the titled compound was obtained as light yellow oil (35.2 mg, 75%).

**$^1$H NMR** (400 MHz, $CDCl_3$) δ 7.76 (dd, *J* = 5.0, 1.1 Hz, 1H), 7.72 (t, *J* = 1.8 Hz, 1H), 7.67 (dd, *J* = 3.8, 1.2 Hz, 1H), 7.62 (dt, *J* = 7.4, 1.5 Hz, 1H), 7.48 (ddd, *J* = 7.9, 2.0, 1.2 Hz, 1H), 7.42 (t, *J* = 7.7 Hz, 1H), 7.19 (dd, *J* = 4.9, 3.8 Hz, 1H), 2.55 (s, 3H).

**$^{13}$C NMR** (100 MHz, $CDCl_3$) δ 187.9, 143.5, 139.7, 138.8, 135.1, 134.6, 130.1, 128.8, 128.2, 126.5, 125.8, 15.7.

**HRMS (+ESI)**: calcd for $C_{12}H_{10}OS_2Na$ $[M+Na]^+$ 257.0052, found 257.0060.

**thiophen-2-yl(p-tolyl)methanone**

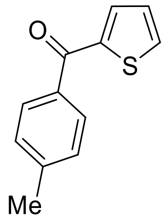

Prepared according to general procedure **C,** the titled compound was obtained as light yellow solid (25.8 mg, 64%).

**$^1$H NMR** (400 MHz, $CDCl_3$) δ 7.80 – 7.77 (m, 2H), 7.70 (dd, *J* = 4.9, 1.1 Hz, 1H), 7.65 (dd, *J* = 3.8, 1.2 Hz, 1H), 7.30 (d, *J* = 7.8 Hz, 2H), 7.16 (dd, *J* = 5.0, 3.8 Hz, 1H), 2.45 (s, 3H).

**$^{13}$C NMR** (100 MHz, $CDCl_3$) δ 188.1, 143.9, 143.2, 135.5, 134.6, 134.0, 129.5, 129.2, 128.0, 21.8.

**HRMS (+APCI)**: calcd for $C_{12}H_{11}OS$ $[M+H]^+$ 203.0525, found 203.0520.

**mp:** 70-72 °C.

**[1,1'-biphenyl]-4-yl(thiophen-2-yl)methanone**

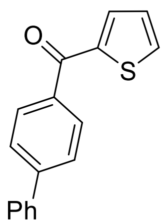

Prepared according to general procedure **C,** the titled compound was obtained as light yellow solid (47.4 mg, 90%).

**$^1$H NMR** (400 MHz, $CDCl_3$) δ 7.97 (d, *J* = 8.3 Hz, 2H), 7.75 – 7.70 (m, 4H), 7.66 (dt, *J* = 6.0, 1.3 Hz, 2H), 7.51 – 7.47 (m, 2H), 7.44 – 7.39 (m, 1H), 7.19 (dd, *J* = 4.9, 3.8 Hz, 1H).

**$^{13}$C NMR** (100 MHz, $CDCl_3$) δ 187.9, 145.2, 143.8, 140.0, 136.9, 134.8, 134.3, 130.0, 129.1, 128.3, 128.1, 127.4, 127.2.

**HRMS (+ESI)**: calcd for $C_{17}H_{12}OSNa$ $[M+Na]^+$ 287.0501, found 287.0495.

**mp:** 76-78 °C.

**(2,4-difluorophenyl)(thiophen-2-yl)methanone**

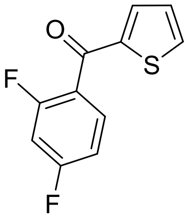

Prepared according to general procedure **C,** the titled compound was obtained as light yellow solid (25.0 mg, 56%).

**$^1$H NMR** (400 MHz, $CDCl_3$) δ 7.76 (dd, *J* = 5.0, 1.2 Hz, 1H), 7.64 – 7.58 (m, 1H), 7.53 (ddd, *J* = 3.6, 2.2, 1.2 Hz, 1H), 7.15 (dd, *J* = 4.9, 3.8 Hz, 1H), 7.02 – 6.97 (m, 1H), 6.96 – 6.91 (m, 1H).

**$^{19}$F NMR** (376 MHz, $CDCl_3$) δ -103.95 – -104.04 (m), -107.48 (tdd, *J* = 10.0, 7.3, 2.1 Hz).

**$^{13}$C NMR** (100 MHz, $CDCl_3$) δ 183.9, 164.8 (dd, 252.9, 11.6 Hz), 160.6 (dd, 254.1, 12.2 Hz), 144.0, 135.5, 135.4 (d, *J* = 2.9 Hz), 132.0 (dd, 10.2, 4.3 Hz), 128.4, 123.7 (d, 14.9 Hz), 111.9 (dd, 21.5, 3.6 Hz), 105.0 (t, 25.3 Hz).

**HRMS (+APCI)**: calcd for $C_{11}H_7OSF_2$ $[M+H]^+$ 225.0180, found 225.0172.

**mp:** 49-52 °C.

**(4-chlorophenyl)(thiophen-2-yl)methanone**

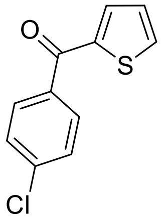

Prepared according to general procedure **C,** the titled compound was obtained as light yellow solid (24.8 mg, 56%).

**$^1$H NMR** (400 MHz, $CDCl_3$) δ 7.83 – 7.80 (m, 2H), 7.74 (dd, *J* = 5.0, 1.2 Hz, 1H), 7.62 (dd, *J* = 3.8, 1.2 Hz, 1H), 7.49 – 7.46 (m, 2H), 7.17 (dd, *J* = 5.0, 3.8 Hz, 1H).

**$^{13}$C NMR** (100 MHz, $CDCl_3$) δ 187.1, 143.3, 138.8, 136.5, 134.9, 134.7, 130.7, 128.9, 128.2.

**HRMS (+APCI)**: calcd for $C_{11}H_8OClS$ $[M+H]^+$ 222.9979, found 222.9971.

**mp:** 66-68 °C.

**(4-bromophenyl)(thiophen-2-yl)methanone**

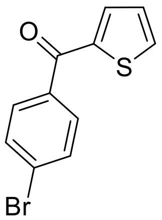

Prepared according to general procedure **C,** the titled compound was obtained as light yellow solid (33.6 mg, 63%).

**$^1$H NMR** (400 MHz, $CDCl_3$) δ 7.76 – 7.72 (m, 3H), 7.66 – 7.61 (m, 3H), 7.17 (dd, *J* = 5.0, 3.8 Hz, 1H).

**$^{13}$C NMR** (100 MHz, $CDCl_3$) δ 187.2, 143.3, 137.0, 134.9, 134.7, 131.9, 130.8, 128.2, 127.4.

**HRMS (+APCI)**: calcd for $C_{11}H_8OBrS$ $[M+H]^+$ 266.9474, found 266.9467.

The data are consistent with those already published.[13]

**(4-nitrophenyl)(thiophen-2-yl)methanone**

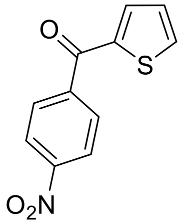

Prepared according to general procedure **C,** the titled compound was obtained as light yellow solid (14.4 mg, 31%).

**$^1$H NMR** (400 MHz, $CDCl_3$) δ 8.36 (d, *J* = 8.7 Hz, 2H), 8.00 (d, *J* = 8.8 Hz, 2H), 7.82 (dd, *J* = 5.0, 1.2 Hz, 1H), 7.62 (dd, *J* = 3.8, 1.2 Hz, 1H), 7.21 (dd, *J* = 5.0, 3.8 Hz, 1H).

**$^{13}$C NMR** (100 MHz, $CDCl_3$) δ 186.4, 150.0, 143.5, 142.8, 135.9, 135.6, 130.1, 128.6, 123.8.

**HRMS (-APCI)**: calcd for $C_{11}H_7O_3NS$ $[M]^-$ 233.0141, found 233.0143.

**mp:** 142-146 °C.

**naphthalen-2-yl(thiophen-2-yl)methanone**

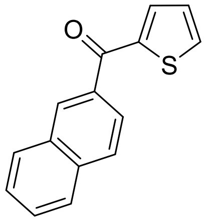

Prepared according to general procedure **C,** the titled compound was obtained as light yellow solid (33.8 mg, 71%).

Telescoped process:

An oven-dried microwave vial was sealed and placed on the Schlenk line to vacuum and refill with nitrogen three times. Then 2-(naphthalen-2-yl)pyridine (0.2 mmol, 1.0 equiv.), EtOAc (2 mL, 0.1 M) was added under a flow of nitrogen. The reaction solution was cooled to -78 $^oC$ and $Tf_2O$ (1.0 equiv.) was added dropwise. After the addition was completed, the reaction was kept -78 $^oC$ for 30 min and then a solution of dibenzylamine (1.2 equiv.) in EtOAc (1.0 M) was added slowly followed by collidine (1.0 equiv.). The reaction mixture was stirred at -78 $^oC$ for an additional 30 min and then allowed to warm to room temperature and stirred for 30 min. Then $K_2CO_3$ (2,0 equiv.) and $H_2O$ (0.2 mL) were added into the vial and the mixture was stirred at 100 $^oC$ overnight. Then EtOAc and $H_2O$ were removed under reduced pressure. $S_8$ (0.6 mmol, 3.0 equiv.) was added into the microwave vial. Dry DMF (2 mL) was added under nitrogen and the reaction mixture was stirred at 60 $^oC$ overnight. After cooling to room temperature, the reaction was diluted with EtOAc and washed with water (x3). The organic phase was dried over $Na_2SO_4$, filtered and concentrated *in vacuo*. The product was isolated as light yellow solid (25.5 mg, 54%) by using flash column chromatography.

**$^1$H NMR** (400 MHz, $CDCl_3$) δ 8.40 (s, 1H), 7.98 – 7.91 (m, 4H), 7.76 (dd, *J* = 5.0, 1.1 Hz, 1H), 7.73 (dd, *J* = 3.8, 1.1 Hz, 1H), 7.62 (ddd, *J* = 8.1, 6.8, 1.5 Hz, 1H), 7.58 (ddd, *J* = 8.1, 6.8, 1.5 Hz, 1H), 7.20 (dd, *J* = 4.9, 3.8 Hz, 1H).

**$^{13}$C NMR** (100 MHz, $CDCl_3$) δ 188.3, 143.9, 135.5, 135.3, 135.0, 134.3, 132.4, 130.7, 129.4, 128.6, 128.4, 128.1, 128.0, 127.0, 125.4.

**HRMS (+ESI)**: calcd for $C_{15}H_{10}OSNa$ $[M+Na]^+$ 261.0345, found 261.0339.

**mp:** 85-87 $^oC$.

**(9,9-dimethyl-9*H*-fluoren-2-yl)(thiophen-3-yl)methanone**

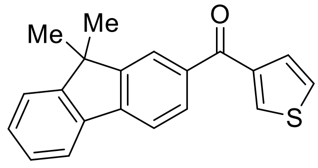

Prepared according to general procedure **C,** the titled compound was obtained as light yellow solid (43.6 mg, 72%).

Telescoped process:

An oven-dried microwave vial was sealed and placed on the Schlenk line to vacuum and refill with nitrogen three times. Then 2-(9,9-dimethyl-9H-fluoren-2-yl)pyridine (0.2 mmol, 1.0 equiv.), EtOAc (2 mL, 0.1 M) was added under a flow of nitrogen. The reaction solution was cooled to -78 $^oC$ and $Tf_2O$ (1.0 equiv.) was added dropwise. After the addition was completed, the reaction was kept -78 $^oC$ for 30 min and then a solution of dibenzylamine (1.2 equiv.) in EtOAc (1.0 M) was added slowly followed by collidine (1.0 equiv.). The reaction mixture was stirred at -78 $^oC$ for an additional 30 min and then allowed to warm to room temperature and stirred for 30 min. Then $K_2CO_3$ (2,0 equiv.) and $H_2O$ (0.2 mL) were added into the vial and the mixture was stirred at 100 $^oC$ overnight. Then EtOAc and $H_2O$ were removed under reduced pressure. $S_8$ (0.6 mmol, 3.0 equiv.) was added into the microwave vial. Dry DMF (2 mL) was added under nitrogen and the reaction mixture was stirred at 60 $^oC$ overnight. After cooling to room temperature, the reaction was diluted with EtOAc and washed with water (x3). The organic phase was dried over $Na_2SO_4$, filtered and concentrated *in vacuo*. The product was isolated as light yellow solid (31.5 mg, 52%) by using flash column chromatography.

**$^1$H NMR** (400 MHz, $CDCl_3$) δ 7.97 (d, *J* = 1.5 Hz, 1H), 7.91 (dd, *J* = 7.9, 1.6 Hz, 1H), 7.83 – 7.78 (m, 2H), 7.73 (dd, *J* = 5.0, 1.1 Hz, 1H), 7.70 (dd, *J* = 3.8, 1.1 Hz, 1H), 7.51 – 7.46 (m, 1H), 7.42 – 7.37 (m, 2H), 7.19 (dd, *J* = 5.0, 3.8 Hz, 1H), 1.54 (s, 6H).

**$^{13}$C NMR** (100 MHz, $CDCl_3$) δ 188.3, 154.8, 153.9, 144.1, 143.6, 138.1, 137.0, 134.6, 134.0, 129.1, 128.7, 128.0, 127.4, 123.9, 123.0, 121.0, 119.8, 47.2, 27.1.

**HRMS (+ESI)**: calcd for $C_{20}H_{16}OSNa$ $[M+Na]^+$ 327.0814, found 327.0810.

**mp:** 126-128 °C.

**furan-3-yl(thiophen-2-yl)methanone**

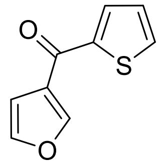

Prepared according to general procedure **C,** the titled compound was obtained as light yellow solid (24.8 mg, 70%).

**$^1$H NMR** (400 MHz, $CDCl_3$) δ 8.11 (s, 1H), 7.80 (dd, *J* = 3.8, 1.2 Hz, 1H), 7.68 (dd, *J* = 4.9, 1.2 Hz, 1H), 7.51 (t, *J* = 1.7 Hz, 1H), 7.17 (dd, *J* = 5.0, 3.8 Hz, 1H), 6.92 (dd, *J* = 1.9, 0.8 Hz, 1H).

**$^{13}$C NMR** (100 MHz, $CDCl_3$) δ 180.4, 147.2, 144.2, 144.1, 133.5, 132.6, 128.2, 126.7, 110.2.

**HRMS (+APCI)**: calcd for $C_9H_7O_2S$ $[M+H]^+$ 179.0161, found 179.0163.

**mp:** 37-39 °C.

**thiophen-2-yl(thiophen-3-yl)methanone**

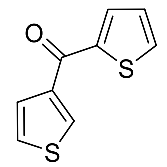

Prepared according to general procedure **C,** the titled compound was obtained as light yellow solid (29.0 mg, 75%).

Telescoped process:

An oven-dried microwave vial was sealed and placed on the Schlenk line to vacuum and refill with nitrogen three times. Then 2-(thiophen-3-yl)pyridine (0.2 mmol, 1.0 equiv.), EtOAc (2 mL, 0.1 M) was added under a flow of nitrogen. The reaction solution was cooled to -78 °C and $Tf_2O$ (1.0 equiv.) was added dropwise. After the addition was completed, the reaction was kept -78 °C for 30 min and then a solution of dibenzylamine (1.2 equiv.) in EtOAc (1.0 M) was added slowly followed by collidine (1.0 equiv.). The reaction mixture was stirred at -78 °C for an additional 30 min and then allowed to warm to room temperature and stirred for 30 min. Then $K_2CO_3$ (2,0 equiv.) and $H_2O$ (0.2 mL) were added into the vial and the mixture was stirred at 100 °C overnight. Then EtOAc and $H_2O$ were removed under reduced pressure. $S_8$ (0.6 mmol, 3.0 equiv.) was added into the microwave vial. Dry DMF (2 mL) was added under nitrogen and the reaction mixture was stirred at 60 °C overnight. After cooling to room temperature, the reaction was diluted with EtOAc and washed with water (x3). The organic phase was dried over $Na_2SO_4$, filtered and concentrated *in vacuo*. The product was isolated as light yellow solid (17.3 mg, 45%) by using flash column chromatography.

**$^1$H NMR** (400 MHz, $CDCl_3$) δ 8.09 (dd, *J* = 2.9, 1.2 Hz, 1H), 7.79 (dd, *J* = 3.8, 1.2 Hz, 1H), 7.70 (dd, *J* = 4.9, 1.2 Hz, 1H), 7.62 (dd, *J* = 5.1, 1.3 Hz, 1H), 7.40 (dd, *J* = 5.1, 2.9 Hz, 1H), 7.18 (dd, *J* = 5.0, 3.8 Hz, 1H).

**$^{13}$C NMR** (100 MHz, $CDCl_3$) δ 181.4, 144.1, 141.2, 133.8, 133.7, 132.3, 128.4, 128.1, 126.5.

**HRMS (+APCI)**: calcd for $C_9H_7OS_2$ $[M+H]^+$ 194.9933, found 194.9929.

**mp:** 60-62 °C.

**(9-phenyl-9*H*-carbazol-3-yl)(thiophen-2-yl)methanone**

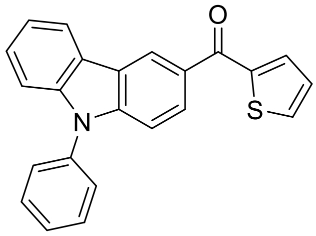

Prepared according to general procedure **C,** the titled compound was obtained as light yellow solid (50.2 mg, 71%).

**$^1$H NMR** (400 MHz, $CDCl_3$) δ 8.76 (d, *J* = 1.7 Hz, 1H), 8.20 (d, *J* = 7.7 Hz, 1H), 8.02 (dd, *J* = 8.6, 1.7 Hz, 1H), 7.74 (ddd, *J* = 11.8, 4.4, 1.2 Hz, 2H), 7.67 – 7.63 (m, 2H), 7.59 – 7.57 (m, 2H), 7.56 – 7.41 (m, 4H), 7.35 (ddd, *J* = 8.0, 6.8, 1.3 Hz, 1H), 7.21 (dd, *J* = 5.0, 3.7 Hz, 1H).

**$^{13}$C NMR** (100 MHz, $CDCl_3$) δ 188.0, 144.5, 143.5, 141.9, 137.1, 134.3, 133.4, 130.2, 128.3, 128.0, 127.9, 127.3, 126.9, 123.5, 123.2, 123.0, 121.0, 120.8, 110.4, 109.7.

**HRMS (+ESI)**: calcd for $C_{23}H_{15}ONSNa$ $[M+Na]^+$ 376.0767, found 376.0762.

**mp:** 128-132 °C.

**dibenzo[b,d]furan-2-yl(thiophen-2-yl)methanone**

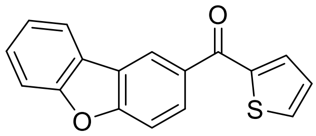

Prepared according to general procedure **C,** the titled compound was obtained as light yellow solid (40.0 mg, 72%).

**$^1$H NMR** (400 MHz, $CDCl_3$) δ 8.52 (d, *J* = 1.8 Hz, 1H), 8.04 (dd, *J* = 8.5, 1.8 Hz, 1H), 8.01 (d, *J* = 7.1 Hz, 1H), 7.75 (dd, *J* = 5.0, 1.2 Hz, 1H), 7.72 (dd, *J* = 3.8, 1.1 Hz, 1H), 7.66 (d, *J* = 8.6 Hz, 1H), 7.62 (d, *J* = 8.2 Hz, 1H), 7.52 (td, *J* = 7.8, 1.3 Hz, 1H), 7.40 (td, *J* = 7.5, 1.0 Hz, 1H), 7.21 (dd, *J* = 5.0, 3.8 Hz, 1H).

**$^{13}$C NMR** (100 MHz, $CDCl_3$) δ 187.8, 158.7, 157.0, 144.0, 134.8, 134.2, 133.3, 129.0, 128.2, 128.1, 124.6, 123.8, 123.5, 122.7, 121.2, 112.1, 111.8.

**HRMS (+ESI)**: calcd for $C_{17}H_{10}O_2SNa$ $[M+Na]^+$ 301.0294, found 301.0289.

**mp:** 98-102 °C.

**dibenzo[b,d]thiophen-2-yl(thiophen-2-yl)methanone**

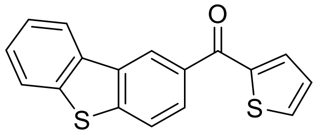

Prepared according to general procedure **C,** the titled compound was obtained as light yellow solid (47.6 mg, 81%).

**$^1$H NMR** (400 MHz, $CDCl_3$) δ 8.67 (dd, *J* = 1.6, 0.8 Hz, 1H), 8.23 – 8.19 (m, 1H), 8.00 – 7.94 (m, 2H), 7.91 – 7.87 (m, 1H), 7.76 (dd, *J* = 5.0, 1.1 Hz, 1H), 7.73 (dd, *J* = 3.8, 1.1 Hz, 1H), 7.54 – 7.48 (m, 2H), 7.21 (dd, *J* = 5.0, 3.8 Hz, 1H).

**$^{13}$C NMR** (100 MHz, $CDCl_3$) δ 188.0, 144.0, 143.9, 139.9, 135.6, 135.2, 134.9, 134.6, 134.3, 128.2, 127.6, 127.4, 125.0, 123.1, 122.9, 122.8, 122.1.

**HRMS (+ESI)**: calcd for $C_{17}H_{10}OS_2Na$ $[M+Na]^+$ 317.0065, found 317.0062.

**mp:** 62-68 °C.

**(2-methylbenzofuro[2,3-b]pyridin-8-yl)(thiophen-2-yl)methanone**

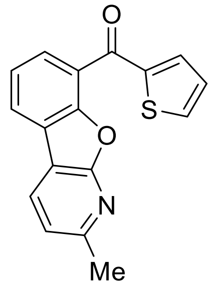

Prepared according to general procedure **C,** the titled compound was obtained as light yellow solid (39.2 mg, 67%).

**$^1$H NMR** (400 MHz, $CDCl_3$) δ 8.19 (d, *J* = 7.8 Hz, 1H), 8.07 (dd, *J* = 7.7, 1.3 Hz, 1H), 7.79 – 7.75 (m, 2H), 7.63 (dd, *J* = 3.8, 1.2 Hz, 1H), 7.48 (t, *J* = 7.7 Hz, 1H), 7.24 (d, *J* = 7.8 Hz, 1H), 7.13 (dd, *J* = 4.9, 3.8 Hz, 1H), 2.67 (s, 3H).

**$^{13}$C NMR** (100 MHz, $CDCl_3$) δ 185.2, 163.2, 157.5, 151.2, 144.2, 135.8, 135.2, 130.2, 128.4, 128.3, 124.3, 124.0, 124.0, 123.4, 119.4, 113.2, 24.7.

**HRMS (+ESI)**: calcd for $C_{17}H_{11}NO_2SNa$ $[M+Na]^+$ 316.0403, found 316.0397.

**mp:** 120-122 °C.

**2-chloro-N-(4-chloro-3-(thiophene-2-carbonyl)phenyl)-4-(methylsulfonyl)benzamide**

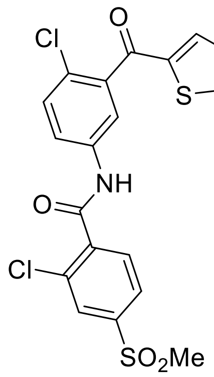

To an oven-dried microwave vial were added Zincke ketone (0.1 mmol, 1.0 equiv.) and $S_8$ (0.3 mmol, 9.6 mg, 3.0 equiv.) and DMSO (1 mL) and the reaction mixture was stirred at 60 °C overnight. After cooling to room temperature, the reaction was diluted with EtOAc and washed with water (x3). The organic phase was dried over $Na_2SO_4$, filtered and concentrated *in vacuo*. The product was isolated as light yellow solid (6.8 mg, 15%) by using flash column chromatography.

**$^1$H NMR** (400 MHz, $CDCl_3$) δ 8.36 (s, 1H), 7.96 (s, 1H), 7.85 (s, 2H), 7.80 – 7.76 (m, 3H), 7.51 – 7.47 (m, 2H), 7.15 (dd, *J* = 4.9, 3.8 Hz, 1H), 3.07 (s, 3H).

**$^{13}$C NMR** (100 MHz, $CDCl_3$) δ 163.3, 143.4, 139.0, 136.5, 136.2, 136.0, 132.4, 131.2, 131.2, 129.4, 128.7, 127.2, 126.2, 123.0, 120.6, 44.6.

**HRMS (+APCI)**: calcd for $C_{19}H_{14}O_4NCl_2S_2$ $[M+H]^+$ 453.9736, found 453.9733.

**mp:** 85-87 °C.

**phenyl(3-phenylthiophen-2-yl)methanone**

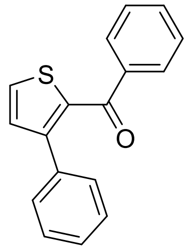

Prepared according to general procedure **C,** the titled compound was obtained as light yellow gum (34.4 mg, 65%).

**$^1$H NMR** (400 MHz, $CDCl_3$) δ 7.64 – 7.60 (m, 3H), 7.35 – 7.31 (m, 1H), 7.24 – 7.12 (m, 8H)

**$^{13}$C NMR** (100 MHz, $CDCl_3$) δ 190.4, 146.9, 137.7, 137.3, 135.8, 132.4, 130.7, 130.7, 129.9, 129.4, 128.2, 127.9, 127.7.

**HRMS (+ESI)**: calcd for $C_{17}H_{13}OS$ $[M+H]^+$ 265.0691, found 265.0690.

**(3-(4-methoxyphenyl)thiophen-2-yl)(phenyl)methanone**

Prepared according to general procedure **C,** the titled compound was obtained as light yellow gum (26.4 mg, 45%).

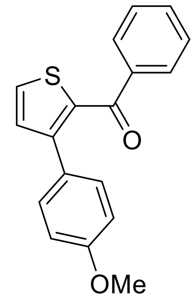

**$^1$H NMR** (400 MHz, $CDCl_3$) δ 7.64 – 7.60 (m, 3H), 7.37 – 7.33 (m, 1H), 7.22 – 7.13 (m, 5H), 6.70 – 6.66 (m, 2H), 3.73 (s, 3H).

**$^{13}$C NMR** (100 MHz, $CDCl_3$) δ 190.5, 159.3, 146.6, 137.8, 136.4, 132.4, 130.7, 130.6, 130.6, 129.9, 128.3, 128.0, 113.7, 55.4.

**HRMS (+ESI)**: calcd for $C_{18}H_{15}O_2S$ $[M+H]^+$ 295.0801, found 295.0802.

**phenyl(3-(m-tolyl)thiophen-2-yl)methanone**

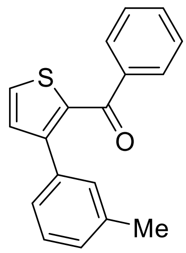

Prepared according to general procedure **C,** the titled compound was obtained as light yellow oil (36.6 mg, 66%).

**$^1$H NMR** (400 MHz, $CDCl_3$) δ 7.63 – 7.59 (m, 3H), 7.34 – 7.30 (m, 1H), 7.22 (d, *J* = 5.0 Hz, 1H), 7.17 (t, *J* = 7.8 Hz, 2H), 7.04 (dd, *J* = 3.8, 1.6 Hz, 2H), 6.97 (s, 1H), 6.95 – 6.92 (m, 1H), 2.16 (s, 3H).

**$^{13}$C NMR** (100 MHz, $CDCl_3$) δ 190.5, 147.1, 137.8, 137.8, 137.3, 135.6, 132.3, 130.7, 130.7, 130.5, 129.7, 128.4, 128.1, 127.9, 126.3, 21.2.

**HRMS (+APCI)**: calcd for $C_{18}H_{15}OS$ $[M+H]^+$ 279.0838, found 279.0830.

**(3-([1,1'-biphenyl]-4-yl)thiophen-2-yl)(phenyl)methanone**

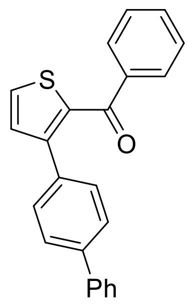

Prepared according to general procedure **C,** the titled compound was obtained as light yellow solid (36.6 mg, 54%).

**$^1$H NMR** (400 MHz, $CDCl_3$) δ 7.66 – 7.64 (m, 3H), 7.51 – 7.48 (m, 2H), 7.44 – 7.28 (m, 9H), 7.19 (t, *J* = 7.7 Hz, 2H).

**$^{13}$C NMR** (100 MHz, $CDCl_3$) δ 190.4, 146.6, 140.7, 140.5, 137.8, 137.2, 134.7, 132.4, 130.8, 130.7, 129.9, 129.8, 128.9, 128.0, 127.6, 127.1, 126.9.

**HRMS (+ESI)**: calcd for $C_{23}H_{17}OS$ $[M+H]^+$ 341.1005, found 341.1006.

**mp:** 88-90 °C.

**2-(1-phenylvinyl)thiophene**

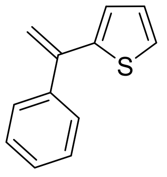

To a suspension of $MePPh_3Br$ (1.5 equiv.) in THF (2 mL) at 0 °C was added $^n$BuLi (1.5 equiv.) dropwise and then the reaction was stirred at room temperature for 30 min. Phenyl(thiophen-2-yl)methanone (0.2 mmol, 1.0 equiv.) was added. The reaction mixture was stirred at 50 °C for 2 h. After cooling to room temperature, the reaction was quenched with sat. aq. $NH_4Cl$, extracted with EtOAc (x2). The organic layers were combined, dried over $Na_2SO_4$, filtered and concentrated under reduced pressure. The desired compound was purified through flash column chromatography and obtained as colourless oil (32.0 mg, 86%).

**$^1$H NMR** (400 MHz, $CDCl_3$) δ 7.47 – 7.43 (m, 2H), 7.40 – 7.36 (m, 3H), 7.24 (dd, *J* = 5.1, 1.2 Hz, 1H), 6.98 (dd, *J* = 5.1, 3.6 Hz, 1H), 6.92 (dd, *J* = 3.5, 1.2 Hz, 1H), 5.59 (s, 1H), 5.25 (s, 1H).

**$^{13}$C NMR** (100 MHz, $CDCl_3$) δ 144.9, 143.5, 141.2, 128.5, 128.3, 128.2, 127.4, 126.6, 125.2, 113.8.

**HRMS (+APCI)**: calcd for $C_{12}H_{11}S$ $[M+H]^+$ 187.0576, found 187.0577.

**phenyl(thiophen-2-yl)methanethione**

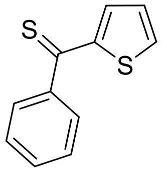


To a solution of phenyl(thiophen-2-yl)methanone (0.2 mmol, 1.0 equiv.) in toluene (2 mL) was added Lawesson's reagent (0.6 equiv.). The resulting mixture was refluxed until complete consumption of the ketone and then cooled to room temperature. The solvent was removed under reduced pressure and the residue was purified by column chromatography. The final product was generated as green oil (40.6 mg, >99%).

**$^1$H NMR** (400 MHz, $CDCl_3$) δ 7.78 (d, *J* = 5.0 Hz, 1H), 7.69 (d, *J* = 6.9 Hz, 2H), 7.53 (t, *J* = 7.4 Hz, 1H), 7.42 – 7.38 (m, 3H), 7.17 – 7.14 (m, 1H).

**$^{13}$C NMR** (100 MHz, $CDCl_3$) δ 223.9, 155.3, 147.4, 138.5, 131.7, 131.3, 129.1, 128.8, 128.1.

**HRMS (+APCI)**: calcd for $C_{11}H_9S_2$ $[M+H]^+$ 205.0140, found 205.0137.

**2-benzylthiophene**

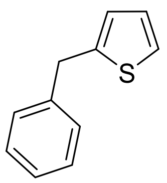


A mixture of phenyl(thiophen-2-yl)methanone (0.2 mmol, 1.0 equiv.), $NaBH_4$ (5.0 equiv.) and anhydrous $AlCl_3$ (3.0 equiv.) was added into an oven-dried microwave vial and then THF was injected under $N_2$ atmosphere. The mixture was stirred under reflux until the starting material was fully converted. The reaction was cooled to room temperature, quenched with slow addition of water, extracted with EtOAc. The organic phases were dried over $Na_2SO_4$, filtered and concentrated *in vacuo*. The titled compound was given as colourless oil (31.3 mg, 90%) after purification through flash column chromatography.

**$^1$H NMR** (400 MHz, $CDCl_3$) δ 7.31 (dd, *J* = 8.2, 6.8 Hz, 2H), 7.25 (d, *J* = 7.4 Hz, 4H), 7.15 (dd, *J* = 5.1, 1.2 Hz, 1H), 6.93 (dd, *J* = 5.2, 3.4 Hz, 1H), 6.81 – 6.80 (m, 1H), 4.16 (s, 2H).

**$^{13}$C NMR** (100 MHz, $CDCl_3$) δ 144.2, 140.5, 128.73, 128.68, 127.0, 126.6, 125.3, 124.1, 36.2.

The data are consistent with those already published.[14]

**(5-(2,4-dimethoxyphenyl)thiophen-2-yl)(phenyl)methanone**

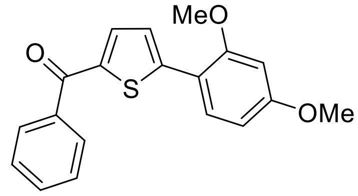


In an oven-dried microwave vial, phenyl(thiophen-2-yl)methanone (0.2 mmol, 1.0 equiv.), 2,6-dimethoxybenzoic acid (1.2 equiv.), $Pd(OAc)_2$ (5 mol%), $PCy_3$ (10 mol%) and $Ag_2CO_3$ (2.0 equiv.) were added. The vial was sealed, followed by three cycles of vacuum/$N_2$ backfill. Then DMSO (0.3 mL) and DME (1.7 mL) was introduced. The reaction was stirred at 120 °C for 24 h and cooled to room temperature. The mixture was diluted with EtOAc and washed with brine (x3), dried over $Na_2SO_4$ and concentrated under reduced pressure. The product was purified by flash column chromatography and observed as light yellow solid (42.1 mg, 65%).

**$^1$H NMR** (400 MHz, $CDCl_3$) δ 7.91 – 7.88z (m, 2H), 7.61 (d, *J* = 4.0 Hz, 1H), 7.60 – 7.55 (m, 2H), 7.49 (t, *J* = 7.3 Hz, 2H), 7.30 (t, *J* = 8.4 Hz, 1H), 6.67 (d, *J* = 8.4 Hz, 2H), 3.88 (s, 6H).

**$^{13}$C NMR** (100 MHz, $CDCl_3$) δ 188.7, 158.0, 144.2, 142.1, 138.8, 134.5, 131.9, 130.1, 130.0, 129.3, 128.4, 111.6, 104.4, 56.1.

**HRMS (+APCI)**: calcd for $C_{19}H_{17}O_3S$ $[M+H]^+$ 325.0893, found 325.0891.

**mp:** 84-86 °C.

**thiophen-2-yl(4-vinylphenyl)methanone**

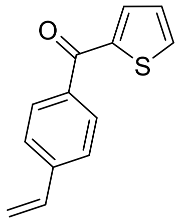

An oven-dried microwave vial was sealed and placed on the Schlenk line to vacuum and refill with nitrogen three times. Then 2-(4-vinylphenyl)pyridine (0.2 mmol, 1.0 equiv.), EtOAc (2 mL, 0.1 M) was added under a flow of nitrogen. The reaction solution was cooled to -78 °C and $Tf_2O$ (1.0 equiv.) was added dropwise. After the addition was completed, the reaction was kept -78 °C for 30 min and then a solution of dibenzylamine (1.2 equiv.) in EtOAc (1.0 M) was added slowly followed by collidine (1.0 equiv.). The reaction mixture was stirred at -78 °C for an additional 30 min and then allowed to warm to room temperature and stirred for 30 min. Then $K_2CO_3$ (2,0 equiv.) and $H_2O$ (0.2 mL) were added into the vial and the mixture was stirred at 100 °C overnight. Then EtOAc and $H_2O$ were removed under reduced pressure. $S_8$ (0.6 mmol, 3.0 equiv.) was added into the microwave vial. Dry DMF (2 mL) was added under nitrogen and the reaction mixture was stirred at 60 °C overnight. After cooling to room temperature, the reaction was diluted with EtOAc and washed with water (x3). The organic phase was dried over $Na_2SO_4$, filtered and concentrated *in vacuo*. The product was isolated as light yellow solid (26.5 mg, 62%) by using flash column chromatography.

**$^1$H NMR** (400 MHz, $CDCl_3$) δ 7.85 (d, *J* = 8.4 Hz, 2H), 7.72 (dd, *J* = 4.9, 1.2 Hz, 1H), 7.66 (dd, *J* = 3.8, 1.2 Hz, 1H), 7.52 (d, *J* = 8.4 Hz, 2H), 7.17 (dd, *J* = 4.9, 3.7 Hz, 1H), 6.79 (dd, *J* = 17.6, 10.9 Hz, 1H), 5.89 (d, *J* = 17.6 Hz, 1H), 5.41 (d, *J* = 10.9 Hz, 1H).

**$^{13}$C NMR** (100 MHz, $CDCl_3$) δ 187.8, 143.8, 141.6, 137.4, 136.1, 134.7, 134.2, 129.8, 128.1, 126.3, 116.7.

The data are consistent with those already published.[16]

## 4. Mechanism

We proposed the plausible mechanism of the reaction depicted in **Figure S1.** The initial step is proposed to involve a nucleophilic attack by C2 of Zincke ketone **1a** on the cyclic $S_8$ monomer, leading to ring opening and formation of intermediate **A**. Intermediate **A** can then undergo a conformational change *via* double bond rotation to give intermediate **B** (**Pathway 1**). An intramolecular nucleophilic attack on the iminium ion subsequently forms a C–S bond, accompanied by the elimination of $S_7$, to generate the 2,5-dihydrothiophene intermediate **C**. Elimination of a molecule of amine from intermediate **C** then furnishes the final thiophene product **2a**. An alternative mechanism (**Pathway 2**) involves the intramolecular nucleophilic attack of intermediate **A** at C3 of Zincke ketone, resulting in the formation of an episulfide intermediate **D**. Subsequent ring opening of the three-membered episulfide moiety affords enolate intermediate **E**, which can then act as a nucleophile and attack the iminium ion to form a new C–C bond, producing the 2,3-dihydrothiophene intermediate **F**. Elimination of the amine from **F** would then give the thiophene product **2a**. However, based on the experimental results obtained from the disubstituted thiophene products, pathway 2 can be ruled out. If this pathway were operative, compound **2'** would be expected from the annulation of substrate **3** with sulfur, which is contrary to our experimental observations.

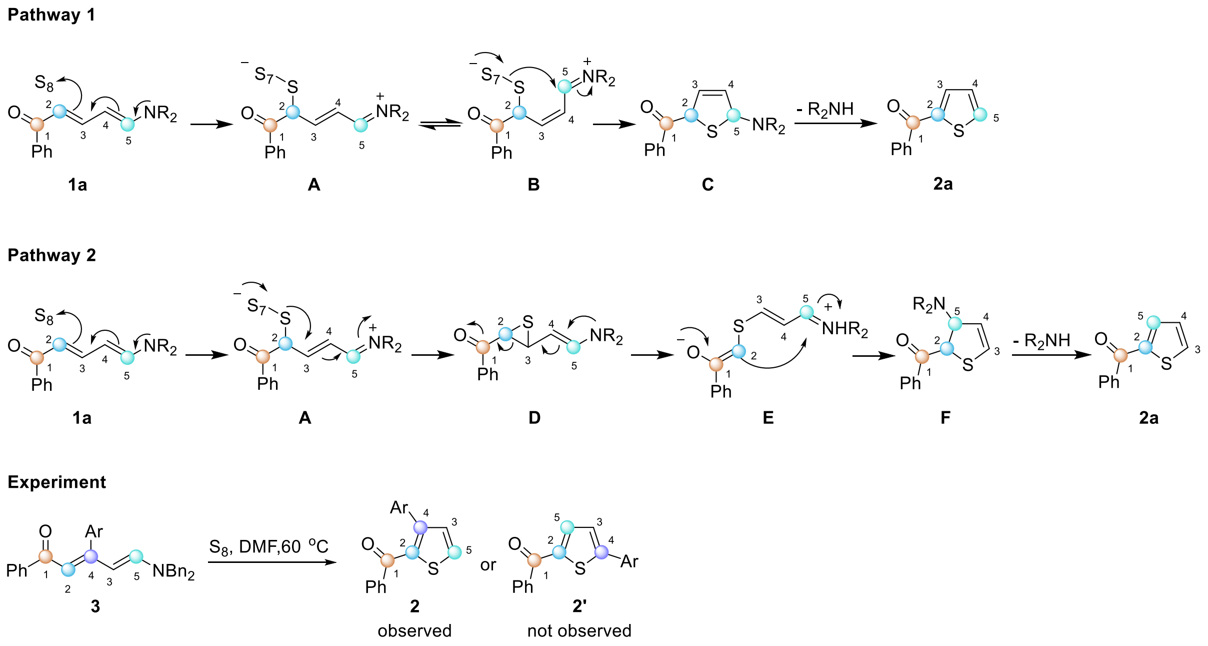


**Figure S1.** Proposed mechanism.

## 5. References


1) H. Wang, M. F. Greaney, *Angew. Chem. Int. Ed.* **2024**, *63*, e202315418.
2) X. Wang, X. Ji, C. Shao, Y. Zhang, Y. Zhang, *Org. Biomol. Chem.* **2017**, *15*, 5616.
3) F. Pan, Z.-Q. Lei, H. Wang, H. Li, J. Sun, Z.-J. Shi, *Angew. Chem. Int. Ed.* **2013**, *52*, 2063.
4) B. M. Coleridge, C. S. Bello, D. H. Ellenberger, A. Leitner, Tetrahedron Lett. **2010**, *51*, 357.
5) A. Tsuboyama, H. Iwawaki, M. Furugori, T. Mukaide, J. Kamatani, S. Igawa, T. Moriyama, S. Miura, T. Takiguchi, S. Okada, M. Hoshino, K. Ueno, *J. Am. Chem. Soc.* **2003**, *125*, 12971.
6) C. A. Fleckenstein, H. Plenio, *J. Org. Chem.* **2008**, *73*, 3236.
7) J.-S. Ouyang, Y.-F. Li, F.-D. Huang, D.-D. Lu, F.-S. Liu, *ChemCatChem* **2018**, *10*, 371.
8) Y. Ma, D. Zhang, W. Lv, Q. Zhao, W.-Y. Wong, *J. Organometallic Chem.* **2023**, *992*, 122697.
9) W.-X. Li, B.-J. Huo, J.-Y. Huang, W. Rao, H. Xu, X. Zhou, Z.-L. Shen, *J. Catal.* **2024**, *430*, 115359.
10) M. Tavasli, S. Bettington, I. F. Perepichka, A. S. Batsanov, M. R. Bryce, C. Rothe, A. P. Monkman, *Eur. J. Inorg. Chem.* **2007**, *30*, 4808.
11) M. V. Rojo, L. Guetzoyan, I. R. Baxendale, *Org. Bio. Chem.* **2015**, *13*, 1768.

12) P. Gautam, R. Gupta, B. M. Bhanage, *Eur. J. Org. Chem.* **2017**, 3431.
13) P. Ortiz, A. M. d. Hoyo, S. R. Harutyunyan, *Eur. J. Org. Chem*. **2015**, 72.
14) J. Zhou, Z. Zhao, B. Jiang, K. Yamamoto, Y. Sumii, N. Shibata, *Chem. Sci.* **2023**, *14*, 4248.
15) P. Hu, M. Zhang, X. Jie, W. Su, *Angew. Chem. Int. Ed.* **2012**, *51*, 227.
16) H. Li, M. Yang, Y. Qi, J. Xue, *Eur. J. Org. Chem.* **2011**, *14*, 2662.

## 6. NMR spectra

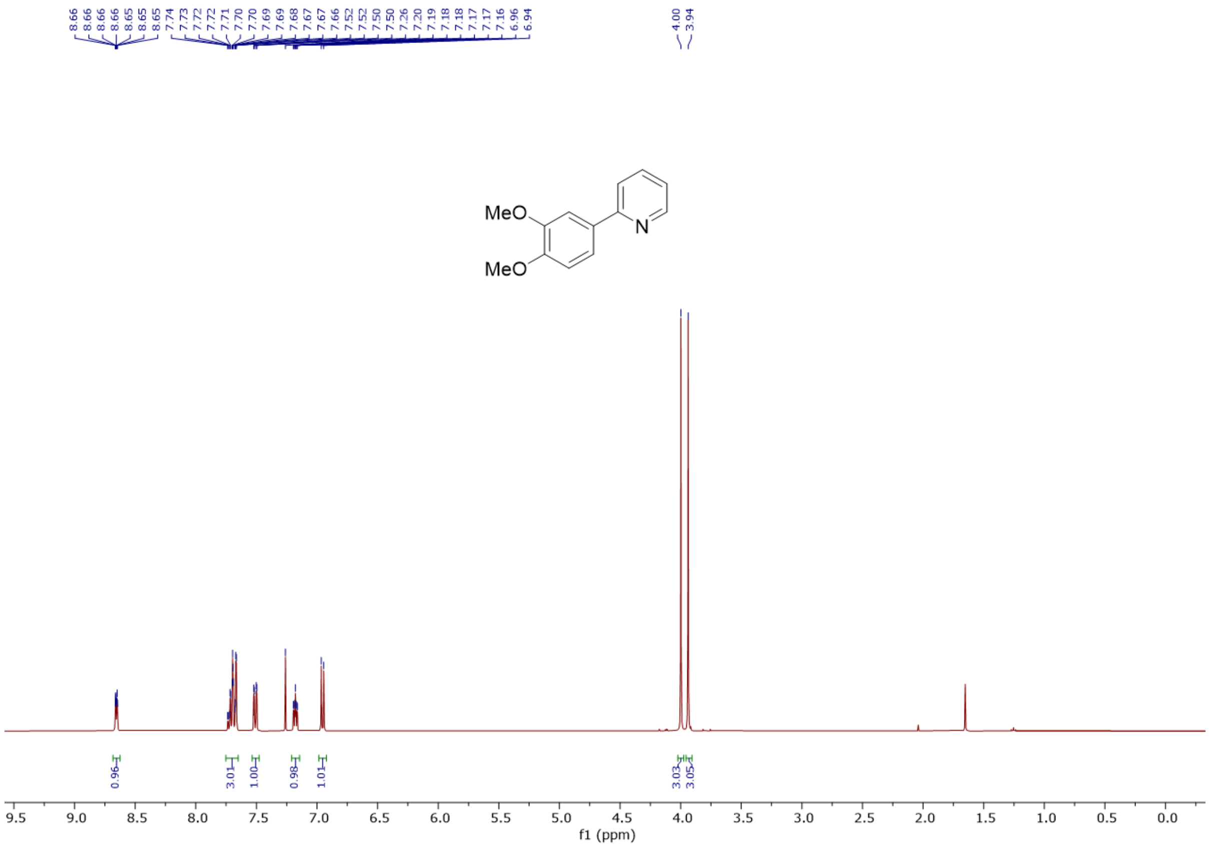


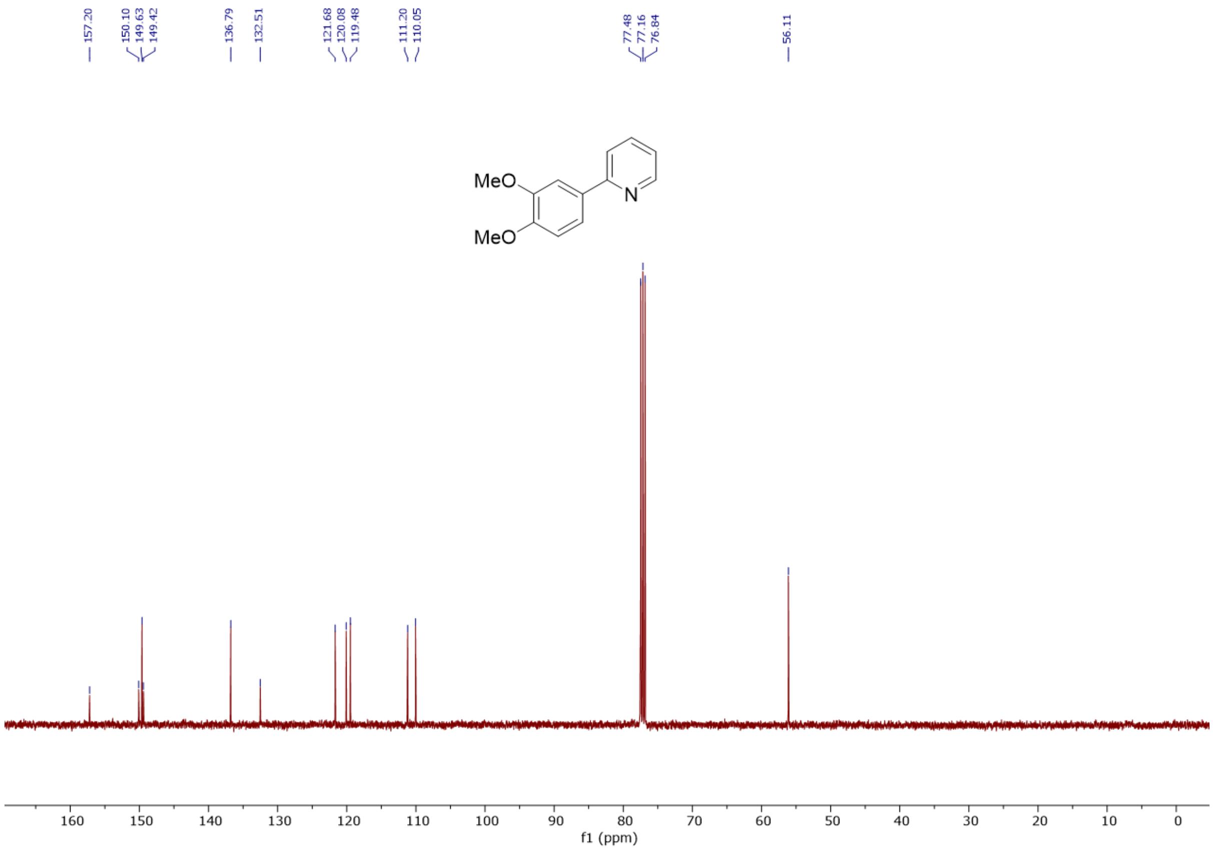

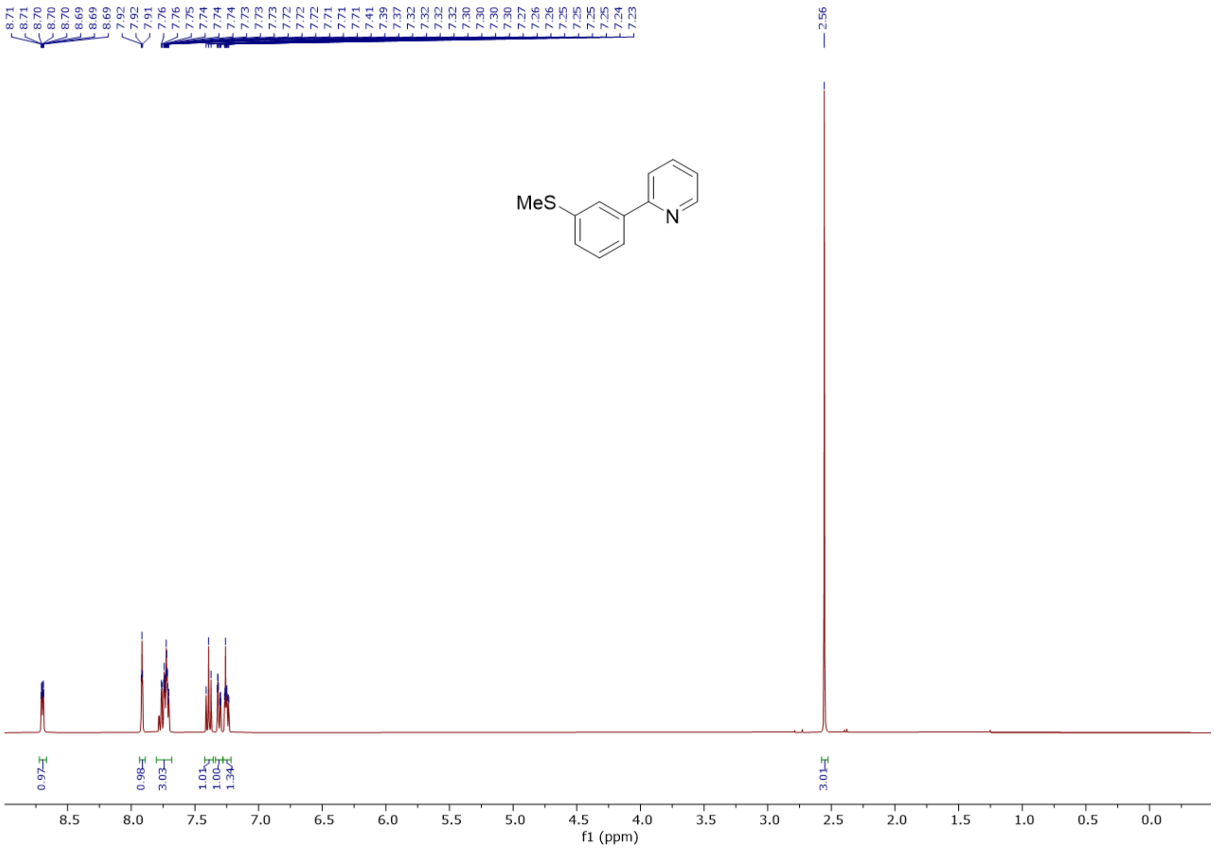

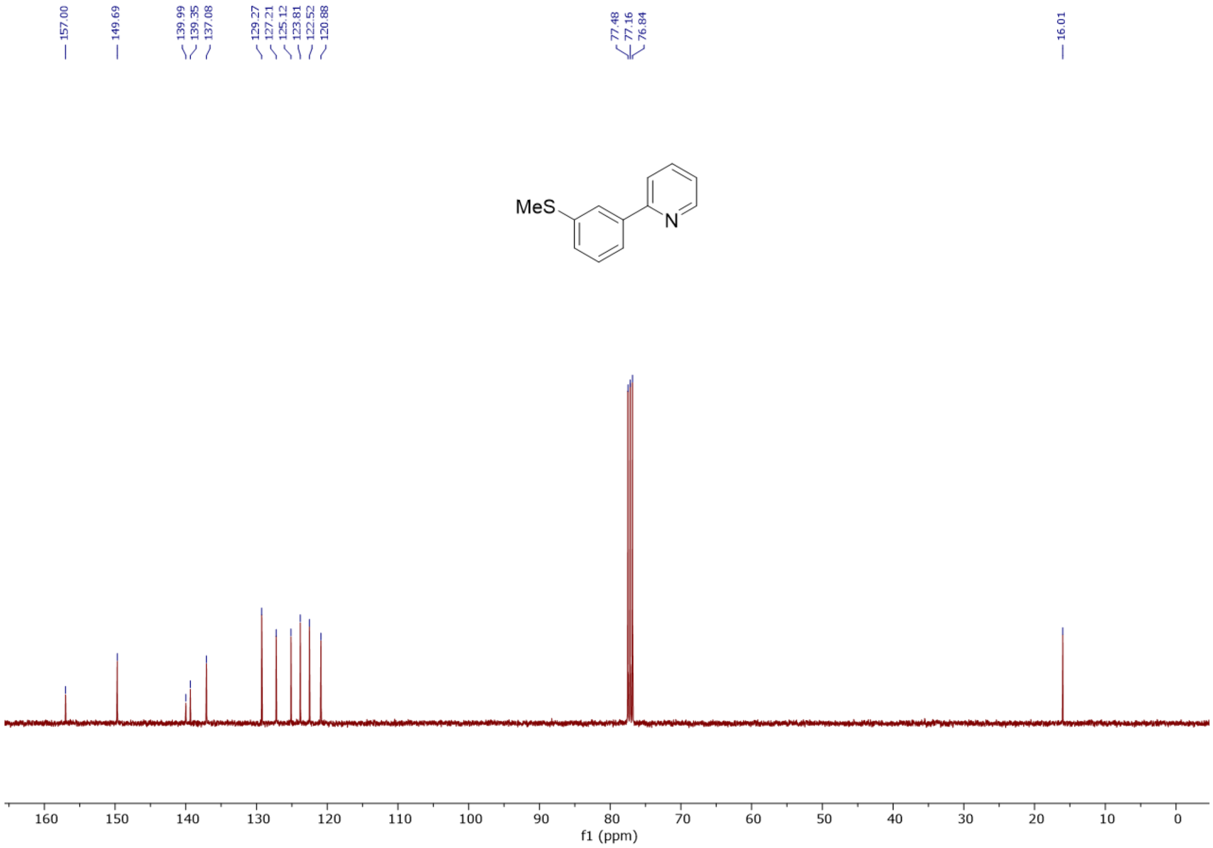

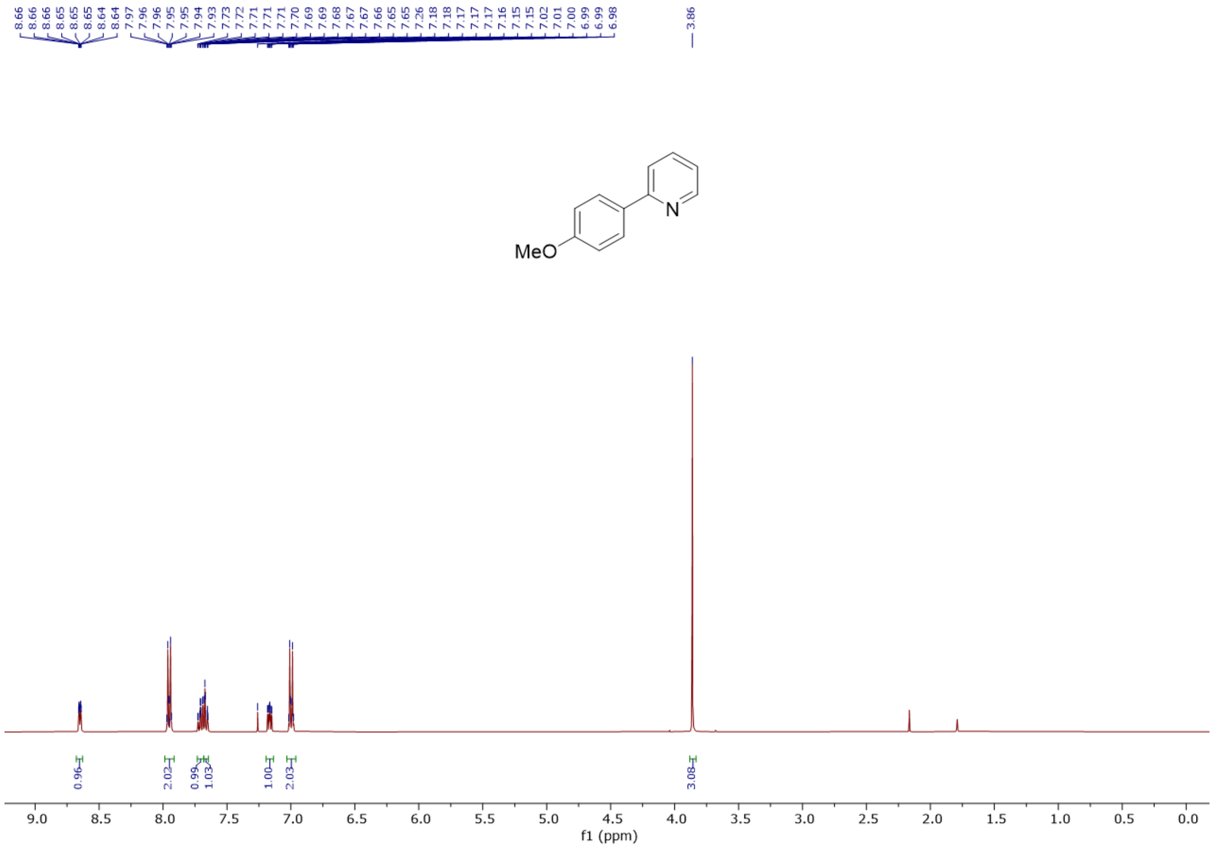
8.66 8.66 8.66 8.65 8.65 8.65 8.64 8.64 7.97 7.96 7.96 7.95 7.95 7.94 7.93 7.73 7.72 7.71 7.71 7.71 7.70 7.69 7.69 7.68 7.67 7.67 7.66 7.65 7.65 7.26 7.18 7.18 7.17 7.17 7.17 7.16 7.15 7.15 7.02 7.01 7.00 6.99 6.99 6.98
3.86
N
MeO
0.96
2.02
0.99
1.03
1.00
2.03
3.08
f1 (ppm)

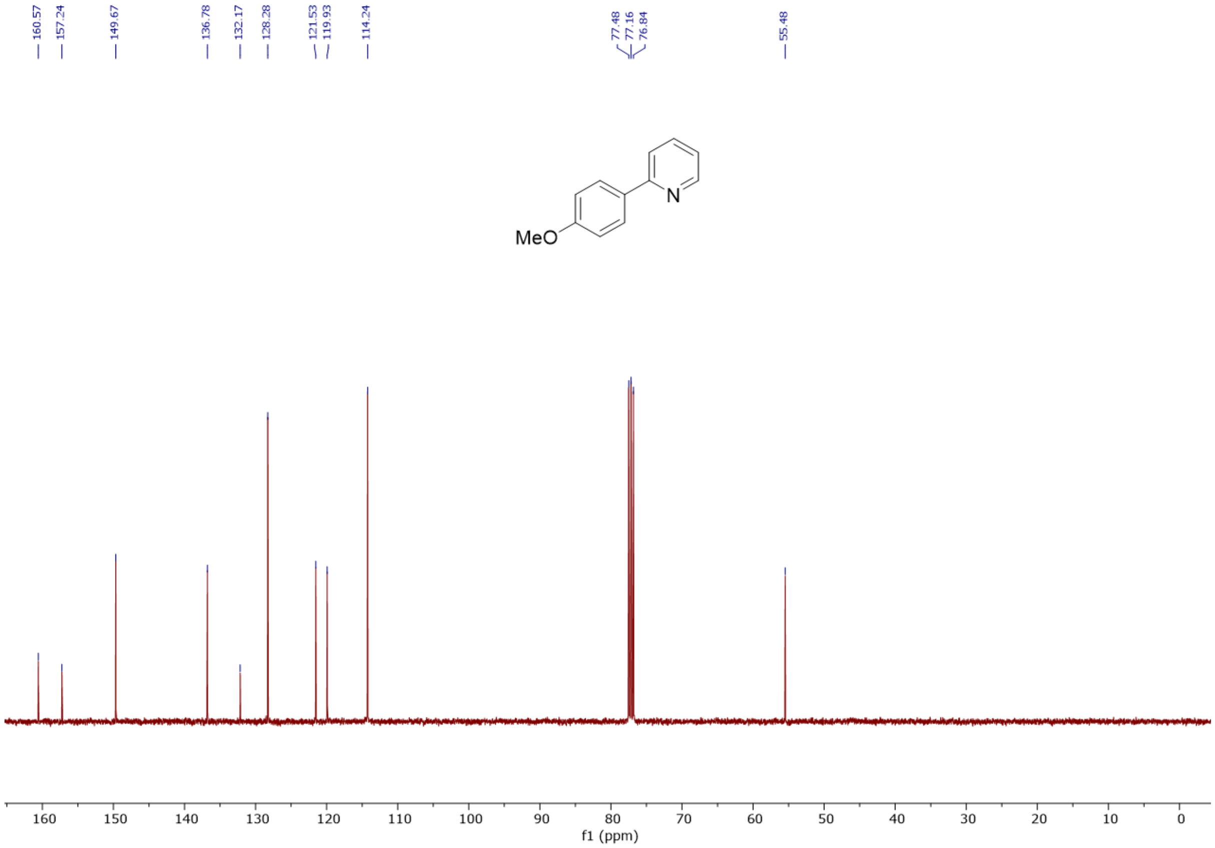
160.57
157.24
149.67
136.78
132.17
128.28
121.53
119.93
114.24
77.48
77.16
76.84
55.48
N
MeO
f1 (ppm)

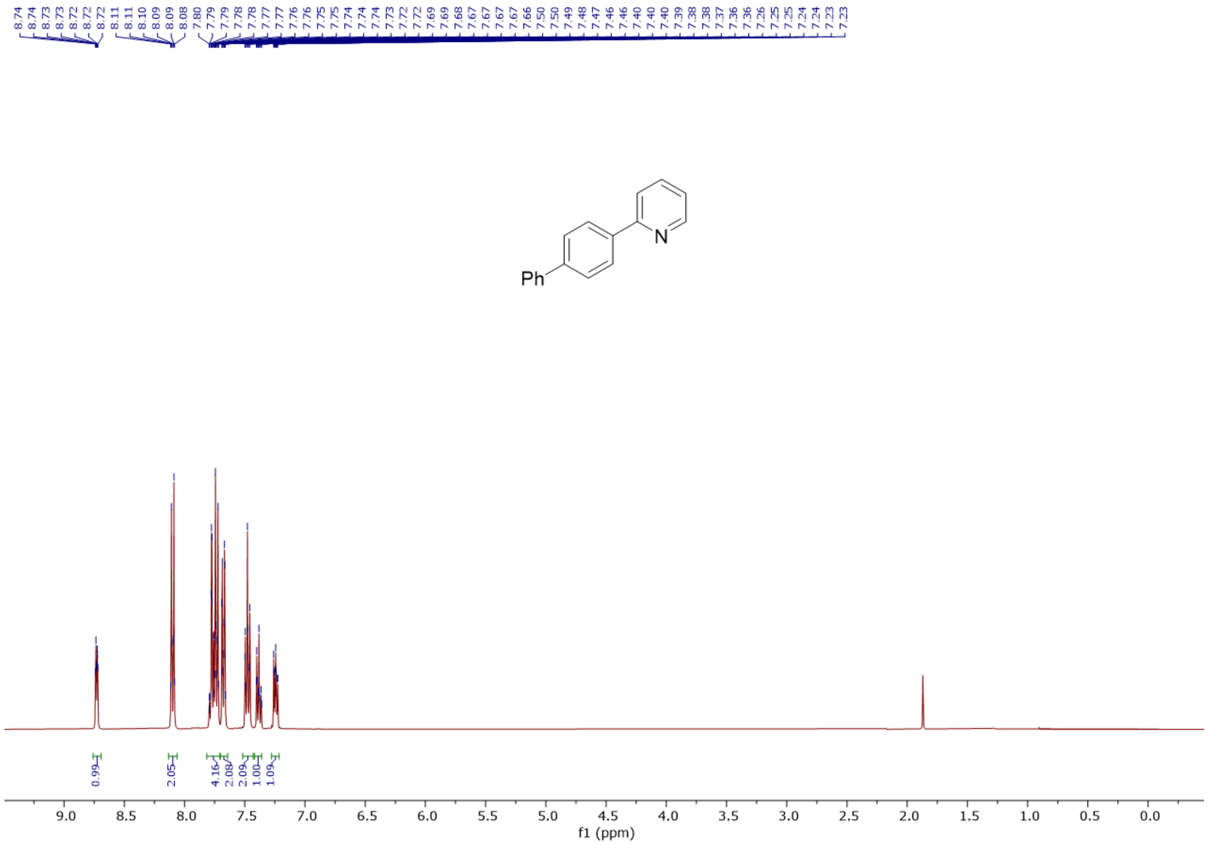
8.74 8.74 8.73 8.73 8.72 8.72 8.72 8.11 8.11 8.10 8.09 8.09 8.08 7.80 7.79 7.79 7.78 7.78 7.77 7.77 7.76 7.76 7.75 7.75 7.74 7.74 7.74 7.73 7.72 7.72 7.69 7.69 7.68 7.67 7.67 7.67 7.67 7.66 7.50 7.50 7.49 7.48 7.47 7.46 7.46 7.40 7.40 7.40 7.39 7.38 7.38 7.37 7.36 7.36 7.26 7.25 7.25 7.24 7.24 7.23 7.23
Ph
N
0.99
2.05
4.16
2.08
2.09
1.00
1.09
9.0 8.5 8.0 7.5 7.0 6.5 6.0 5.5 5.0 4.5 4.0 3.5 3.0 2.5 2.0 1.5 1.0 0.5 0.0
f1 (ppm)

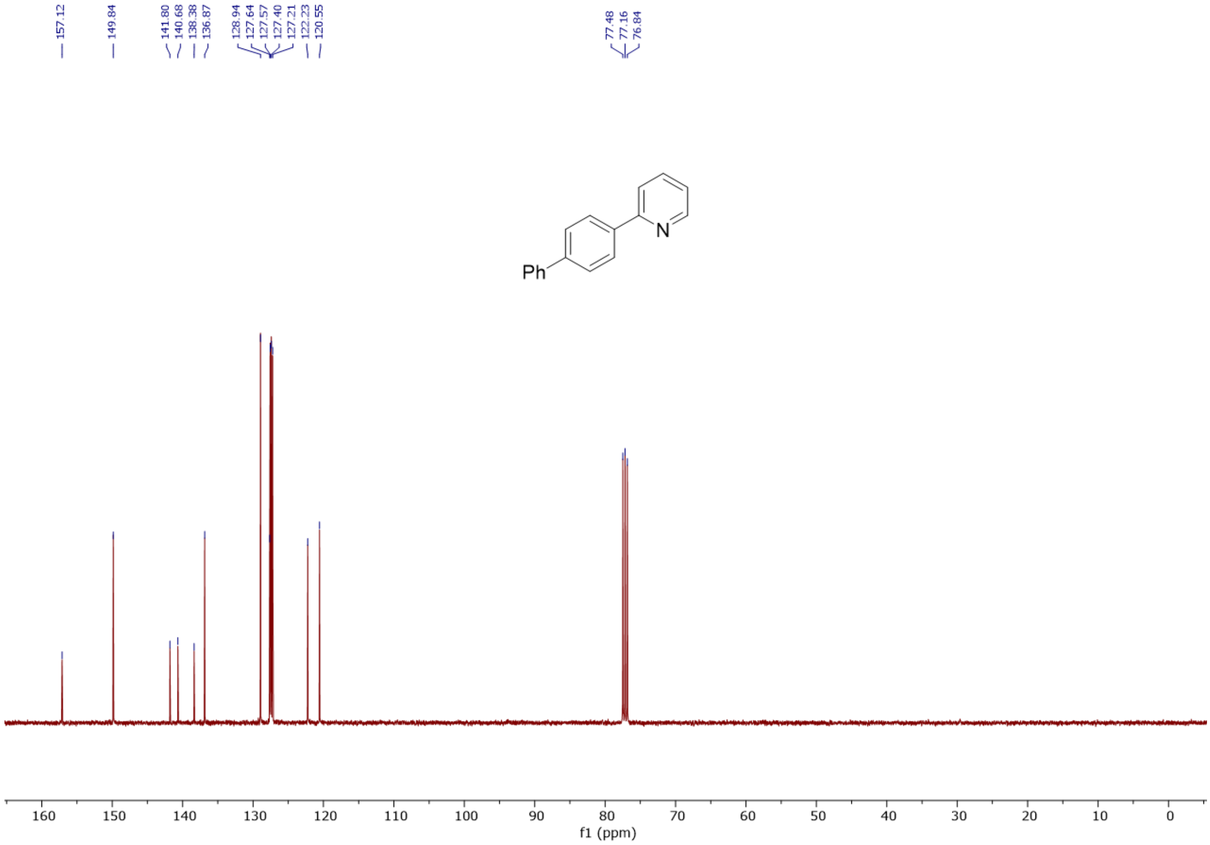
157.12
149.84
141.80 140.68 138.38 136.87
128.94 127.64 127.57 127.40 127.21 122.23 120.55
77.48 77.16 76.84
Ph
N
160 150 140 130 120 110 100 90 80 70 60 50 40 30 20 10 0
f1 (ppm)

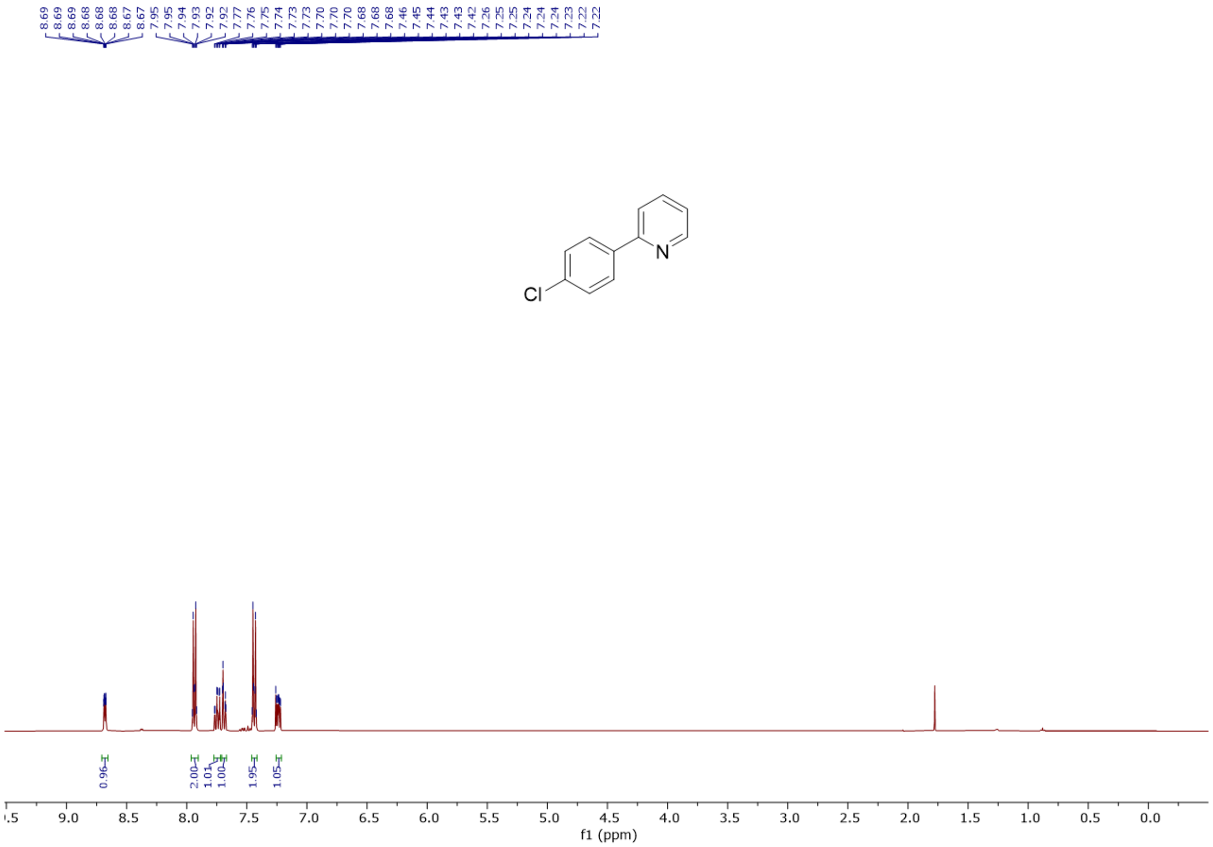
8.69 8.69 8.69 8.68 8.68 8.68 8.67 7.95 7.95 7.94 7.93 7.92 7.92 7.77 7.76 7.75 7.74 7.73 7.73 7.70 7.70 7.70 7.68 7.68 7.68 7.46 7.45 7.44 7.43 7.43 7.42 7.26 7.25 7.25 7.24 7.24 7.24 7.23 7.22 7.22
Cl
N
0.96
2.00
1.01
1.00
1.95
1.05
f1 (ppm)

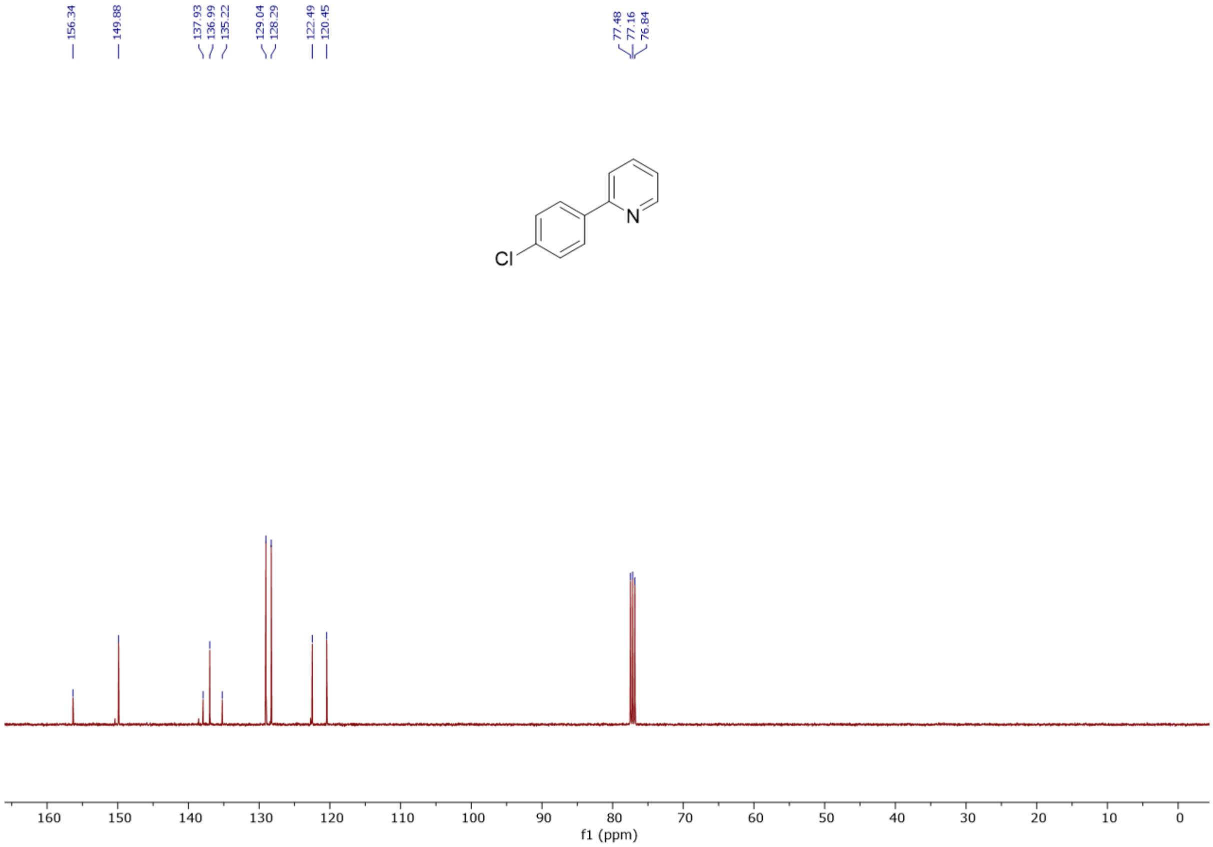
156.34
149.88
137.93 136.99 135.22
129.04 128.29
122.49 120.45
77.48 77.16 76.84
Cl
N
f1 (ppm)

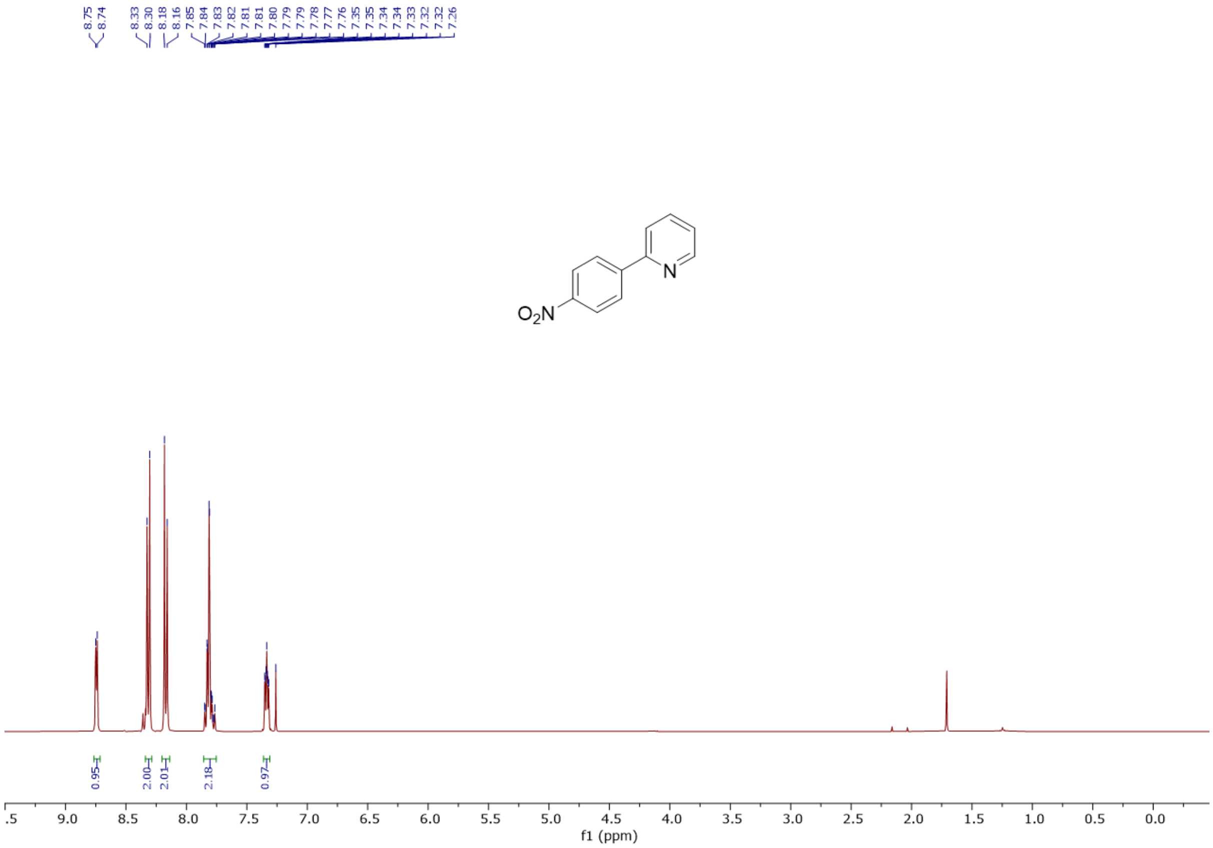
8.75
8.74
8.33
8.30
8.18
8.16
7.85
7.84
7.83
7.82
7.81
7.81
7.80
7.79
7.79
7.78
7.77
7.76
7.35
7.35
7.34
7.34
7.33
7.32
7.32
7.26
O2N
N
0.95
2.00
2.01
2.18
0.97
f1 (ppm)

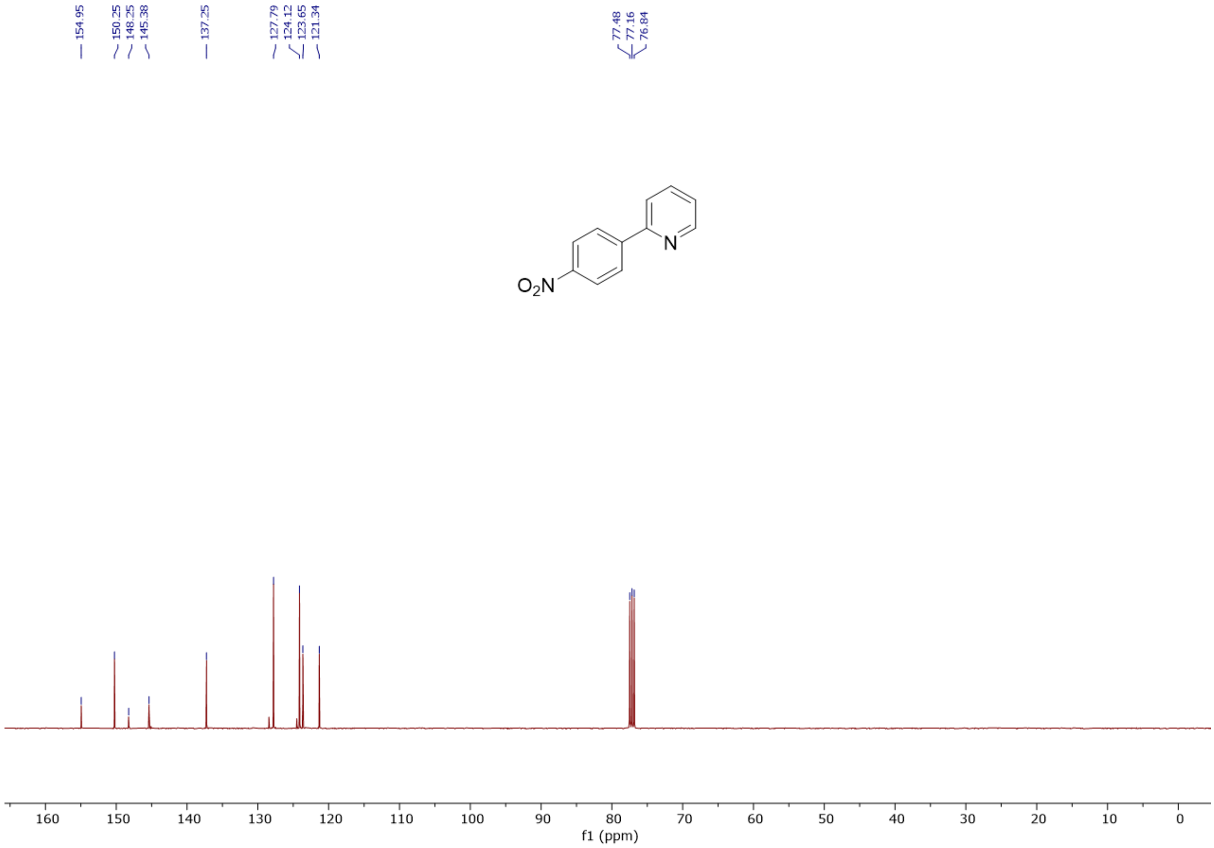
154.95
150.25
148.25
145.38
137.25
127.79
124.12
123.65
121.34
77.48
77.16
76.84
O2N
N
f1 (ppm)

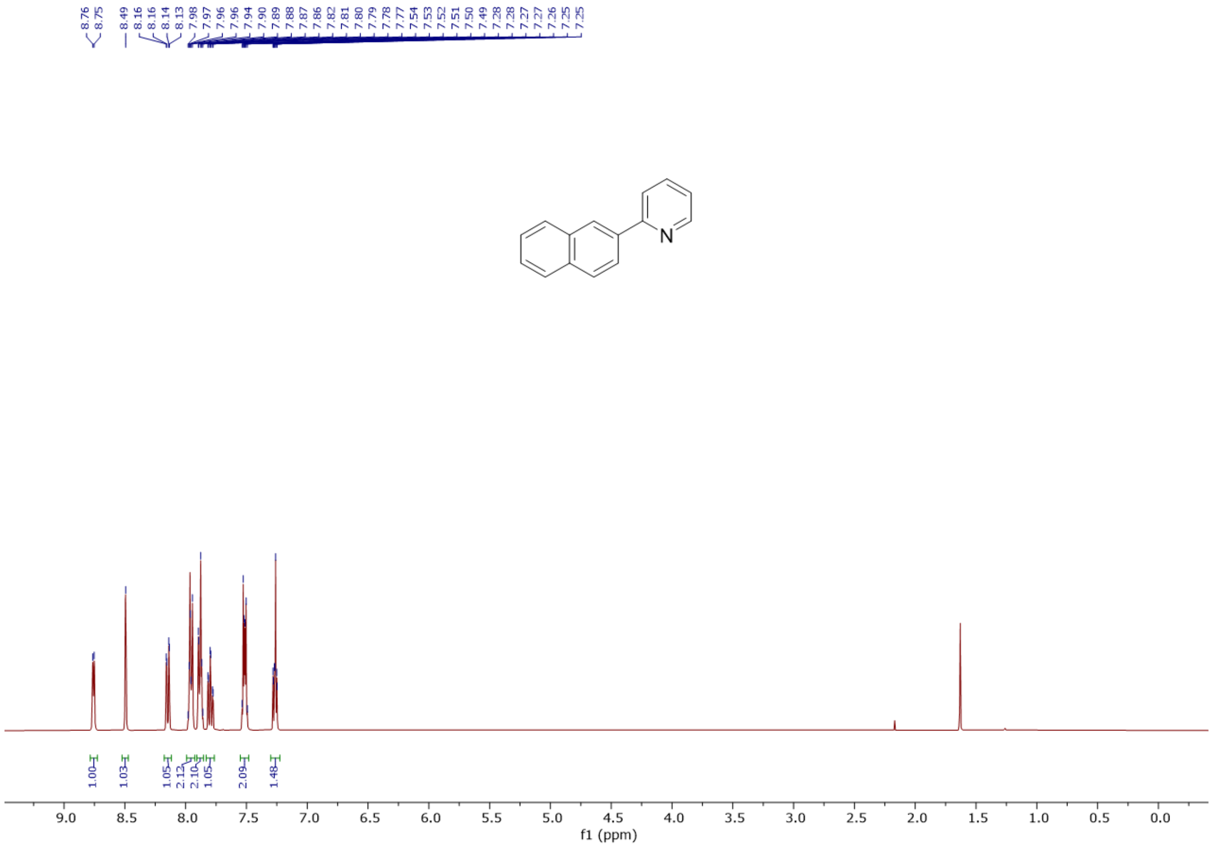
8.76
8.75
8.49
8.16
8.16
8.14
8.13
7.98
7.97
7.96
7.96
7.94
7.90
7.89
7.88
7.87
7.86
7.82
7.81
7.80
7.79
7.78
7.77
7.54
7.53
7.52
7.51
7.50
7.49
7.28
7.28
7.27
7.27
7.26
7.25
7.25
N
1.00
1.03
1.05
2.13
2.10
1.05
2.09
1.48
f1 (ppm)

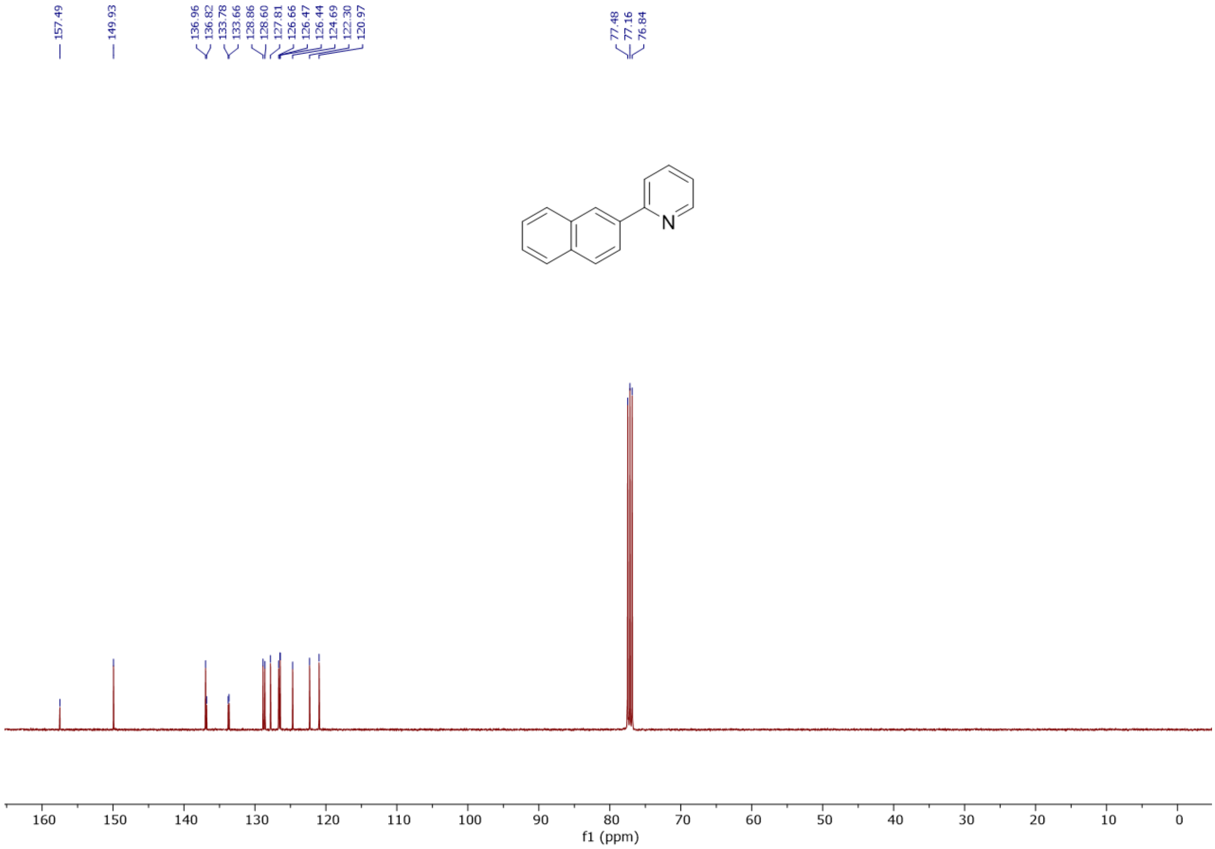
157.49
149.93
136.96
136.82
133.78
133.66
128.86
128.60
127.81
126.66
126.47
126.44
124.69
122.30
120.97
77.48
77.16
76.84
N
f1 (ppm)

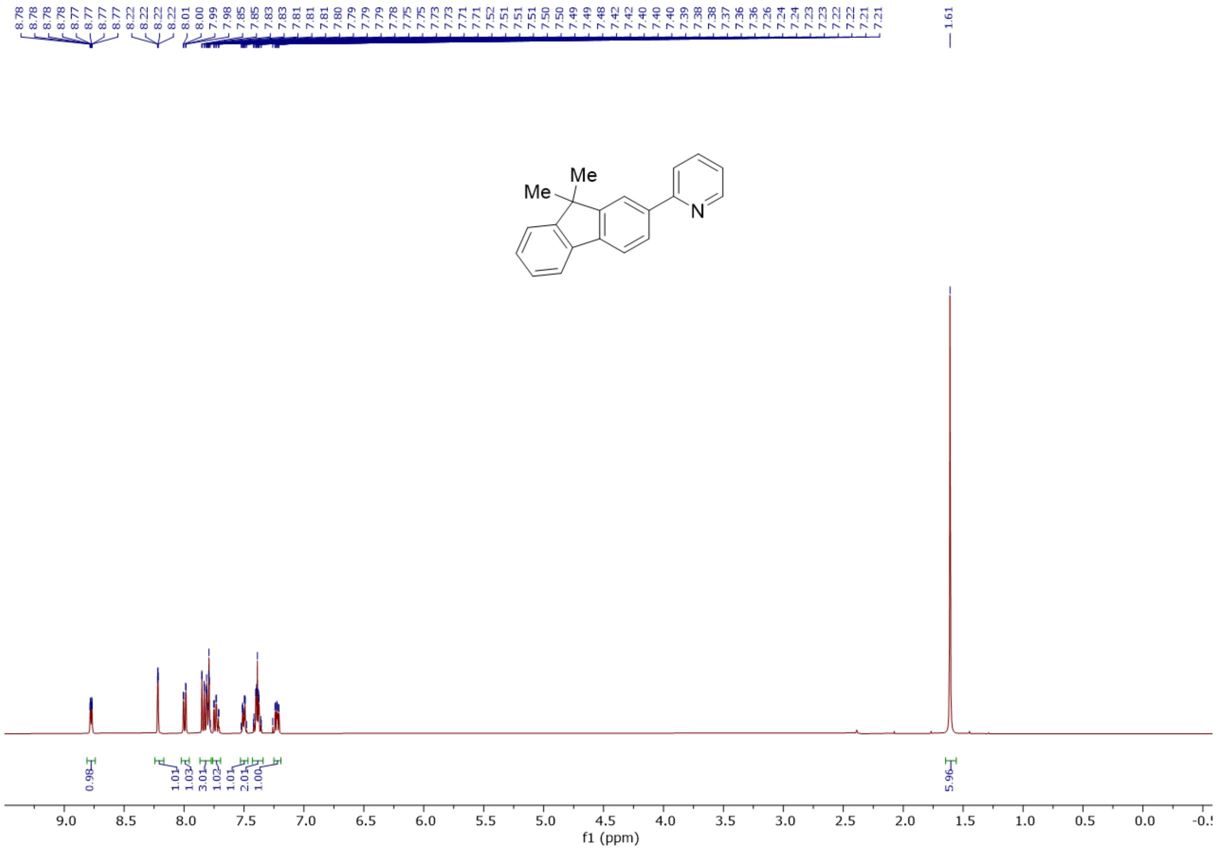

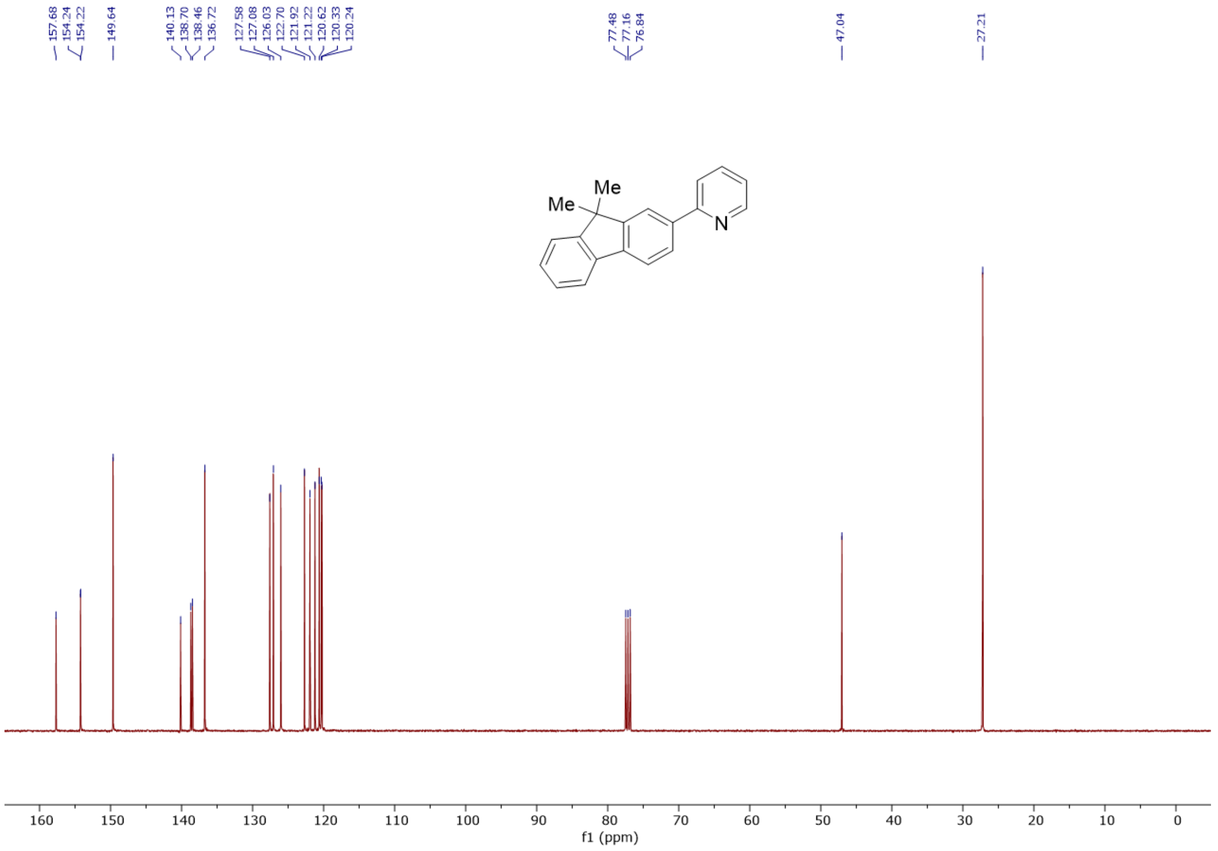

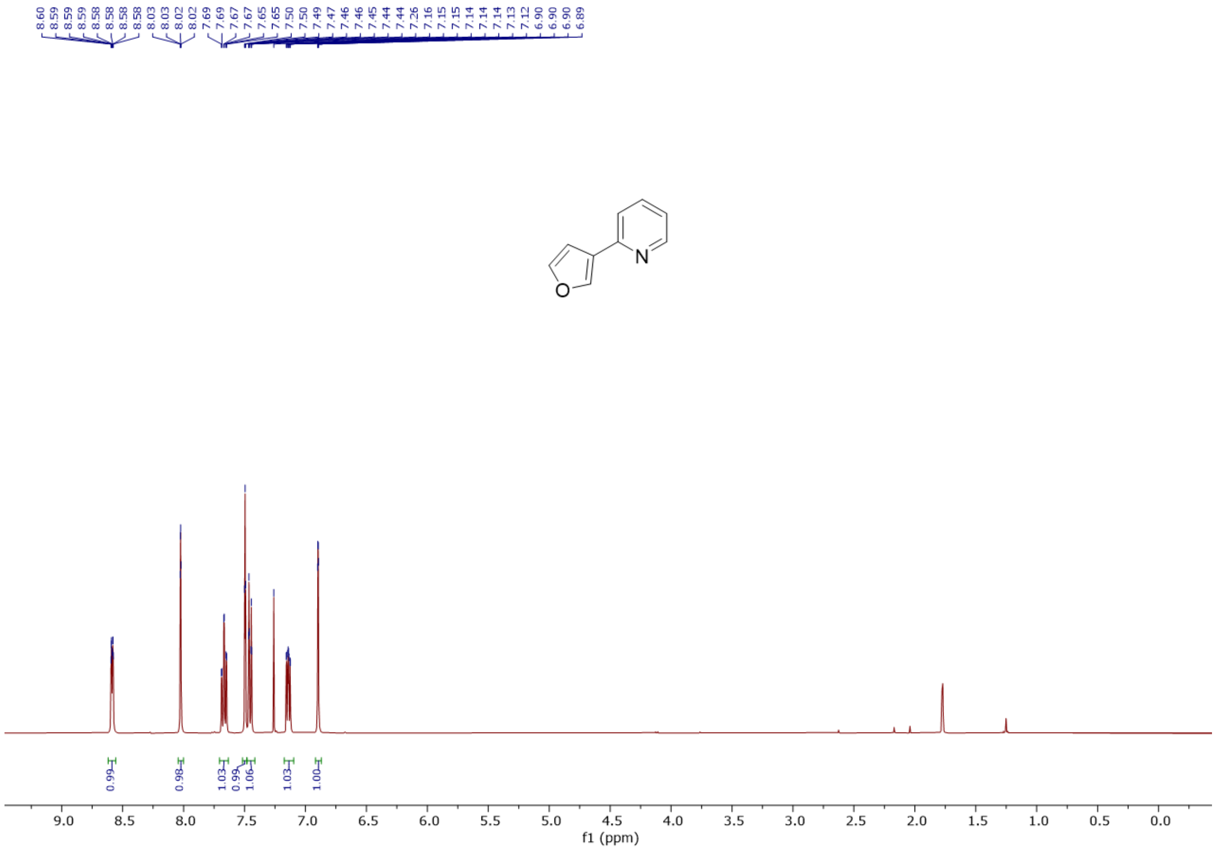

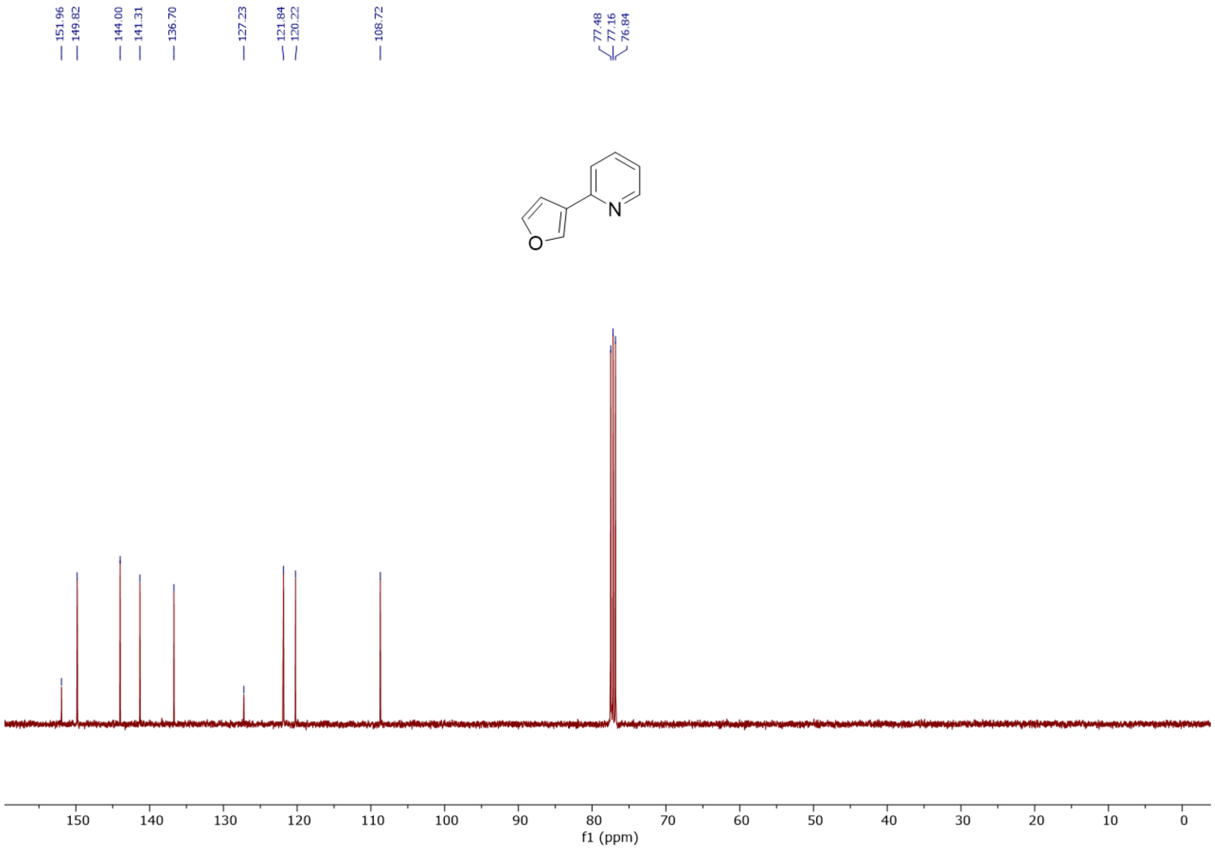

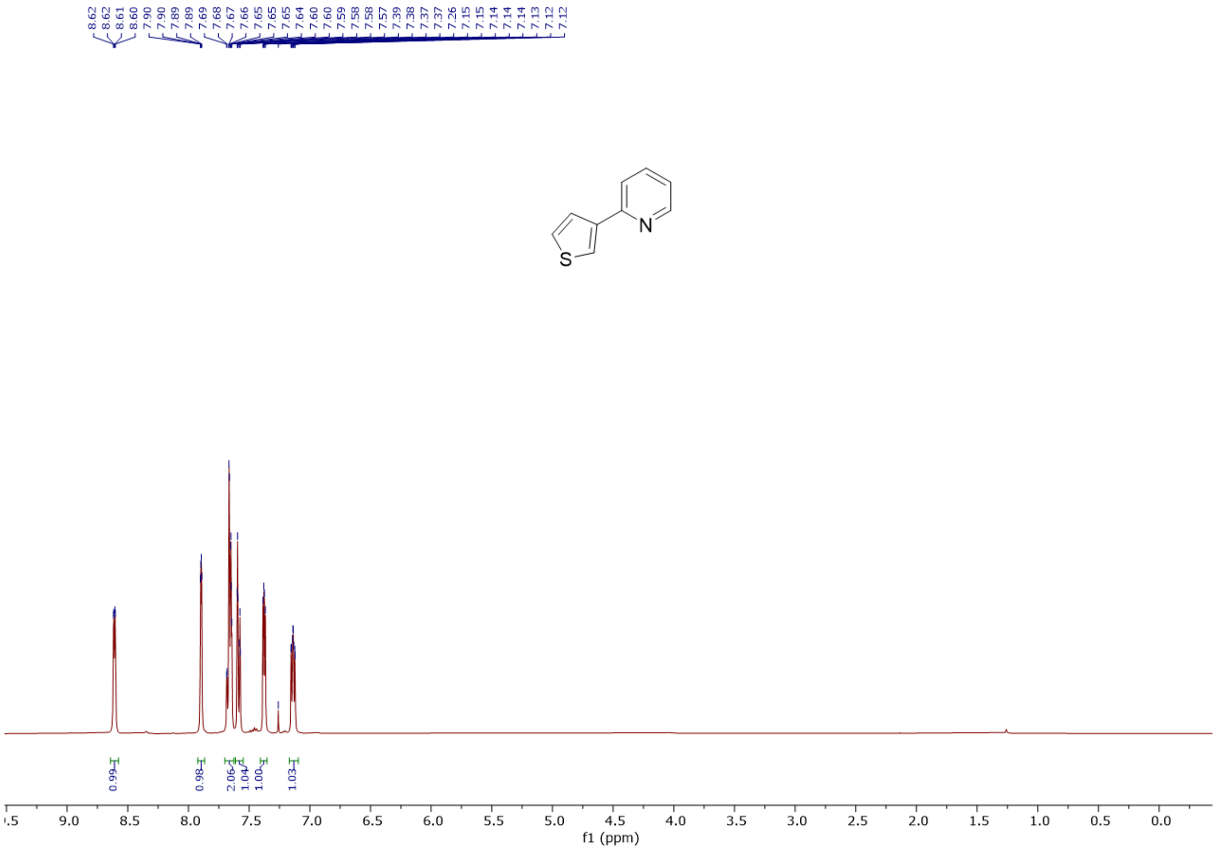
8.62
8.62
8.61
8.60
7.90
7.90
7.89
7.89
7.69
7.68
7.67
7.66
7.65
7.65
7.65
7.64
7.60
7.60
7.59
7.58
7.58
7.57
7.39
7.38
7.37
7.37
7.26
7.15
7.15
7.14
7.14
7.13
7.12
7.12
N
S
0.99
0.98
2.06
1.04
1.00
1.03
f1 (ppm)

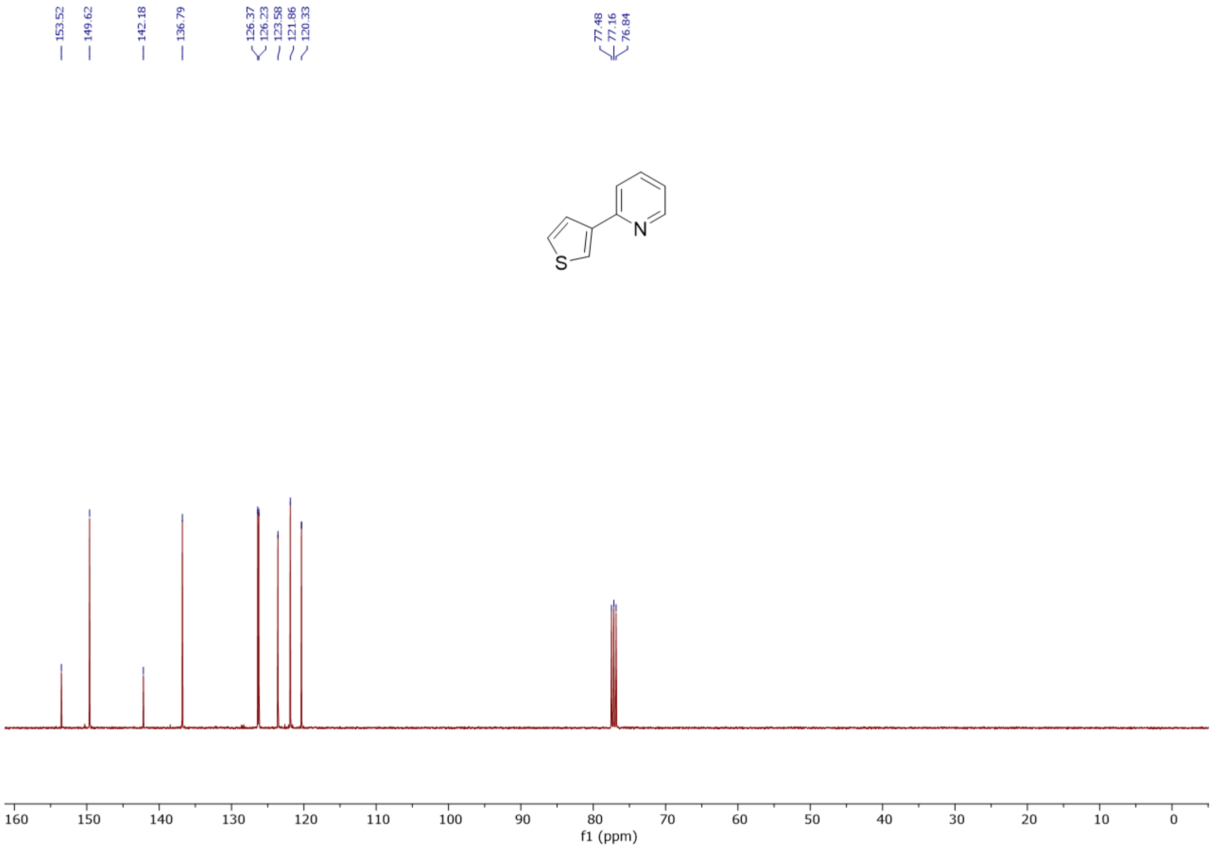
153.52
149.62
142.18
136.79
126.37
126.23
123.58
121.86
120.33
77.48
77.16
76.84
N
S
f1 (ppm)

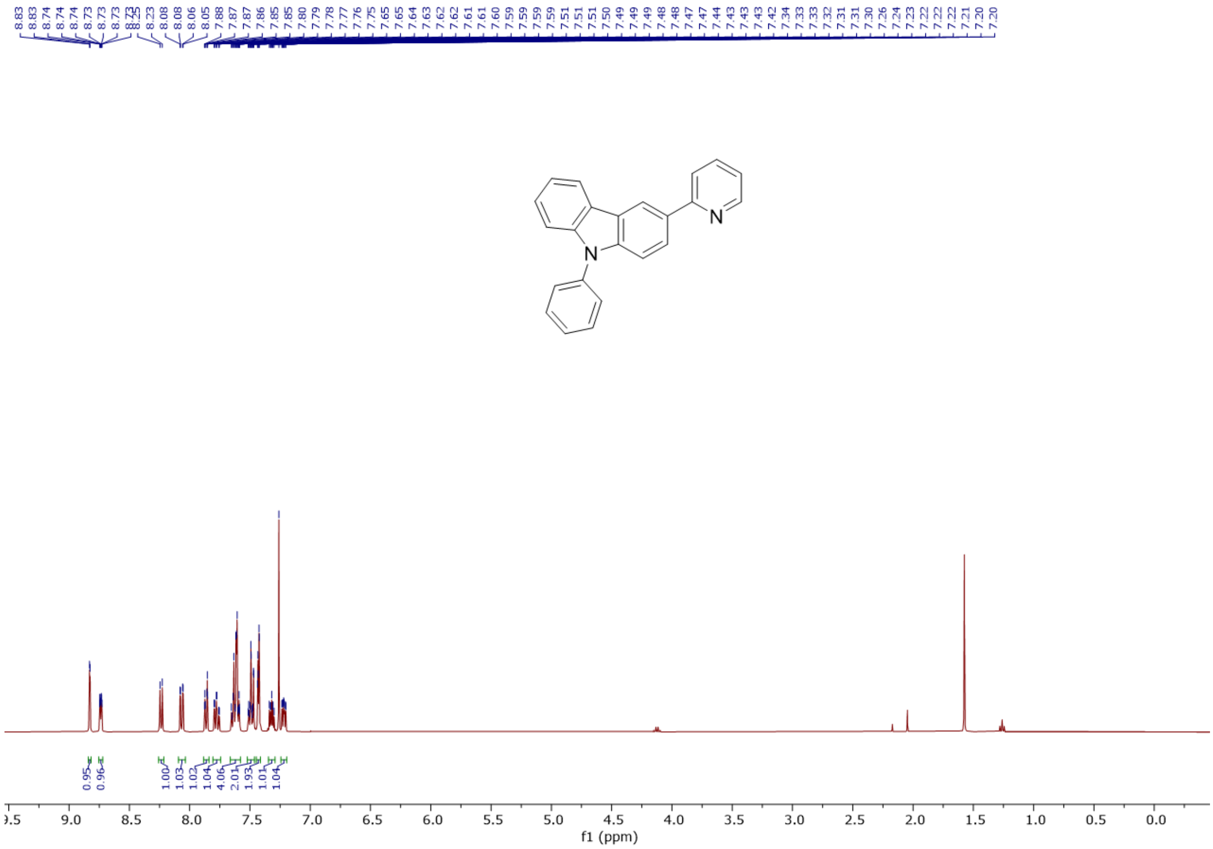
8.83 8.83 8.74 8.74 8.74 8.73 8.73 8.73 8.73 8.23 8.23 8.08 8.08 8.06 8.05 7.88 7.87 7.87 7.86 7.85 7.85 7.80 7.79 7.78 7.77 7.76 7.75 7.65 7.65 7.64 7.63 7.62 7.61 7.60 7.59 7.59 7.59 7.59 7.51 7.51 7.51 7.50 7.49 7.49 7.49 7.48 7.48 7.47 7.47 7.44 7.43 7.43 7.43 7.42 7.34 7.33 7.33 7.32 7.31 7.31 7.30 7.26 7.24 7.23 7.22 7.22 7.22 7.21 7.20 7.20
N
N
0.95 0.96 1.00 1.03 1.02 1.04 4.06 2.01 1.93 1.01 1.04
f1 (ppm)

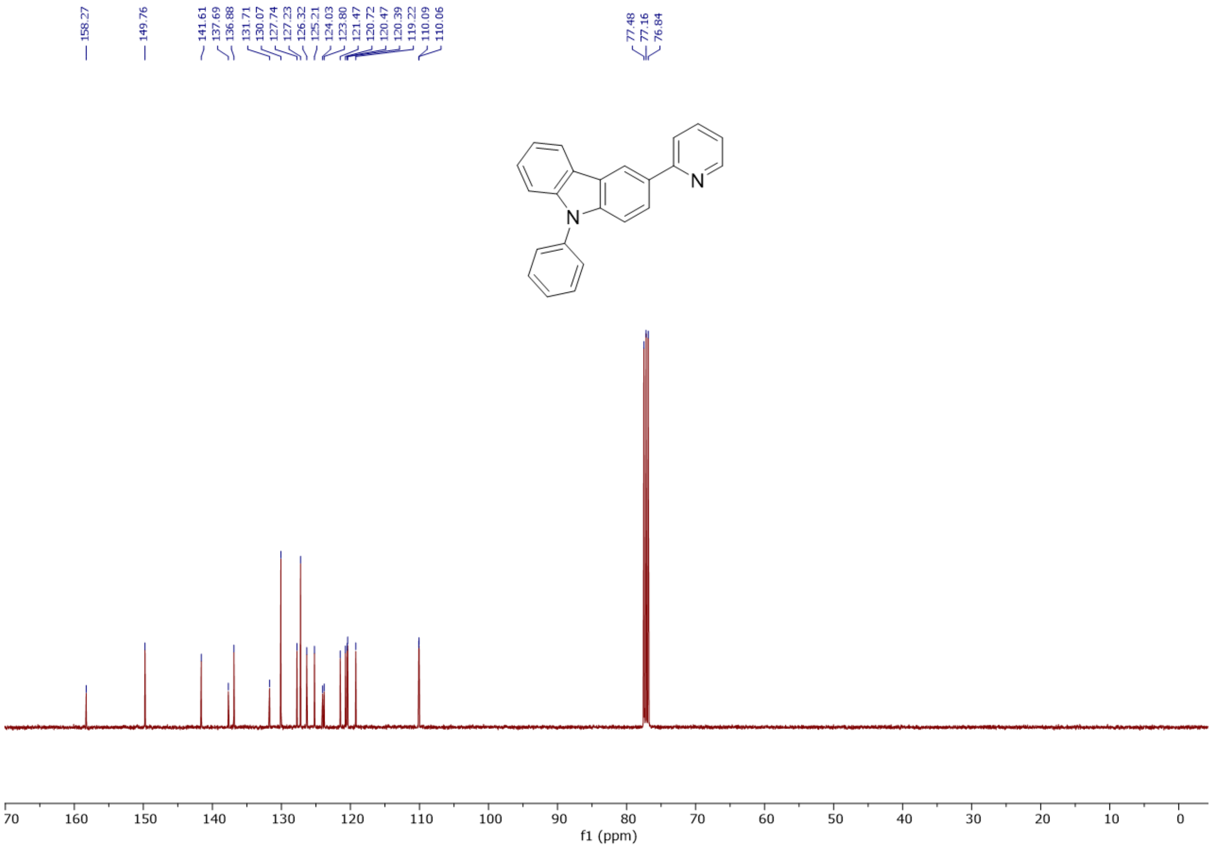
158.27
149.76
141.61 137.69 136.88 131.71 130.07 127.74 127.23 126.32 125.21 124.03 123.80 121.47 120.72 120.47 120.39 119.22 110.09 110.06
77.48 77.16 76.84
N
N
f1 (ppm)

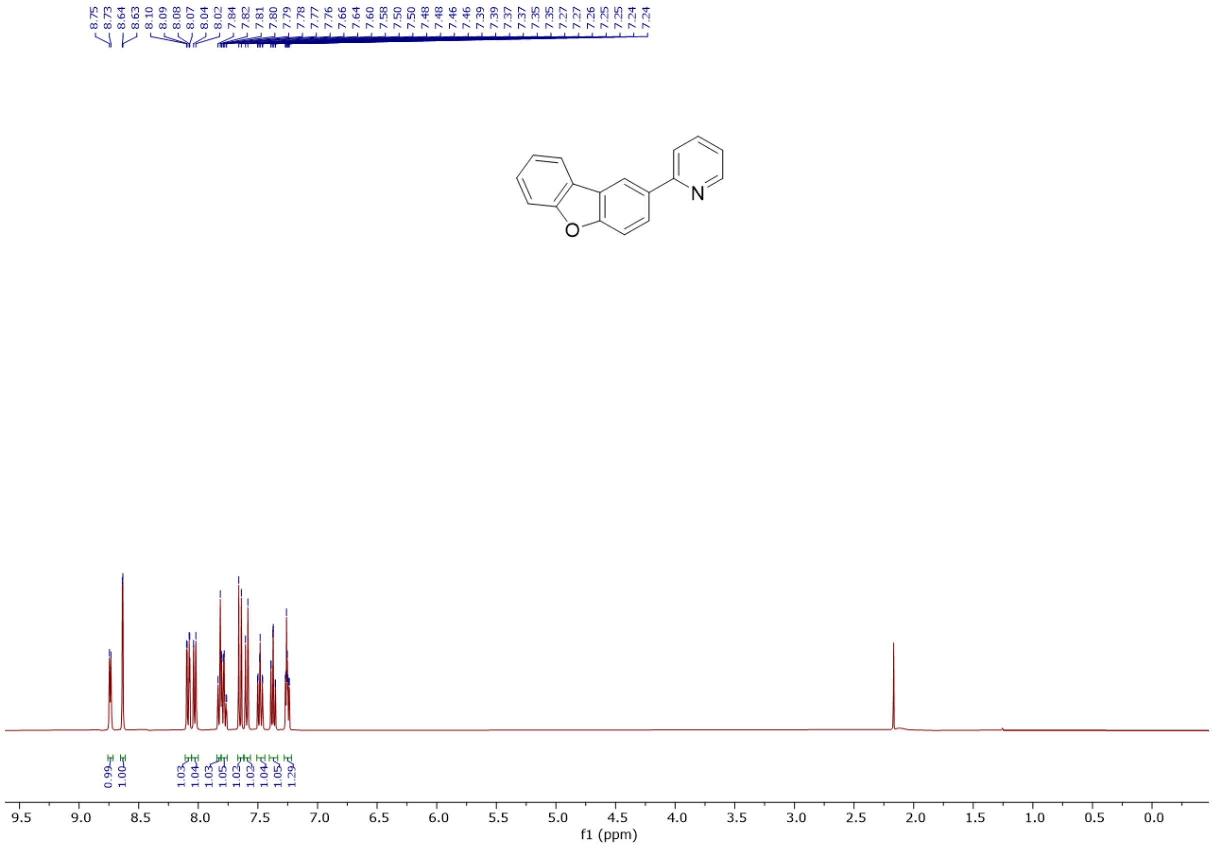
8.75
8.73
8.64
8.63
8.10
8.09
8.08
8.07
8.04
8.02
7.84
7.82
7.81
7.80
7.79
7.78
7.77
7.76
7.66
7.64
7.60
7.58
7.50
7.50
7.48
7.48
7.46
7.46
7.39
7.39
7.37
7.37
7.35
7.35
7.27
7.27
7.26
7.25
7.25
7.24
7.24
N
O
0.99
1.00
1.03
1.04
1.03
1.05
1.02
1.02
1.04
1.05
1.29
9.5 9.0 8.5 8.0 7.5 7.0 6.5 6.0 5.5 5.0 4.5 4.0 3.5 3.0 2.5 2.0 1.5 1.0 0.5 0.0
f1 (ppm)

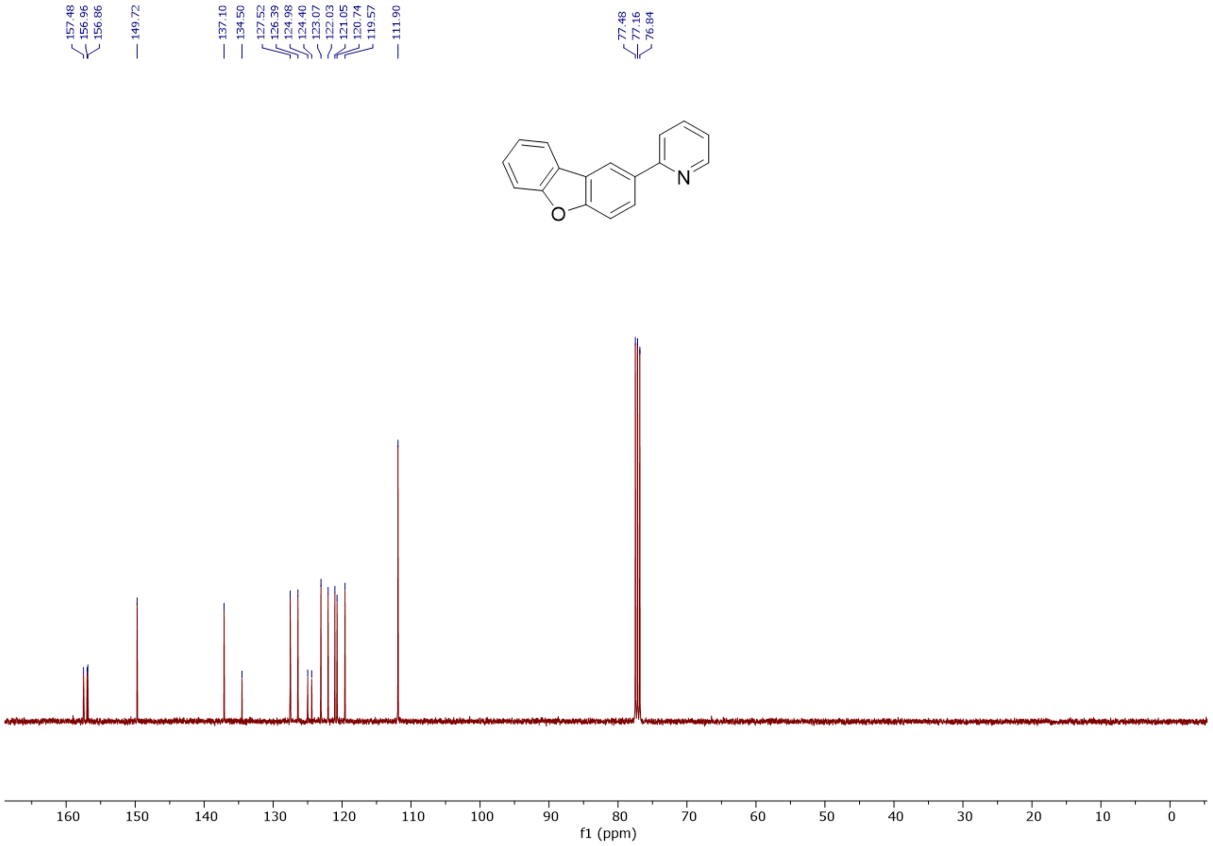
157.48
156.96
156.86
149.72
137.10
134.50
127.52
126.39
124.98
124.40
123.07
122.03
121.05
120.74
119.57
111.90
77.48
77.16
76.84
N
O
160 150 140 130 120 110 100 90 80 70 60 50 40 30 20 10 0
f1 (ppm)

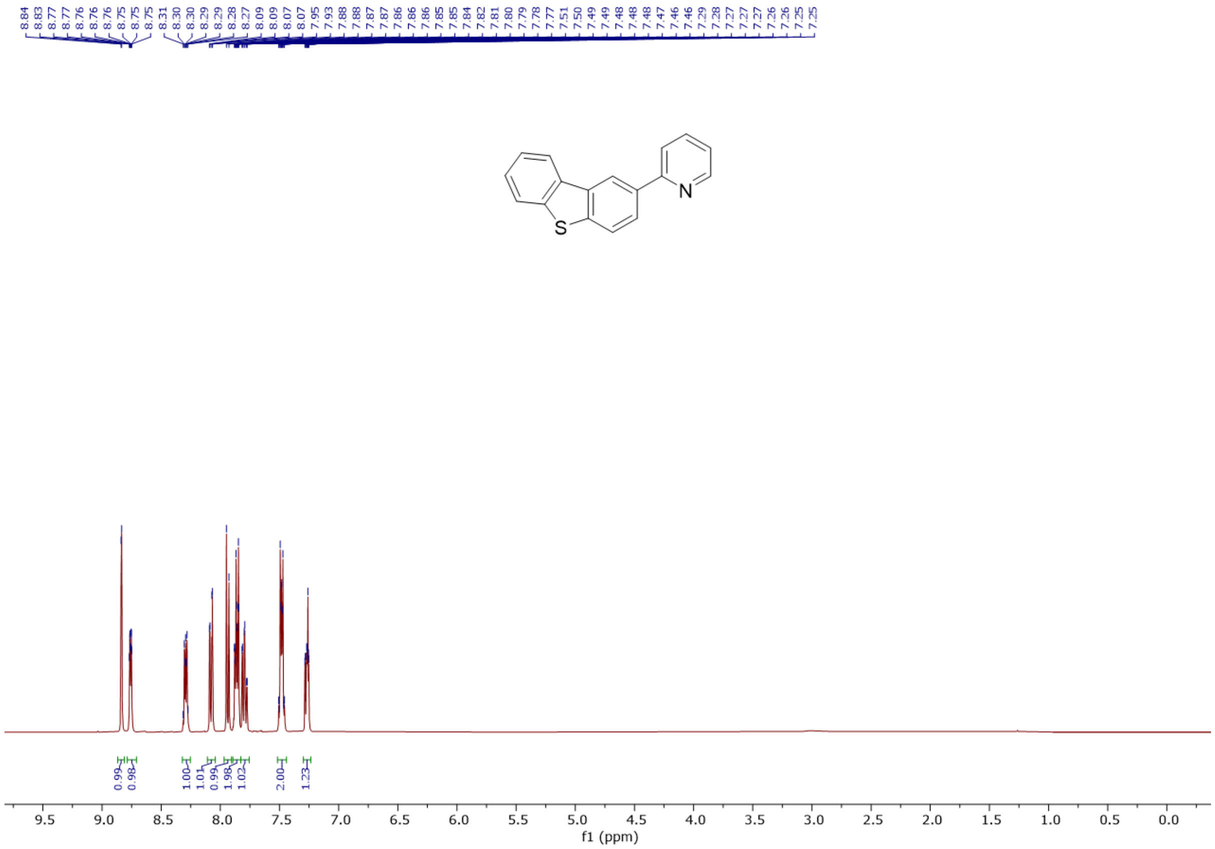

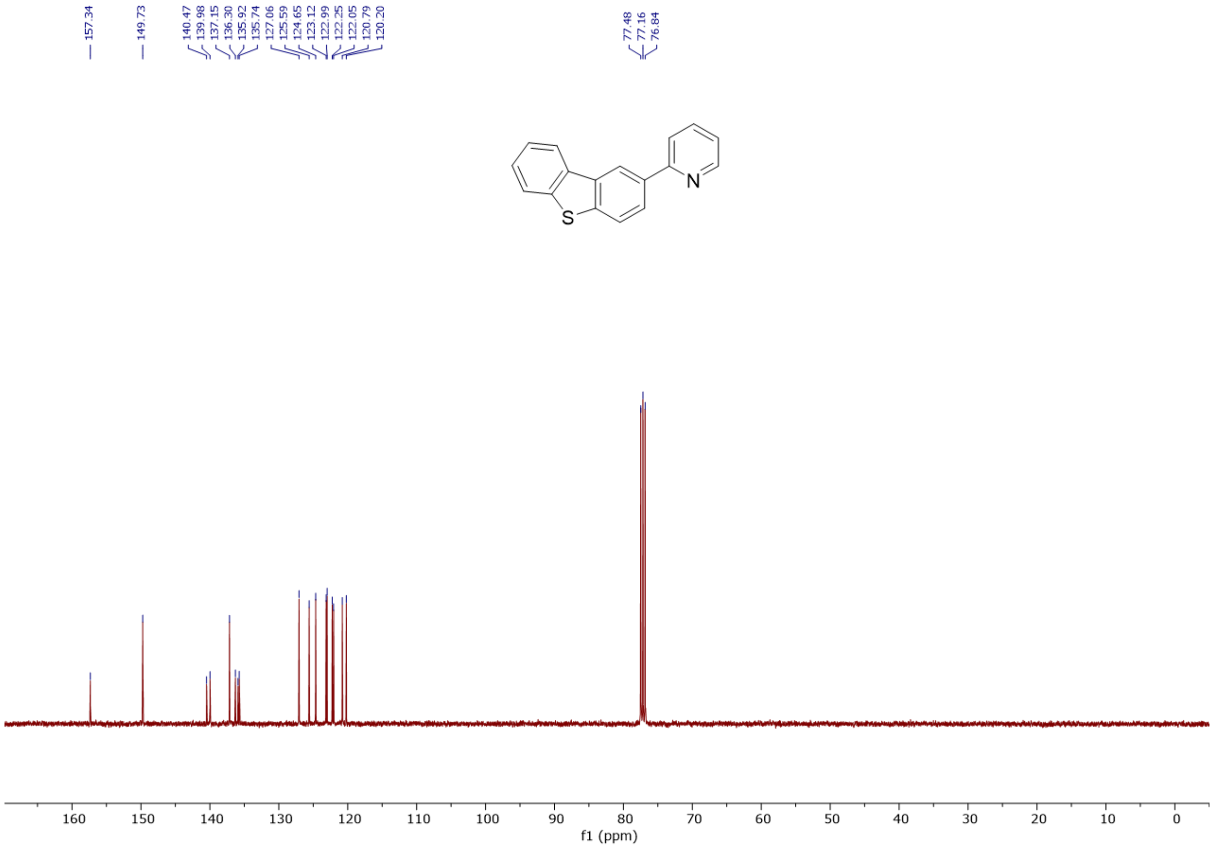

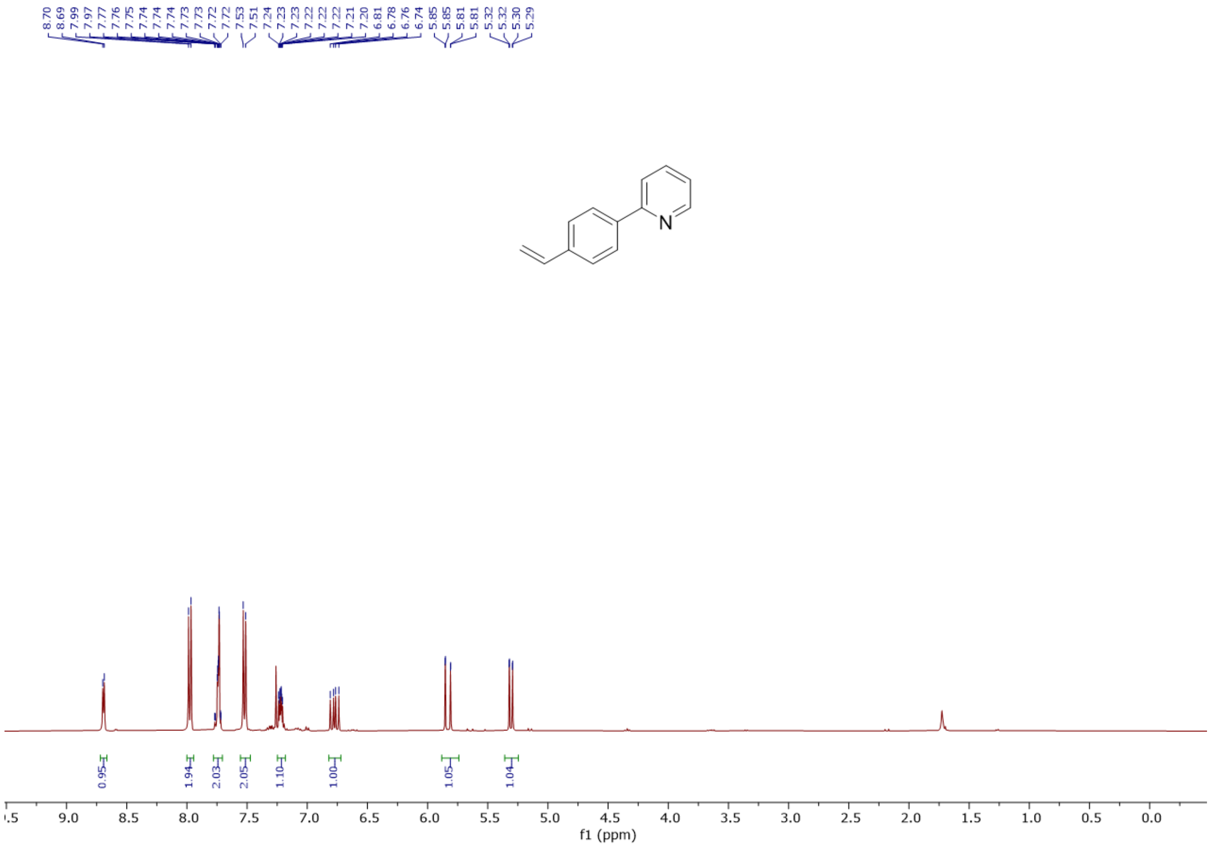
8.70
8.69
7.99
7.97
7.77
7.76
7.75
7.74
7.74
7.74
7.73
7.73
7.72
7.72
7.53
7.51
7.24
7.23
7.23
7.22
7.22
7.22
7.22
7.21
7.20
6.81
6.78
6.76
6.74
5.85
5.85
5.81
5.81
5.32
5.32
5.30
5.29
N
0.95
1.94
2.03
2.05
1.10
1.00
1.05
1.04
f1 (ppm)

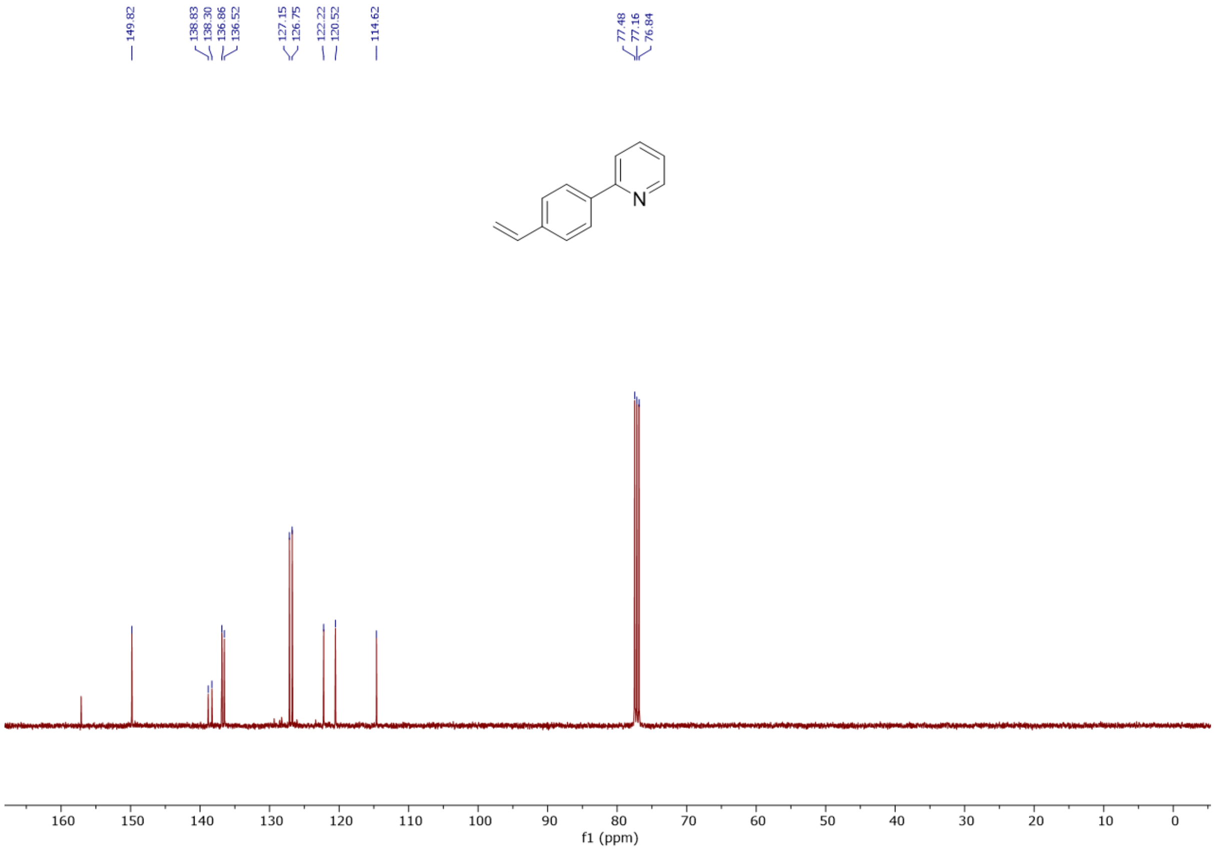
149.82
138.83
138.30
136.86
136.52
127.15
126.75
122.22
120.52
114.62
77.48
77.16
76.84
N
f1 (ppm)

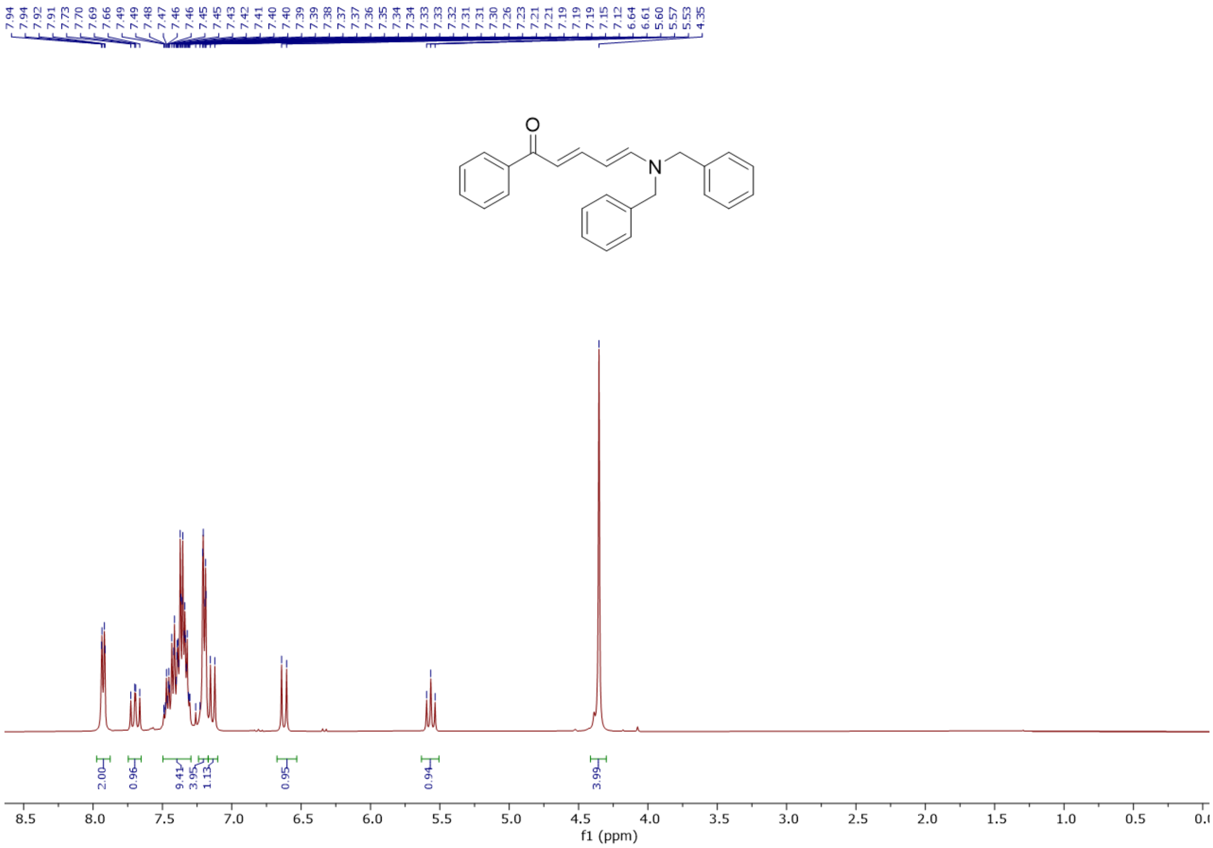

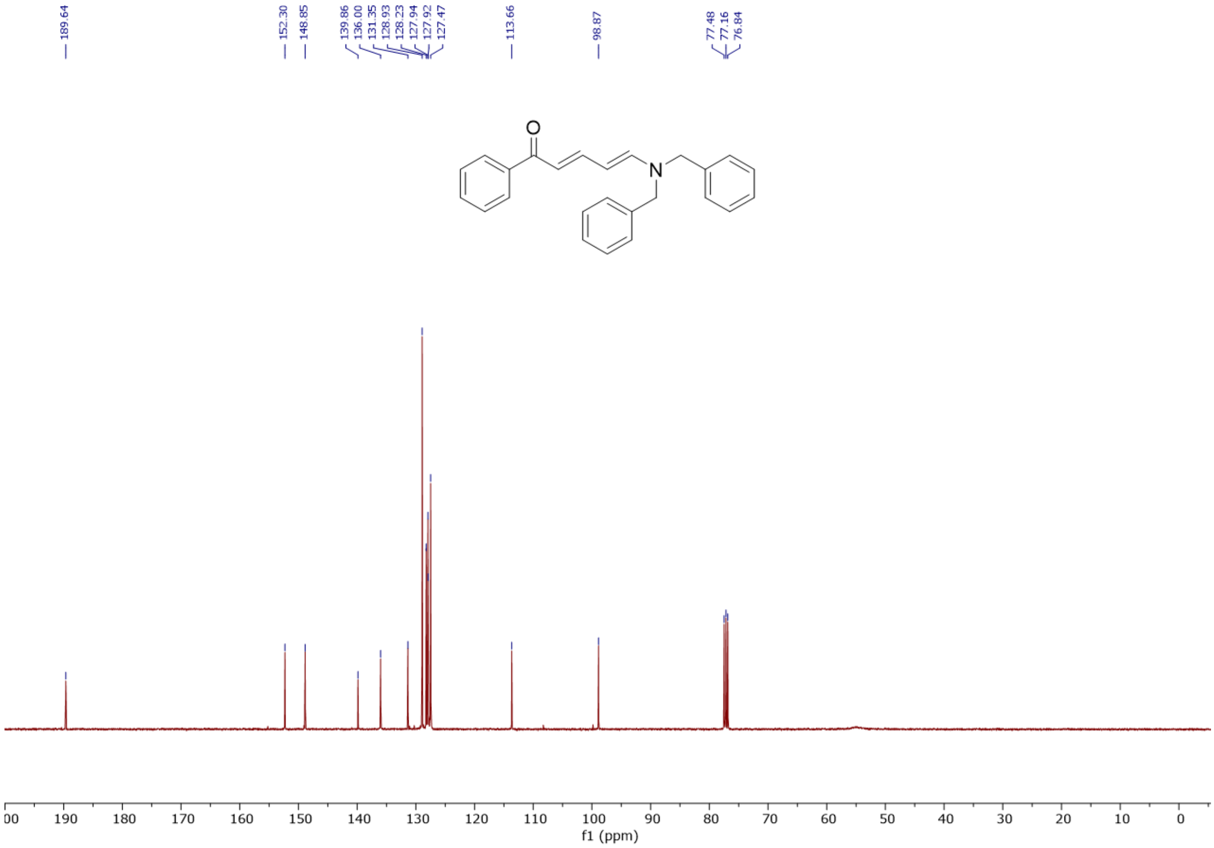

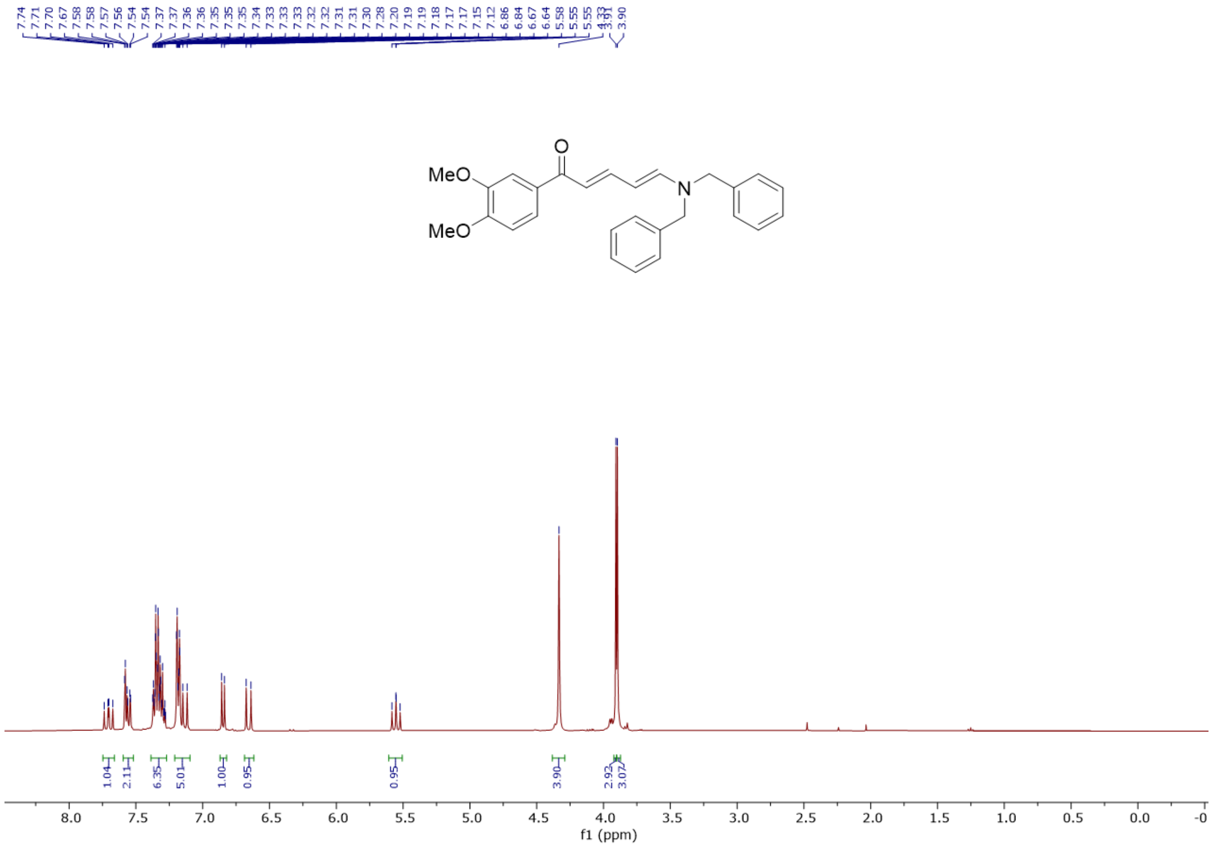
MeO
MeO
O
N
f1 (ppm)

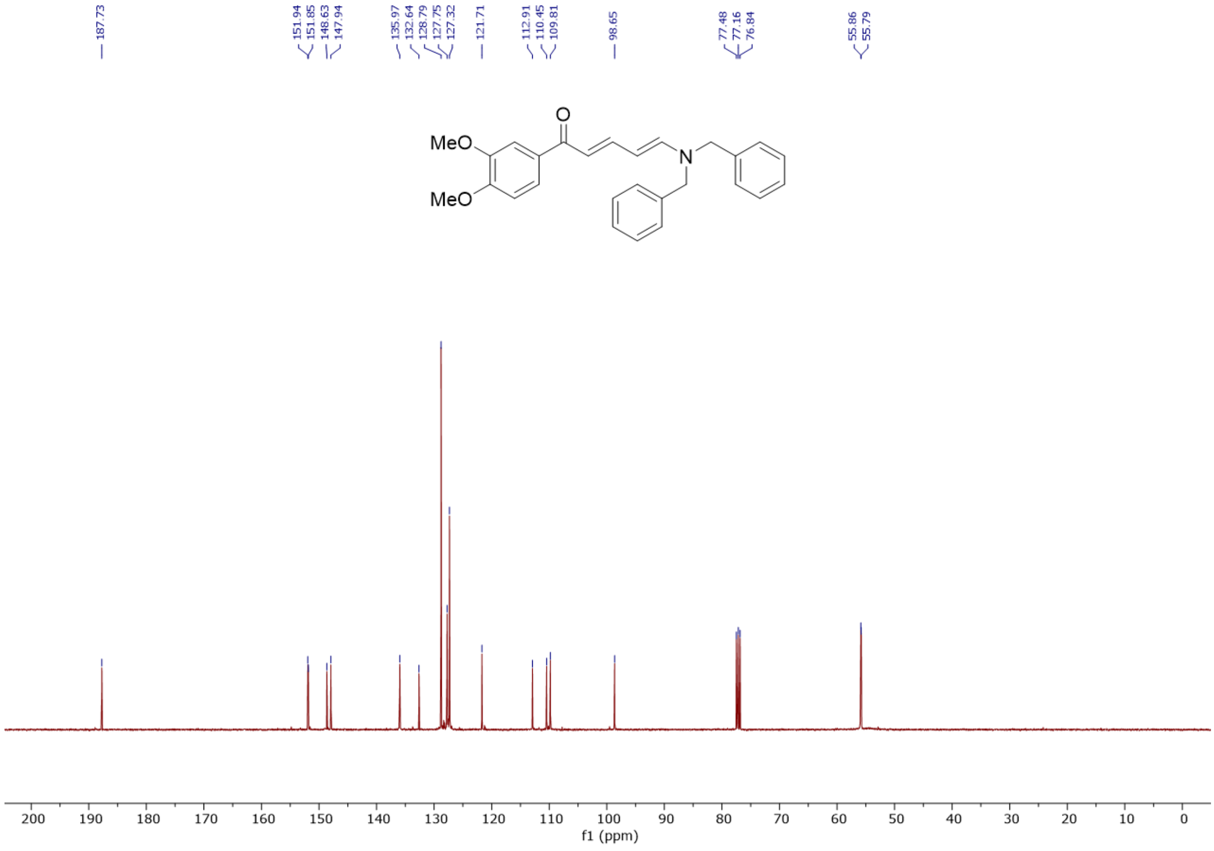
MeO
MeO
O
N
f1 (ppm)

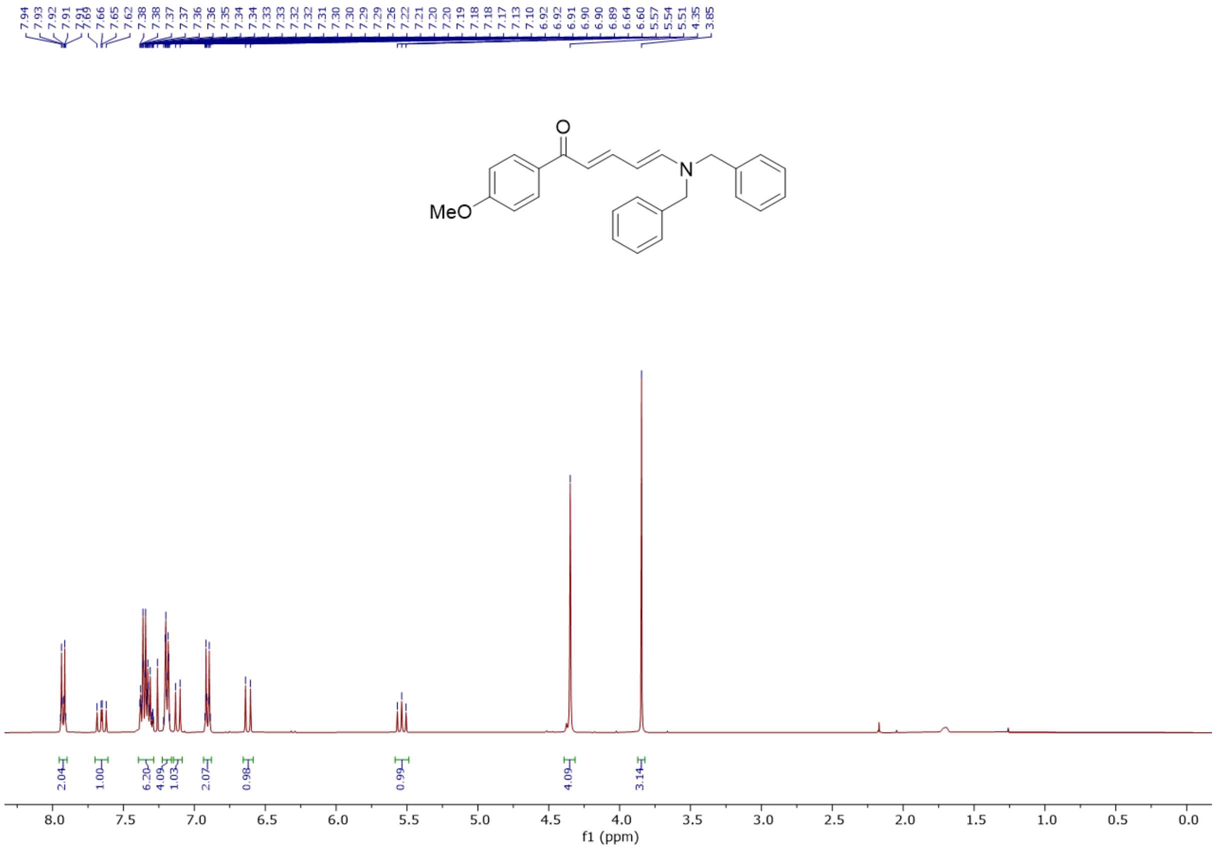
7.94
7.93
7.92
7.91
7.91
7.69
7.66
7.65
7.62
7.38
7.38
7.37
7.37
7.36
7.36
7.35
7.34
7.34
7.33
7.33
7.32
7.32
7.31
7.30
7.30
7.29
7.29
7.26
7.22
7.21
7.20
7.20
7.19
7.18
7.18
7.17
7.13
7.10
6.92
6.92
6.91
6.90
6.90
6.89
6.64
6.60
5.57
5.54
5.51
4.35
3.85
MeO
O
N
2.04
1.00
6.20
4.09
1.03
2.07
0.98
0.99
4.09
3.14
f1 (ppm)

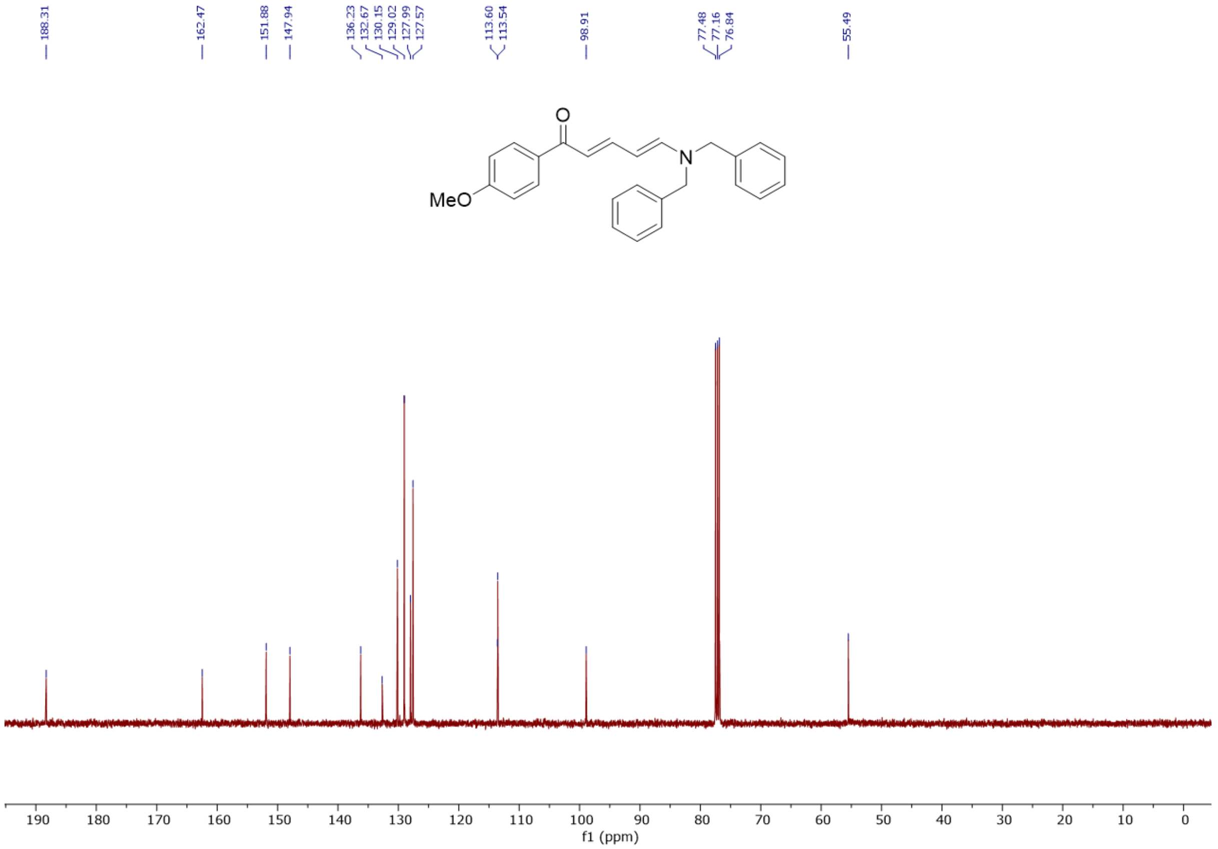
188.31
162.47
151.88
147.94
136.23
132.67
130.15
129.02
127.99
127.57
113.60
113.54
98.91
77.48
77.16
76.84
55.49
MeO
O
N
f1 (ppm)

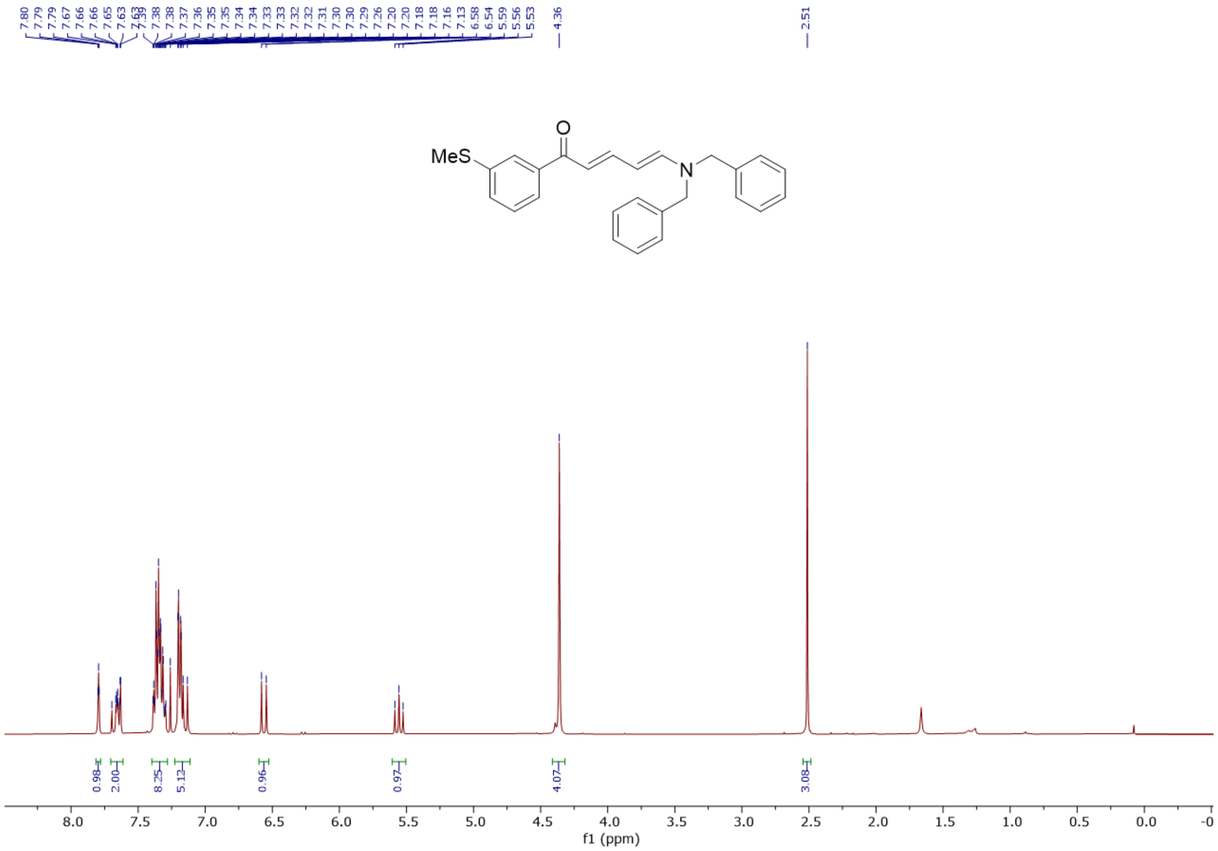
7.80 7.79 7.79 7.67 7.66 7.65 7.63 7.63 7.39 7.38 7.38 7.37 7.36 7.35 7.35 7.34 7.34 7.33 7.33 7.32 7.32 7.31 7.30 7.30 7.29 7.26 7.20 7.20 7.18 7.18 7.16 7.13 6.58 6.54 5.59 5.56 5.53
4.36
2.51
MeS
O
N
0.98
2.00
8.25
5.12
0.96
0.97
4.07
3.08
f1 (ppm)

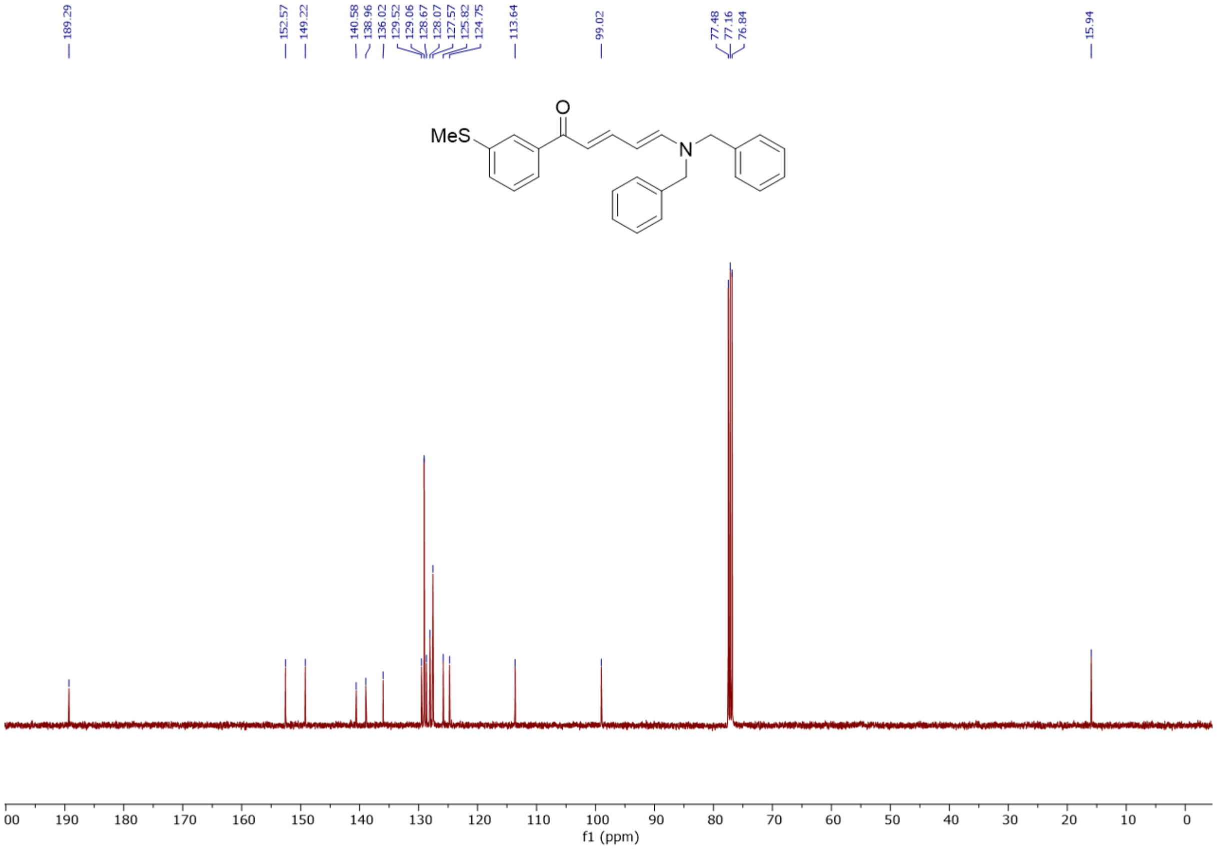
189.29
152.57
149.22
140.58 138.96 136.02 129.52 129.06 128.67 128.07 127.57 125.82 124.75
113.64
99.02
77.48 77.16 76.84
15.94
MeS
O
N
f1 (ppm)

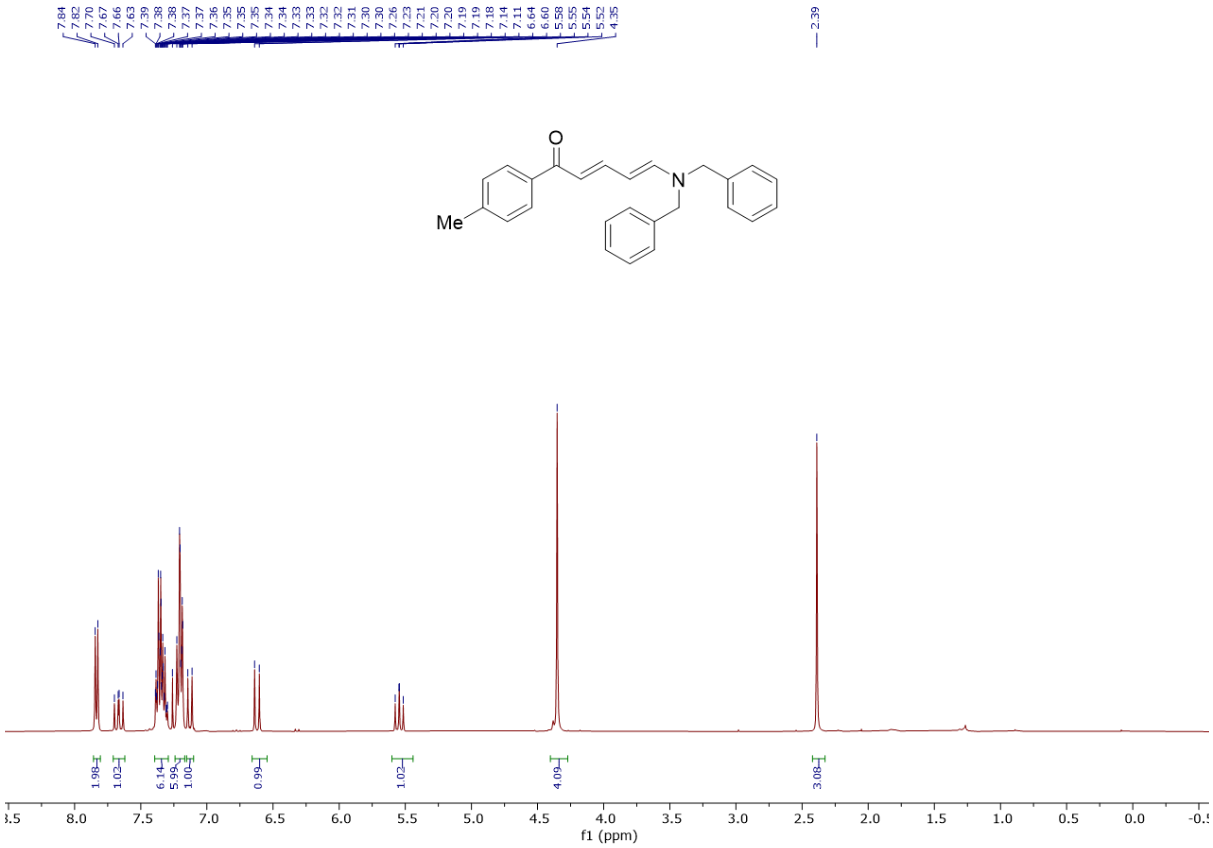
7.84 7.82 7.70 7.67 7.66 7.63 7.39 7.38 7.38 7.37 7.37 7.36 7.35 7.35 7.35 7.34 7.34 7.33 7.33 7.32 7.32 7.31 7.30 7.30 7.30 7.26 7.23 7.21 7.20 7.20 7.19 7.19 7.18 7.14 7.11 6.64 6.60 5.58 5.55 5.54 5.52 4.35
2.39
O
N
Me
1.98 1.02 6.14 5.99 1.00 0.99 1.02 4.09 3.08
8.0 7.5 7.0 6.5 6.0 5.5 5.0 4.5 4.0 3.5 3.0 2.5 2.0 1.5 1.0 0.5 0.0
f1 (ppm)

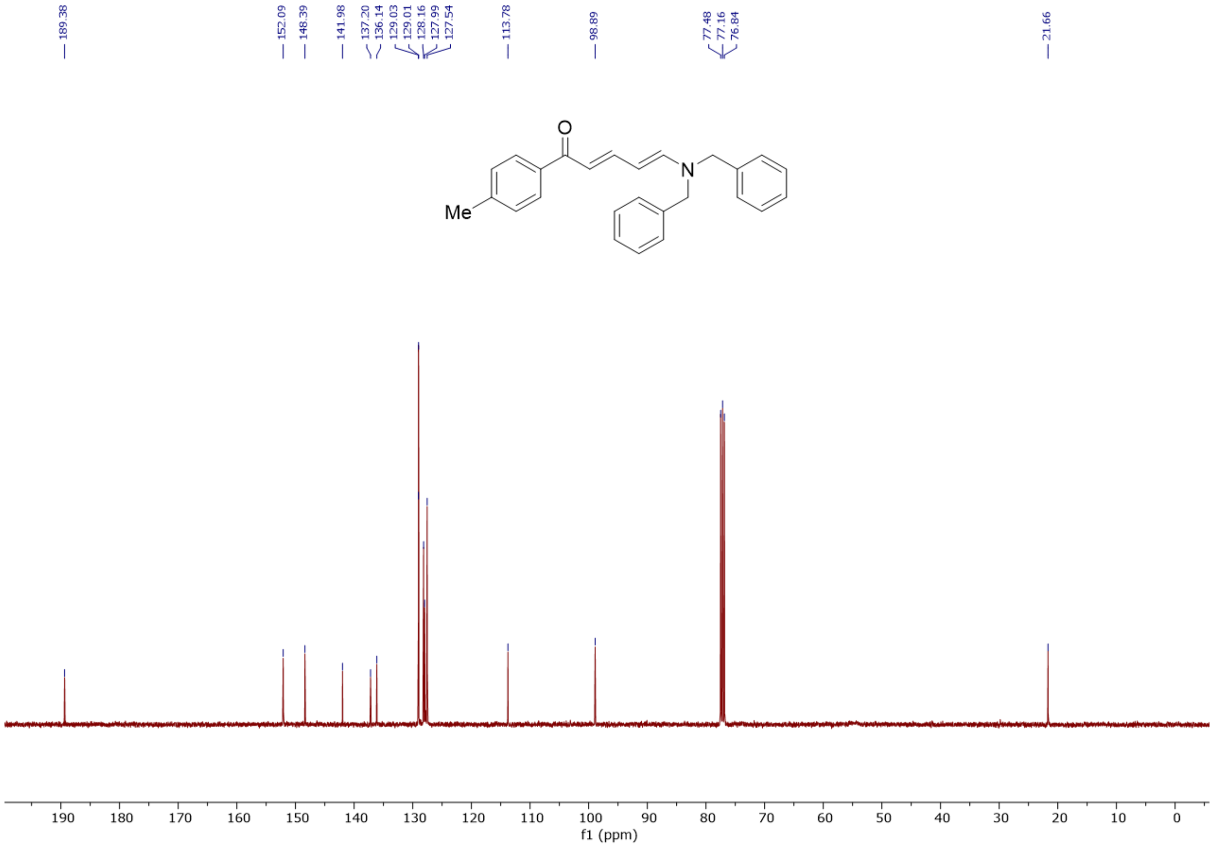
189.38
152.09 148.39 141.98 137.20 136.14 129.03 129.01 128.16 127.99 127.54
113.78
98.89
77.48 77.16 76.84
21.66
O
N
Me
190 180 170 160 150 140 130 120 110 100 90 80 70 60 50 40 30 20 10 0
f1 (ppm)

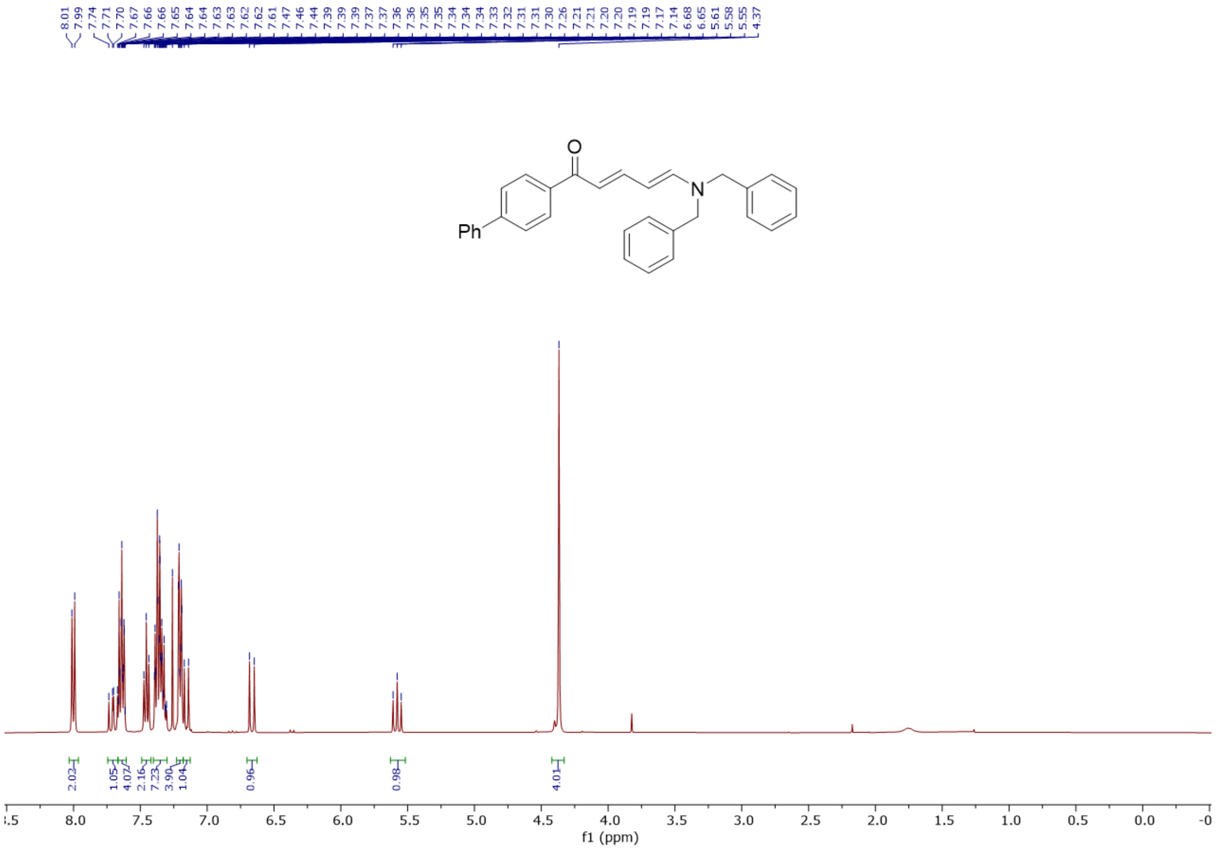
8.01 7.99 7.74 7.71 7.70 7.67 7.66 7.66 7.65 7.64 7.64 7.63 7.63 7.62 7.62 7.61 7.47 7.46 7.44 7.39 7.39 7.39 7.37 7.37 7.36 7.36 7.35 7.35 7.34 7.34 7.34 7.33 7.32 7.31 7.31 7.30 7.26 7.21 7.21 7.20 7.20 7.19 7.19 7.17 7.14 6.68 6.65 5.61 5.58 5.55 4.37
O
N
Ph
2.02
1.05
4.07
2.16
7.23
3.90
1.04
0.96
0.98
4.01
f1 (ppm)

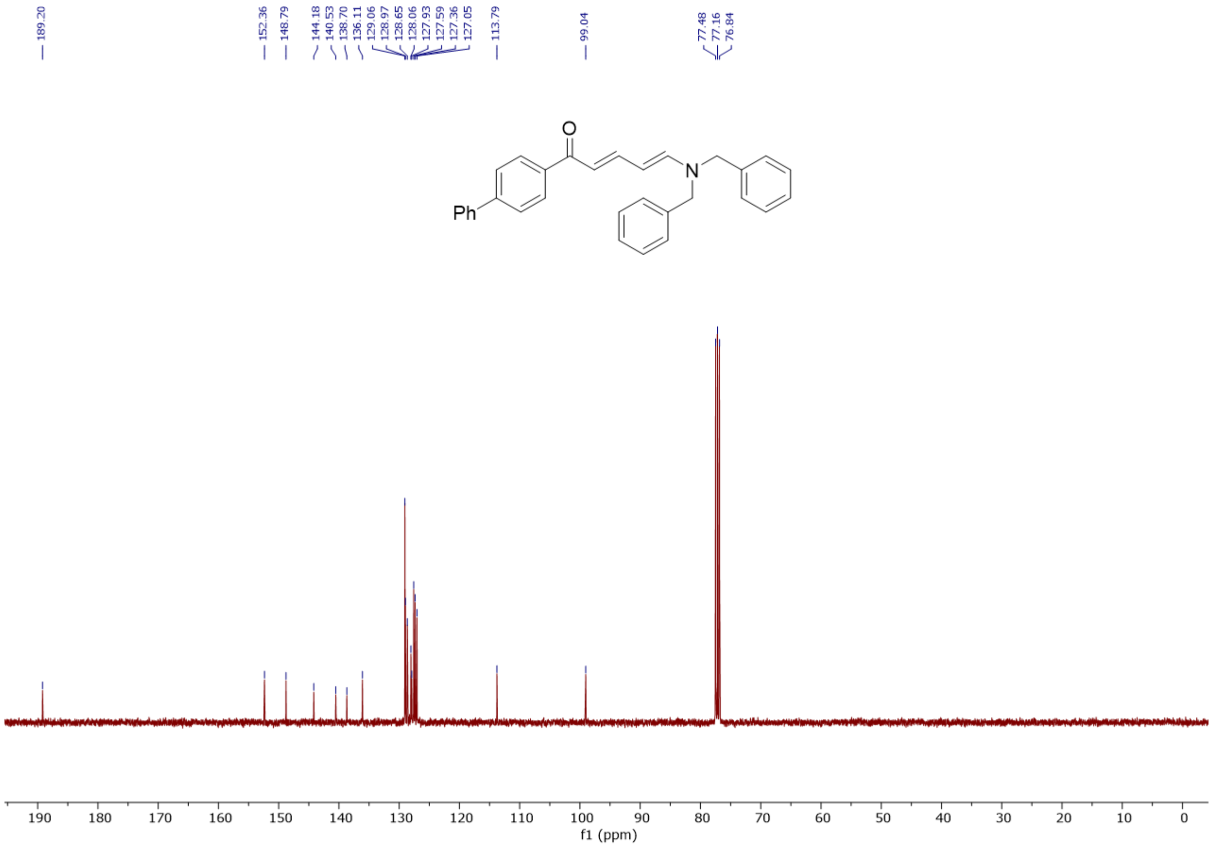
189.20
152.36
148.79
144.18 140.53 138.70 136.11 129.06 128.97 128.65 128.06 127.93 127.59 127.36 127.05
113.79
99.04
77.48 77.16 76.84
O
N
Ph
f1 (ppm)

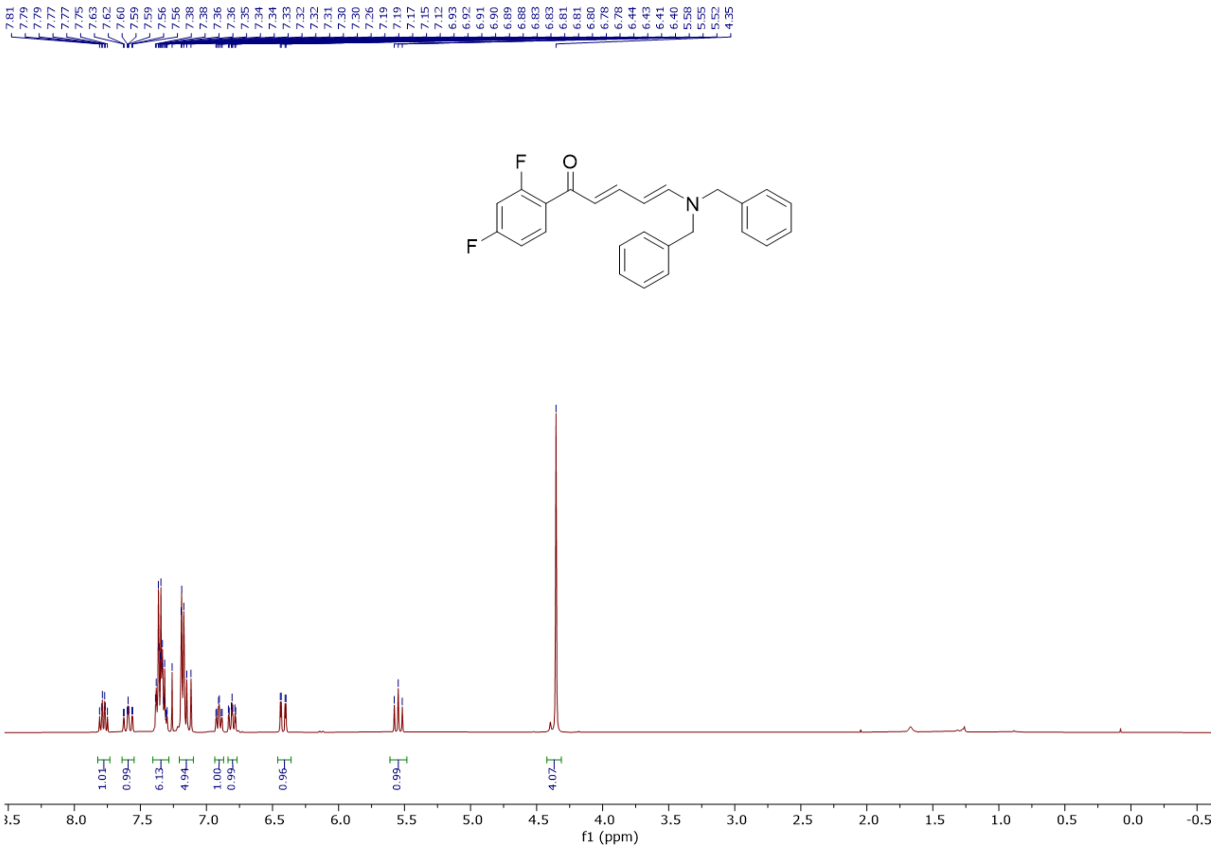
7.81 7.79 7.79 7.77 7.77 7.75 7.63 7.62 7.60 7.59 7.59 7.56 7.56 7.38 7.38 7.36 7.36 7.35 7.34 7.34 7.33 7.32 7.32 7.31 7.30 7.30 7.26 7.19 7.19 7.17 7.15 7.12 6.93 6.92 6.91 6.90 6.89 6.88 6.83 6.83 6.81 6.81 6.80 6.78 6.78 6.44 6.43 6.41 6.40 5.58 5.55 5.52 4.35
F
O
N
F
1.01 0.99 6.13 4.94 1.00 0.99 0.96 0.99 4.07
f1 (ppm)

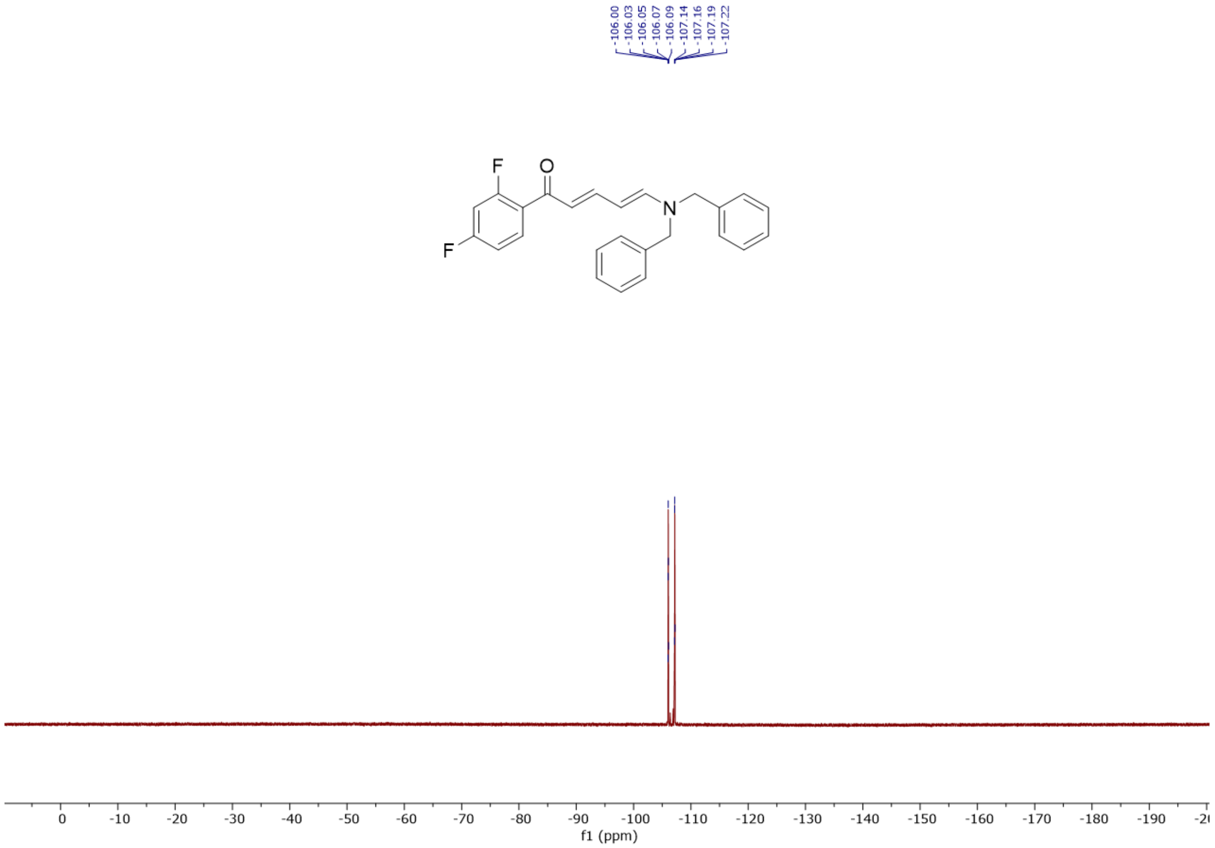
-106.00 -106.03 -106.05 -106.07 -106.09 -107.14 -107.16 -107.19 -107.22
F
O
N
F
f1 (ppm)

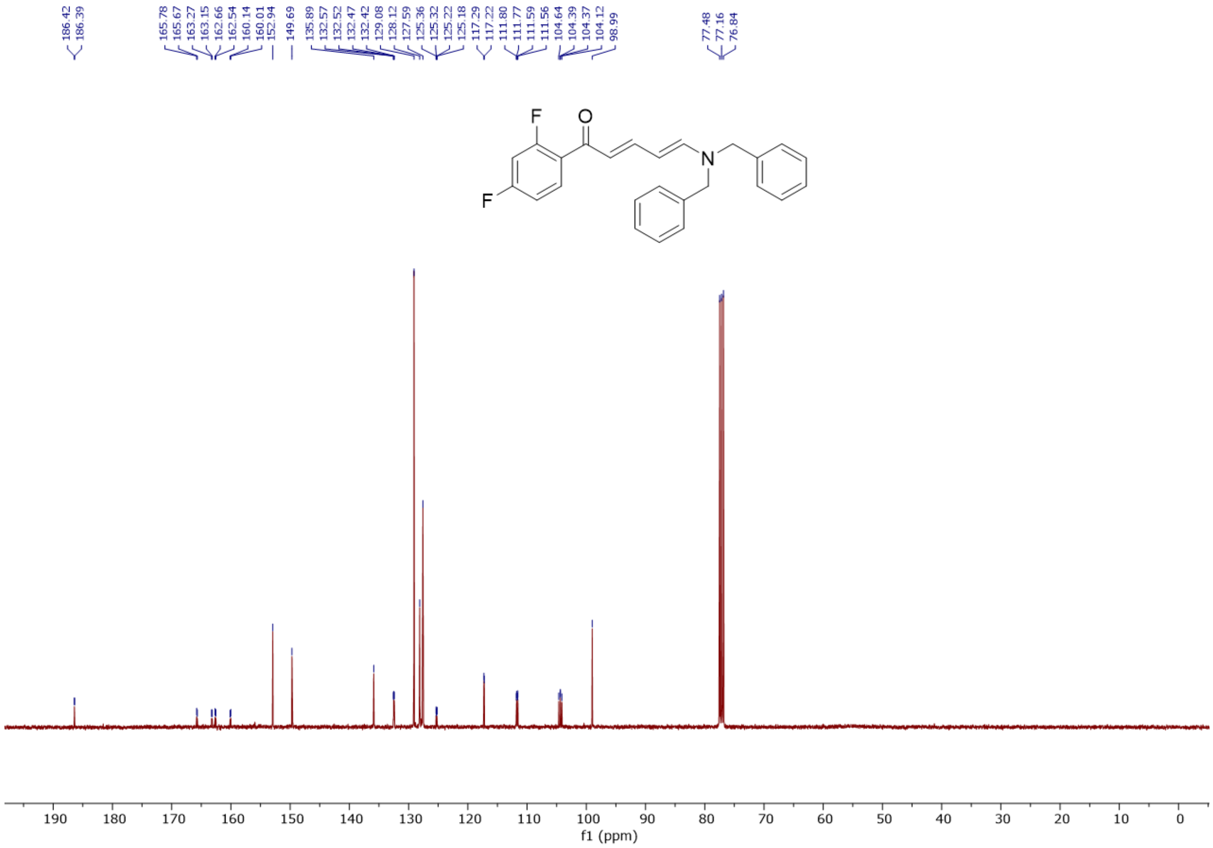
186.42
186.39
165.78
165.67
163.27
163.15
162.66
162.54
160.14
160.01
152.94
149.69
135.89
132.57
132.52
132.47
132.42
129.08
128.12
127.59
125.36
125.32
125.22
125.18
117.29
117.22
111.80
111.77
111.59
111.56
104.64
104.39
104.37
104.12
98.99
77.48
77.16
76.84
F
O
N
F
f1 (ppm)

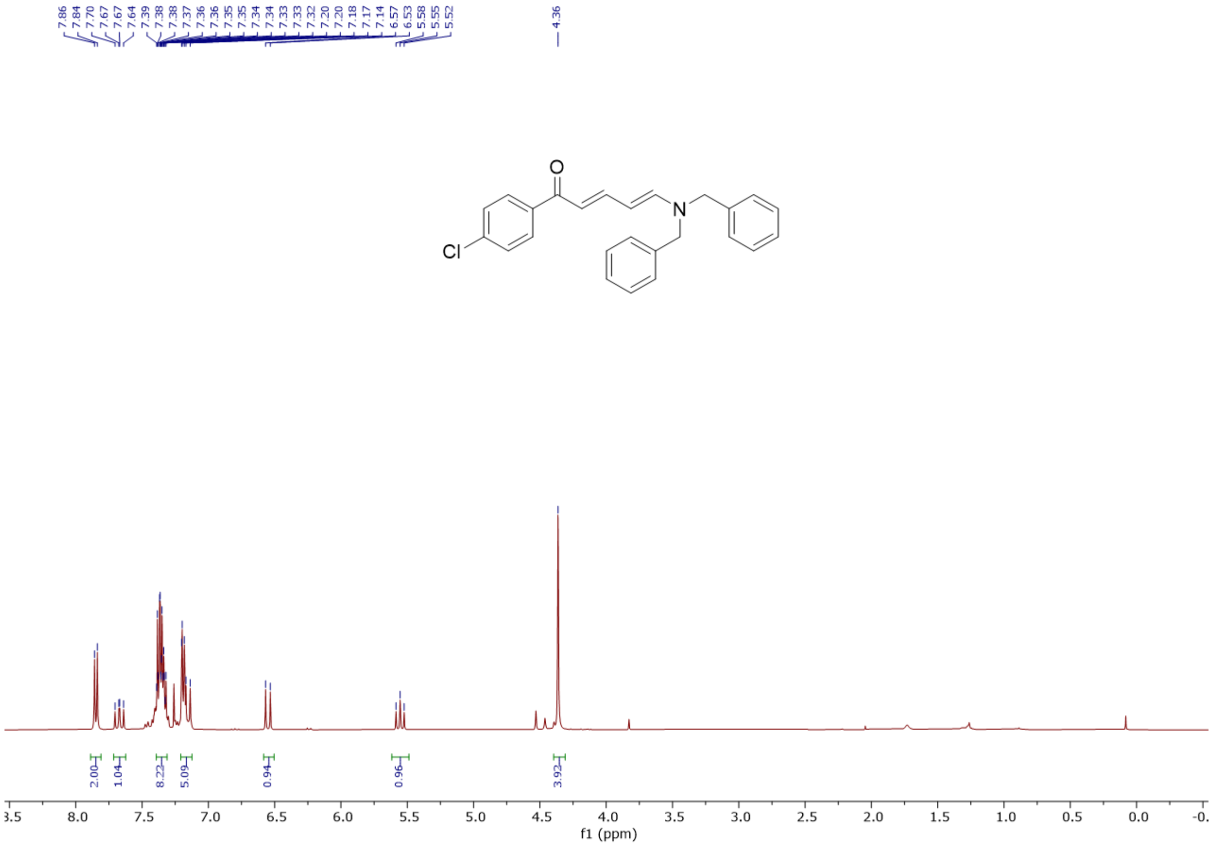
7.86
7.84
7.70
7.67
7.67
7.64
7.39
7.38
7.38
7.37
7.36
7.36
7.35
7.35
7.34
7.34
7.33
7.33
7.32
7.20
7.20
7.18
7.17
7.14
6.57
6.53
5.58
5.55
5.52
4.36
O
N
Cl
2.00
1.04
8.22
5.09
0.94
0.96
3.92
f1 (ppm)

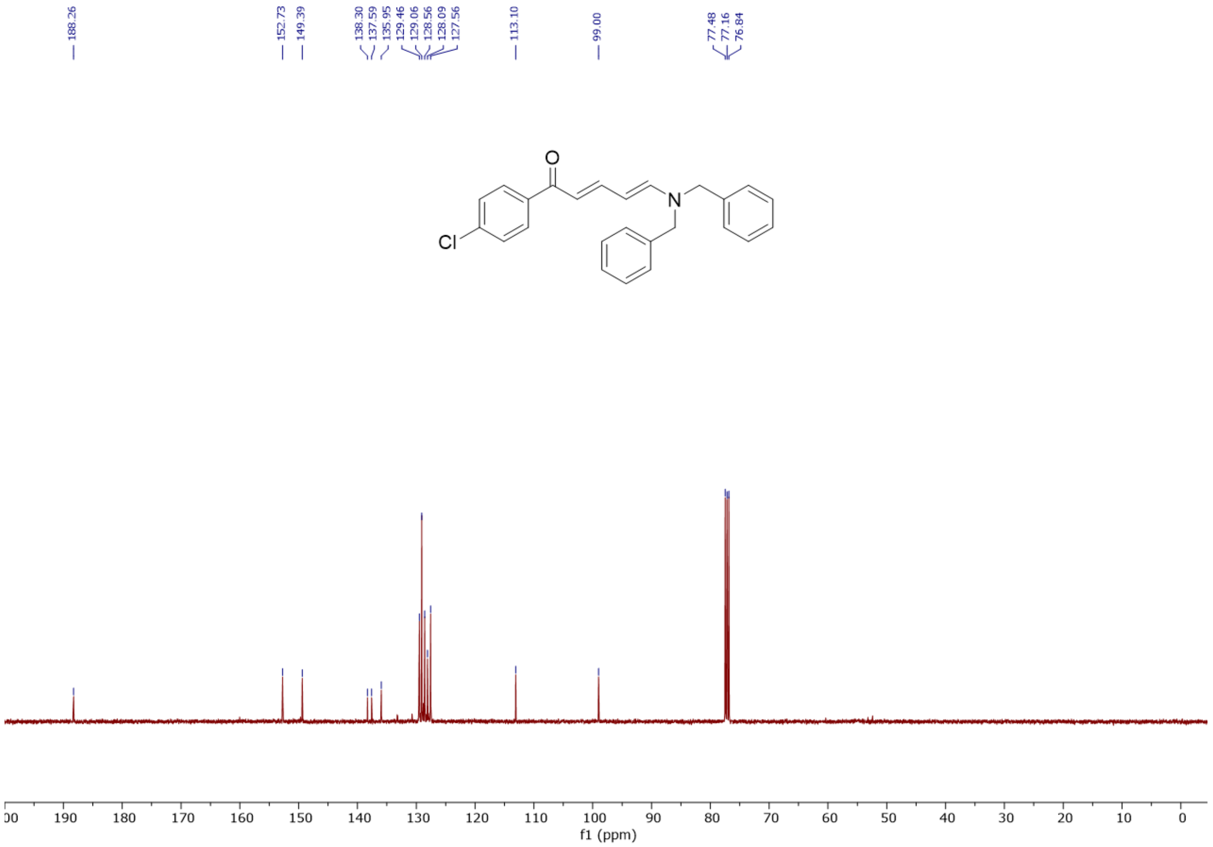
188.26
152.73
149.39
138.30
137.59
135.95
129.46
129.06
128.56
128.09
127.56
113.10
99.00
77.48
77.16
76.84
O
N
Cl
f1 (ppm)

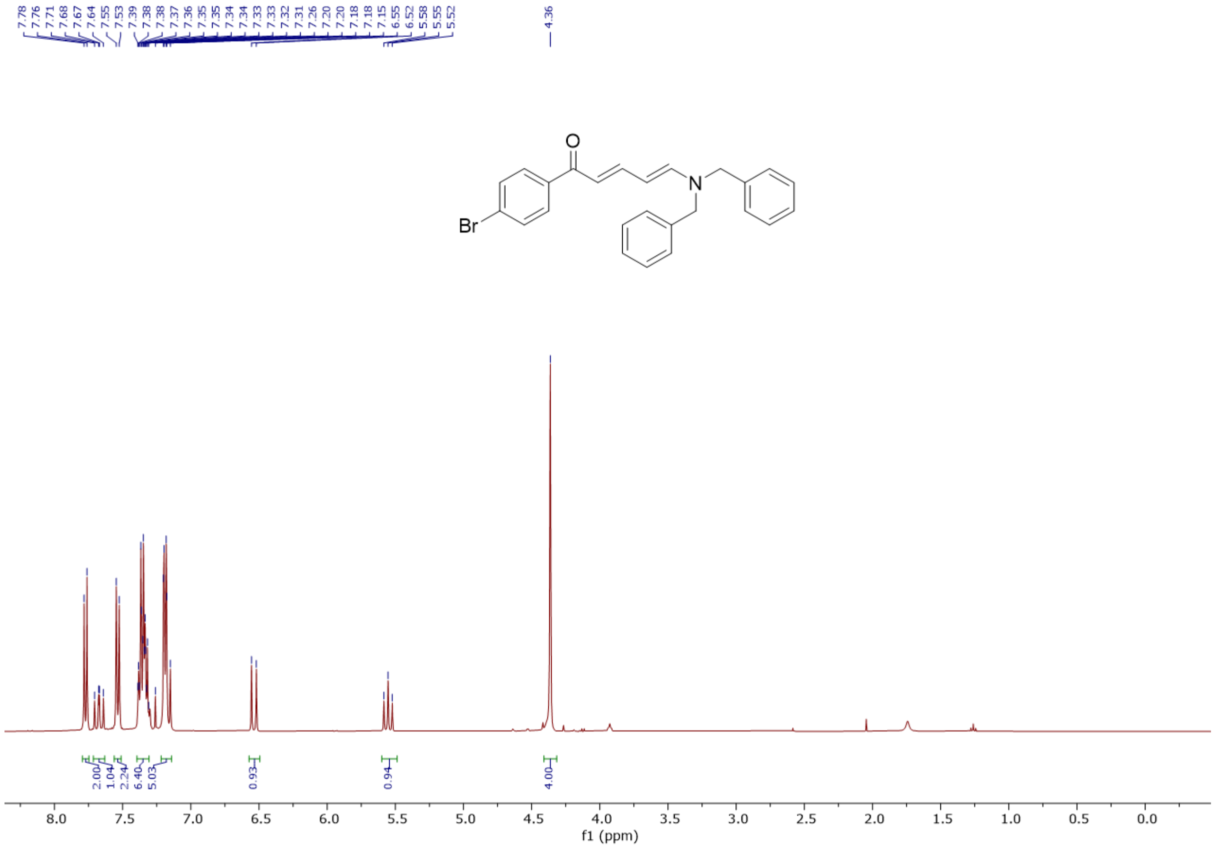
7.78
7.76
7.71
7.68
7.67
7.64
7.55
7.53
7.39
7.38
7.38
7.37
7.36
7.35
7.35
7.34
7.34
7.33
7.33
7.32
7.31
7.26
7.20
7.20
7.18
7.18
7.15
6.55
6.52
5.58
5.55
5.52
4.36
O
N
Br
2.00
1.04
2.24
6.40
5.03
0.93
0.94
4.00
f1 (ppm)

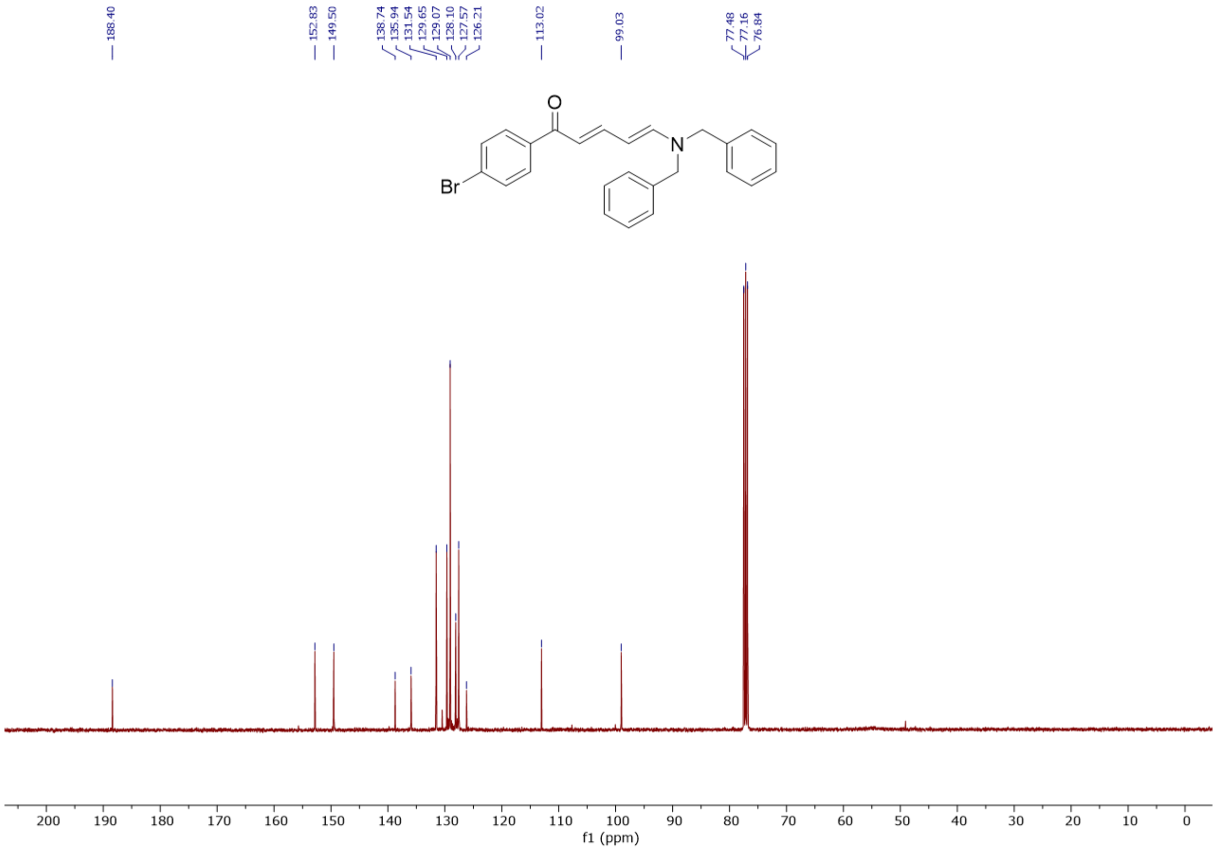
188.40
152.83
149.50
138.74
135.94
131.54
129.65
129.07
128.10
127.57
126.21
113.02
99.03
77.48
77.16
76.84
O
N
Br
f1 (ppm)

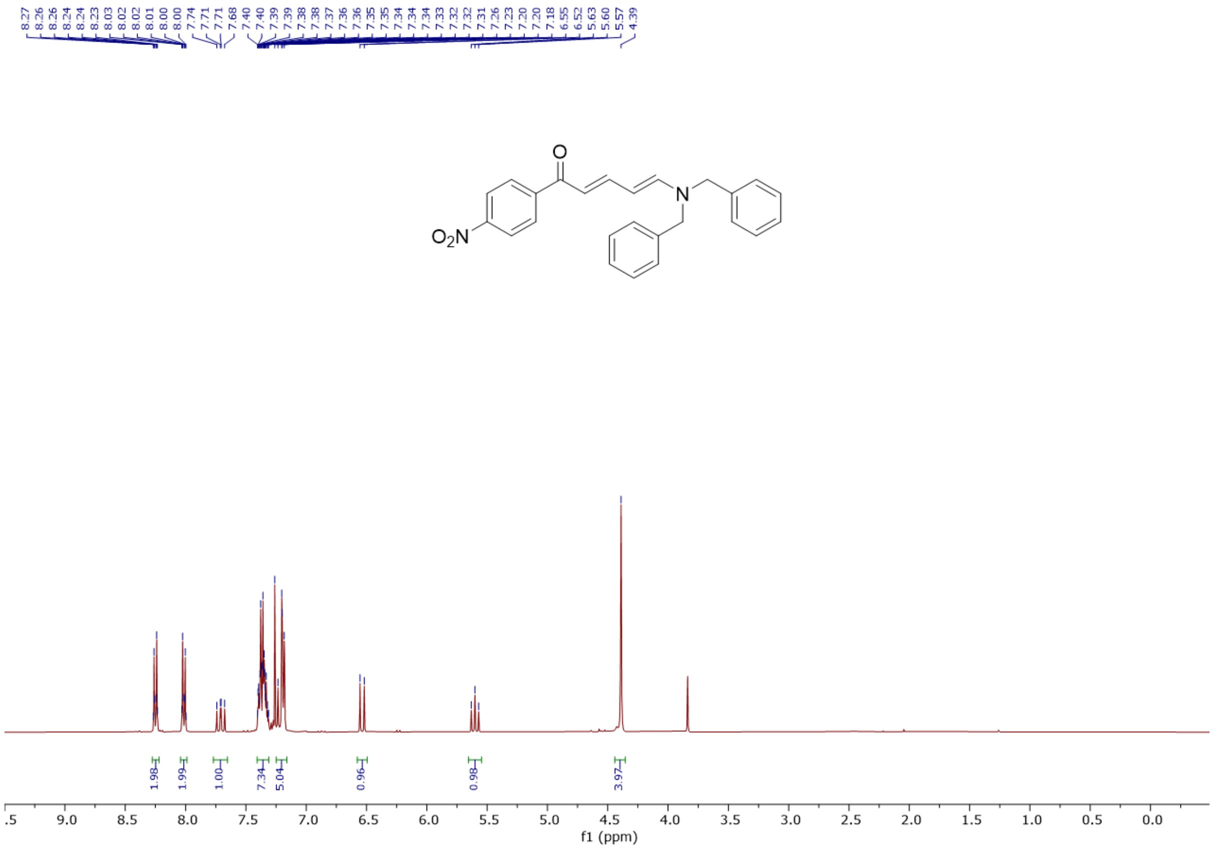
8.27 8.26 8.26 8.24 8.24 8.23 8.03 8.02 8.02 8.01 8.00 8.00 7.74 7.71 7.71 7.68 7.40 7.40 7.39 7.39 7.38 7.38 7.37 7.36 7.36 7.35 7.35 7.34 7.34 7.33 7.33 7.32 7.32 7.31 7.26 7.23 7.20 7.20 7.18 6.55 6.52 5.63 5.60 5.57 4.39
O
O2N
N
1.98
1.99
1.00
7.34
5.04
0.96
0.98
3.97
f1 (ppm)

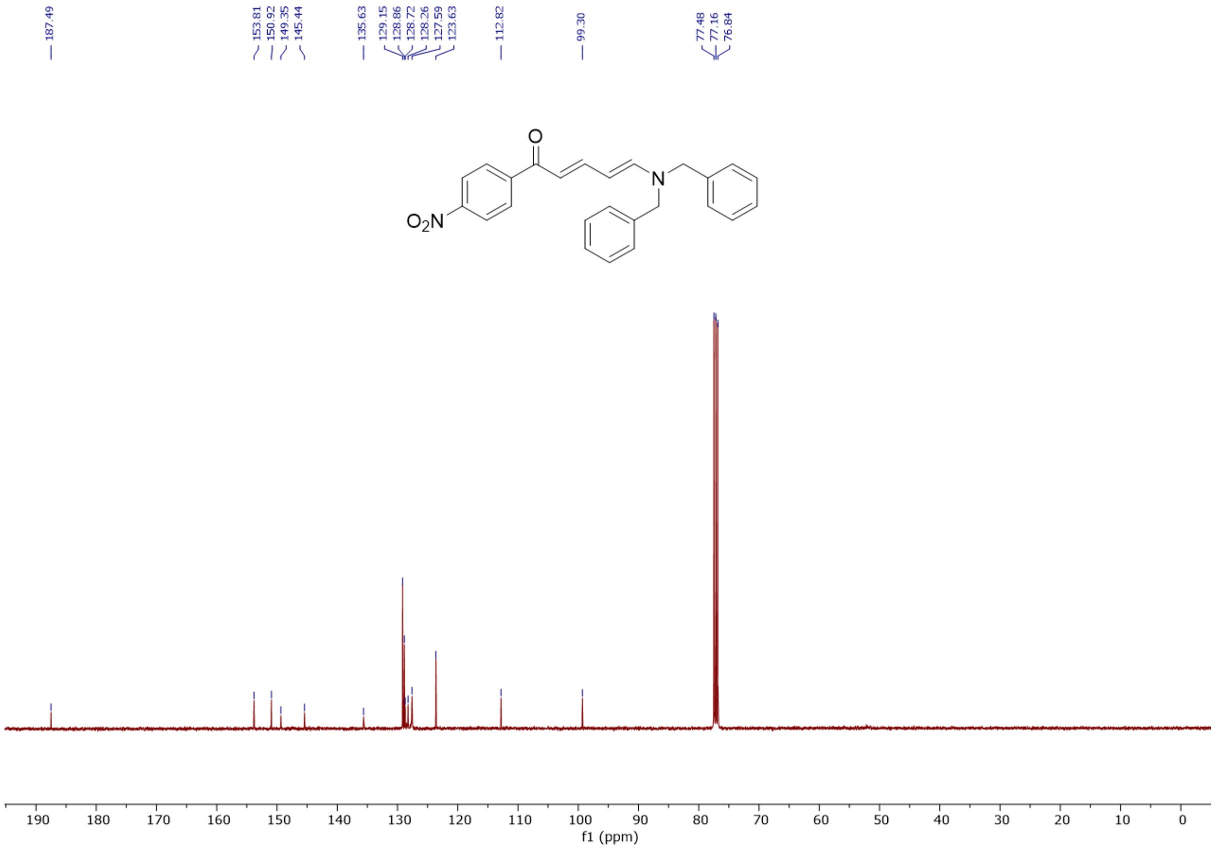

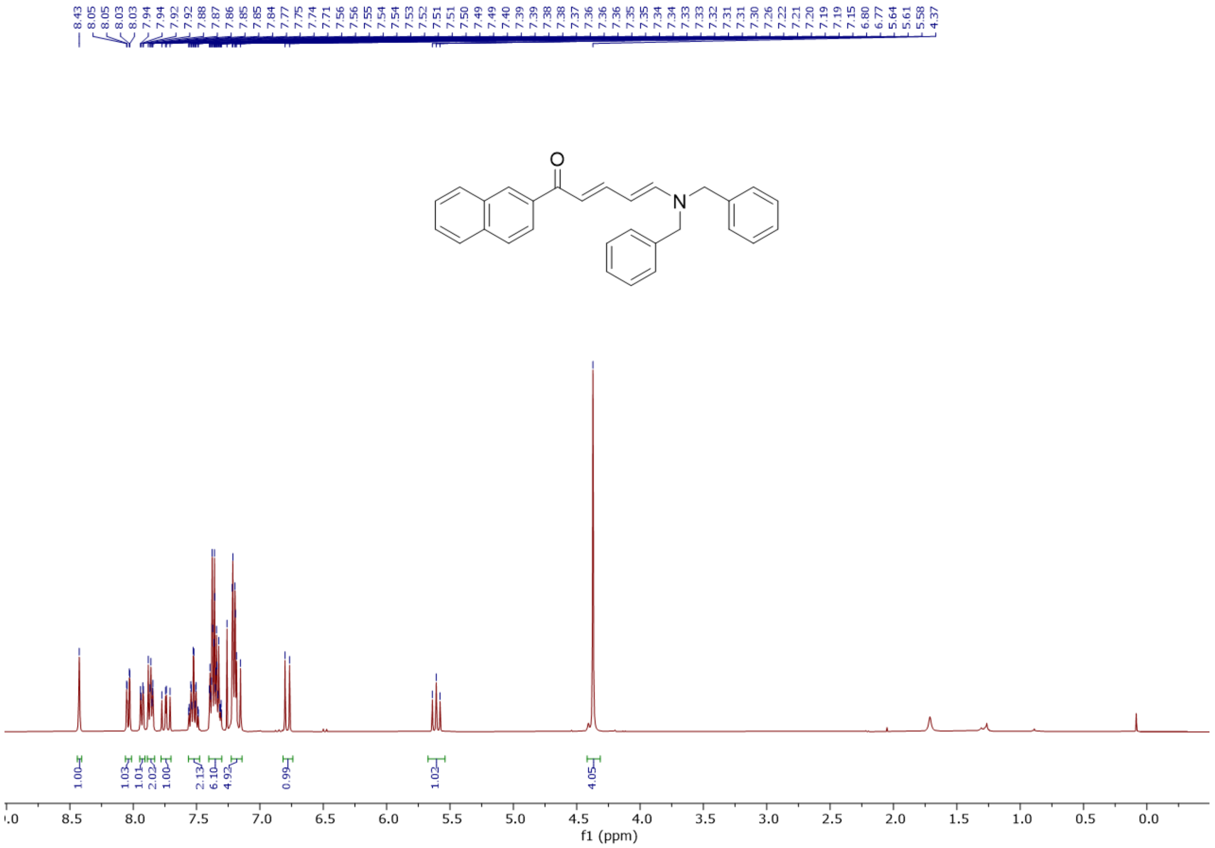

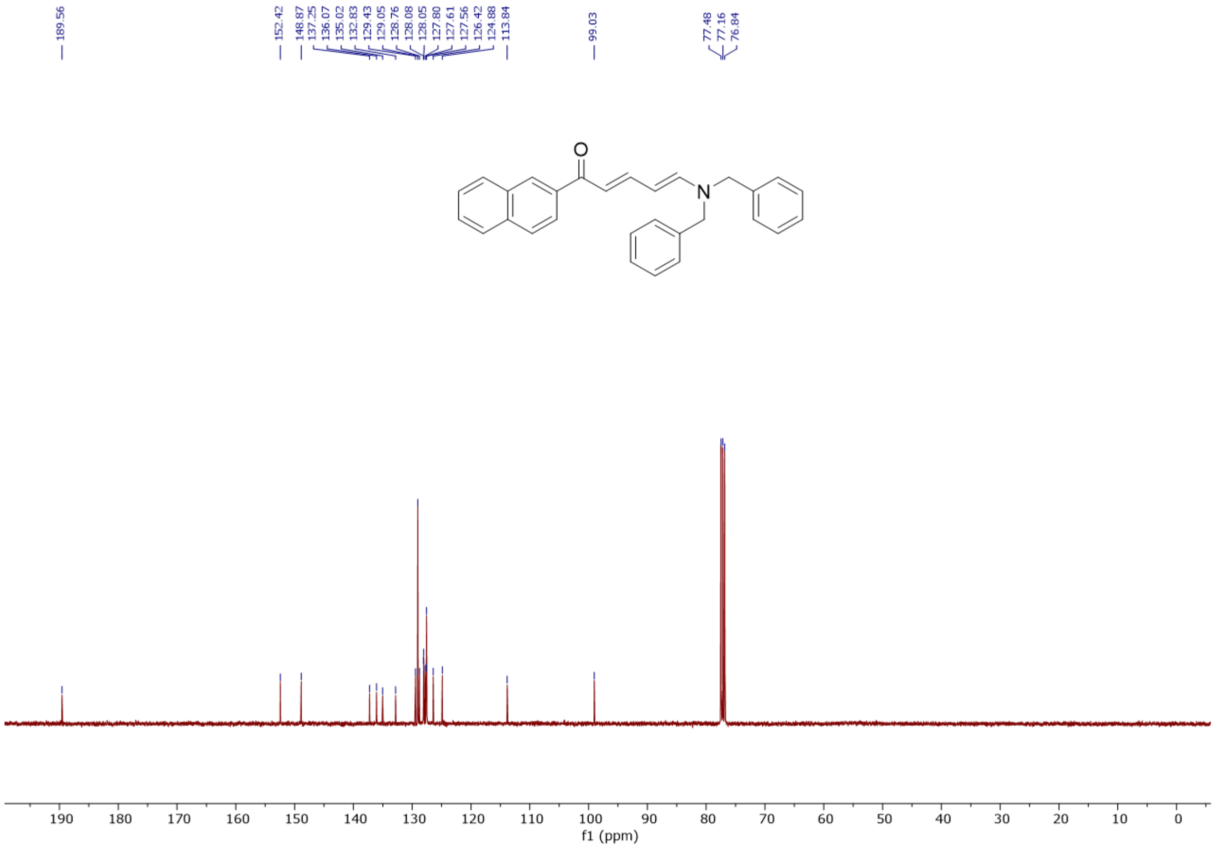

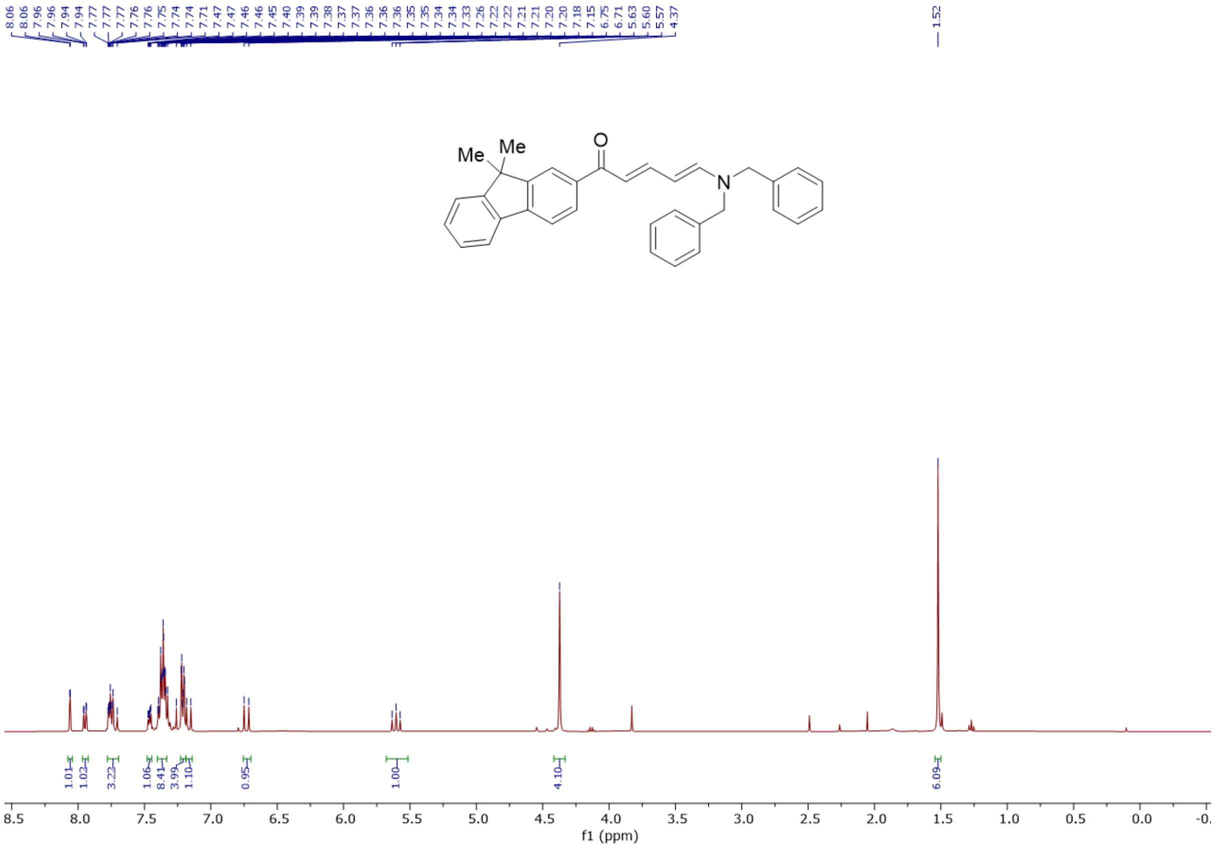

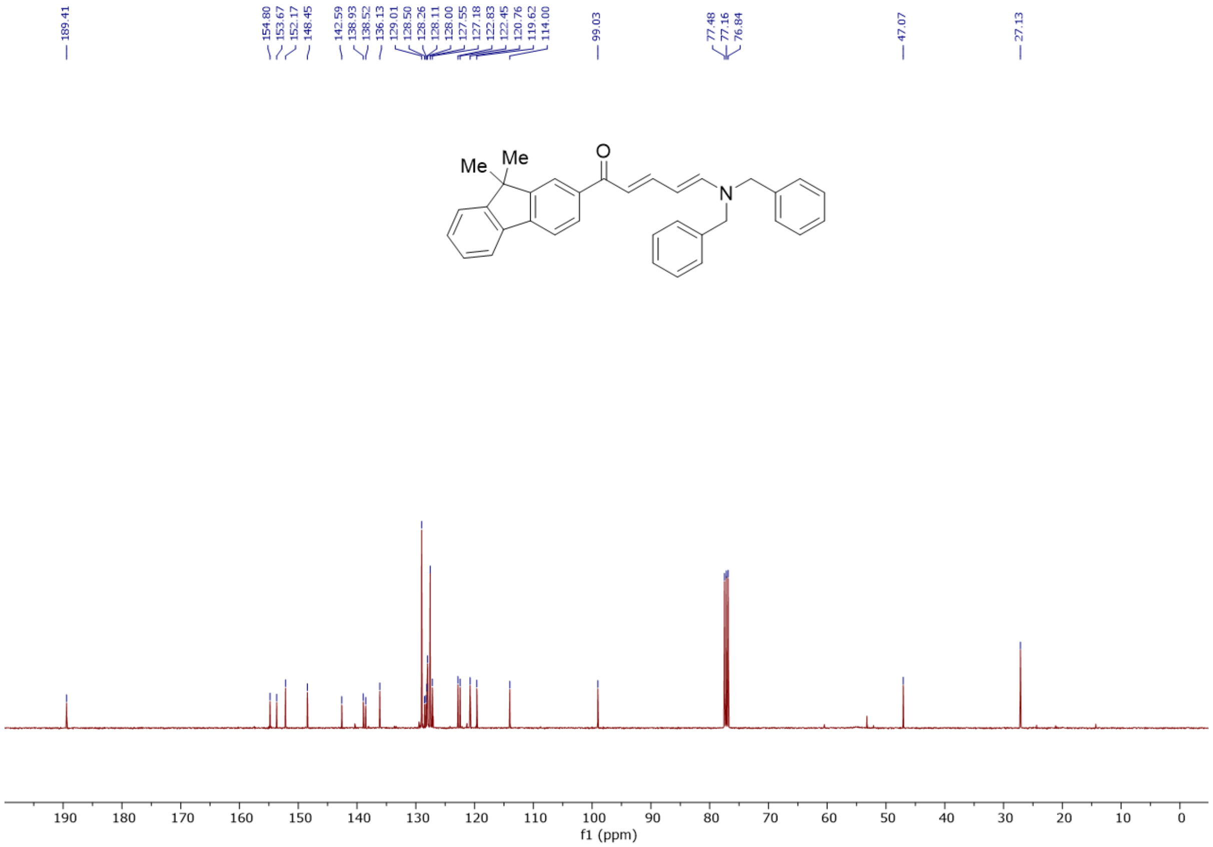
189.41
154.80
153.67
152.17
148.45
142.59
138.93
138.52
136.13
129.01
128.50
128.26
128.11
128.00
127.55
127.18
122.83
122.45
120.76
119.62
114.00
99.03
77.48
77.16
76.84
47.07
27.13
Me
Me
O
N
f1 (ppm)

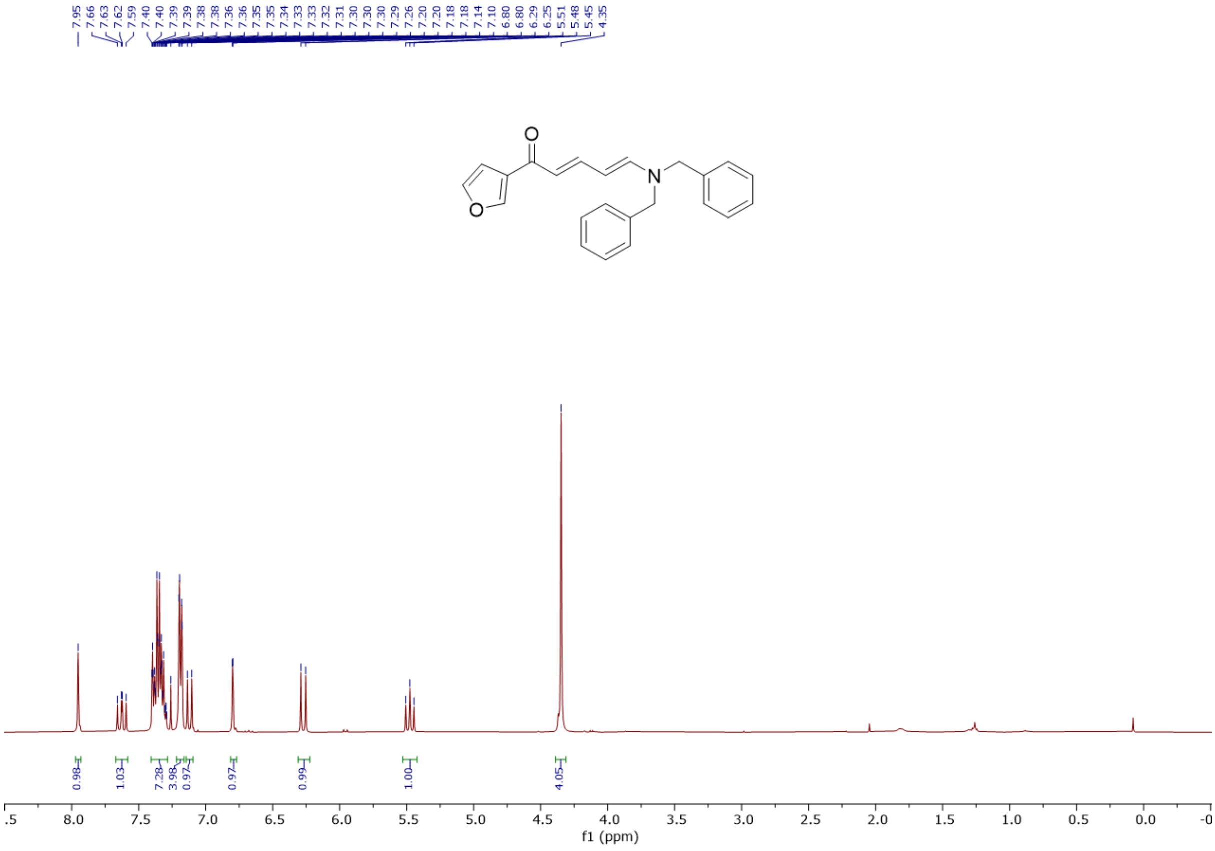
7.95
7.66
7.63
7.62
7.59
7.40
7.40
7.39
7.39
7.38
7.38
7.36
7.36
7.35
7.35
7.34
7.33
7.33
7.32
7.31
7.30
7.30
7.30
7.29
7.26
7.20
7.20
7.18
7.18
7.14
7.10
6.80
6.80
6.29
6.25
5.51
5.48
5.45
4.35
O
O
N
0.98
1.03
7.28
3.98
0.97
0.97
0.99
1.00
4.05
f1 (ppm)

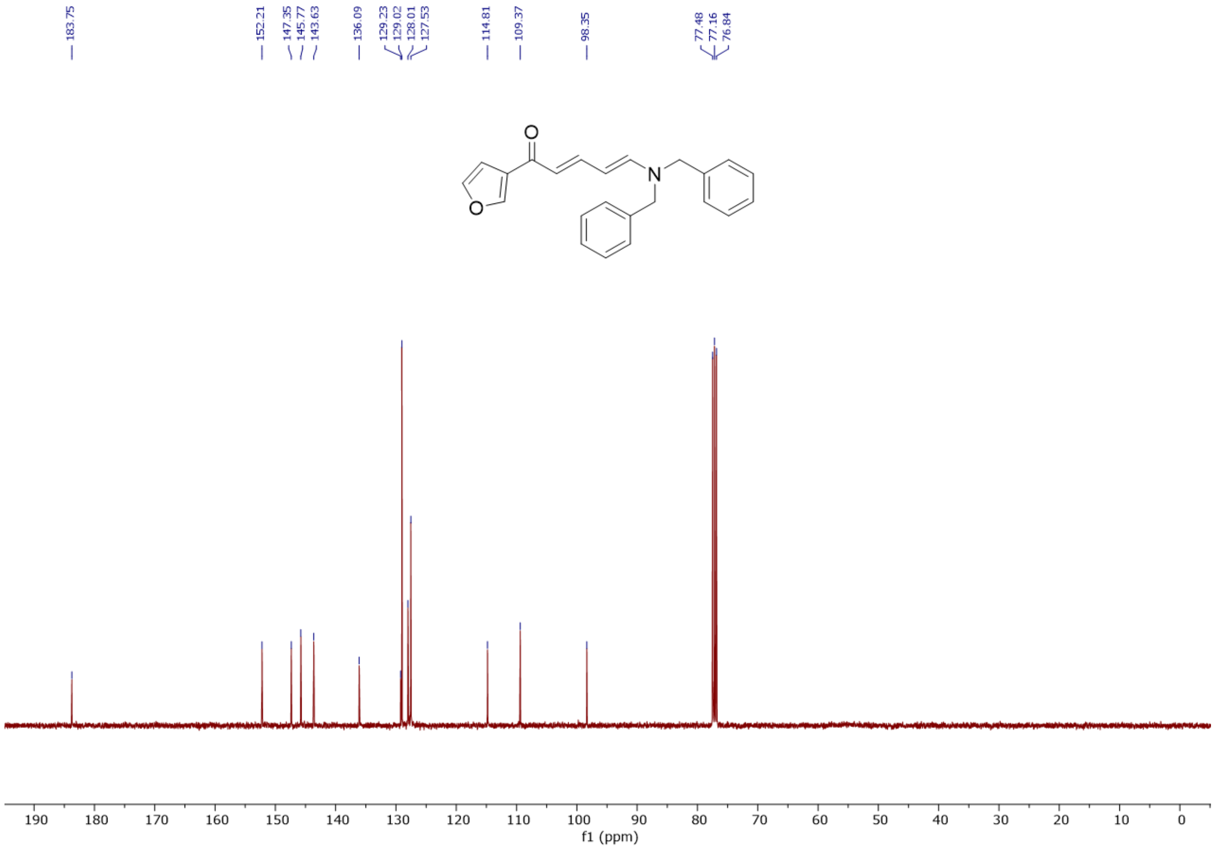

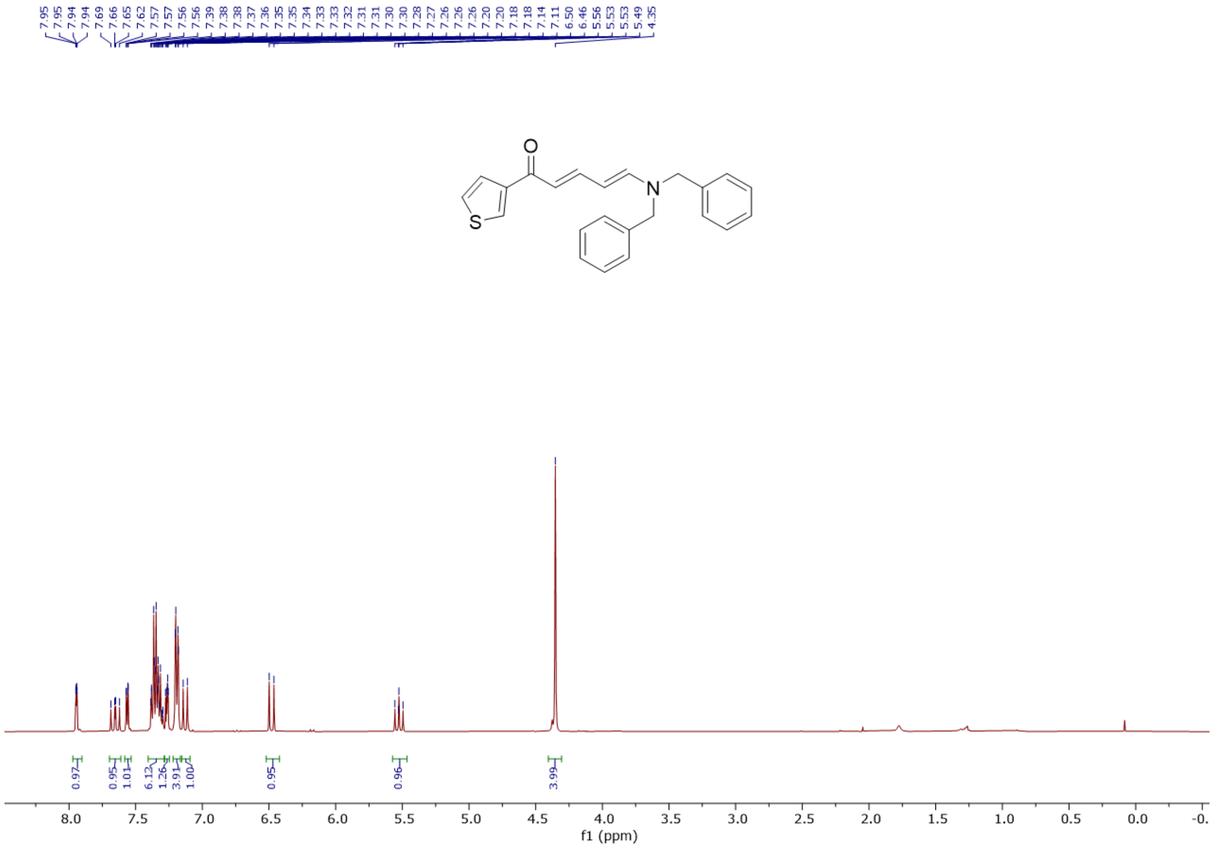

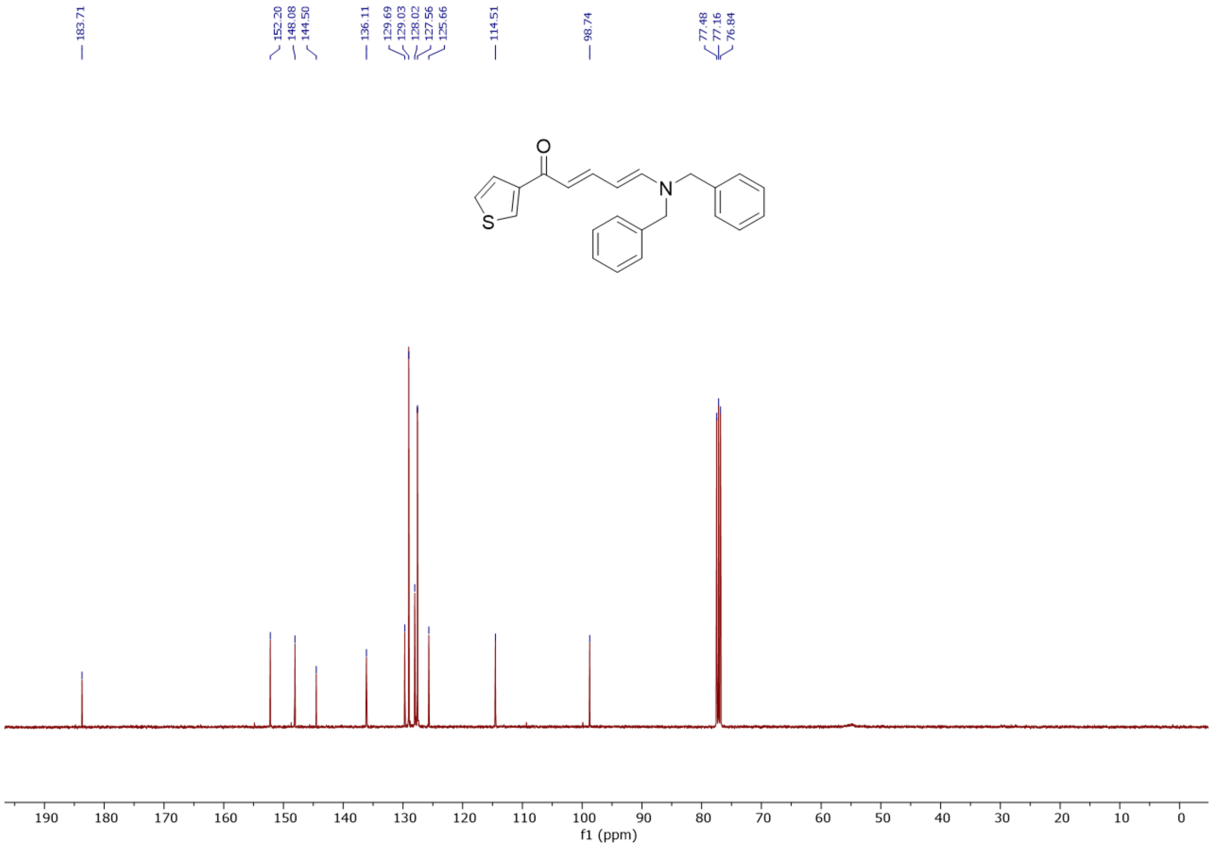
183.71
152.20
148.08
144.50
136.11
129.69
129.03
128.02
127.56
125.66
114.51
98.74
77.48
77.16
76.84
O
S
N
190 180 170 160 150 140 130 120 110 100 90 80 70 60 50 40 30 20 10 0
f1 (ppm)

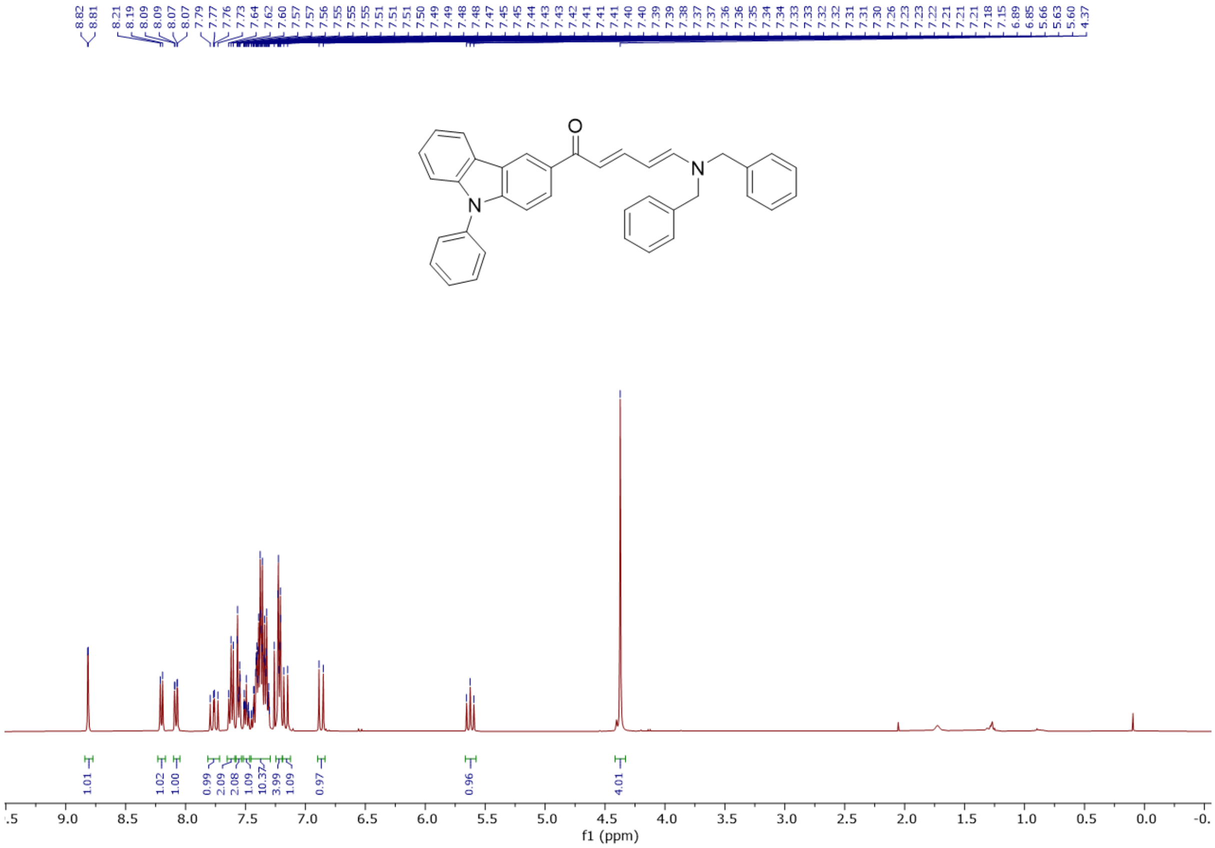
8.82 8.81 8.21 8.19 8.09 8.09 8.07 8.07 7.79 7.77 7.76 7.73 7.64 7.62 7.60 7.57 7.57 7.56 7.55 7.55 7.51 7.51 7.51 7.50 7.49 7.49 7.48 7.48 7.47 7.45 7.45 7.44 7.43 7.43 7.42 7.41 7.41 7.40 7.40 7.39 7.39 7.38 7.37 7.37 7.36 7.36 7.35 7.34 7.34 7.33 7.33 7.32 7.32 7.31 7.31 7.30 7.26 7.23 7.23 7.22 7.21 7.21 7.21 7.18 7.15 6.89 6.85 5.66 5.63 5.60 4.37
O
N
N
1.01
1.02
1.00
0.99
2.09
2.08
1.09
10.37
3.99
1.09
0.97
0.96
4.01
.5 9.0 8.5 8.0 7.5 7.0 6.5 6.0 5.5 5.0 4.5 4.0 3.5 3.0 2.5 2.0 1.5 1.0 0.5 0.0 -0.
f1 (ppm)

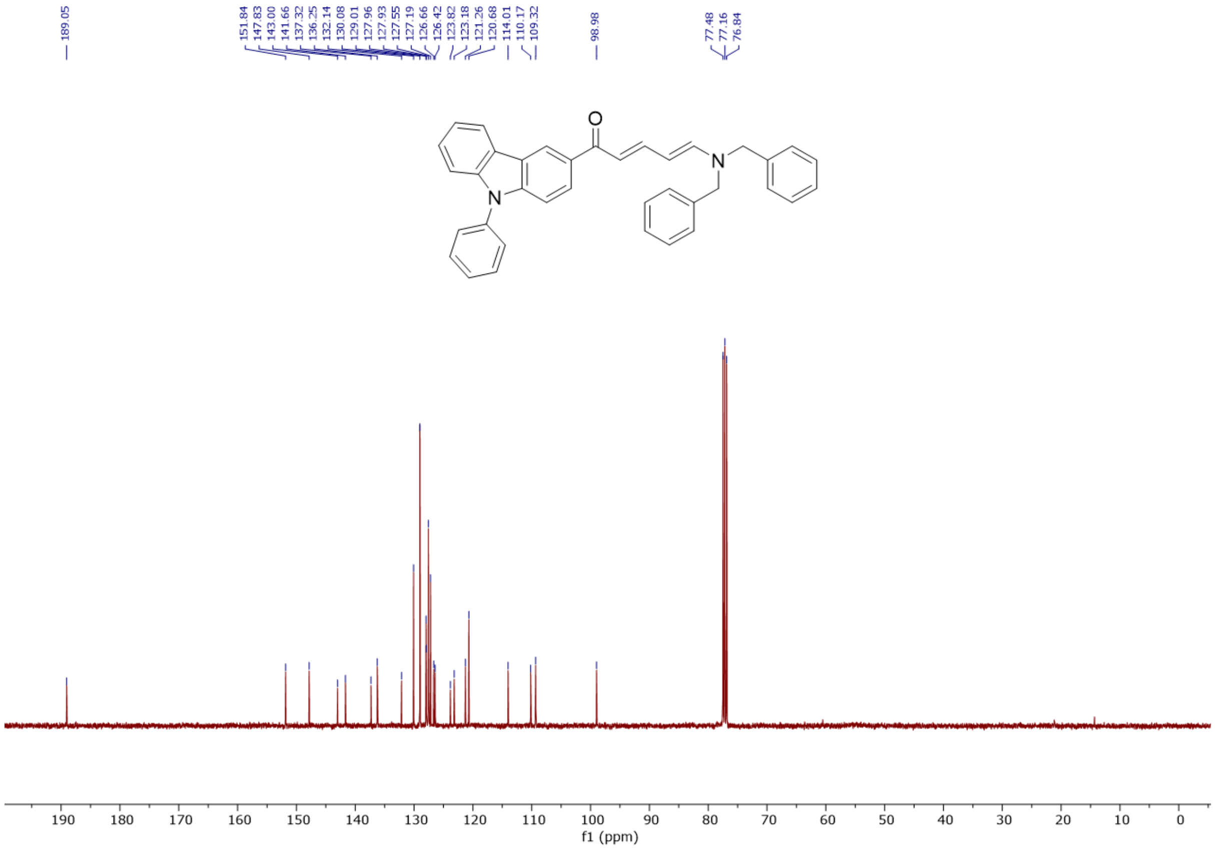
189.05
151.84 147.83 143.00 141.66 137.32 136.25 132.14 130.08 129.01 127.96 127.93 127.55 127.19 126.66 126.42 123.82 123.18 121.26 120.68 114.01 110.17 109.32
98.98
77.48 77.16 76.84
O
N
N
f1 (ppm)

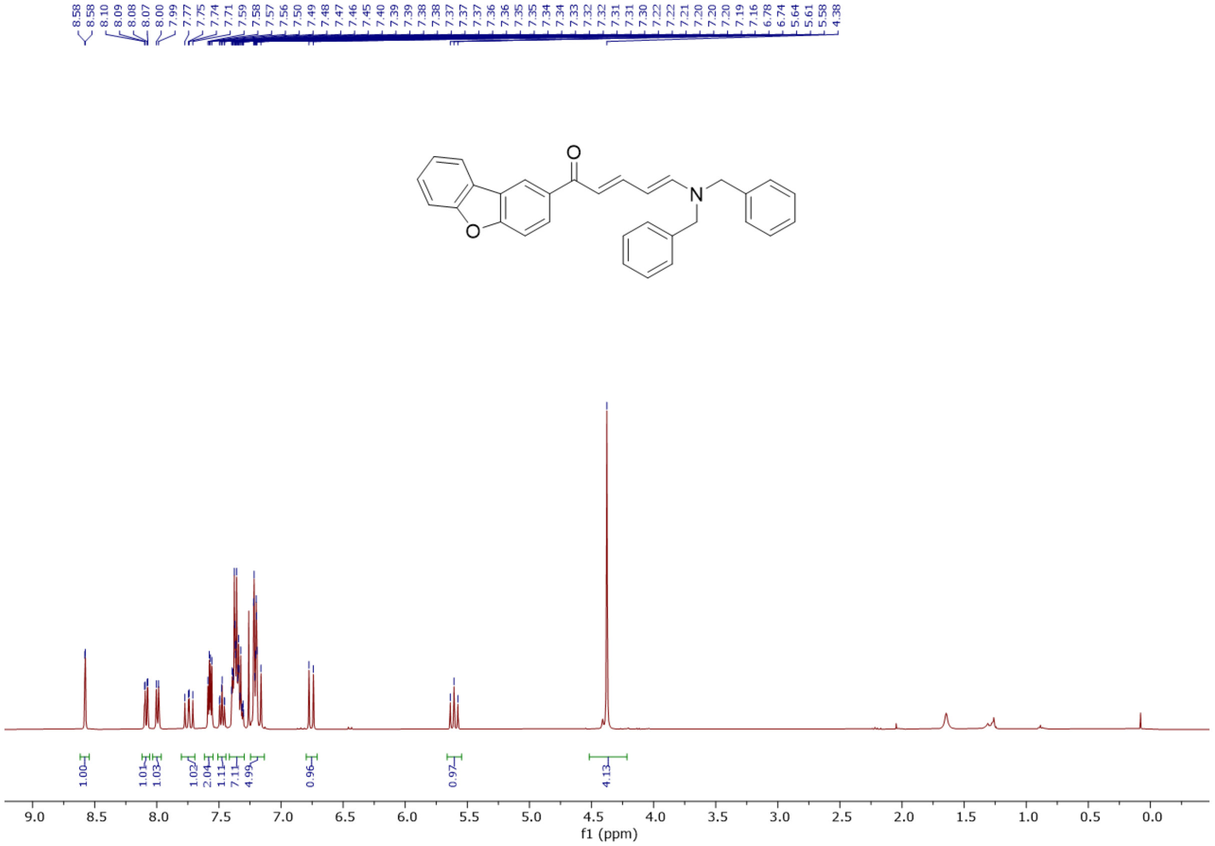
8.58 8.58 8.10 8.09 8.08 8.07 8.00 7.99 7.77 7.75 7.74 7.71 7.59 7.58 7.57 7.56 7.50 7.49 7.48 7.47 7.46 7.45 7.40 7.39 7.39 7.38 7.38 7.37 7.37 7.37 7.36 7.35 7.35 7.34 7.33 7.32 7.32 7.31 7.31 7.30 7.22 7.22 7.21 7.20 7.20 7.20 7.19 7.16 6.78 6.74 5.64 5.61 5.58 4.38
O
N
O
1.00 1.01 1.03 1.02 2.04 1.11 7.11 4.99 0.96 0.97 4.13
f1 (ppm)

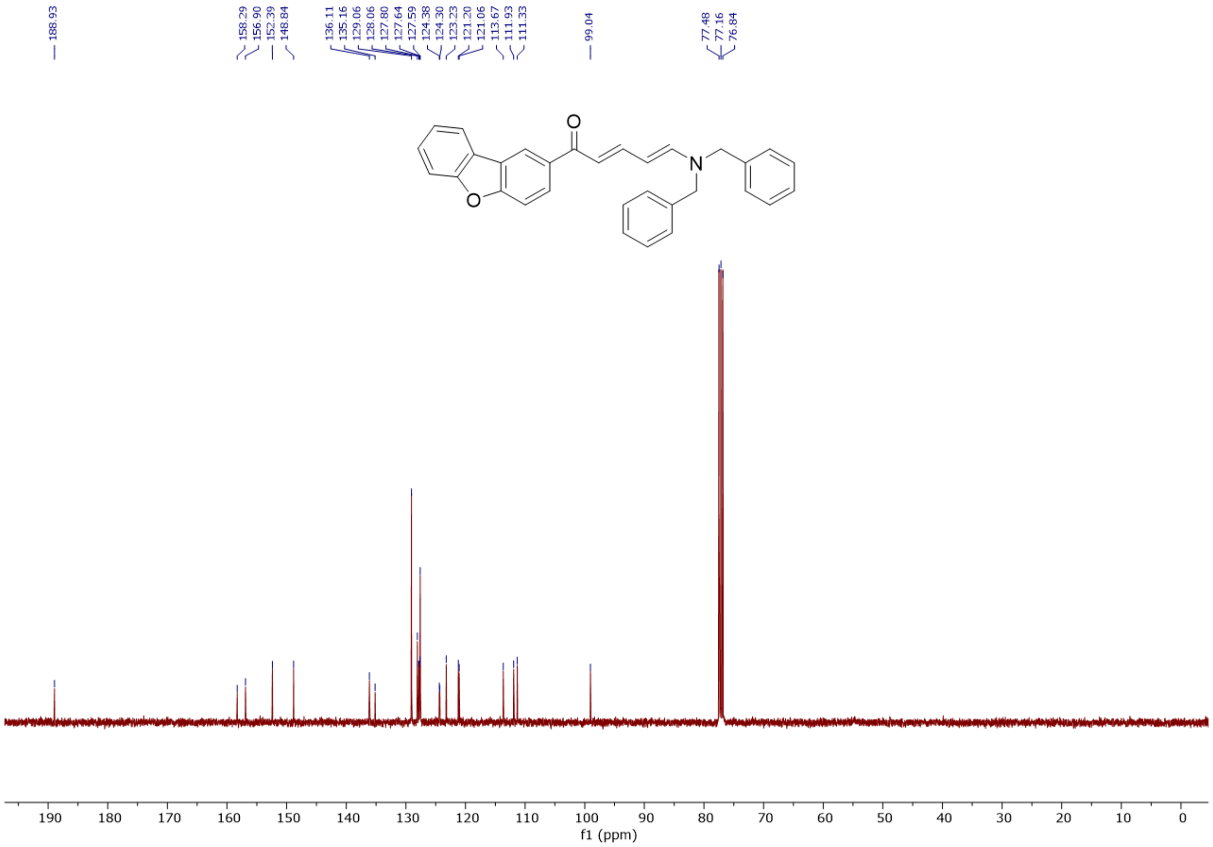
188.93
158.29
156.90
152.39
148.84
136.11
135.16
129.06
128.06
127.80
127.64
127.59
124.38
124.30
123.23
121.20
121.06
113.67
111.93
111.33
99.04
77.48
77.16
76.84
O
N
f1 (ppm)

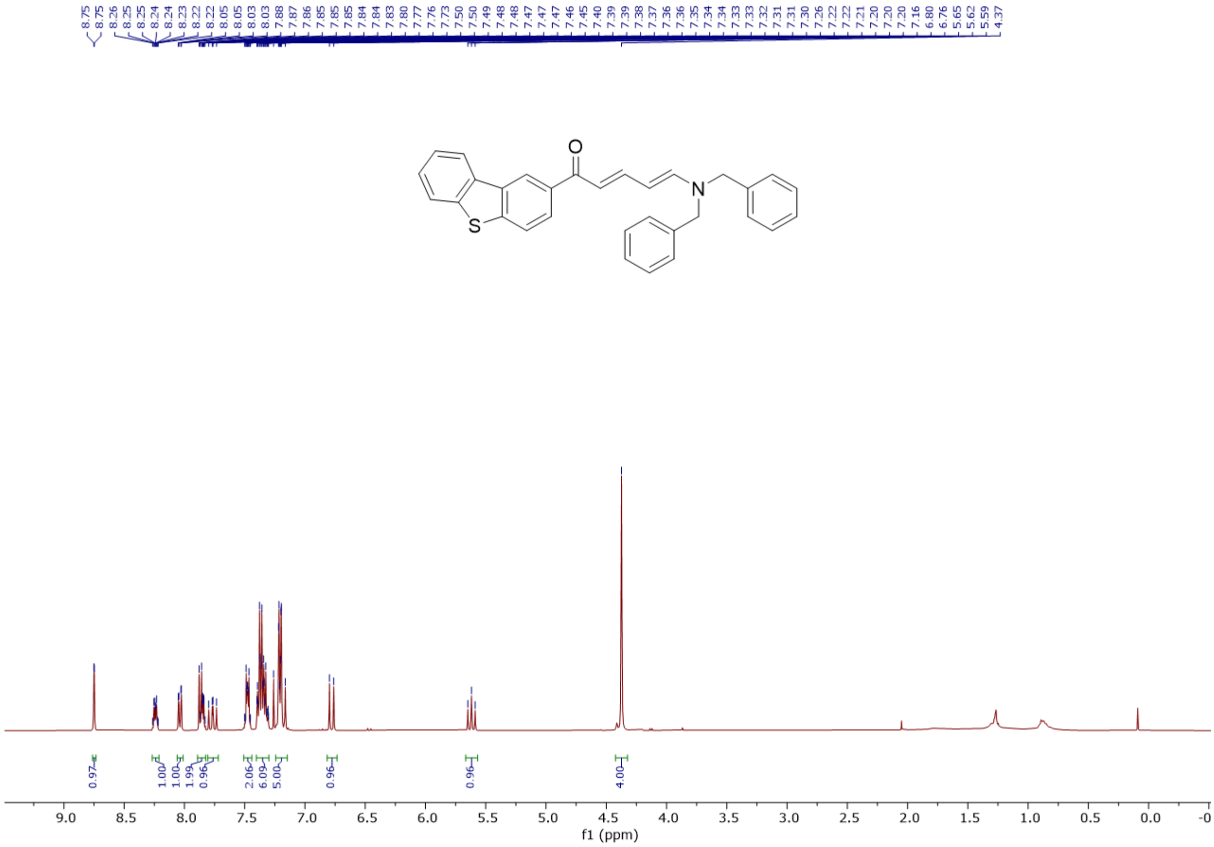
8.75 8.75 8.26 8.25 8.25 8.24 8.24 8.23 8.22 8.22 8.05 8.05 8.03 8.03 7.88 7.87 7.86 7.85 7.85 7.84 7.83 7.80 7.77 7.76 7.73 7.50 7.50 7.49 7.48 7.48 7.47 7.47 7.46 7.45 7.40 7.39 7.39 7.38 7.37 7.36 7.36 7.35 7.34 7.34 7.33 7.33 7.32 7.31 7.31 7.30 7.26 7.22 7.22 7.21 7.20 7.20 7.20 7.16 6.80 6.76 5.65 5.62 5.59 4.37
O
N
S
0.97
1.00
1.00
1.99
0.96
2.06
6.09
5.00
0.96
0.96
4.00
f1 (ppm)

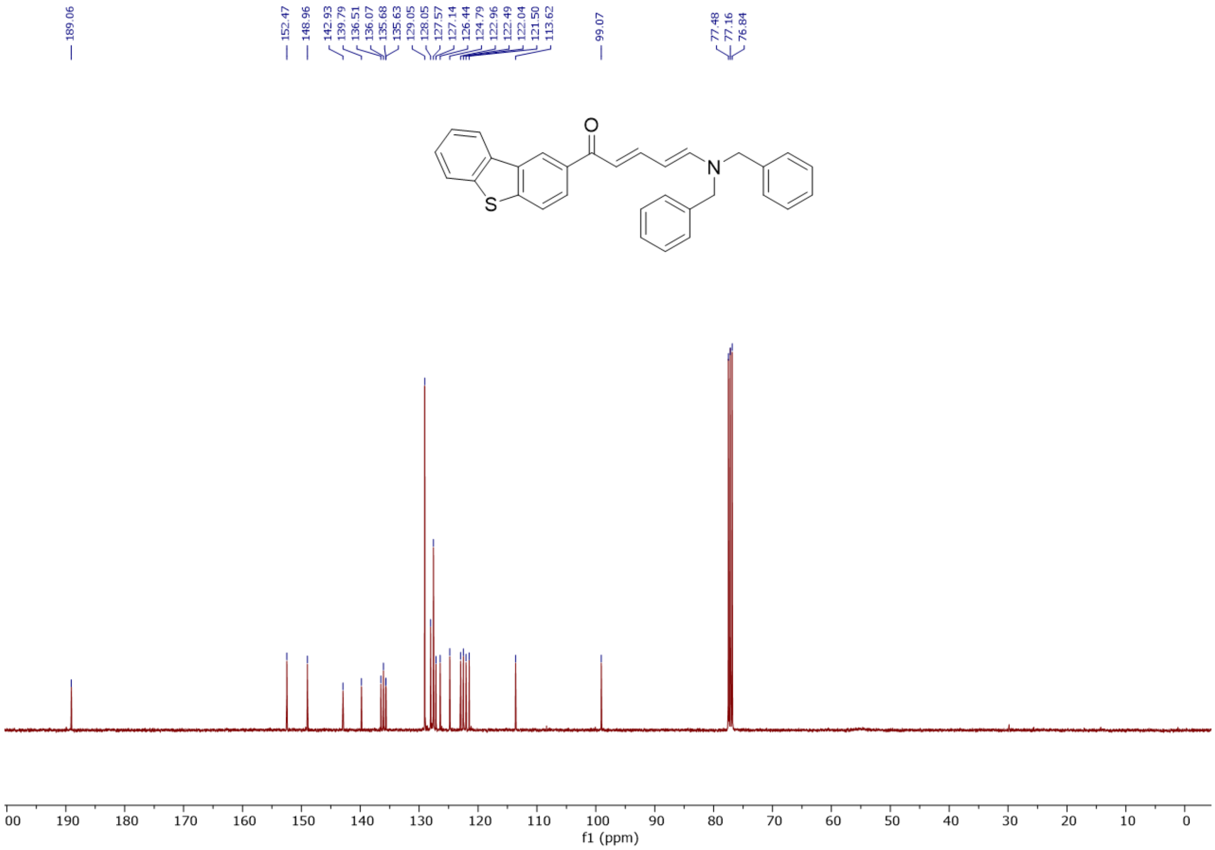
189.06
152.47
148.96
142.93
139.79
136.51
136.07
135.68
135.63
129.05
128.05
127.57
127.14
126.44
124.79
122.96
122.49
122.04
121.50
113.62
99.07
77.48
77.16
76.84
O
N
S
f1 (ppm)

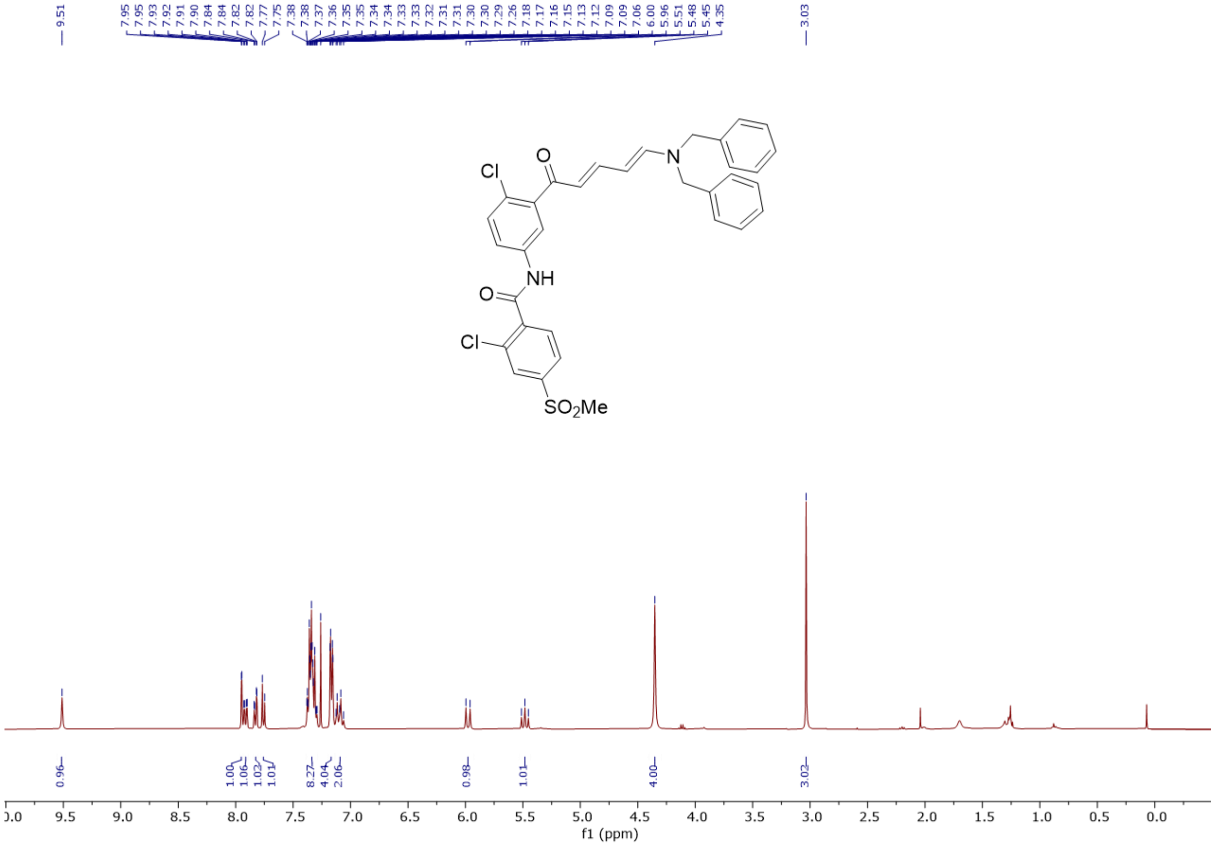
9.51
7.95 7.95 7.93 7.92 7.91 7.90 7.84 7.84 7.82 7.82 7.77 7.75 7.38 7.38 7.37 7.36 7.35 7.35 7.34 7.34 7.33 7.33 7.32 7.31 7.31 7.30 7.30 7.29 7.26 7.18 7.17 7.16 7.15 7.13 7.12 7.09 7.09 7.06 6.00 5.96 5.51 5.48 5.45 4.35
3.03
O
Cl
N
NH
O
Cl
$SO_2Me$
0.96
1.00
1.06
1.02
1.01
8.27
4.04
2.06
0.98
1.01
4.00
3.02
f1 (ppm)

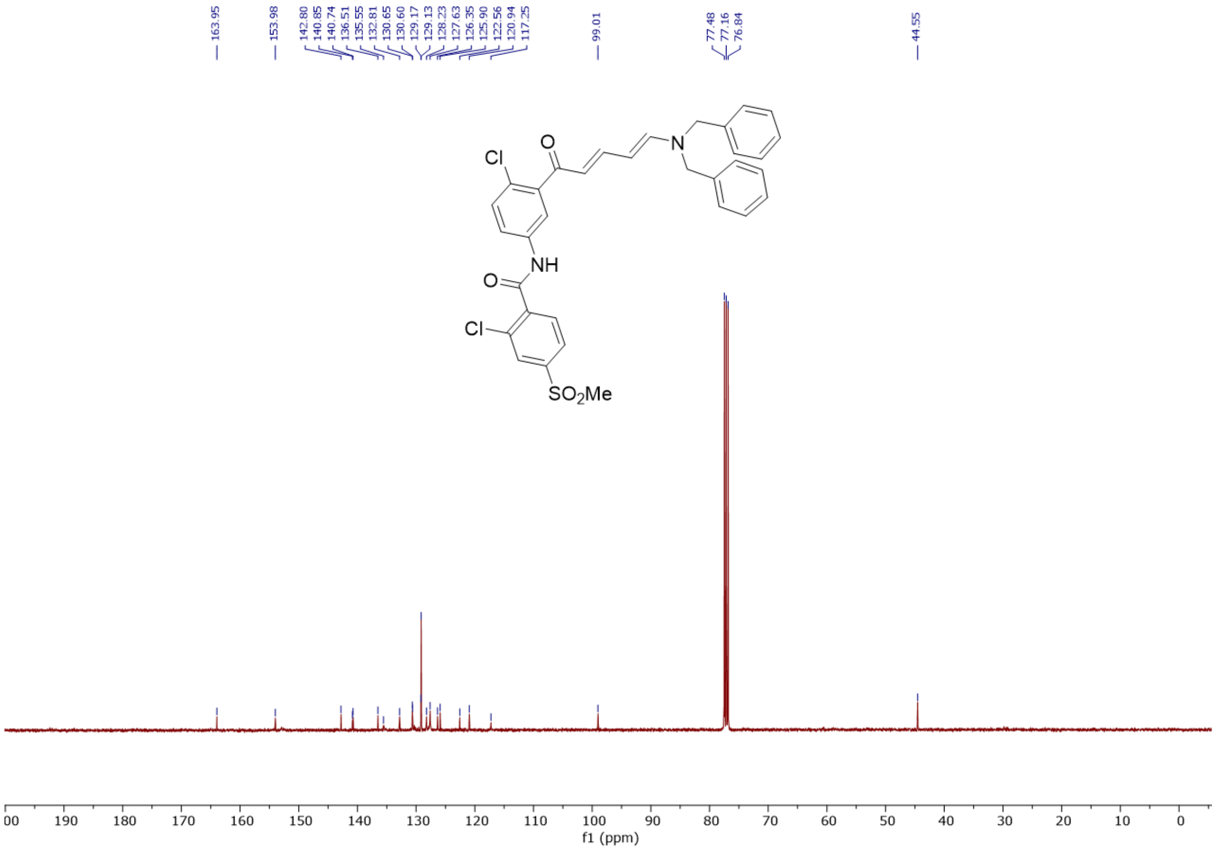
163.95
153.98
142.80
140.85
140.74
136.51
135.55
132.81
130.65
130.60
129.17
129.13
128.23
127.63
126.35
125.90
122.56
120.94
117.25
99.01
77.48
77.16
76.84
44.55
Cl
O
N
NH
O
Cl
$SO_2Me$
f1 (ppm)

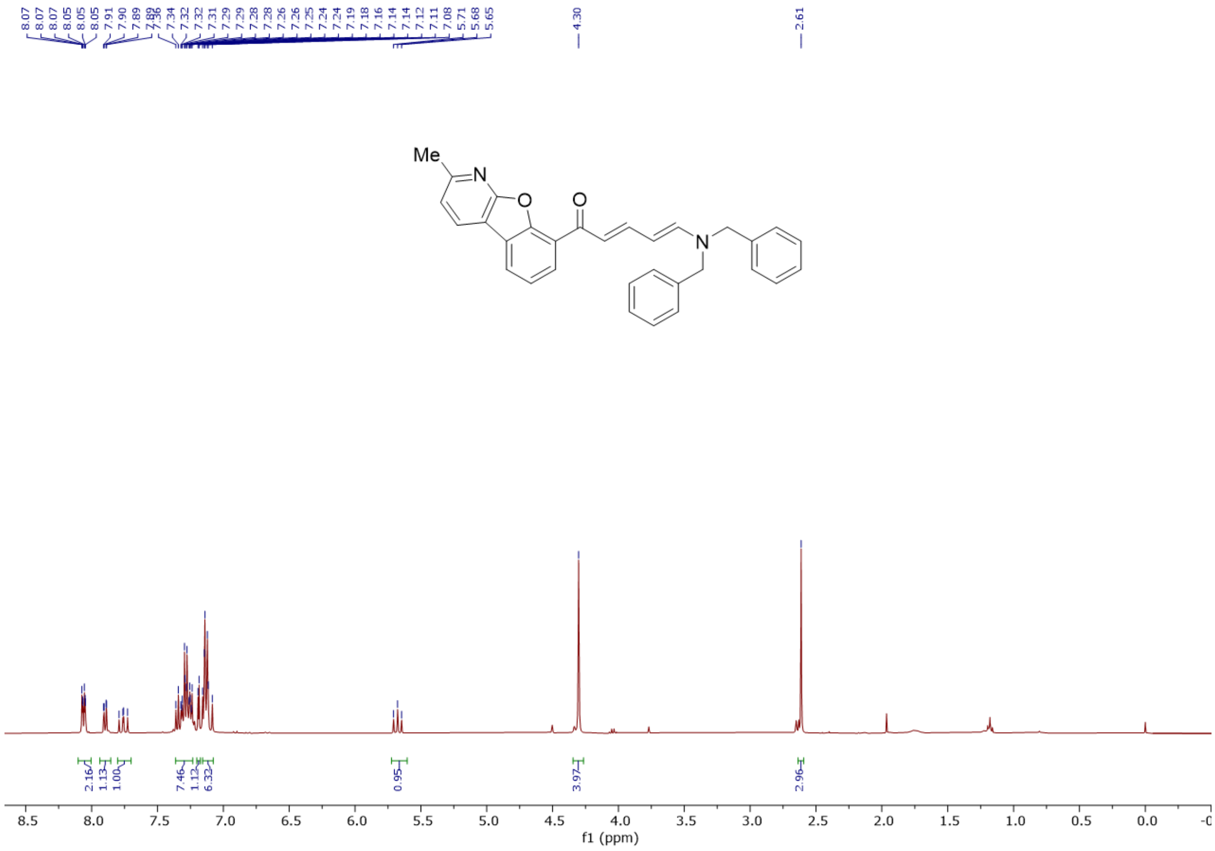
8.07
8.07
8.07
8.05
8.05
8.05
7.91
7.90
7.89
7.34
7.32
7.32
7.31
7.29
7.29
7.28
7.28
7.26
7.26
7.25
7.24
7.24
7.19
7.18
7.16
7.14
7.14
7.12
7.11
7.08
5.71
5.68
5.65
4.30
2.61
Me
N
O
O
N
2.16
1.13
1.00
7.46
1.12
6.32
0.95
3.97
2.96
f1 (ppm)

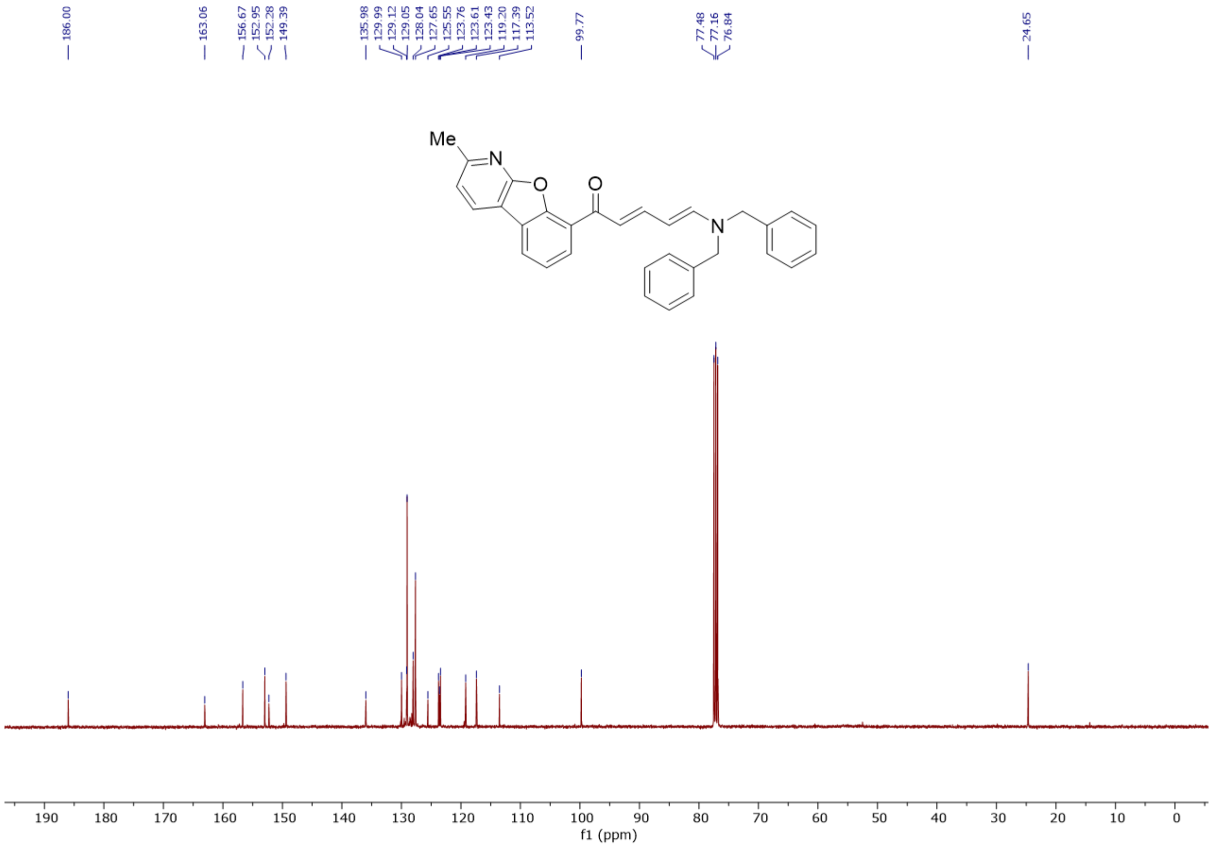
186.00
163.06
156.67
152.95
152.28
149.39
135.98
129.99
129.12
129.05
128.04
127.65
125.55
123.76
123.61
123.43
119.20
117.39
113.52
99.77
77.48
77.16
76.84
24.65
Me
N
O
f1 (ppm)

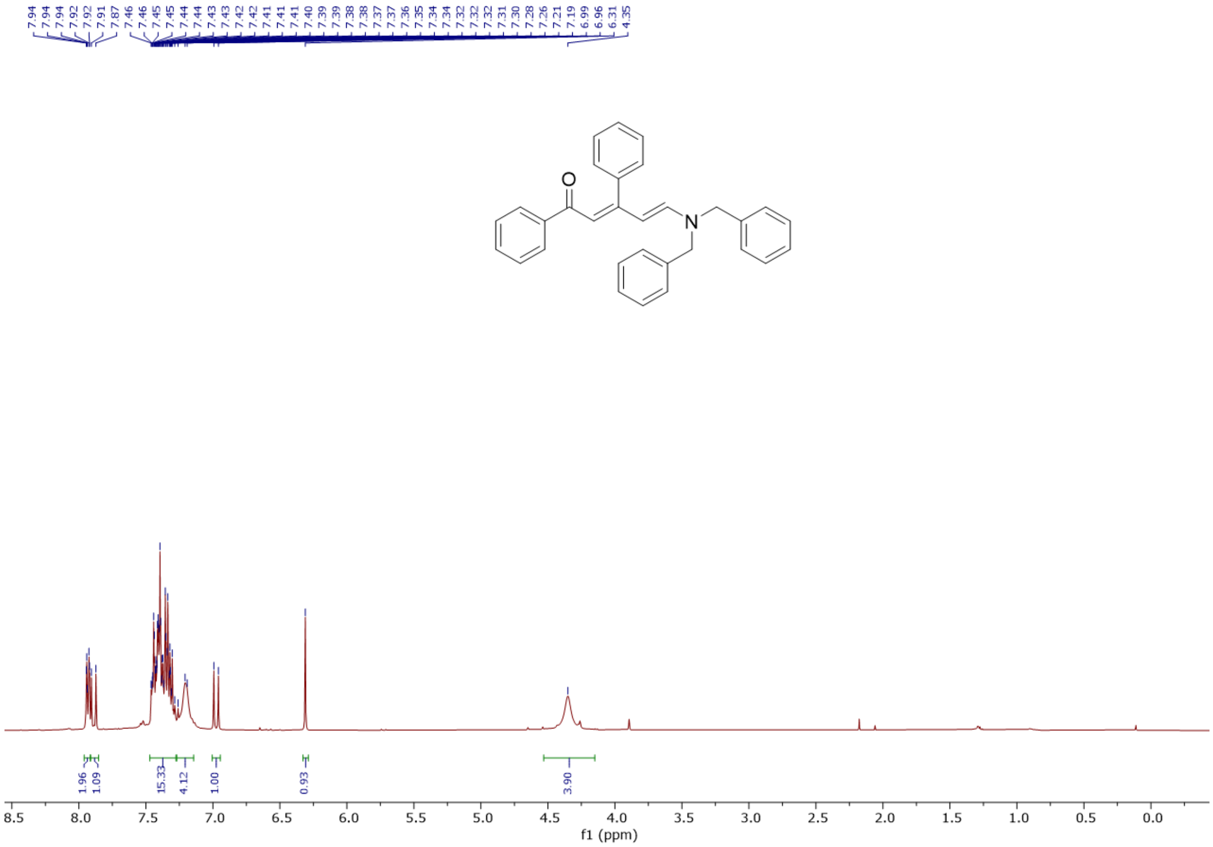
7.94
7.94
7.94
7.92
7.92
7.91
7.87
7.46
7.46
7.45
7.45
7.44
7.44
7.43
7.43
7.42
7.42
7.41
7.41
7.41
7.40
7.39
7.39
7.38
7.38
7.37
7.37
7.36
7.35
7.34
7.34
7.32
7.32
7.32
7.31
7.30
7.28
7.26
7.21
7.19
6.99
6.96
6.31
4.35
O
N
1.96
1.09
15.33
4.12
1.00
0.93
3.90
f1 (ppm)

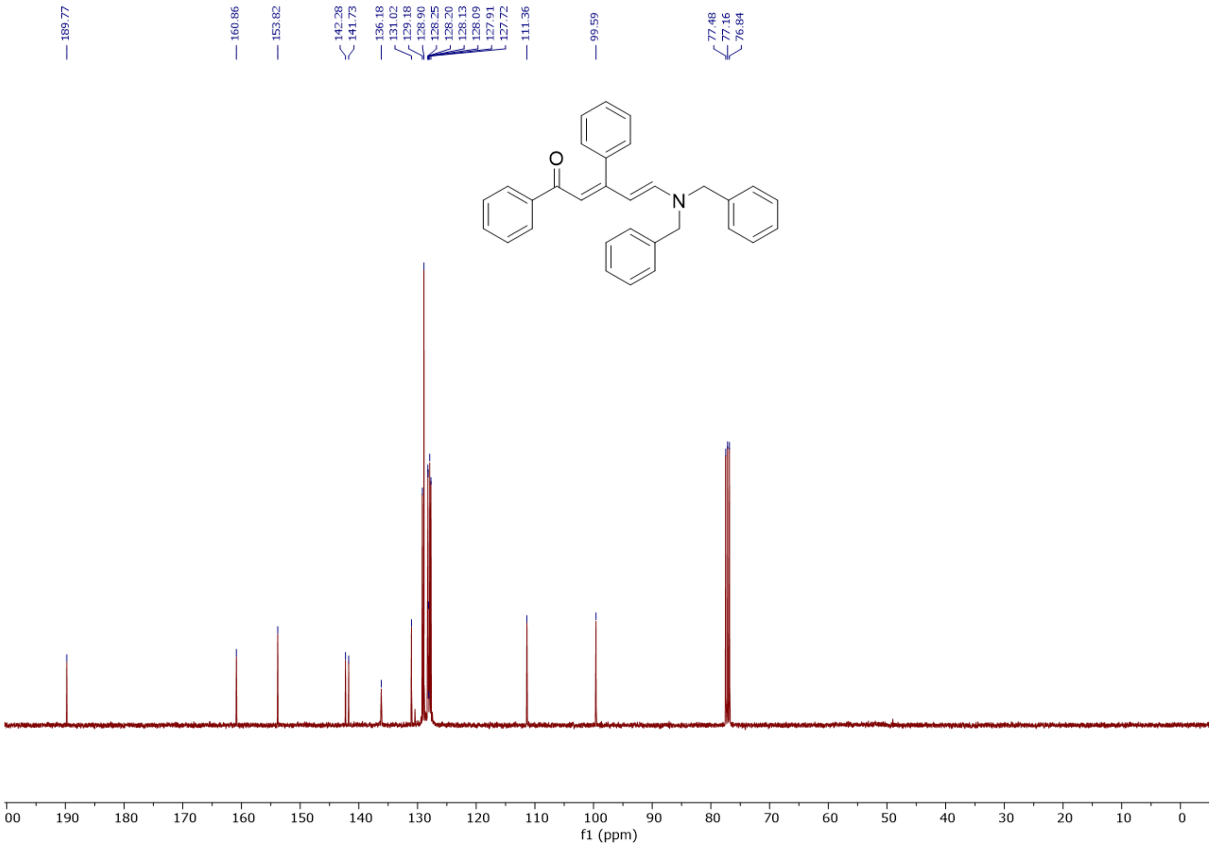
189.77
160.86
153.82
142.28
141.73
136.18
131.02
129.18
128.90
128.25
128.20
128.13
128.09
127.91
127.72
111.36
99.59
77.48
77.16
76.84
O
N
f1 (ppm)

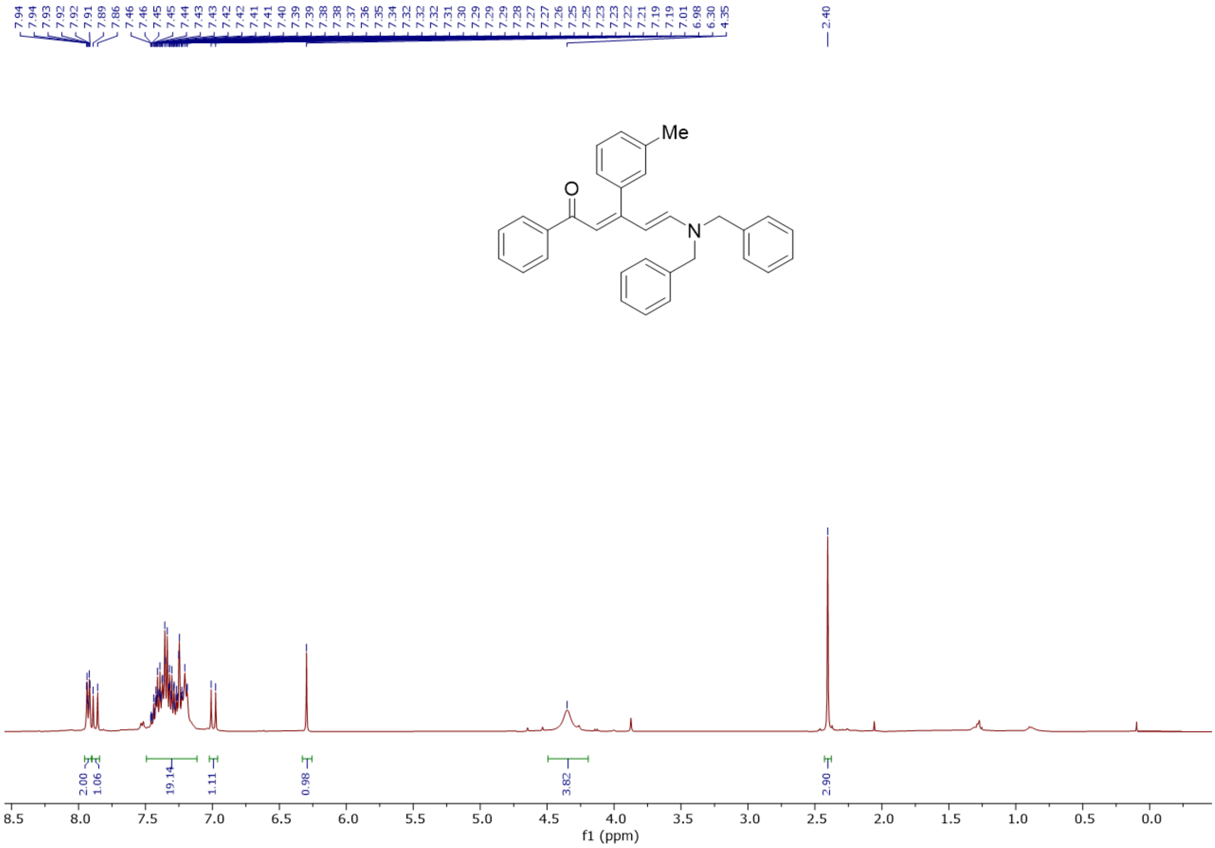
7.94
7.94
7.93
7.92
7.92
7.91
7.89
7.86
7.46
7.46
7.45
7.45
7.44
7.43
7.43
7.42
7.42
7.41
7.41
7.40
7.39
7.39
7.38
7.38
7.37
7.36
7.35
7.34
7.32
7.32
7.32
7.31
7.30
7.29
7.29
7.29
7.28
7.27
7.27
7.26
7.25
7.25
7.23
7.23
7.22
7.21
7.19
7.01
6.98
6.30
4.35
2.40
Me
O
N
2.00
1.06
19.14
1.11
0.98
3.82
2.90
f1 (ppm)

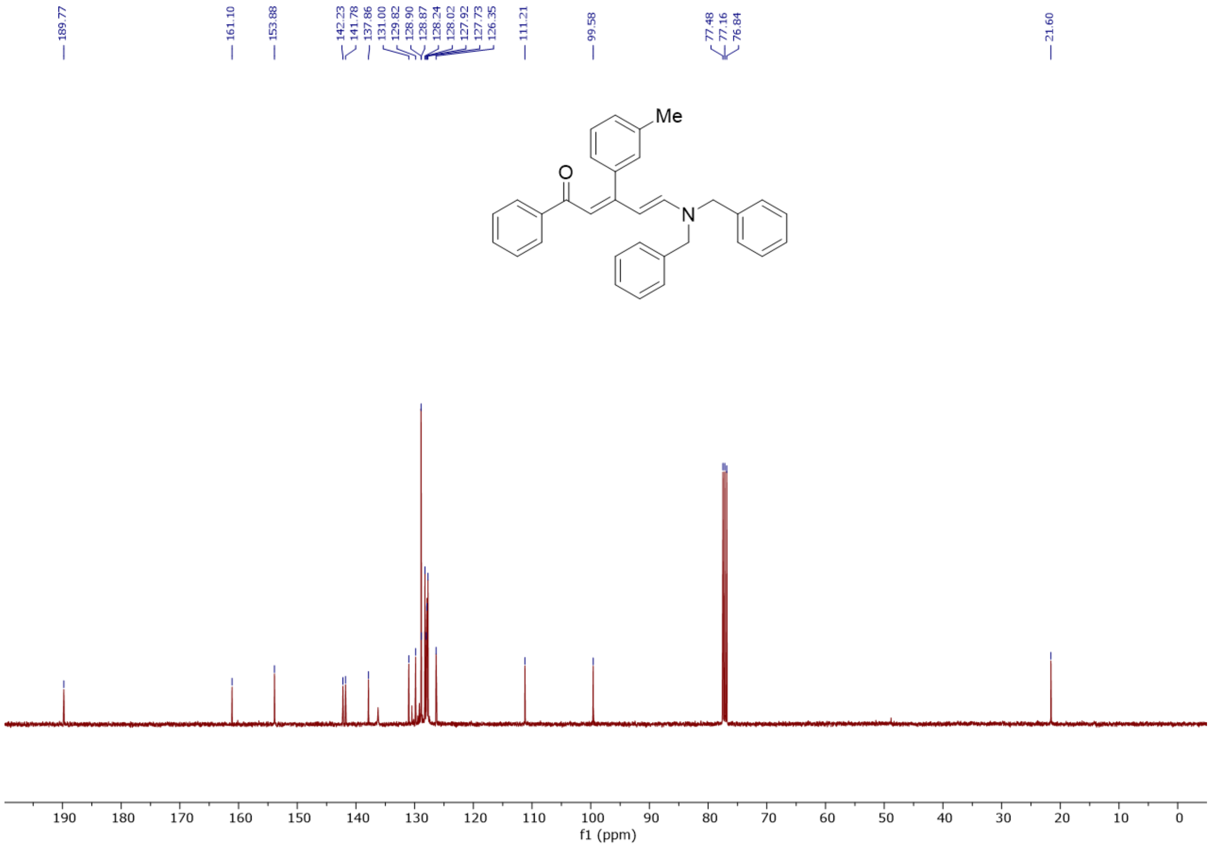
189.77
161.10
153.88
142.23
141.78
137.86
131.00
129.82
128.90
128.87
128.24
128.02
127.92
127.73
126.35
111.21
99.58
77.48
77.16
76.84
21.60
Me
O
N
f1 (ppm)

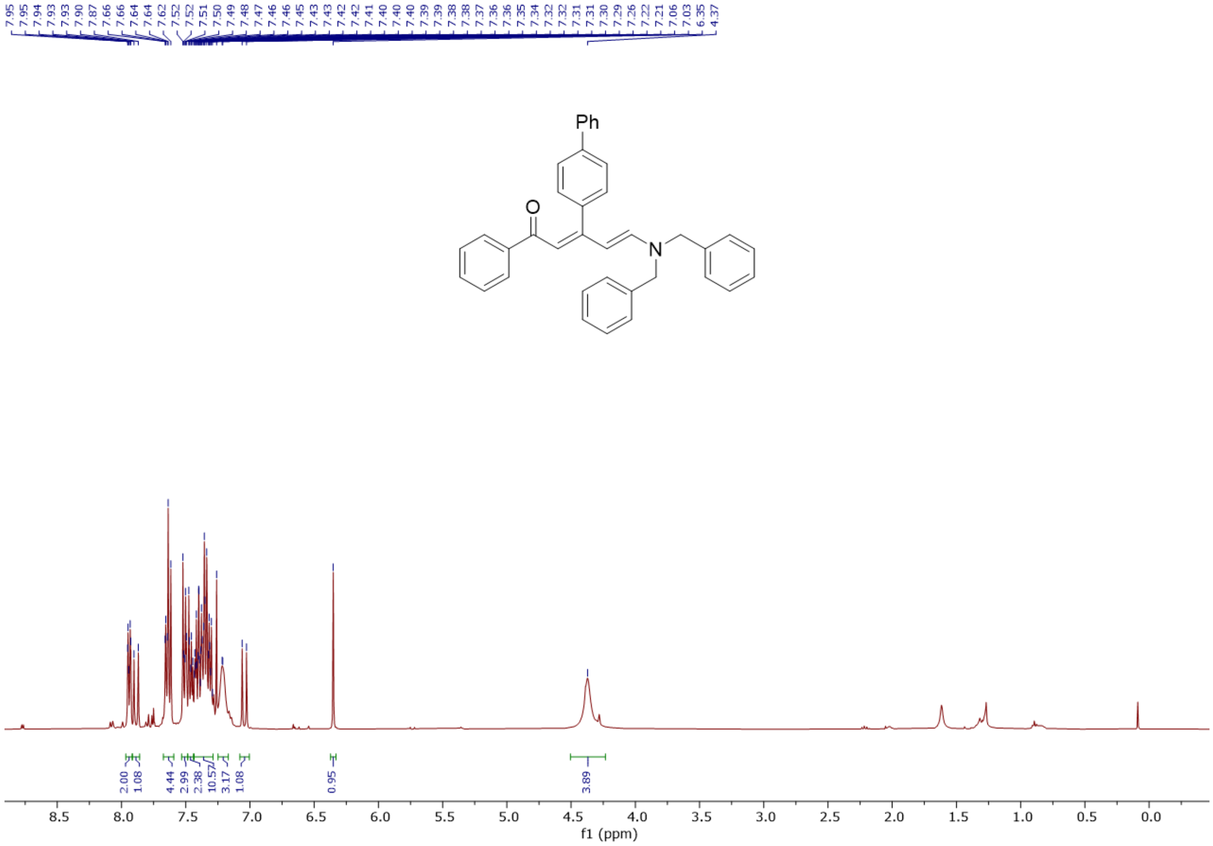
Ph
O
N
2.00
1.08
4.44
2.99
2.38
10.57
3.17
1.08
0.95
3.89
f1 (ppm)

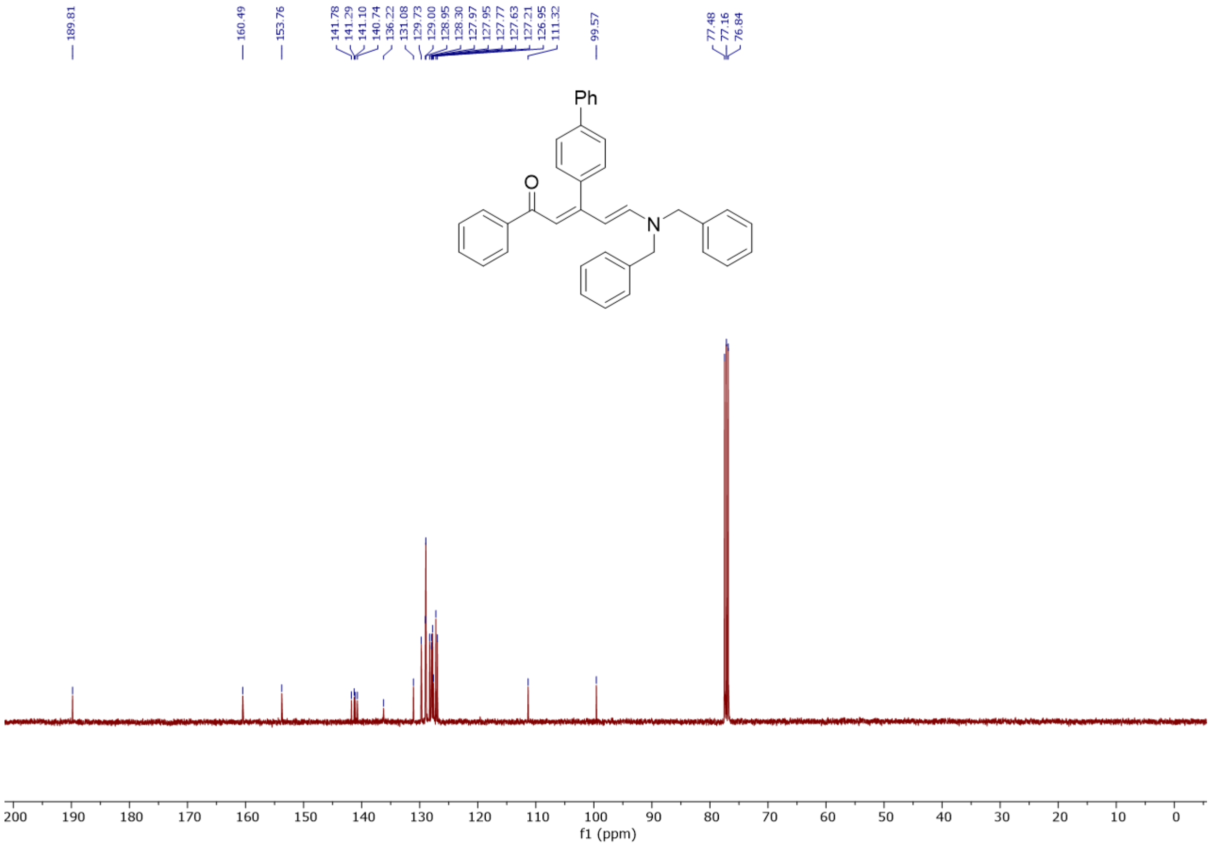
189.81
160.49
153.76
141.78
141.29
141.10
140.74
136.22
131.08
129.73
129.00
128.95
128.30
127.97
127.95
127.77
127.63
127.21
126.95
111.32
99.57
77.48
77.16
76.84
Ph
O
N
200 190 180 170 160 150 140 130 120 110 100 90 80 70 60 50 40 30 20 10 0
f1 (ppm)

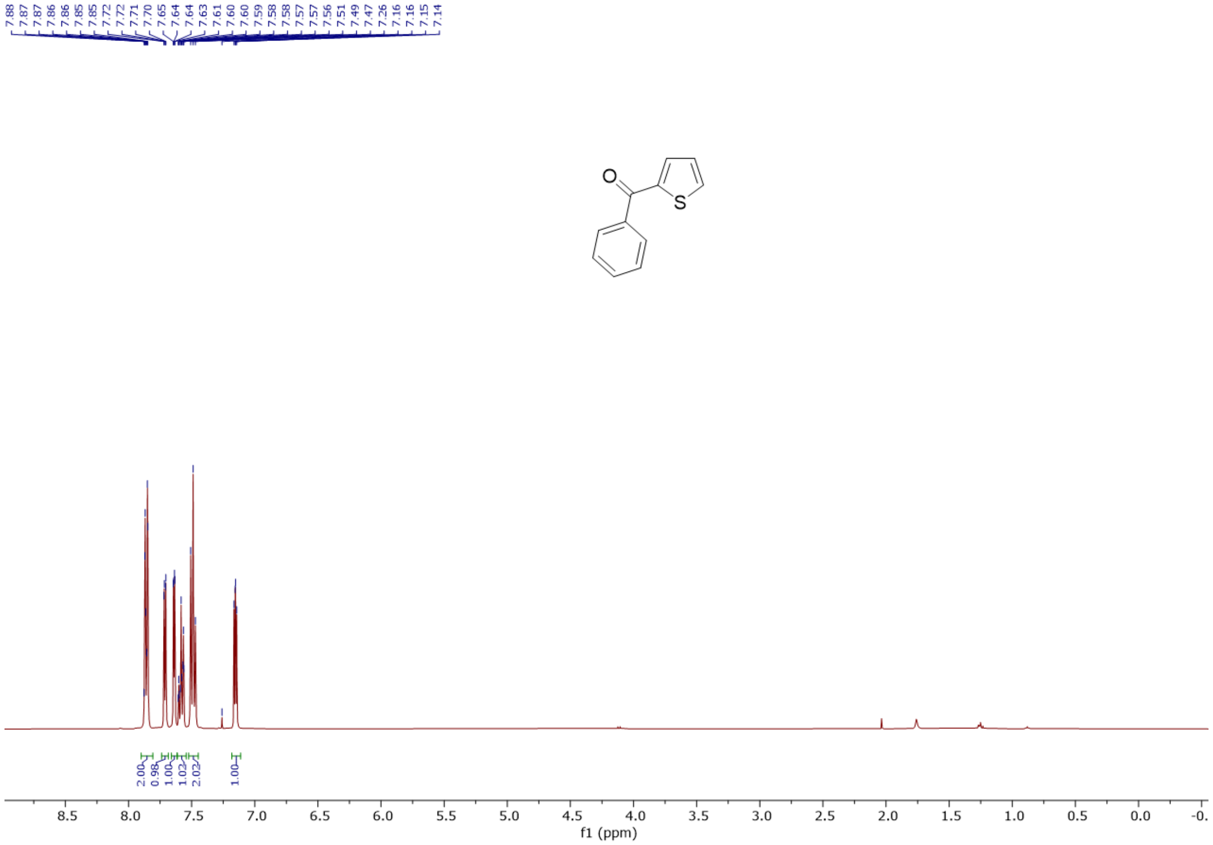
7.88
7.87
7.87
7.86
7.86
7.85
7.85
7.72
7.72
7.71
7.70
7.65
7.64
7.64
7.63
7.61
7.60
7.59
7.58
7.58
7.57
7.57
7.56
7.51
7.49
7.47
7.26
7.16
7.16
7.15
7.14
O
S
2.00
0.98
1.00
1.02
2.02
1.00
8.5 8.0 7.5 7.0 6.5 6.0 5.5 5.0 4.5 4.0 3.5 3.0 2.5 2.0 1.5 1.0 0.5 0.0 -0.
f1 (ppm)

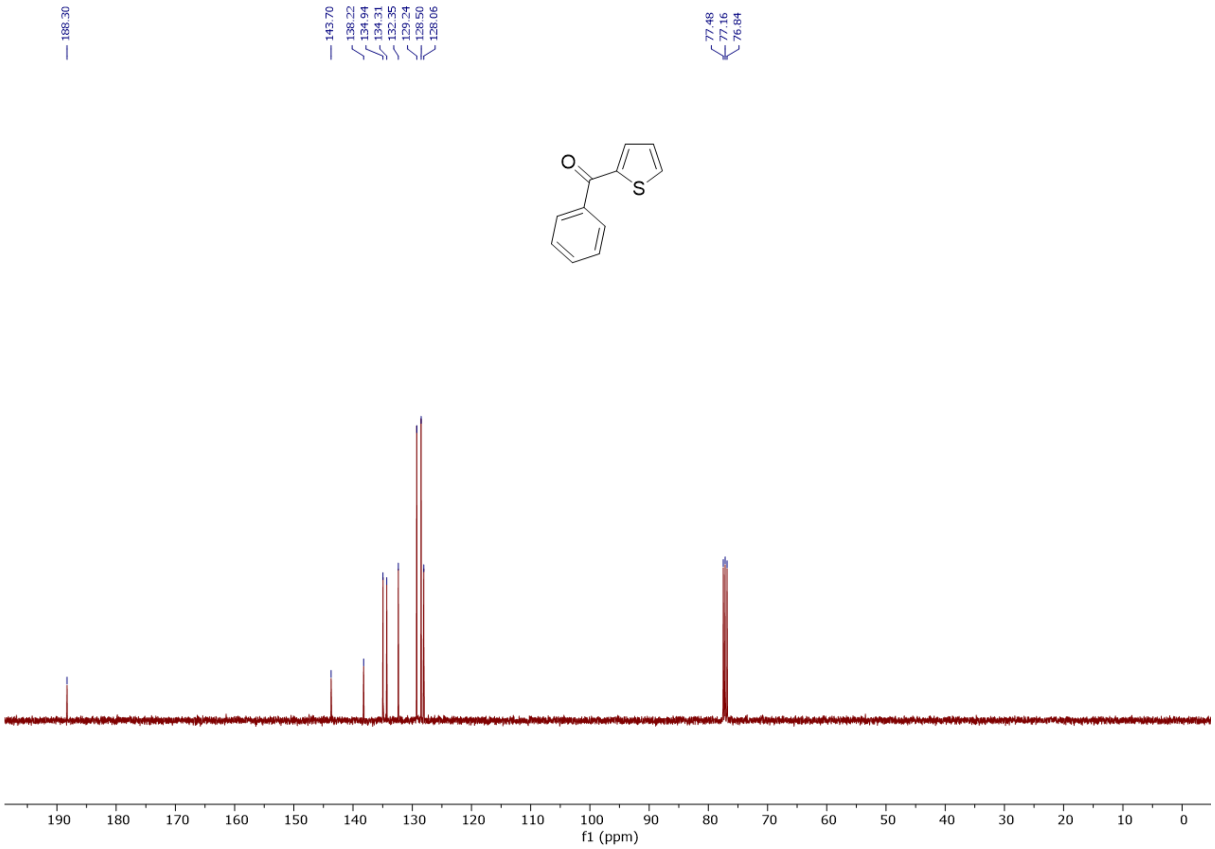

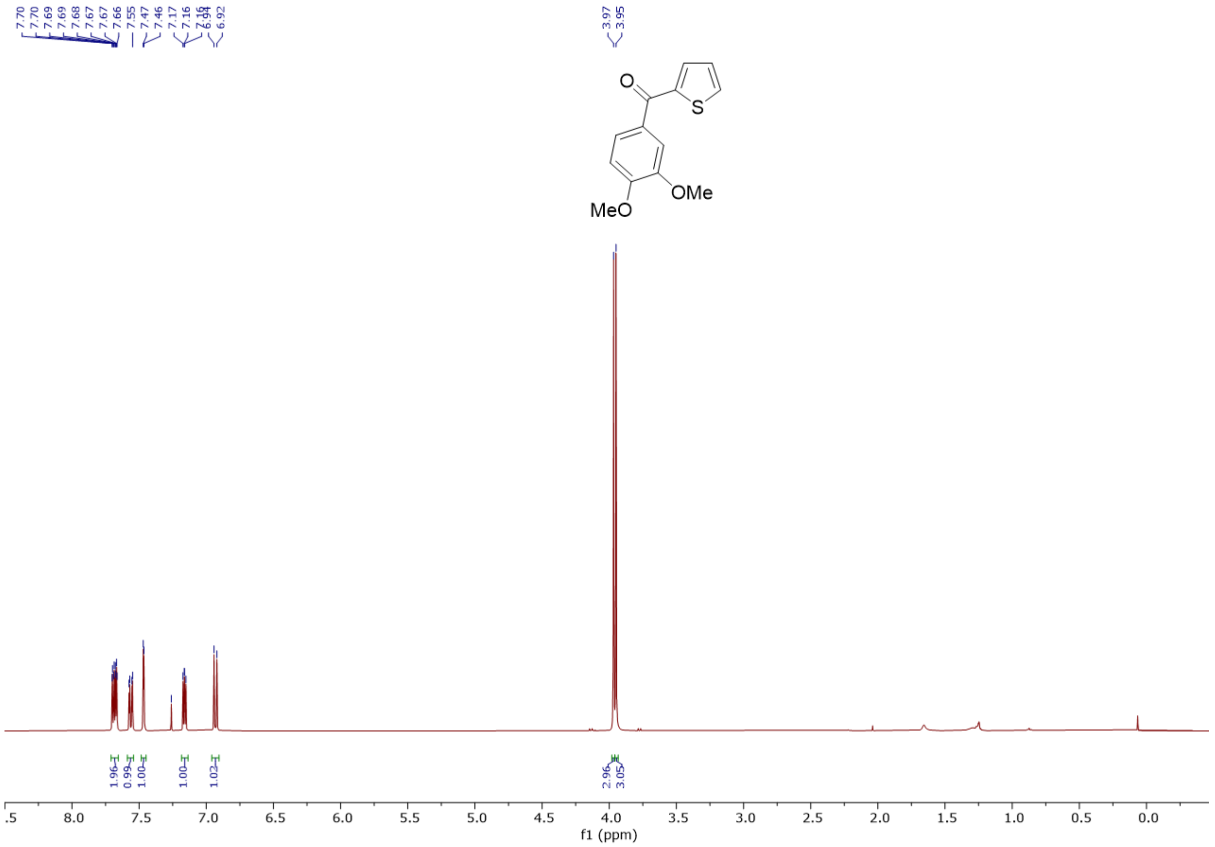

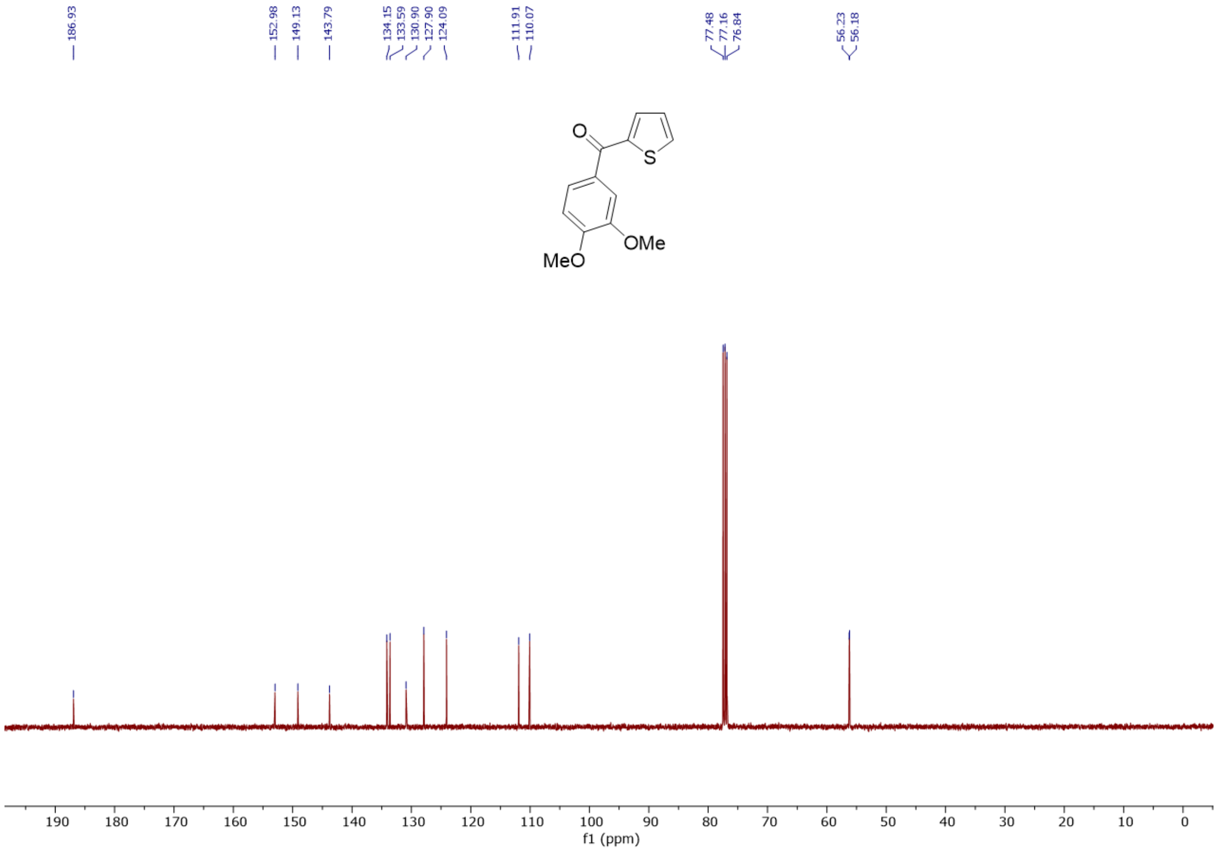
186.93
152.98
149.13
143.79
134.15
133.59
130.90
127.90
124.09
111.91
110.07
77.48
77.16
76.84
56.23
56.18
O
S
OMe
MeO
190 180 170 160 150 140 130 120 110 100 90 80 70 60 50 40 30 20 10 0
f1 (ppm)

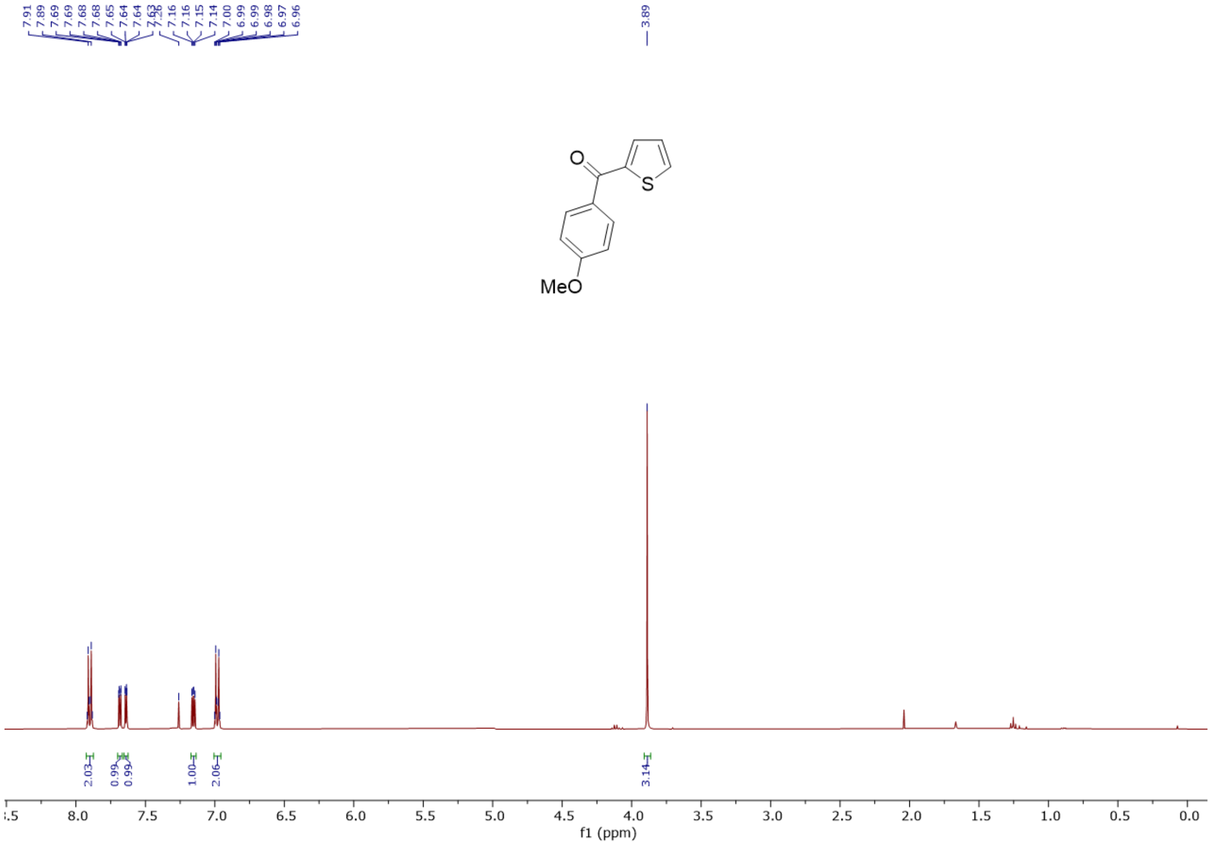
7.91
7.89
7.69
7.69
7.68
7.68
7.65
7.64
7.64
7.63
7.26
7.16
7.16
7.15
7.14
7.00
6.99
6.99
6.98
6.97
6.96
3.89
O
S
MeO
2.03
0.99
0.99
1.00
2.06
3.14
8.0 7.5 7.0 6.5 6.0 5.5 5.0 4.5 4.0 3.5 3.0 2.5 2.0 1.5 1.0 0.5 0.0
f1 (ppm)

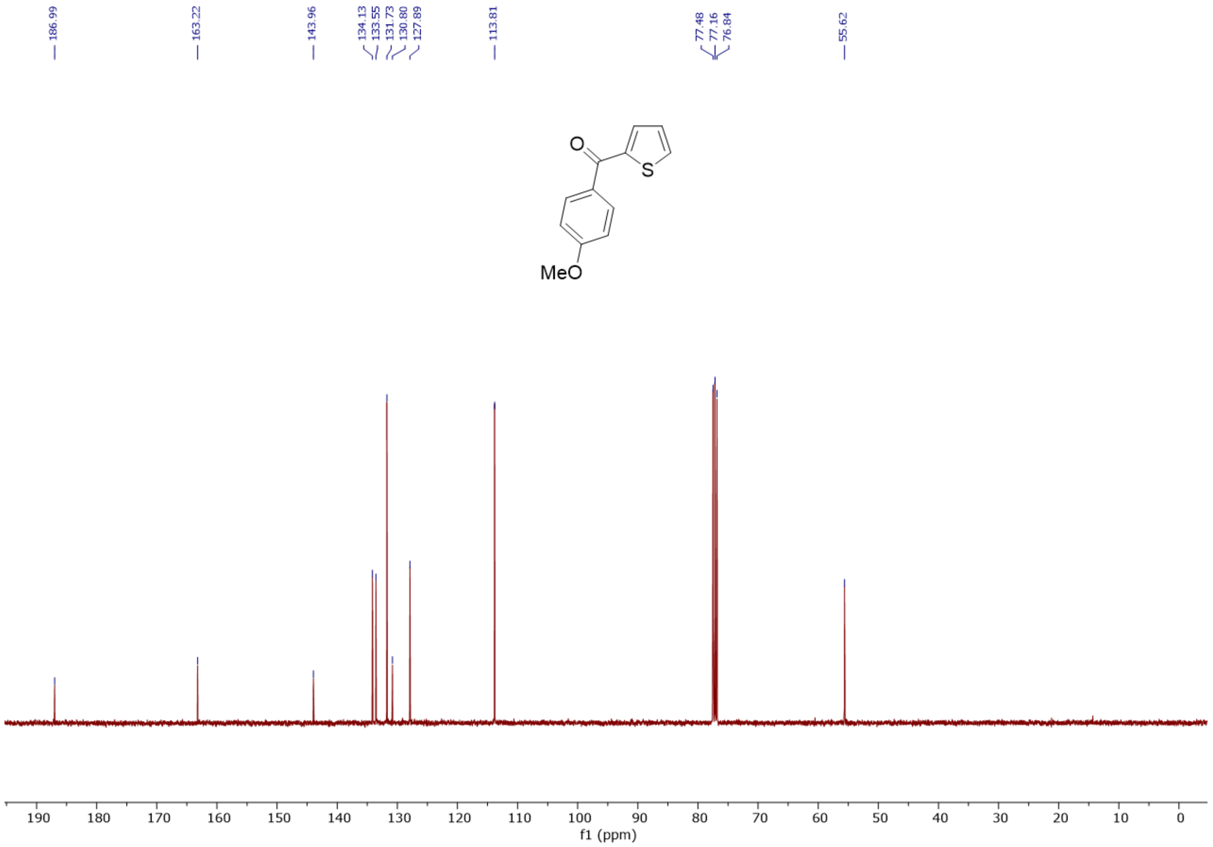
186.99
163.22
143.96
134.13
133.55
131.73
130.80
127.89
113.81
77.48
77.16
76.84
55.62
O
S
MeO
f1 (ppm)

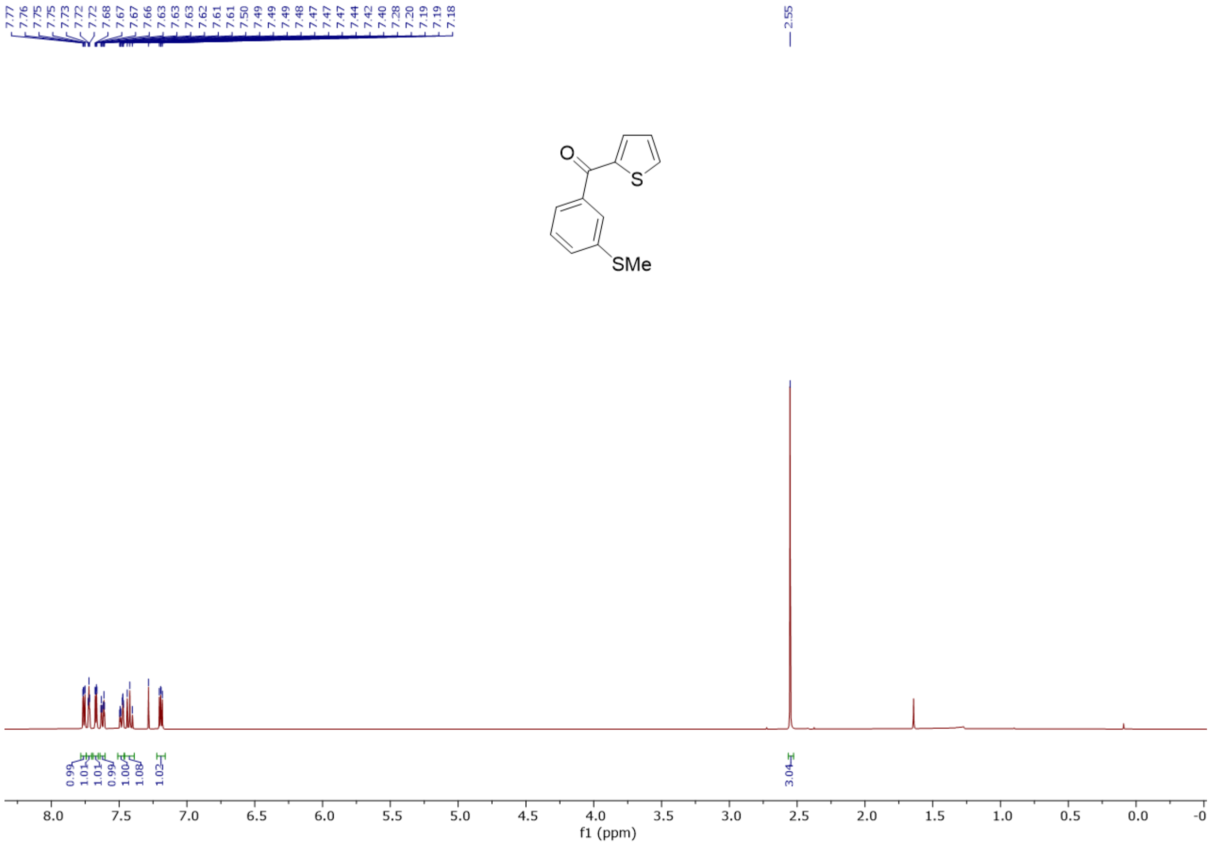
7.77
7.76
7.75
7.75
7.73
7.72
7.72
7.68
7.67
7.67
7.66
7.63
7.63
7.63
7.62
7.61
7.61
7.50
7.49
7.49
7.49
7.48
7.47
7.47
7.47
7.44
7.42
7.40
7.28
7.20
7.19
7.19
7.18
2.55
O
S
SMe
0.99
1.01
1.01
0.99
1.00
1.08
1.02
3.04
f1 (ppm)

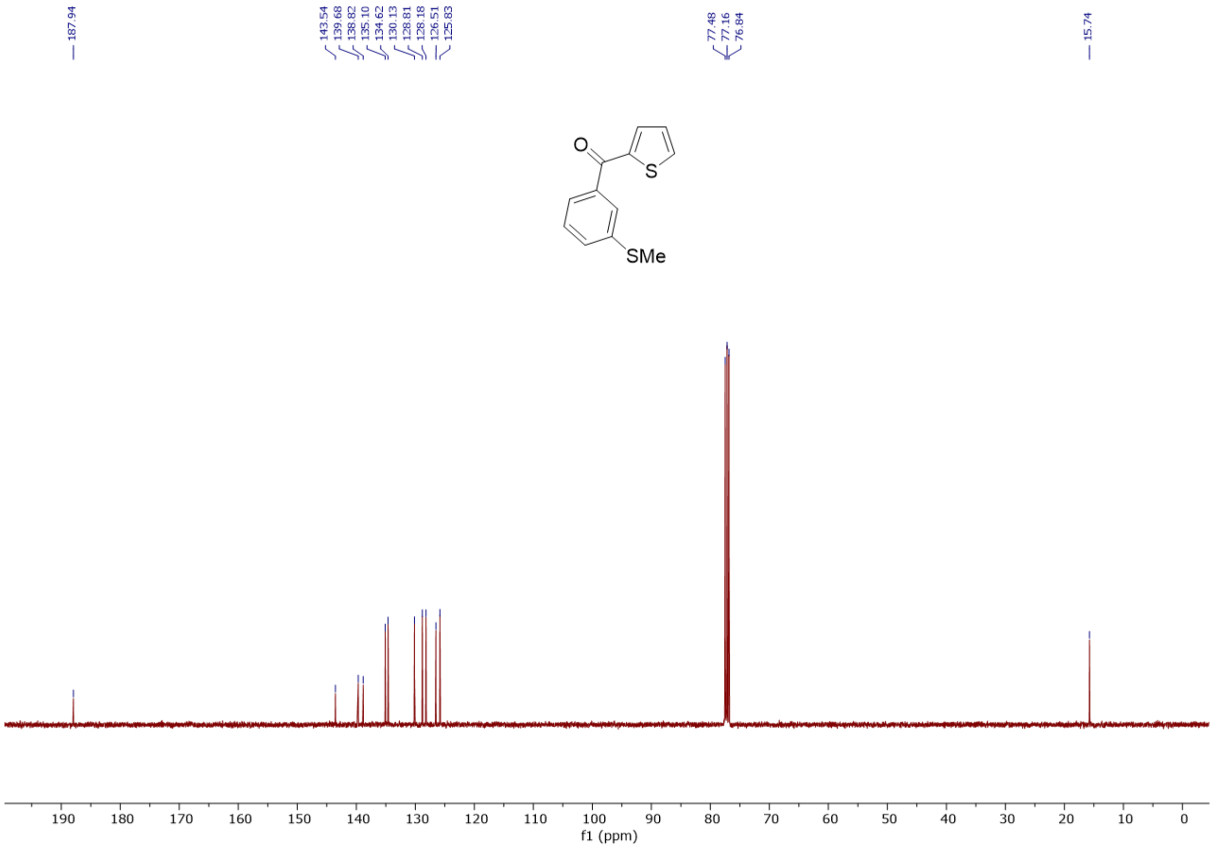

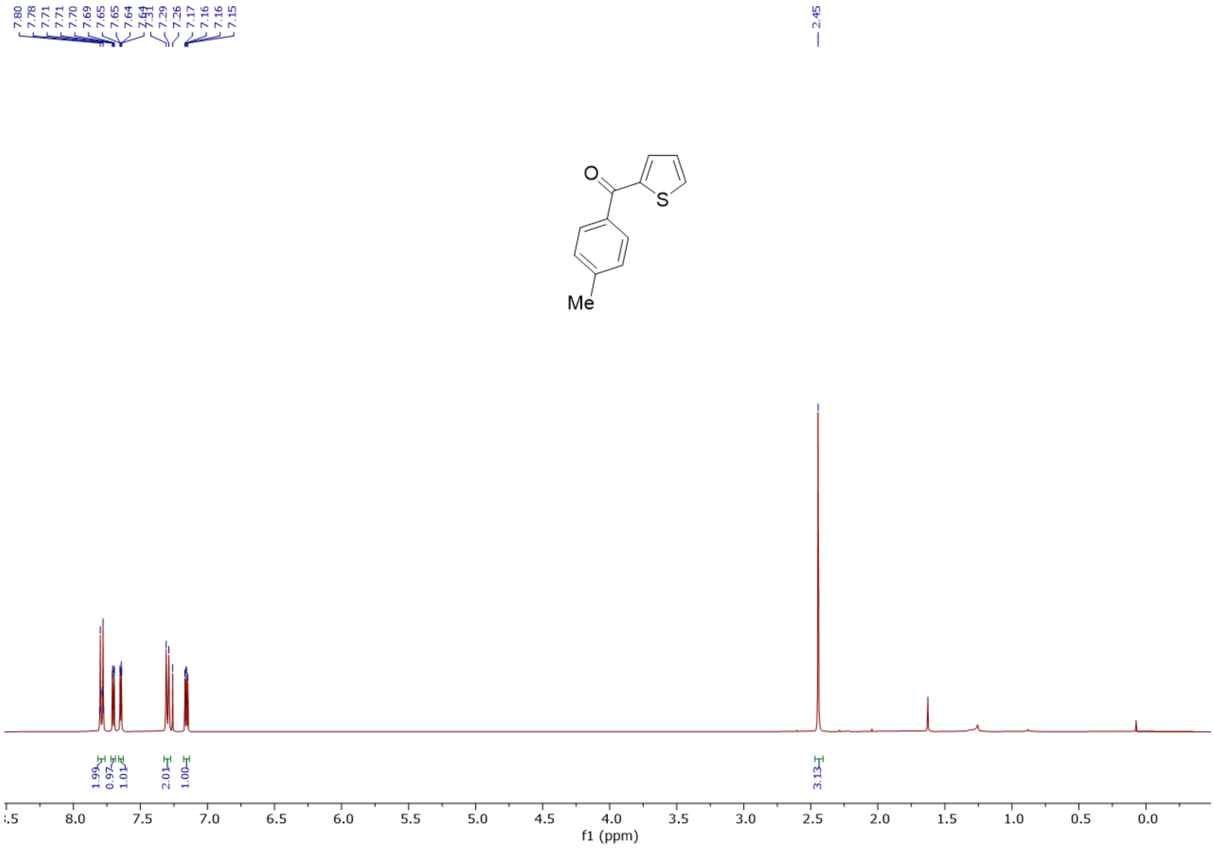

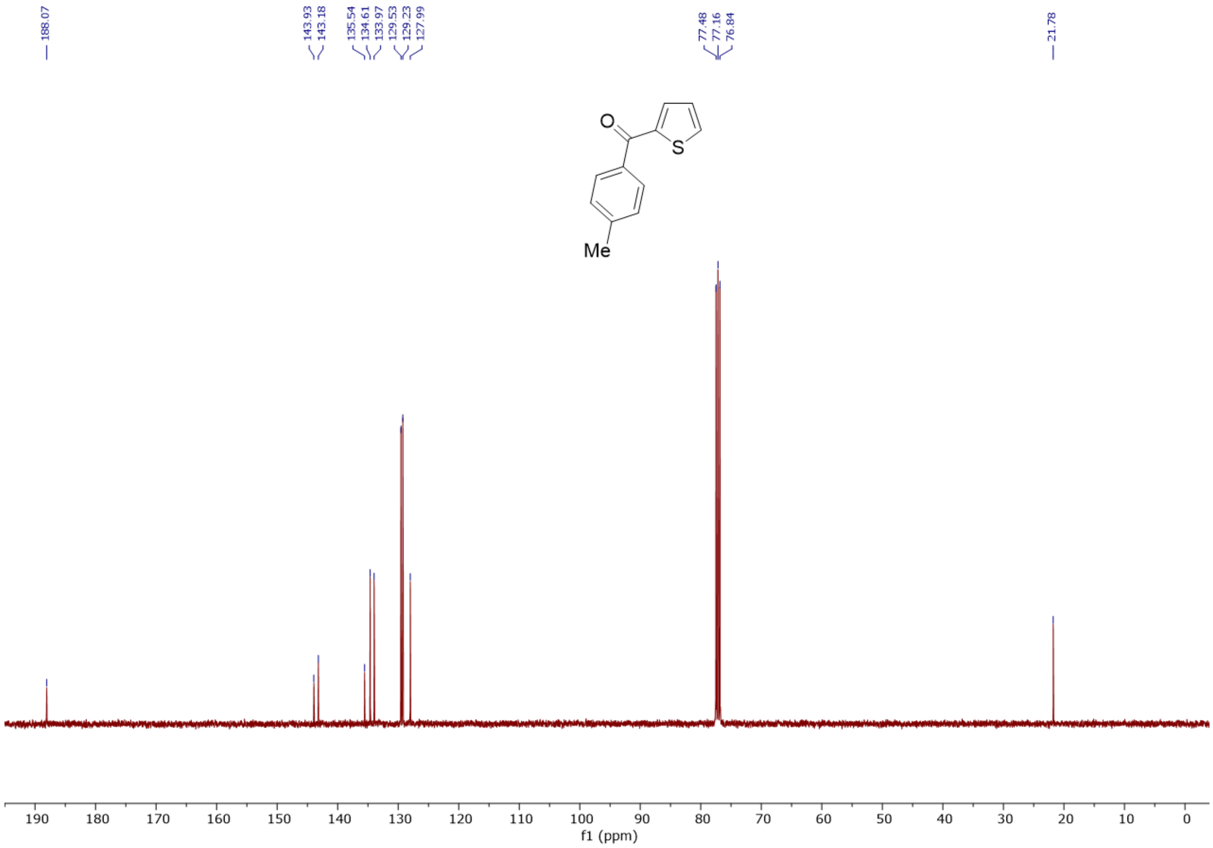
188.07
143.93
143.18
135.54
134.61
133.97
129.53
129.23
127.99
77.48
77.16
76.84
21.78
O
S
Me
f1 (ppm)

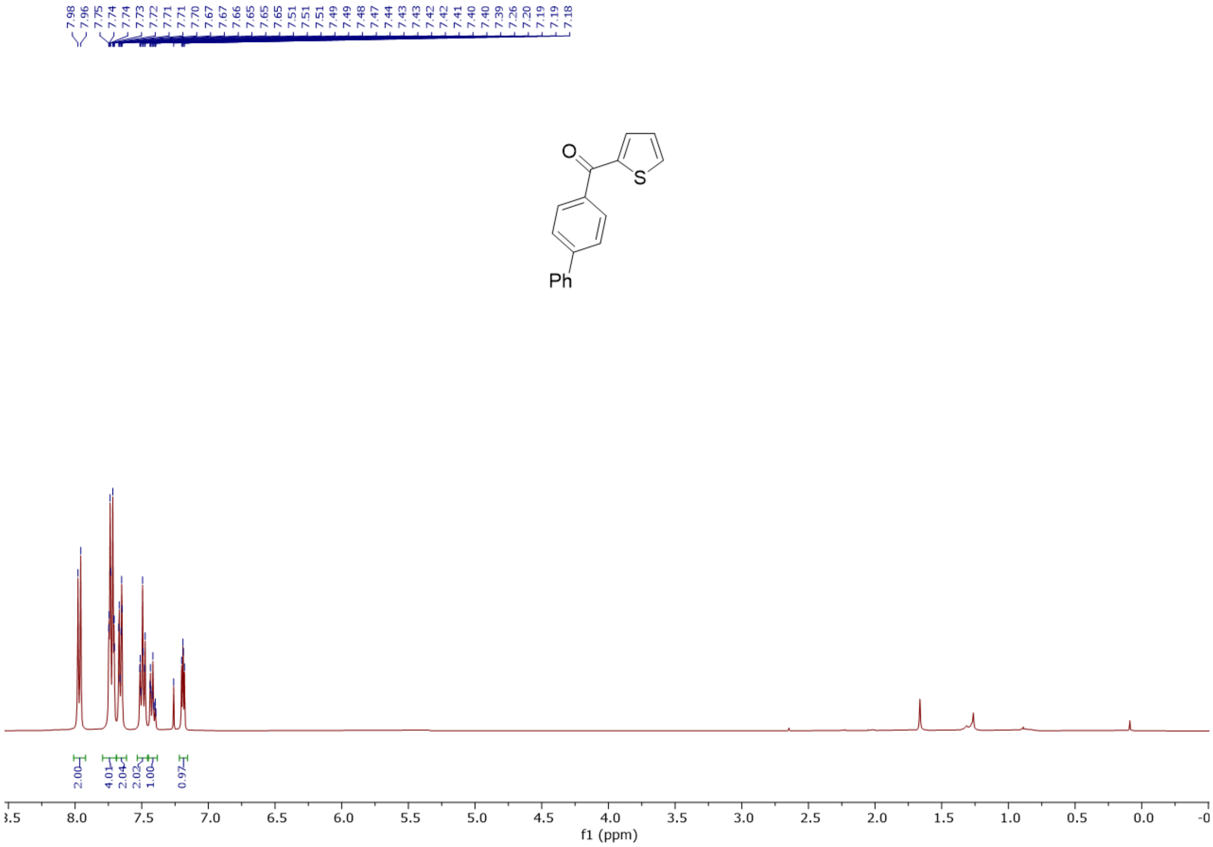
O
S
Ph
2.00
4.01
2.04
2.02
1.00
0.97
f1 (ppm)

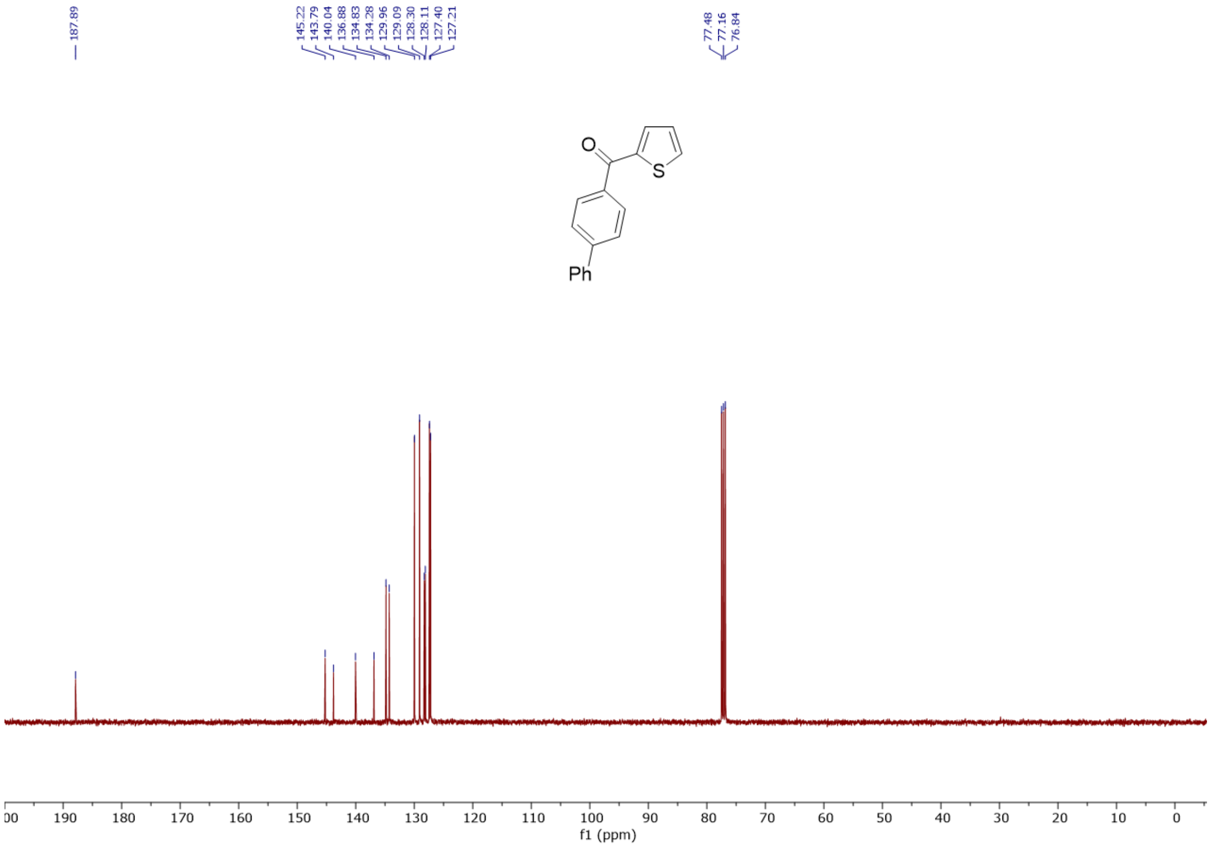

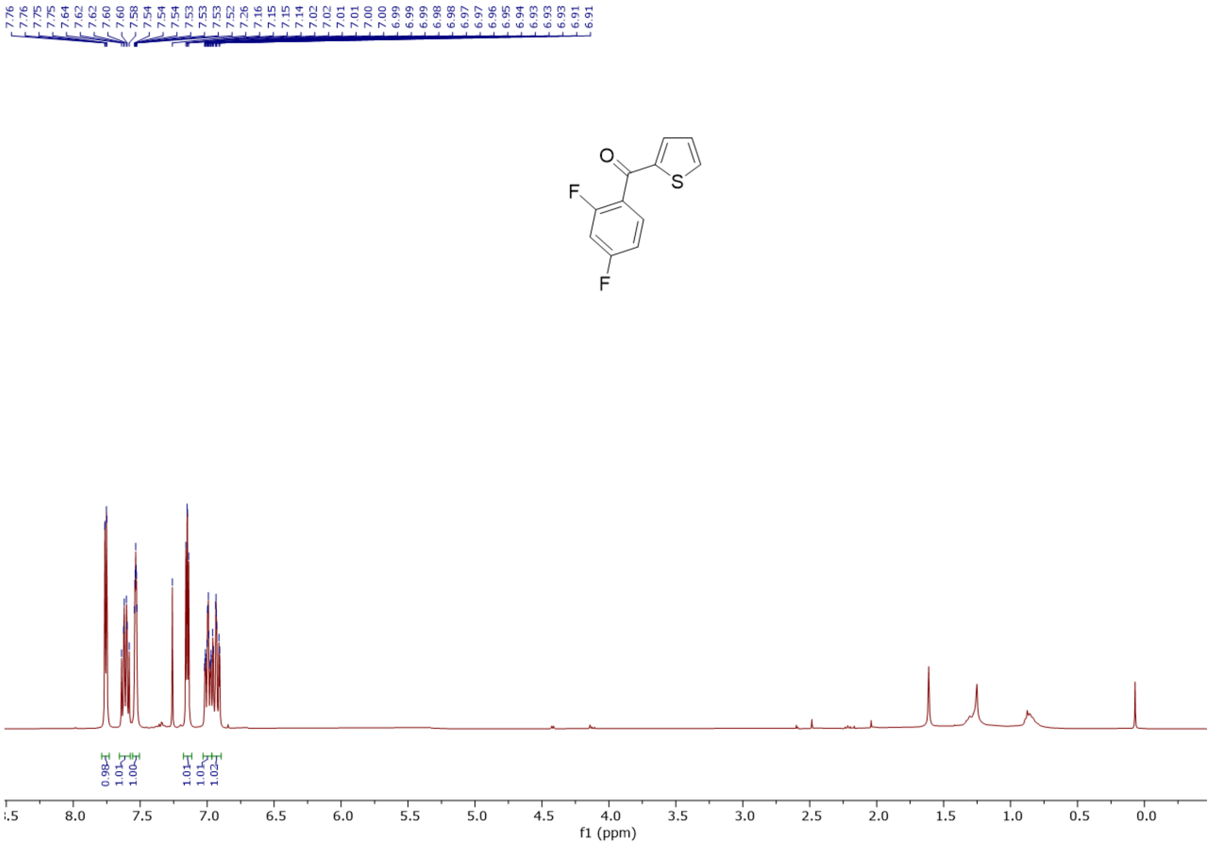

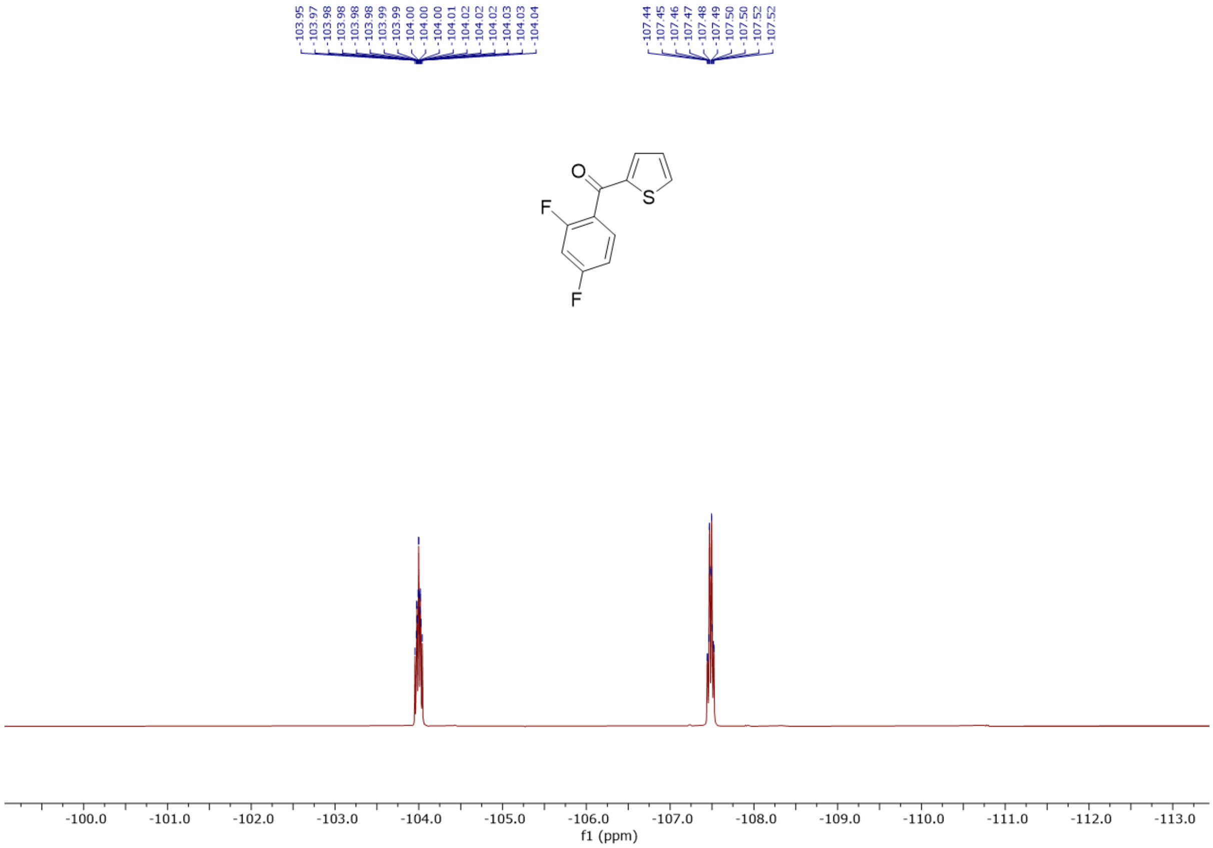

-103.95
-103.97
-103.98
-103.98
-103.98
-103.98
-103.99
-103.99
-104.00
-104.00
-104.00
-104.01
-104.02
-104.02
-104.02
-104.03
-104.03
-104.04
-107.44
-107.45
-107.46
-107.47
-107.48
-107.49
-107.50
-107.50
-107.52
-107.52
O
S
F
F
-100.0
-101.0
-102.0
-103.0
-104.0
-105.0
-106.0
-107.0
-108.0
-109.0
-110.0
-111.0
-112.0
-113.0
f1 (ppm)


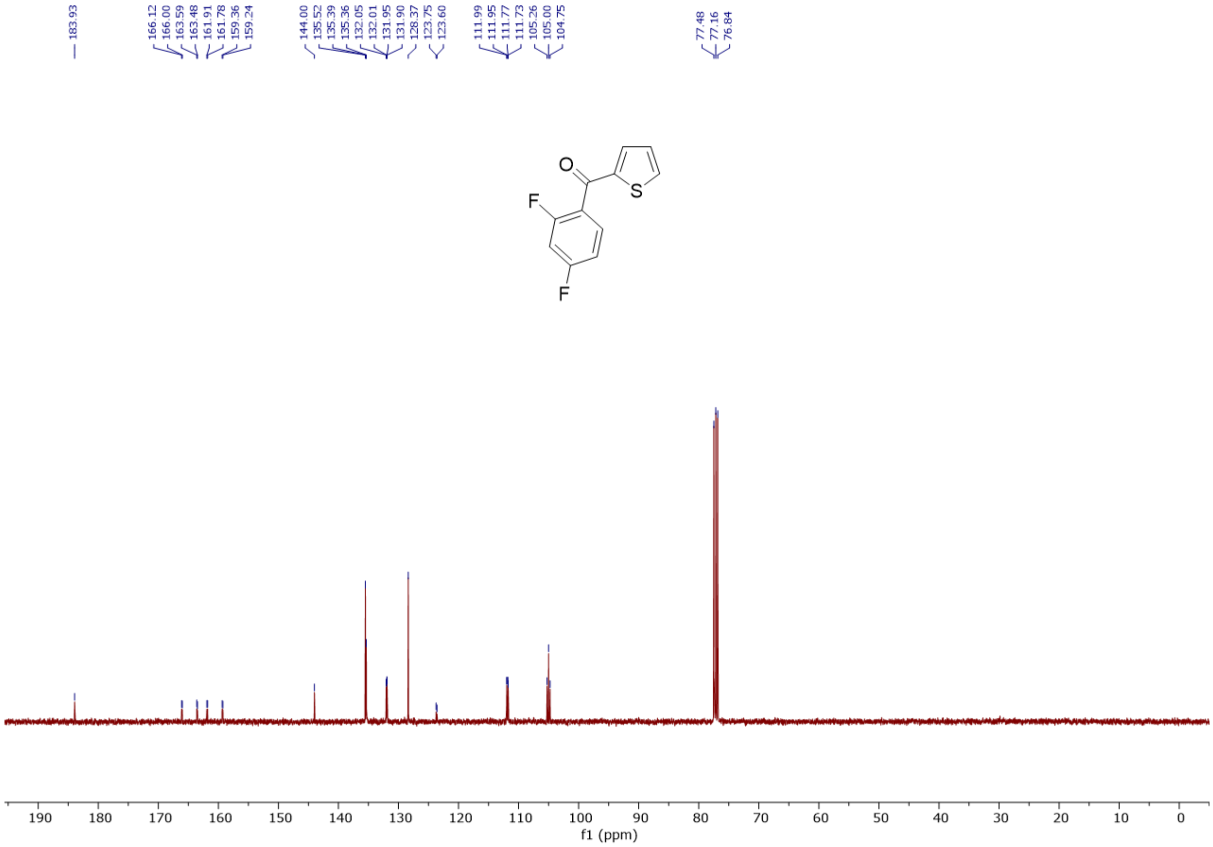

183.93
166.12
166.00
163.59
163.48
161.91
161.78
159.36
159.24
144.00
135.52
135.39
135.36
132.05
132.01
131.95
131.90
128.37
123.75
123.60
111.99
111.95
111.77
111.73
105.26
105.00
104.75
77.48
77.16
76.84
O
S
F
F
190
180
170
160
150
140
130
120
110
100
90
80
70
60
50
40
30
20
10
0
f1 (ppm)

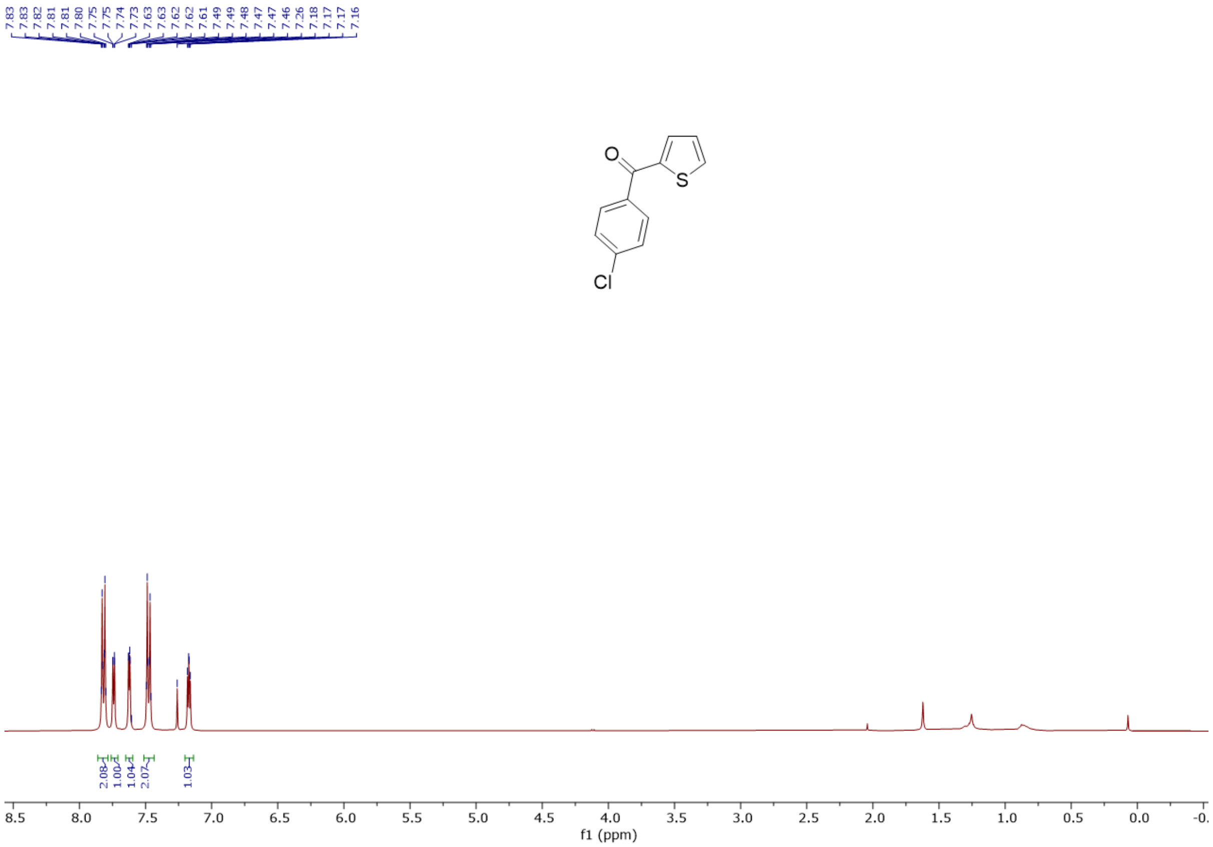
7.83
7.83
7.82
7.81
7.81
7.80
7.75
7.75
7.74
7.73
7.63
7.63
7.62
7.62
7.61
7.49
7.49
7.48
7.47
7.47
7.46
7.26
7.18
7.17
7.17
7.16
O
S
Cl
2.08
1.00
1.04
2.07
1.03
f1 (ppm)

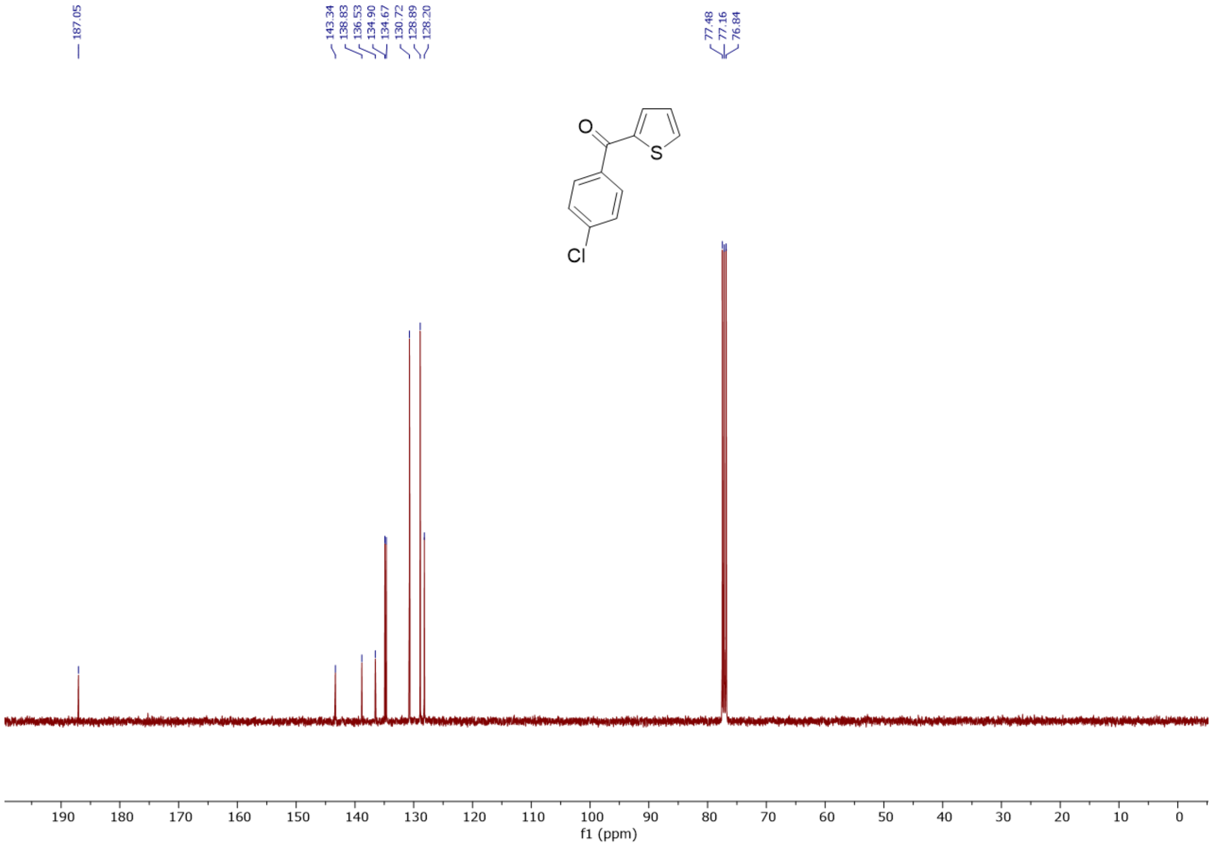
187.05
143.34
138.83
136.53
134.90
134.67
130.72
128.89
128.20
77.48
77.16
76.84
O
S
Cl
f1 (ppm)

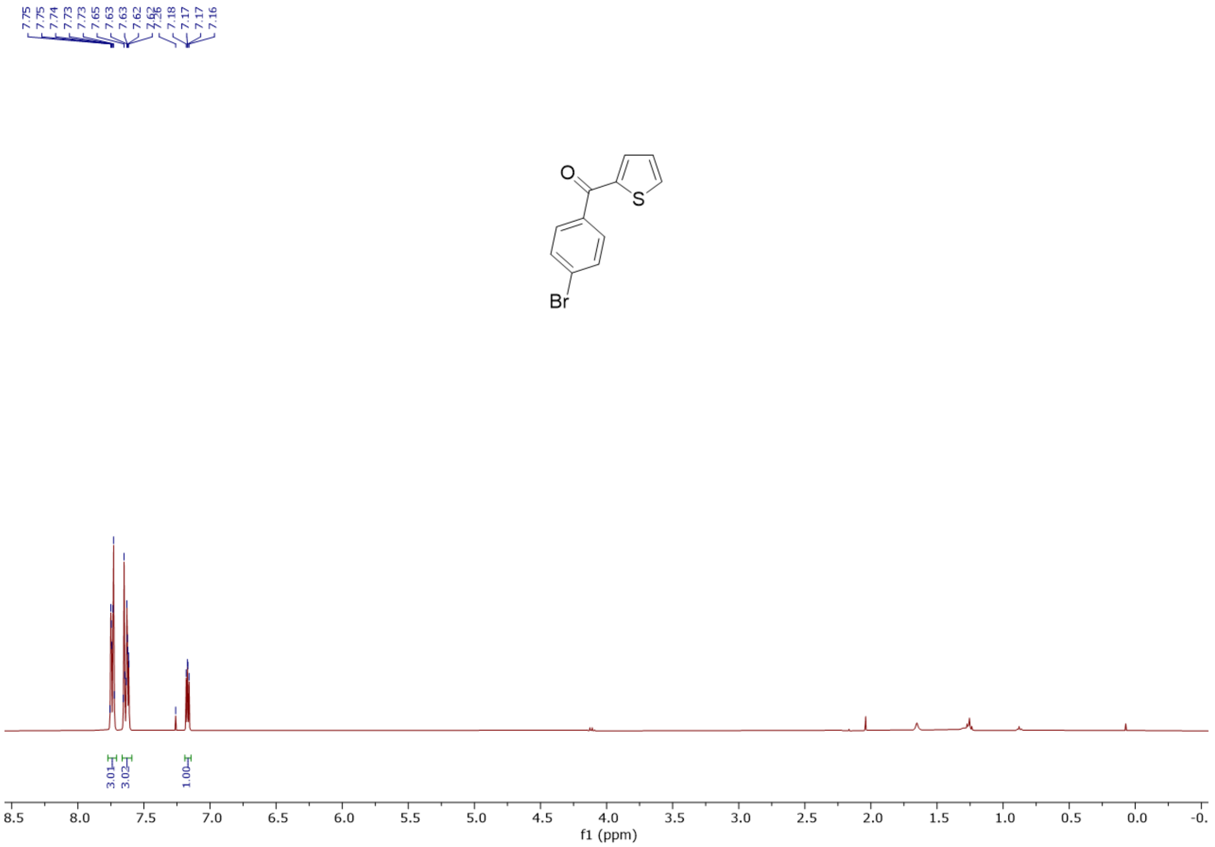
7.75
7.75
7.74
7.73
7.73
7.65
7.63
7.63
7.62
7.62
7.26
7.18
7.17
7.17
7.16
O
S
Br
3.01
3.02
1.00
f1 (ppm)

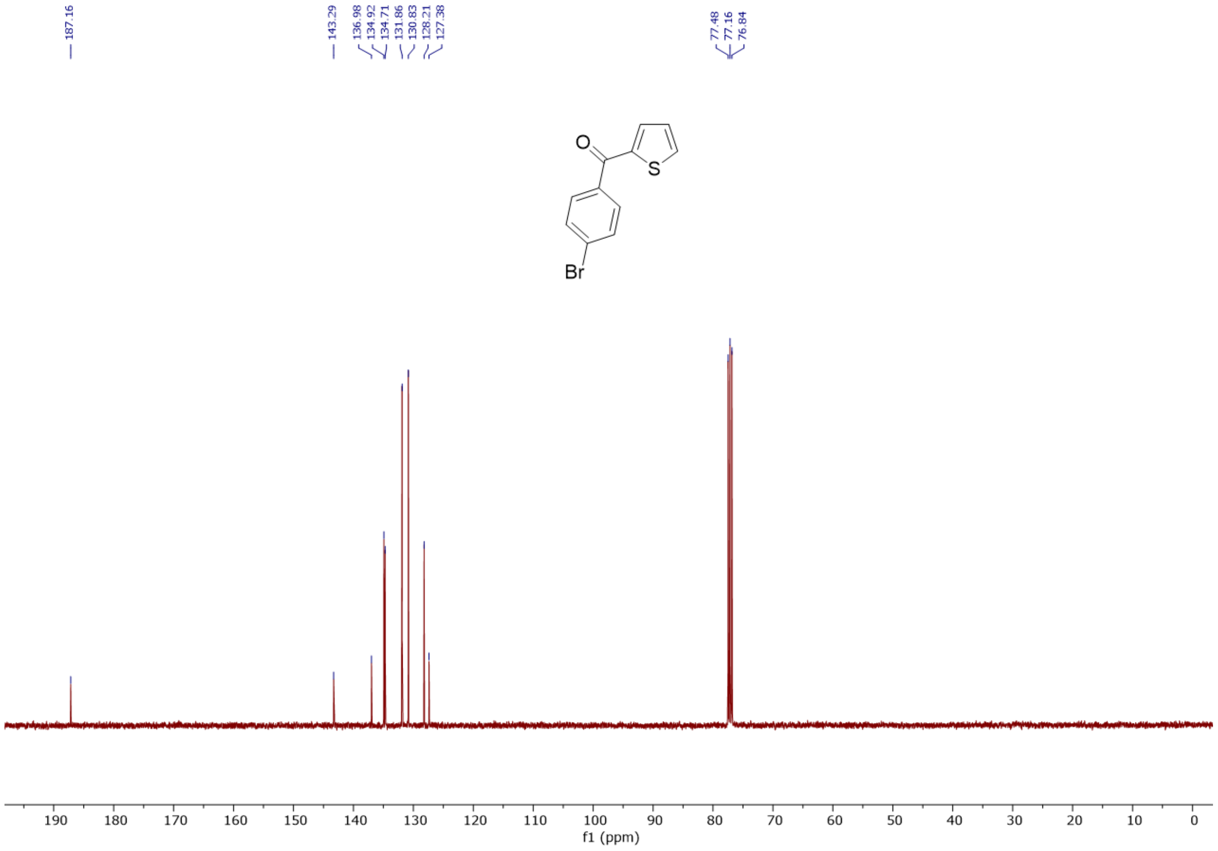
187.16
143.29
136.98
134.92
134.71
131.86
130.83
128.21
127.38
77.48
77.16
76.84
O
S
Br
f1 (ppm)

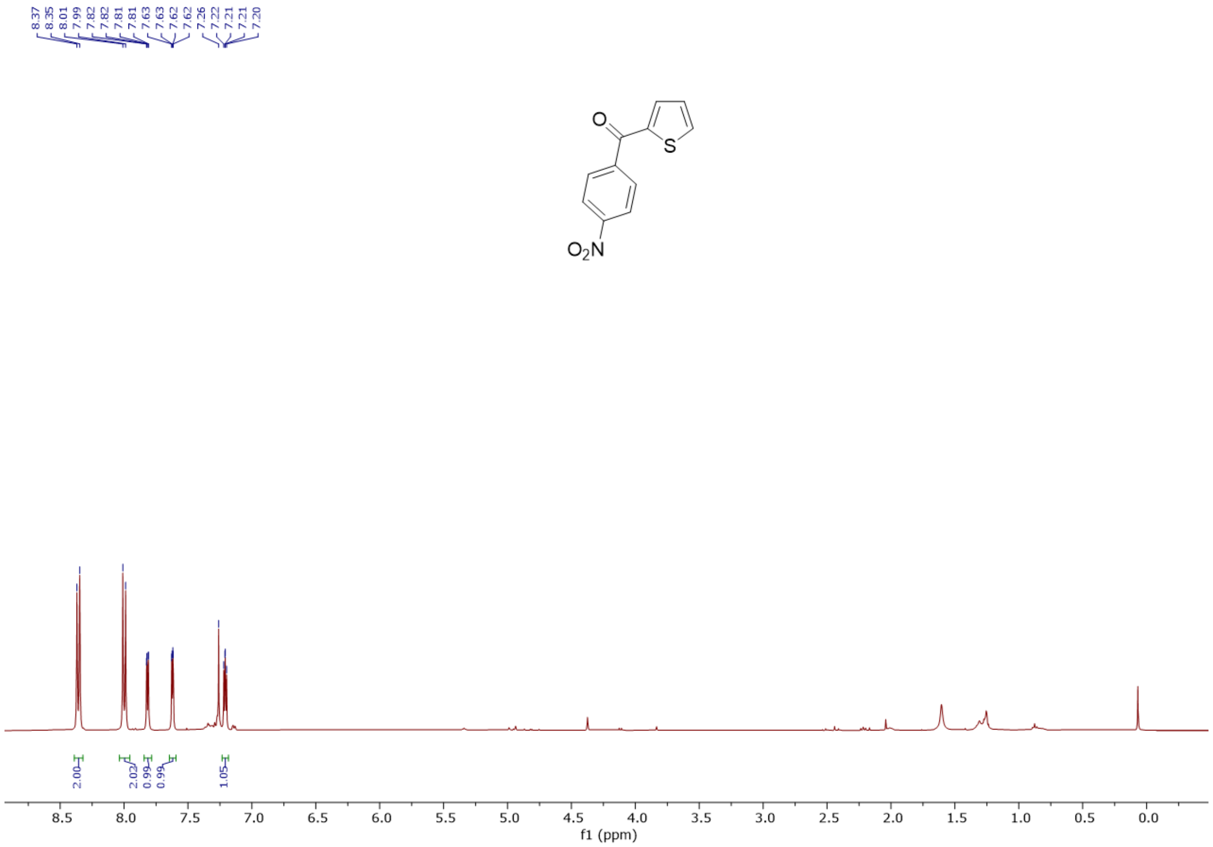
8.37
8.35
8.01
7.99
7.82
7.82
7.81
7.81
7.63
7.63
7.62
7.62
7.26
7.22
7.21
7.21
7.20
O
S
O2N
2.00
2.02
0.99
0.99
1.05
8.5 8.0 7.5 7.0 6.5 6.0 5.5 5.0 4.5 4.0 3.5 3.0 2.5 2.0 1.5 1.0 0.5 0.0
f1 (ppm)

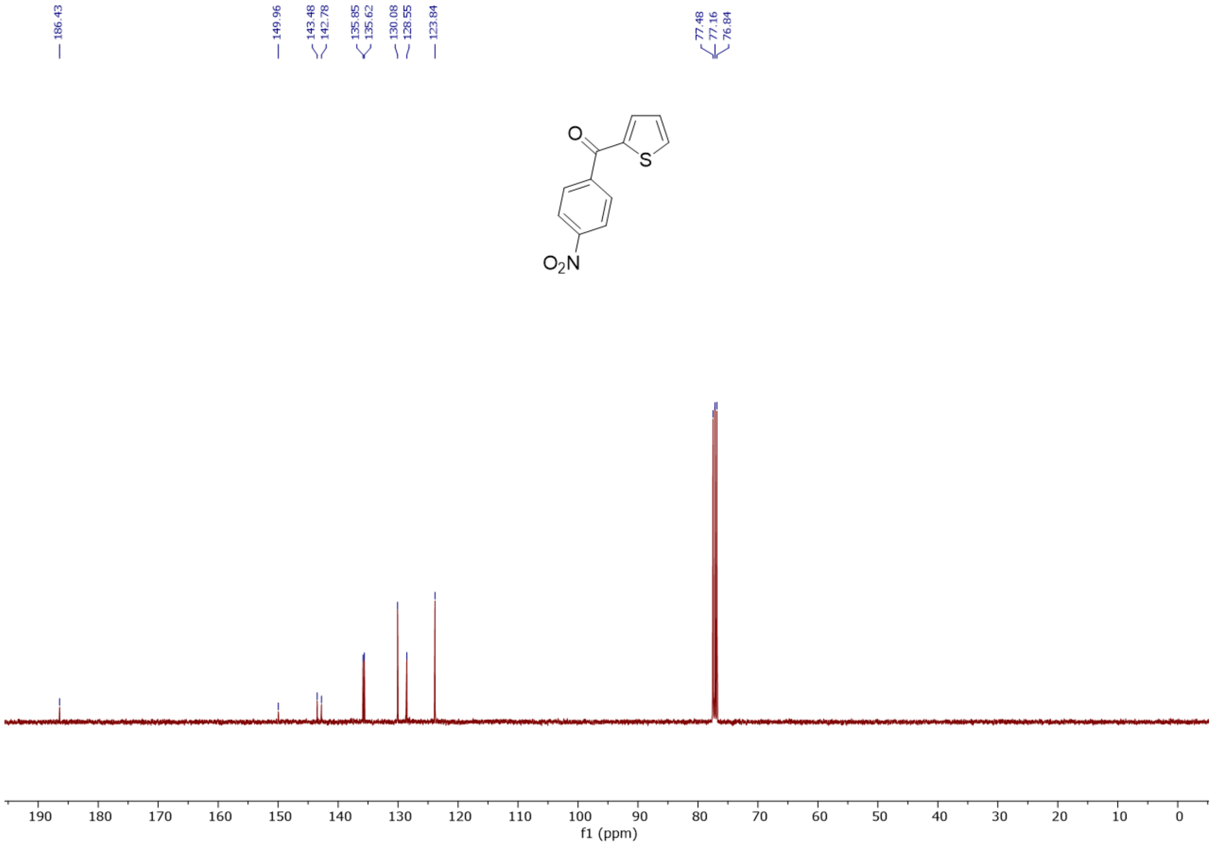
186.43
149.96
143.48
142.78
135.85
135.62
130.08
128.55
123.84
77.48
77.16
76.84
O
S
O2N
190 180 170 160 150 140 130 120 110 100 90 80 70 60 50 40 30 20 10 0
f1 (ppm)

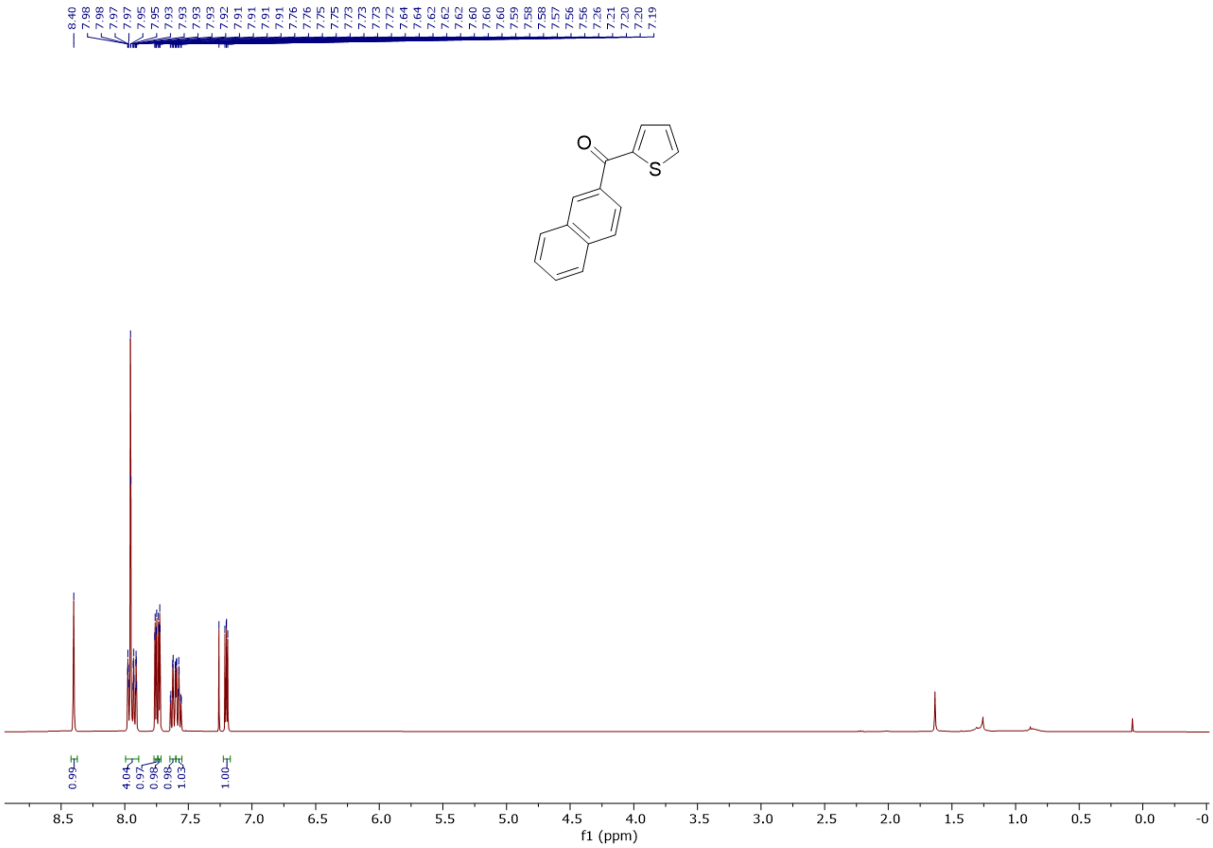

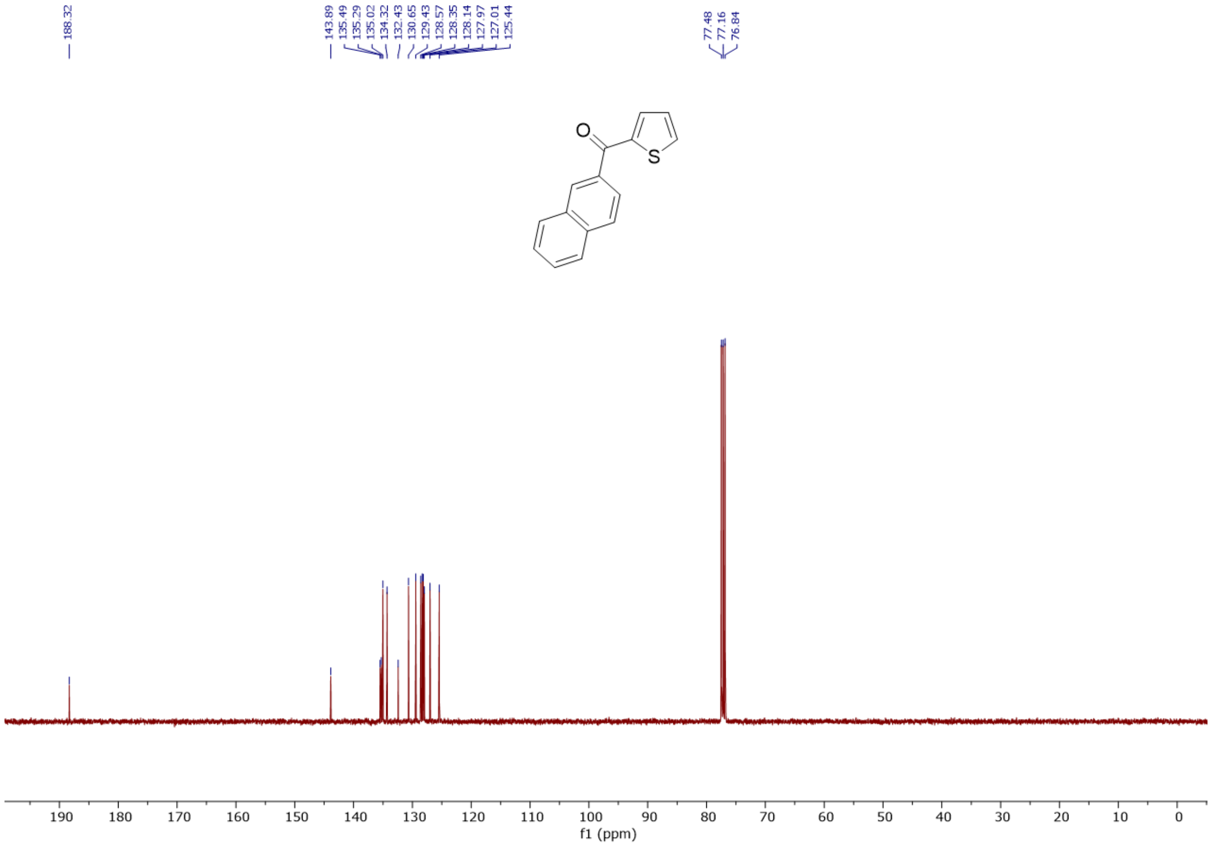

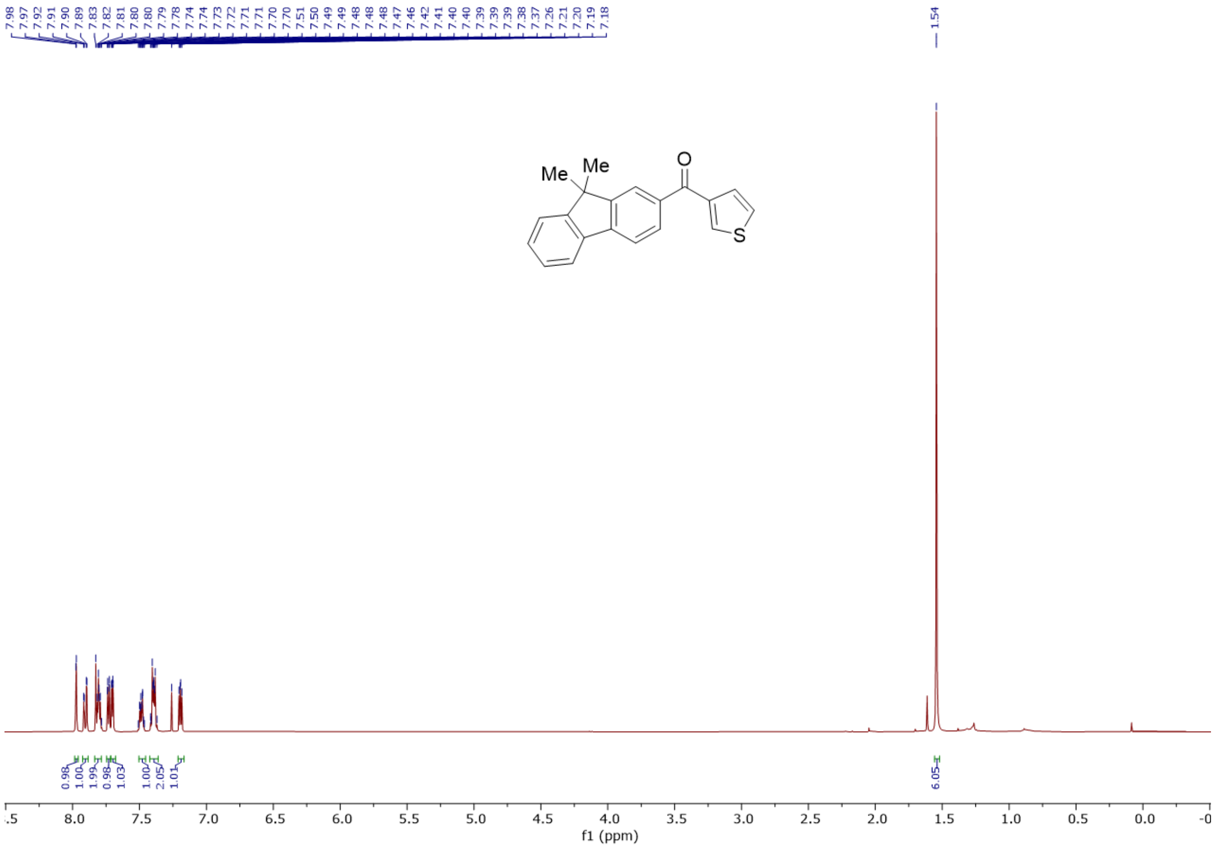
7.98 7.97 7.92 7.91 7.90 7.89 7.83 7.82 7.81 7.80 7.79 7.78 7.74 7.74 7.73 7.72 7.71 7.71 7.70 7.51 7.50 7.49 7.49 7.48 7.48 7.48 7.47 7.46 7.42 7.41 7.40 7.40 7.39 7.39 7.39 7.38 7.37 7.26 7.21 7.20 7.19 7.18
1.54
Me
Me
O
S
0.98 1.00 1.99 0.98 1.03 1.00 2.05 1.01
6.05
f1 (ppm)

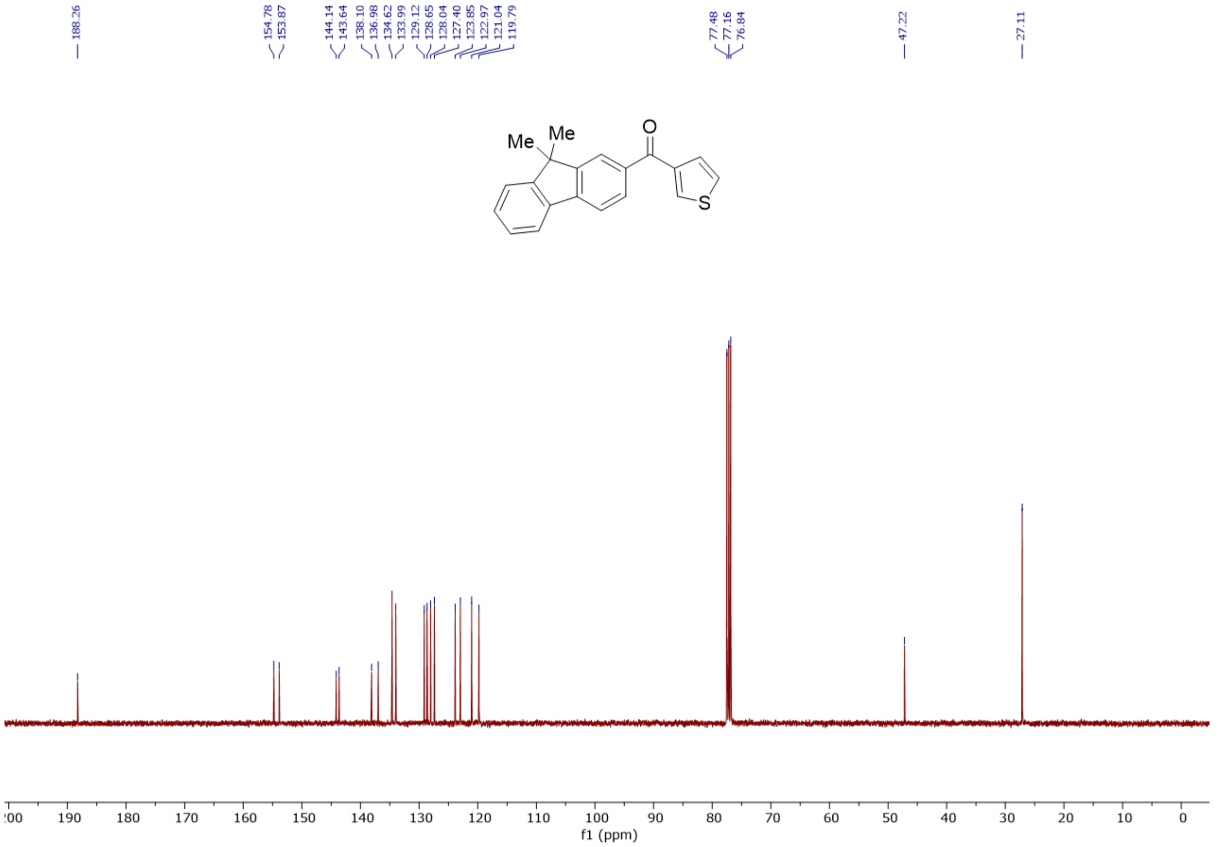
188.26
154.78 153.87
144.14 143.64 138.10 136.98 134.62 133.99 129.12 128.65 128.04 127.40 123.85 122.97 121.04 119.79
77.48 77.16 76.84
47.22
27.11
Me
Me
O
S
f1 (ppm)

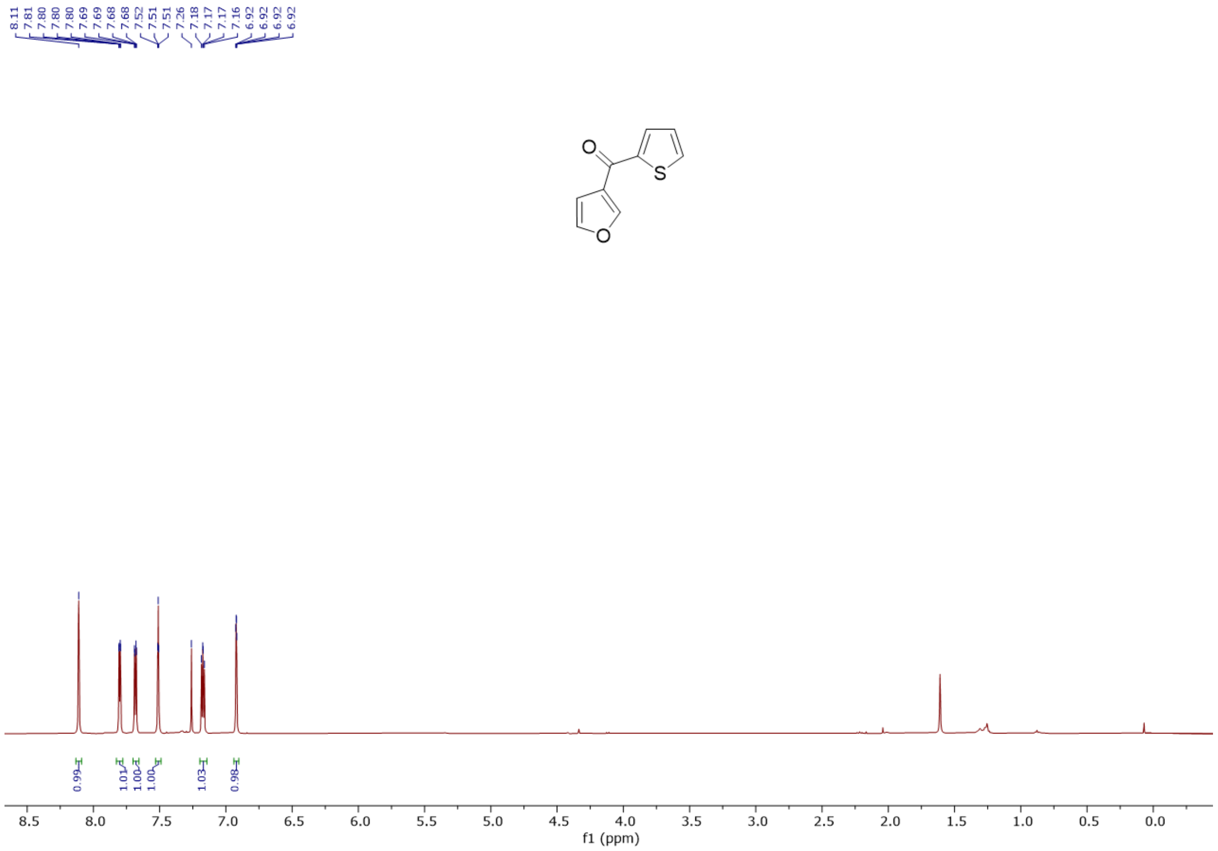
8.11
7.81
7.80
7.80
7.80
7.69
7.69
7.68
7.68
7.52
7.51
7.51
7.26
7.18
7.17
7.17
7.16
6.92
6.92
6.92
6.92
O
S
O
0.99
1.01
1.00
1.00
1.03
0.98
f1 (ppm)

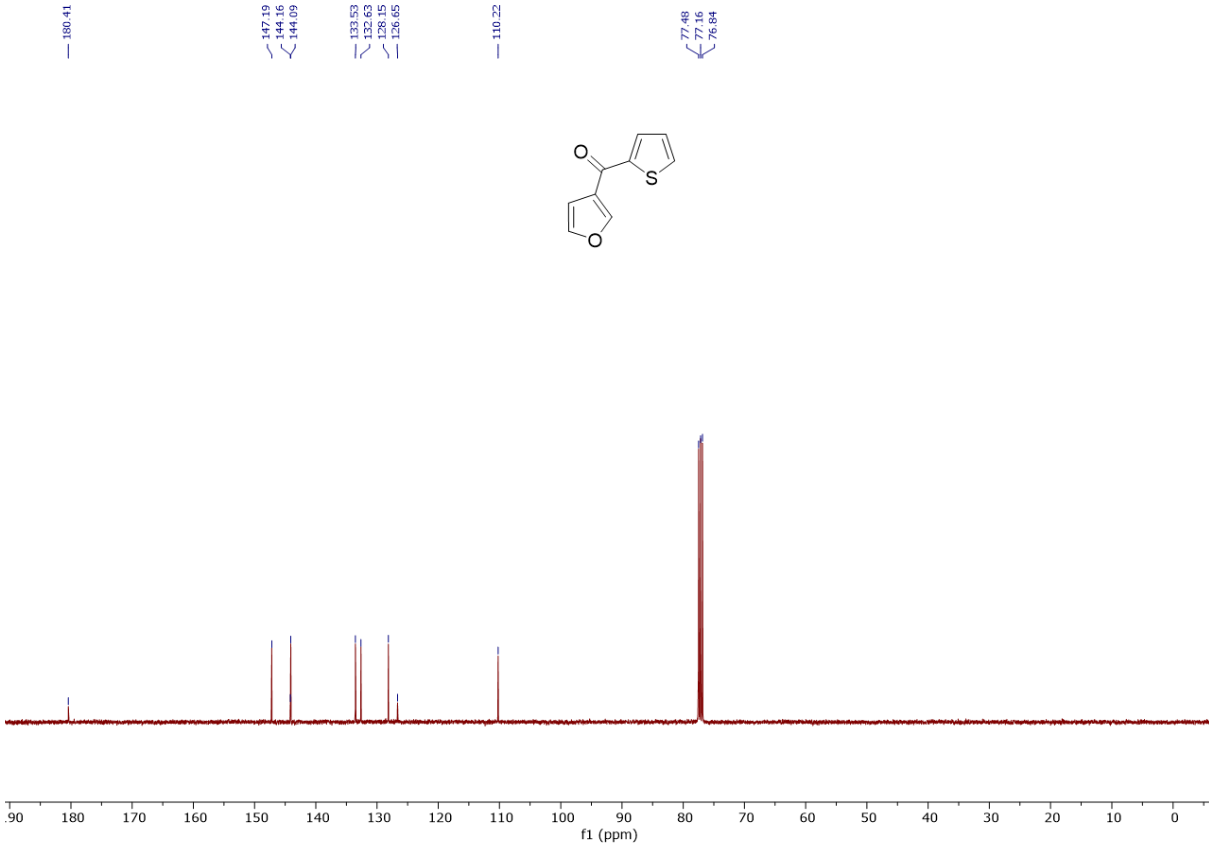
180.41
147.19
144.16
144.09
133.53
132.63
128.15
126.65
110.22
77.48
77.16
76.84
O
S
O
f1 (ppm)

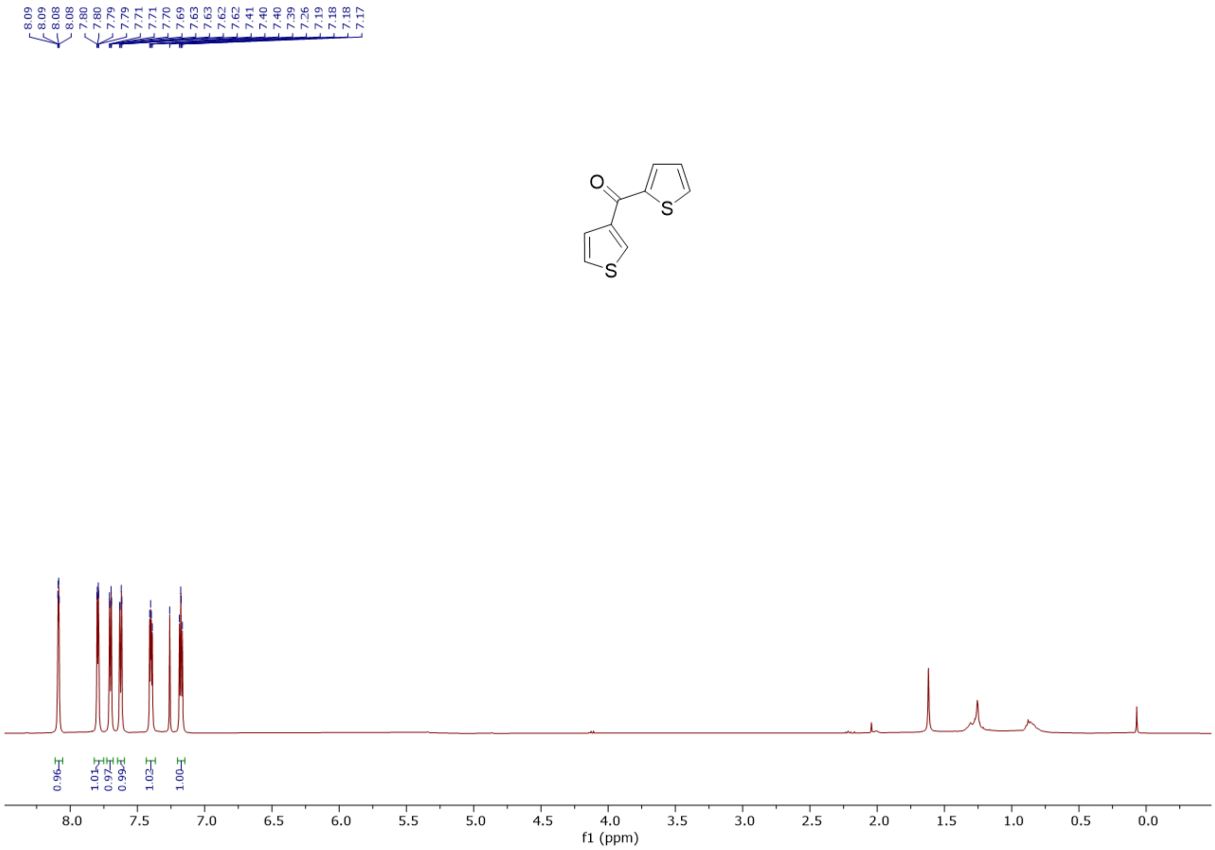
8.09
8.09
8.08
8.08
7.80
7.80
7.79
7.79
7.71
7.71
7.70
7.69
7.63
7.63
7.62
7.62
7.41
7.40
7.40
7.39
7.26
7.19
7.18
7.18
7.17
O
S
S
0.96
1.01
0.97
0.99
1.02
1.00
8.0
7.5
7.0
6.5
6.0
5.5
5.0
4.5
4.0
3.5
3.0
2.5
2.0
1.5
1.0
0.5
0.0
f1 (ppm)

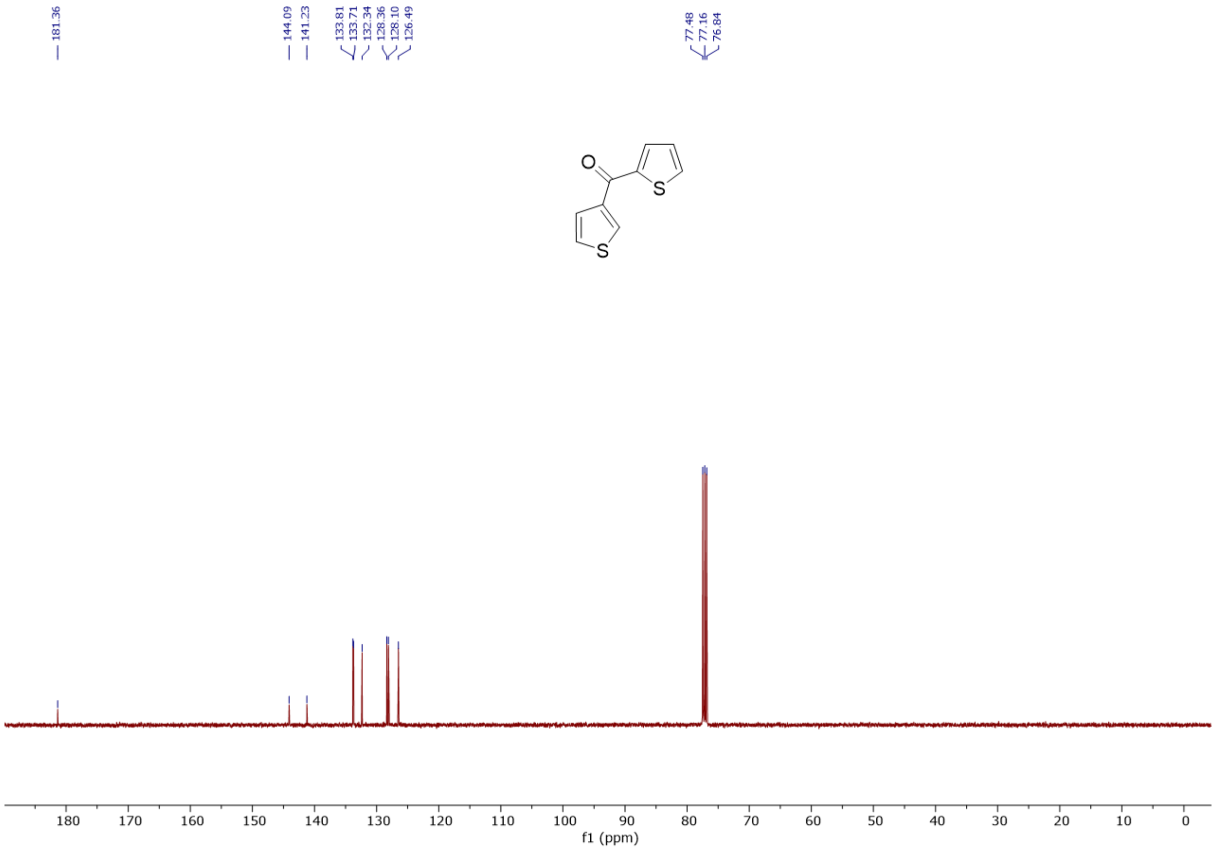
181.36
144.09
141.23
133.81
133.71
132.34
128.36
128.10
126.49
77.48
77.16
76.84
O
S
S
180
170
160
150
140
130
120
110
100
90
80
70
60
50
40
30
20
10
0
f1 (ppm)

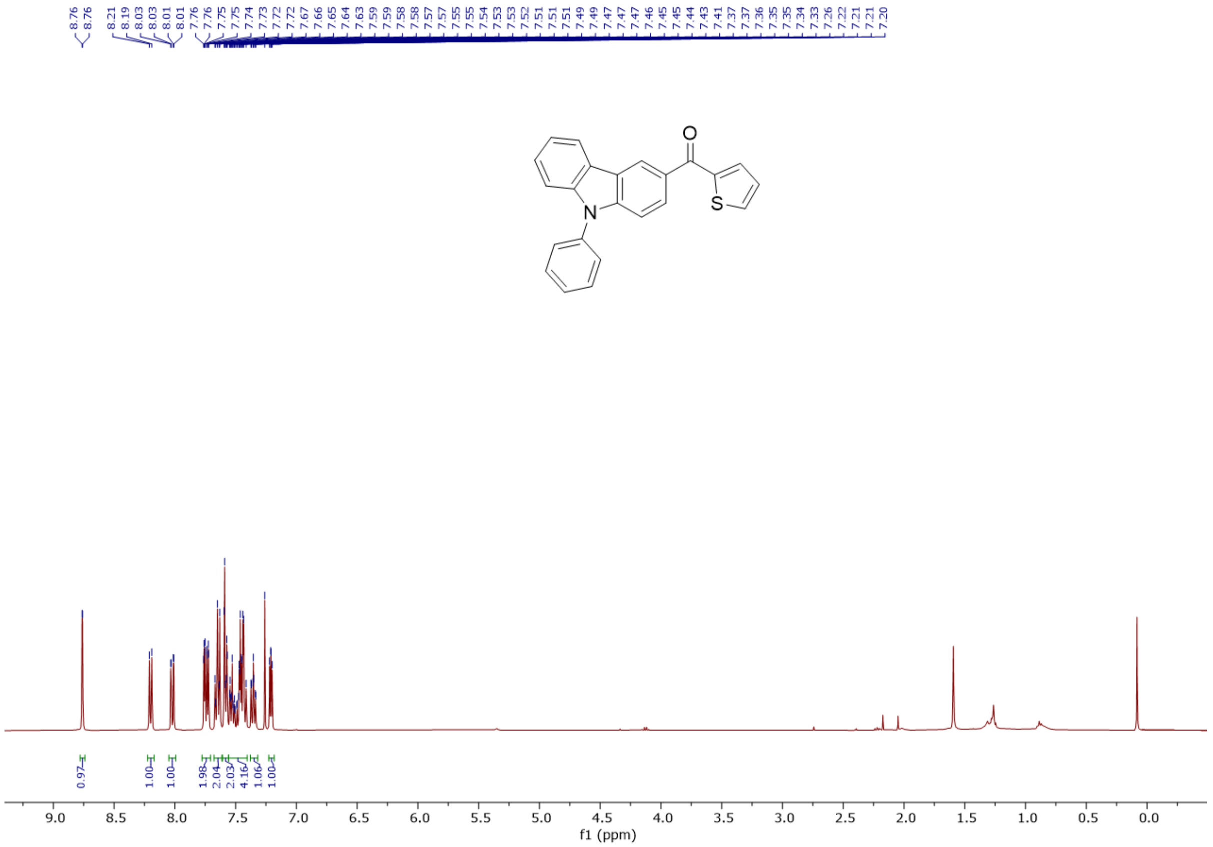
8.76
8.76
8.21
8.19
8.03
8.03
8.01
8.01
7.76
7.76
7.75
7.75
7.74
7.73
7.72
7.72
7.67
7.66
7.65
7.64
7.63
7.59
7.59
7.58
7.58
7.57
7.57
7.55
7.55
7.54
7.53
7.53
7.52
7.51
7.51
7.51
7.49
7.49
7.47
7.47
7.46
7.45
7.45
7.44
7.43
7.41
7.37
7.37
7.36
7.35
7.35
7.34
7.33
7.26
7.22
7.21
7.21
7.20
O
N
S
0.97
1.00
1.00
1.98
2.04
2.03
4.16
1.06
1.00
9.0 8.5 8.0 7.5 7.0 6.5 6.0 5.5 5.0 4.5 4.0 3.5 3.0 2.5 2.0 1.5 1.0 0.5 0.0
f1 (ppm)

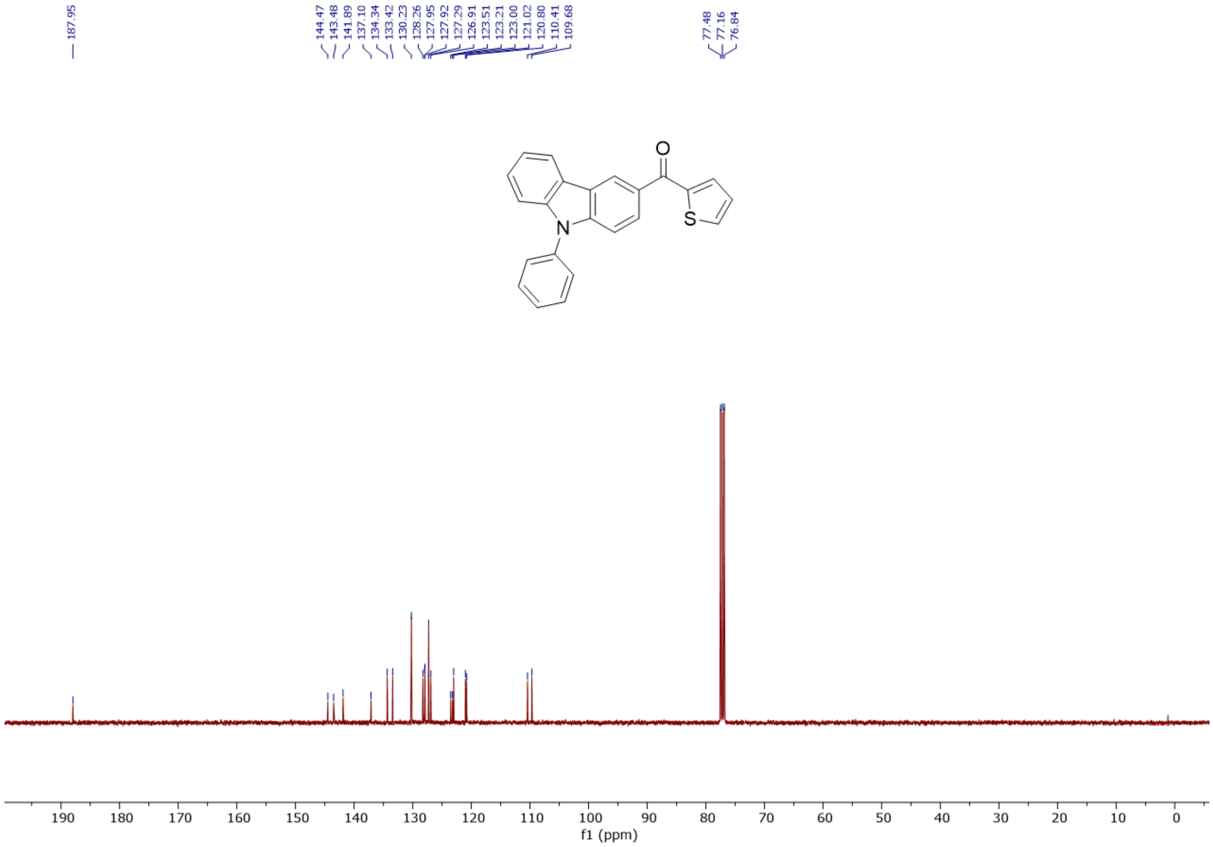
187.95
144.47
143.48
141.89
137.10
134.34
133.42
130.23
128.26
127.95
127.92
127.29
126.91
123.51
123.21
123.00
121.02
120.80
110.41
109.68
77.48
77.16
76.84
O
N
S
190 180 170 160 150 140 130 120 110 100 90 80 70 60 50 40 30 20 10 0
f1 (ppm)

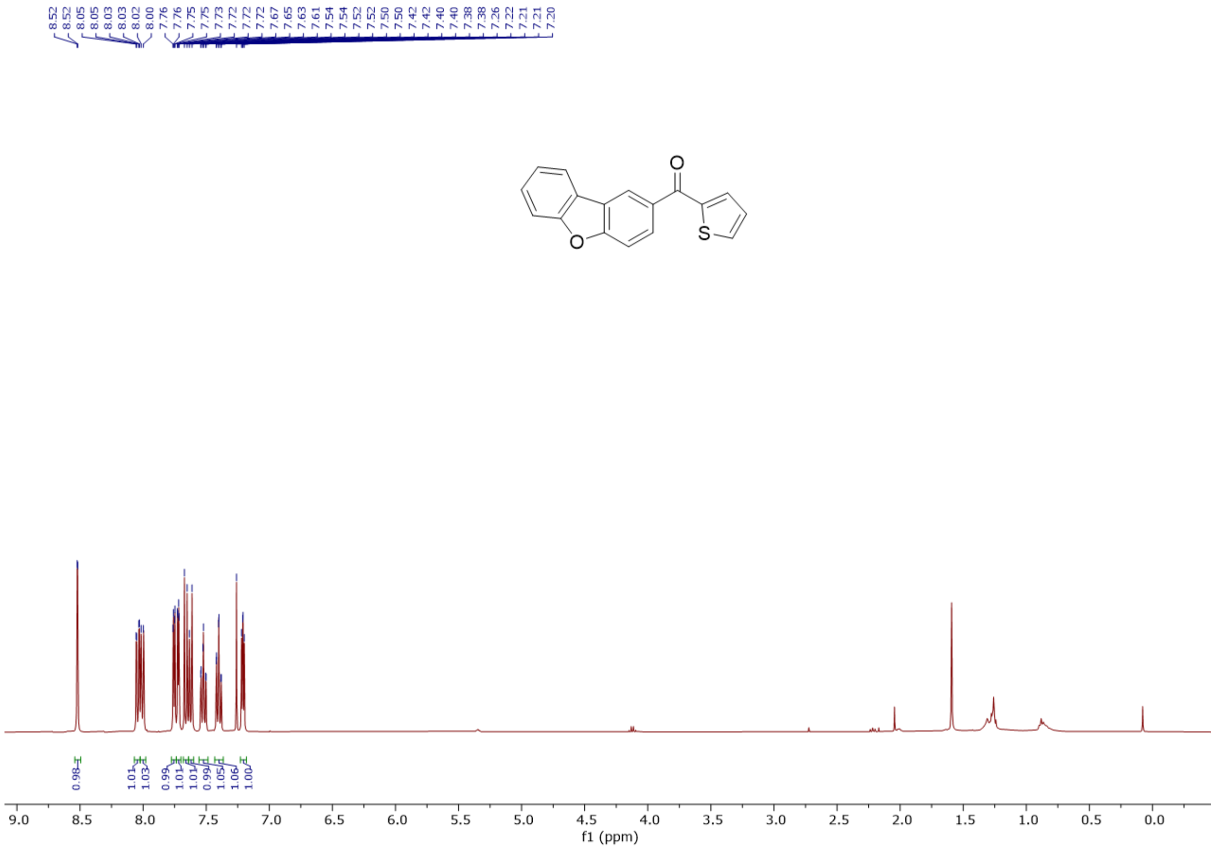
8.52 8.52 8.05 8.05 8.03 8.03 8.02 8.00 7.76 7.76 7.75 7.75 7.73 7.72 7.72 7.72 7.67 7.65 7.63 7.61 7.54 7.54 7.52 7.52 7.50 7.50 7.42 7.42 7.40 7.38 7.38 7.26 7.22 7.21 7.21 7.20
O
S
0.98
1.01
1.03
0.99
1.01
1.01
0.99
1.05
1.06
1.00
9.0 8.5 8.0 7.5 7.0 6.5 6.0 5.5 5.0 4.5 4.0 3.5 3.0 2.5 2.0 1.5 1.0 0.5 0.0
f1 (ppm)

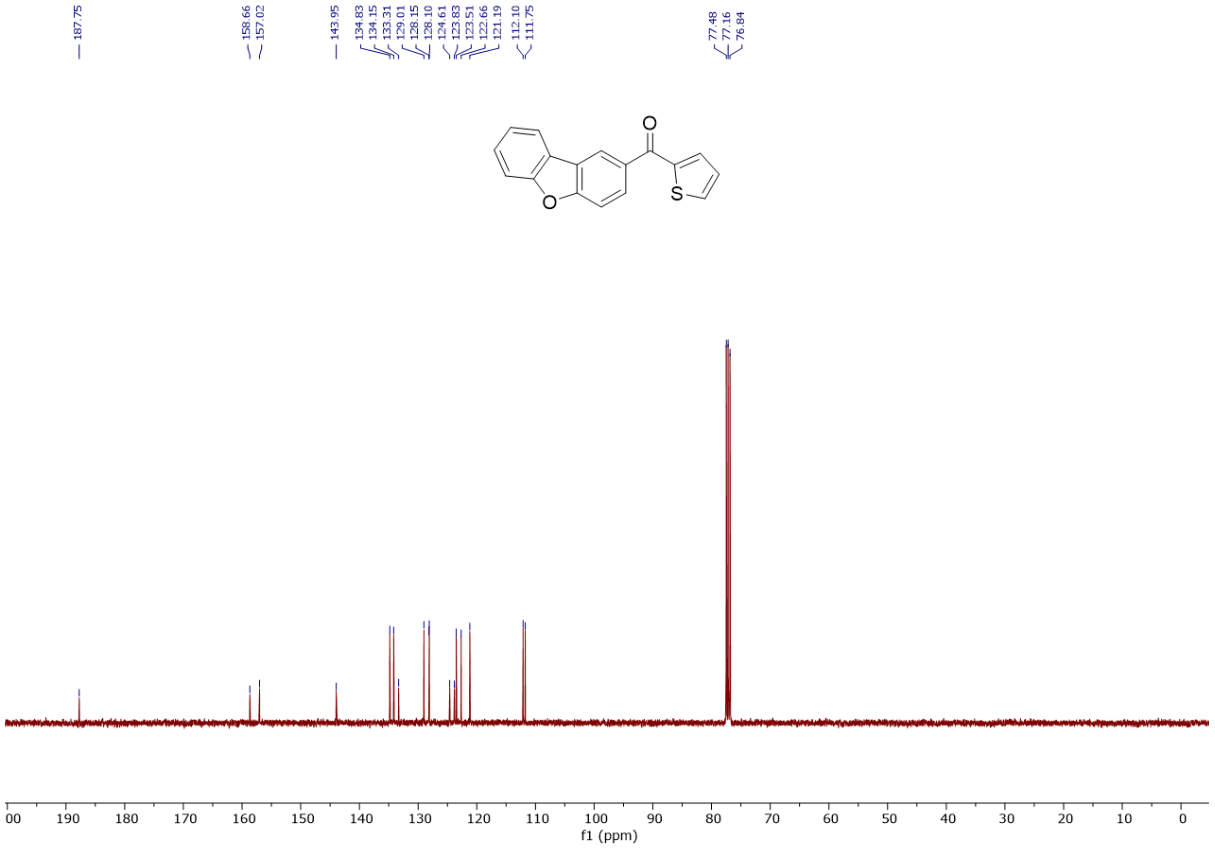
187.75
158.66 157.02
143.95
134.83 134.15 133.31 129.01 128.15 128.10 124.61 123.83 123.51 122.66 121.19
112.10 111.75
77.48 77.16 76.84
O
S
00 190 180 170 160 150 140 130 120 110 100 90 80 70 60 50 40 30 20 10 0
f1 (ppm)

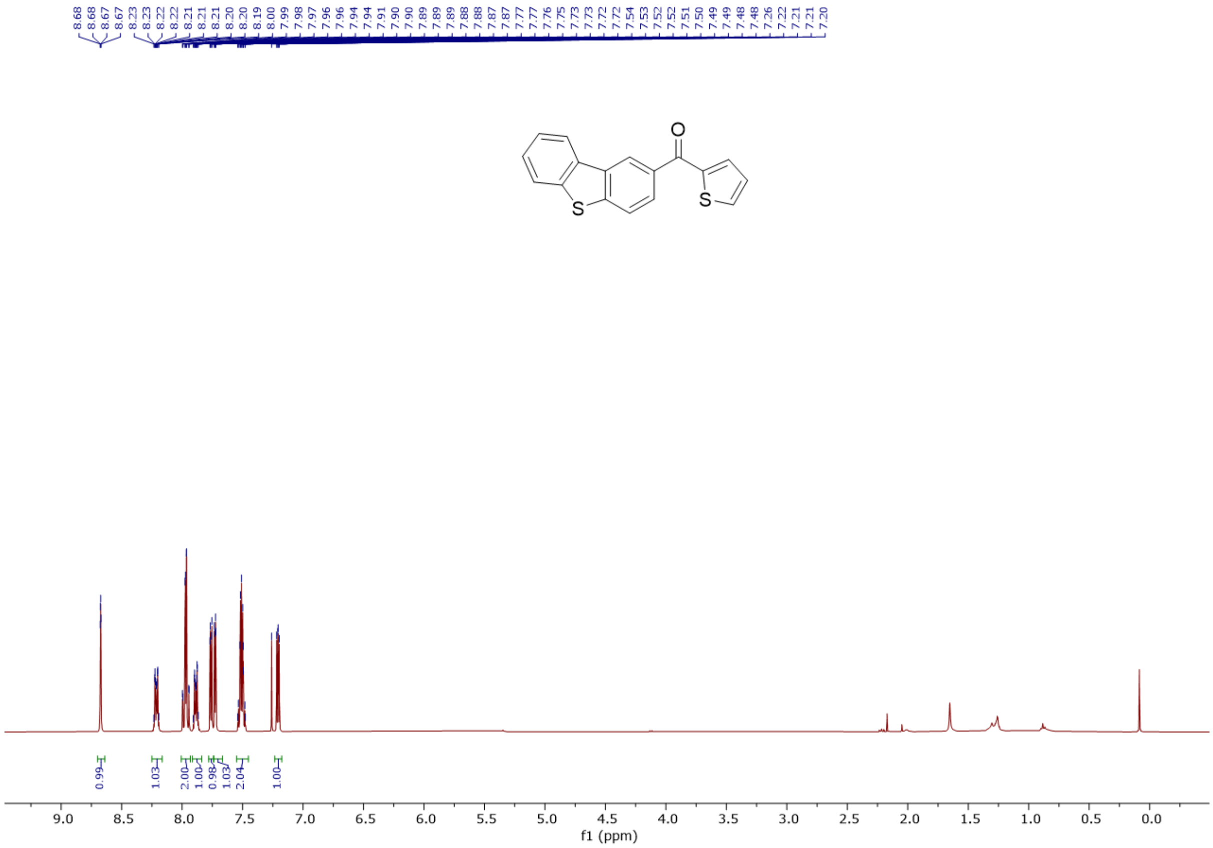
8.68
8.68
8.67
8.67
8.23
8.23
8.22
8.22
8.21
8.21
8.21
8.20
8.20
8.19
8.00
7.99
7.98
7.97
7.96
7.96
7.94
7.94
7.91
7.90
7.90
7.89
7.89
7.89
7.88
7.88
7.87
7.87
7.77
7.77
7.76
7.75
7.73
7.73
7.72
7.72
7.54
7.53
7.52
7.52
7.51
7.50
7.49
7.49
7.48
7.48
7.26
7.22
7.21
7.21
7.20
O
S
S
0.99
1.03
2.00
1.00
0.98
1.03
2.04
1.00
9.0
8.5
8.0
7.5
7.0
6.5
6.0
5.5
5.0
4.5
4.0
3.5
3.0
2.5
2.0
1.5
1.0
0.5
0.0
f1 (ppm)

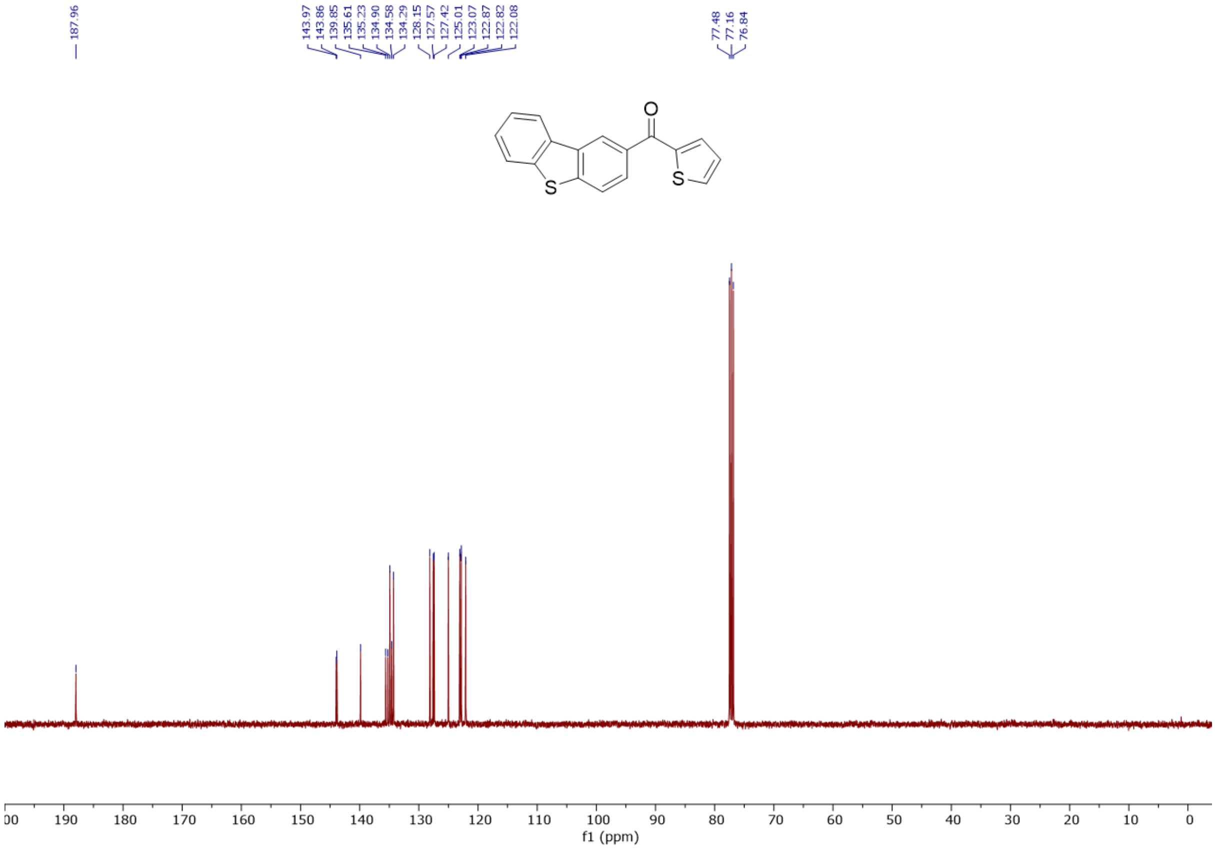
187.96
143.97
143.86
139.85
135.61
135.23
134.90
134.58
134.29
128.15
127.57
127.42
125.01
123.07
122.87
122.82
122.08
77.48
77.16
76.84
O
S
S
00
190
180
170
160
150
140
130
120
110
100
90
80
70
60
50
40
30
20
10
0
f1 (ppm)

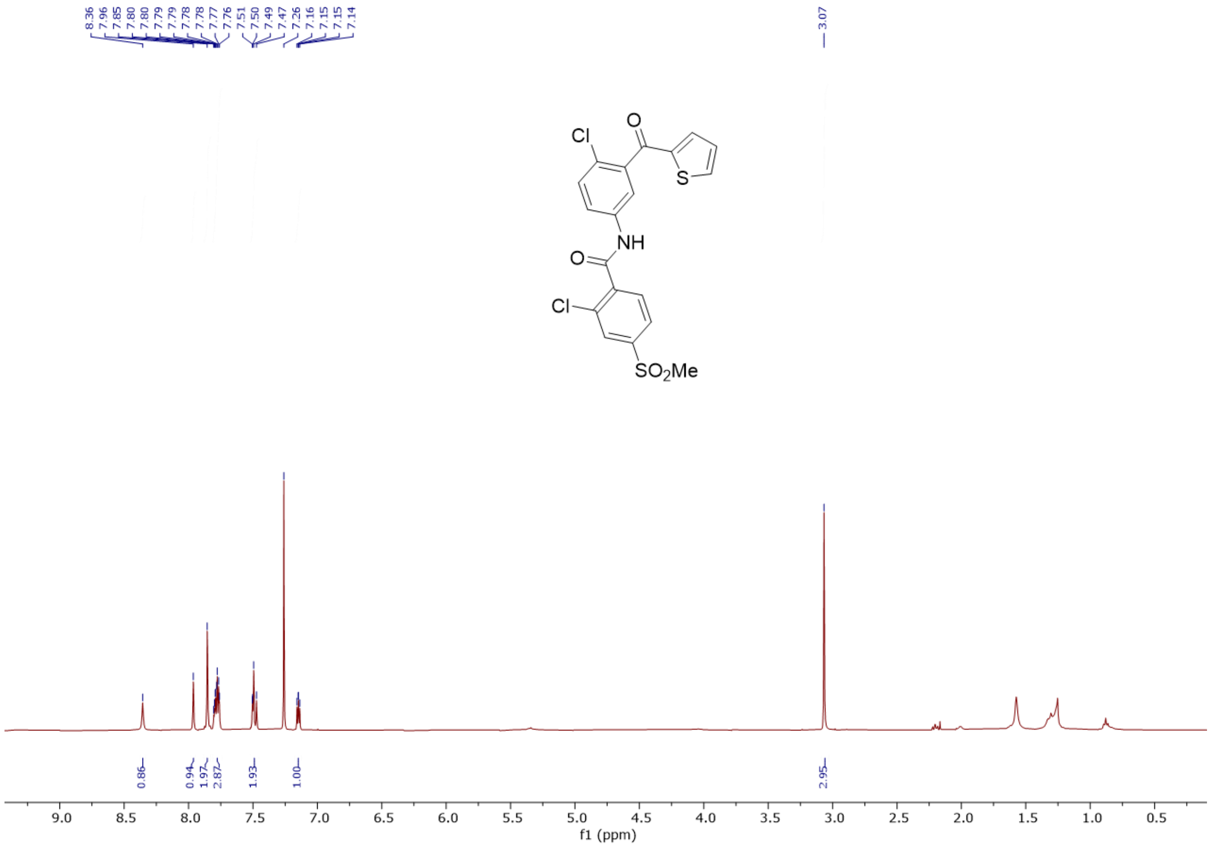

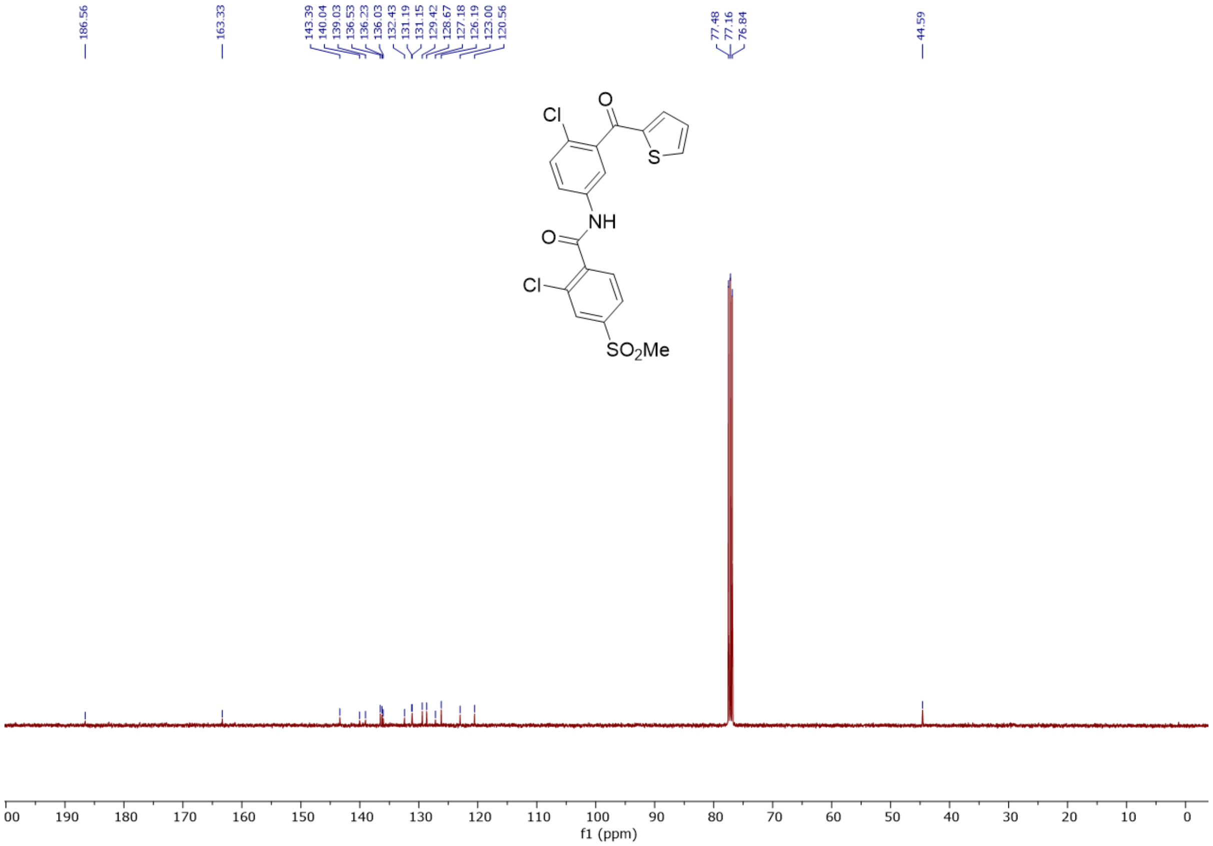

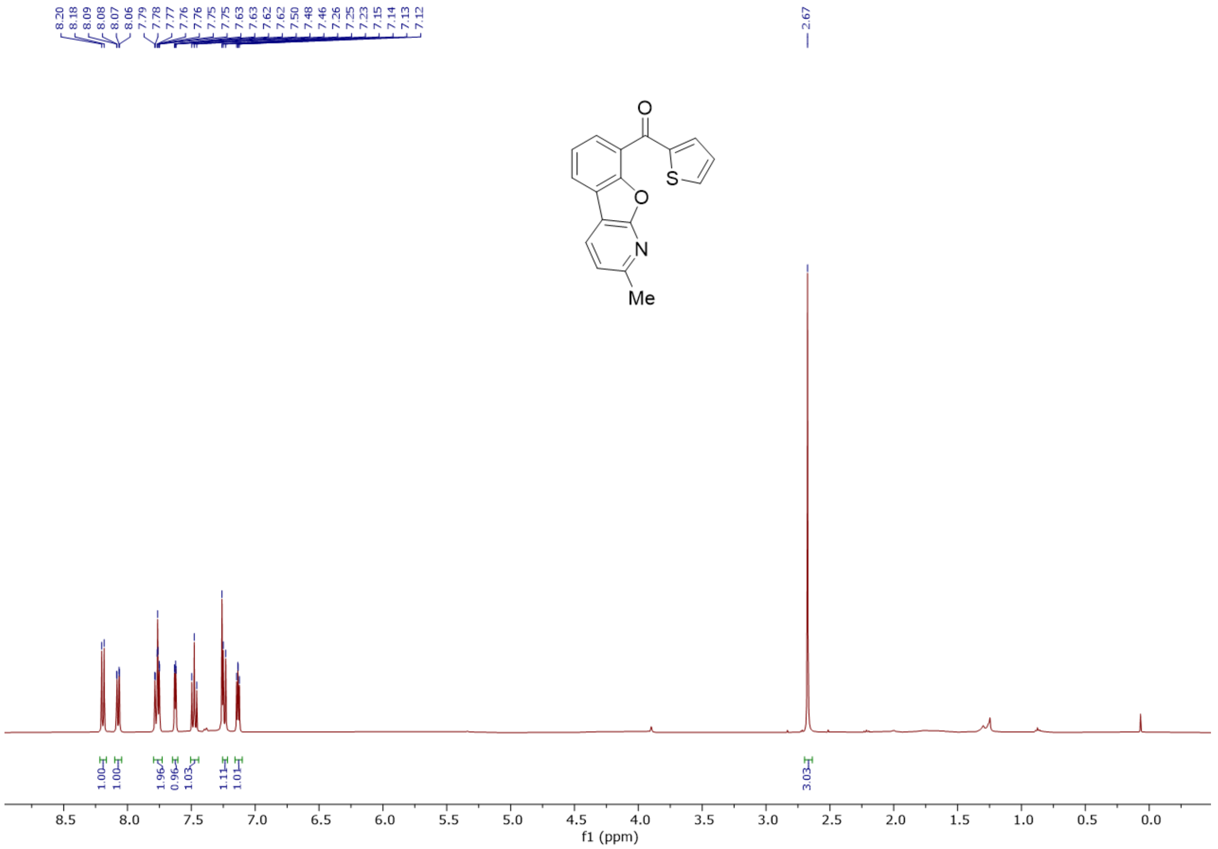
8.20
8.18
8.09
8.08
8.07
8.06
7.79
7.78
7.77
7.76
7.76
7.75
7.75
7.63
7.63
7.62
7.62
7.50
7.48
7.46
7.26
7.25
7.23
7.15
7.14
7.13
7.12
2.67
O
S
O
N
Me
1.00
1.00
1.96
0.96
1.03
1.11
1.01
3.03
f1 (ppm)

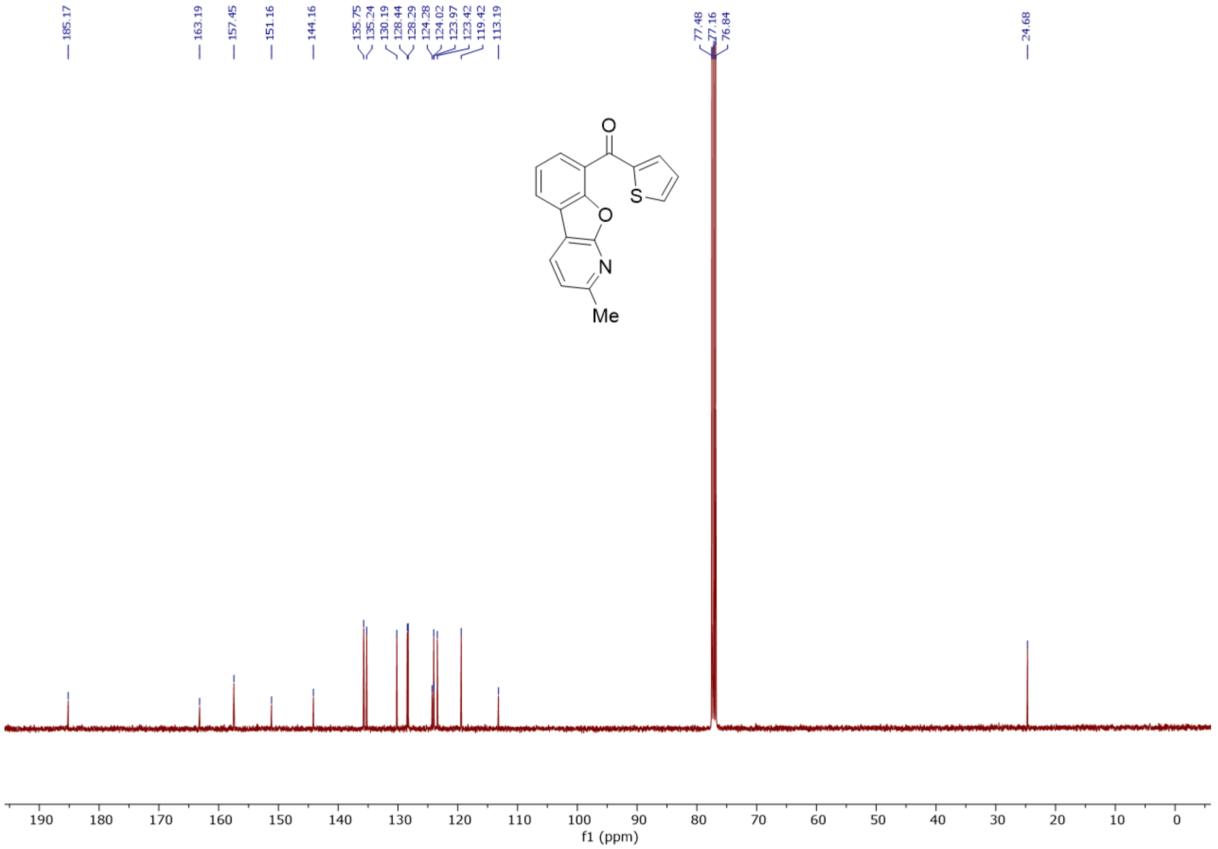
185.17
163.19
157.45
151.16
144.16
135.75
135.24
130.19
128.44
128.29
124.28
124.02
123.97
123.42
119.42
113.19
77.48
77.16
76.84
24.68
O
S
O
N
Me
f1 (ppm)

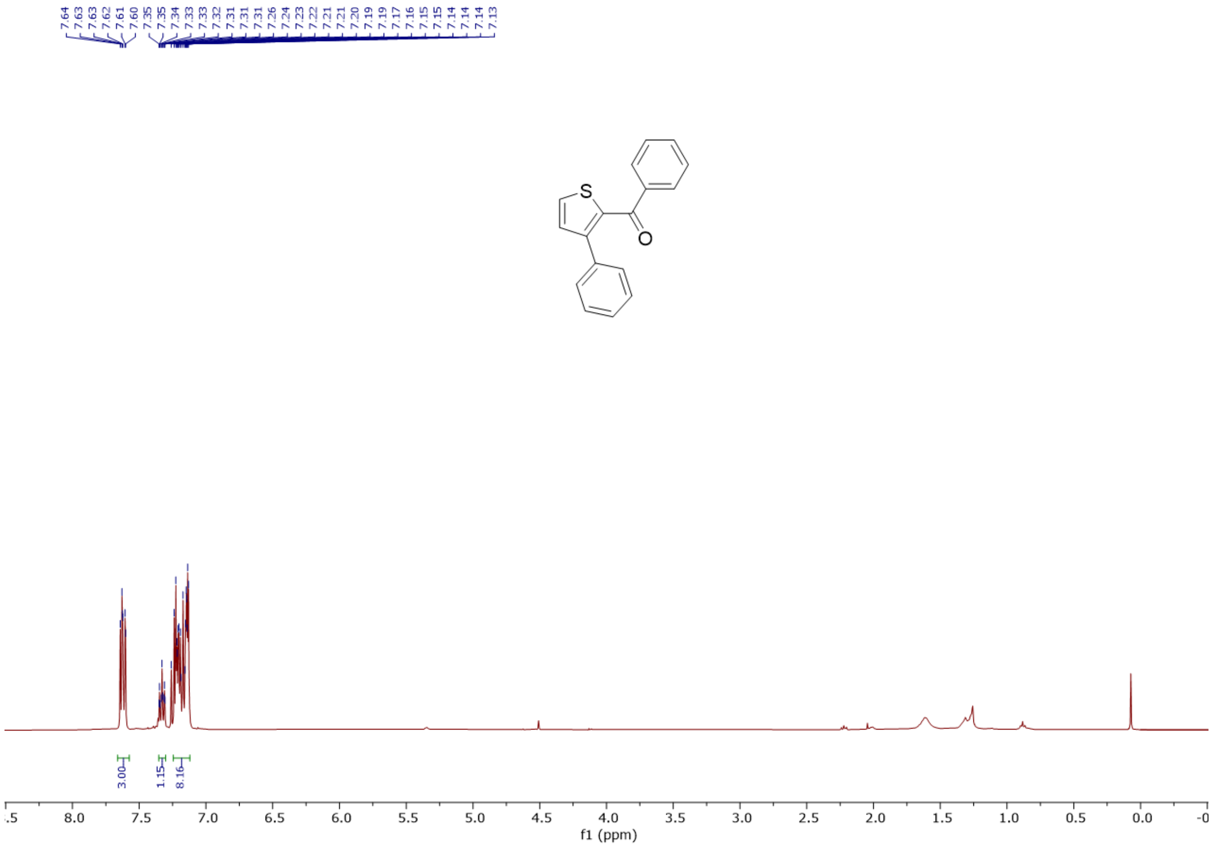
7.64
7.63
7.63
7.62
7.61
7.60
7.35
7.35
7.34
7.33
7.33
7.32
7.31
7.31
7.31
7.26
7.24
7.23
7.22
7.21
7.21
7.20
7.19
7.19
7.17
7.16
7.15
7.15
7.14
7.14
7.14
7.13
S
O
3.00
1.15
8.16
f1 (ppm)

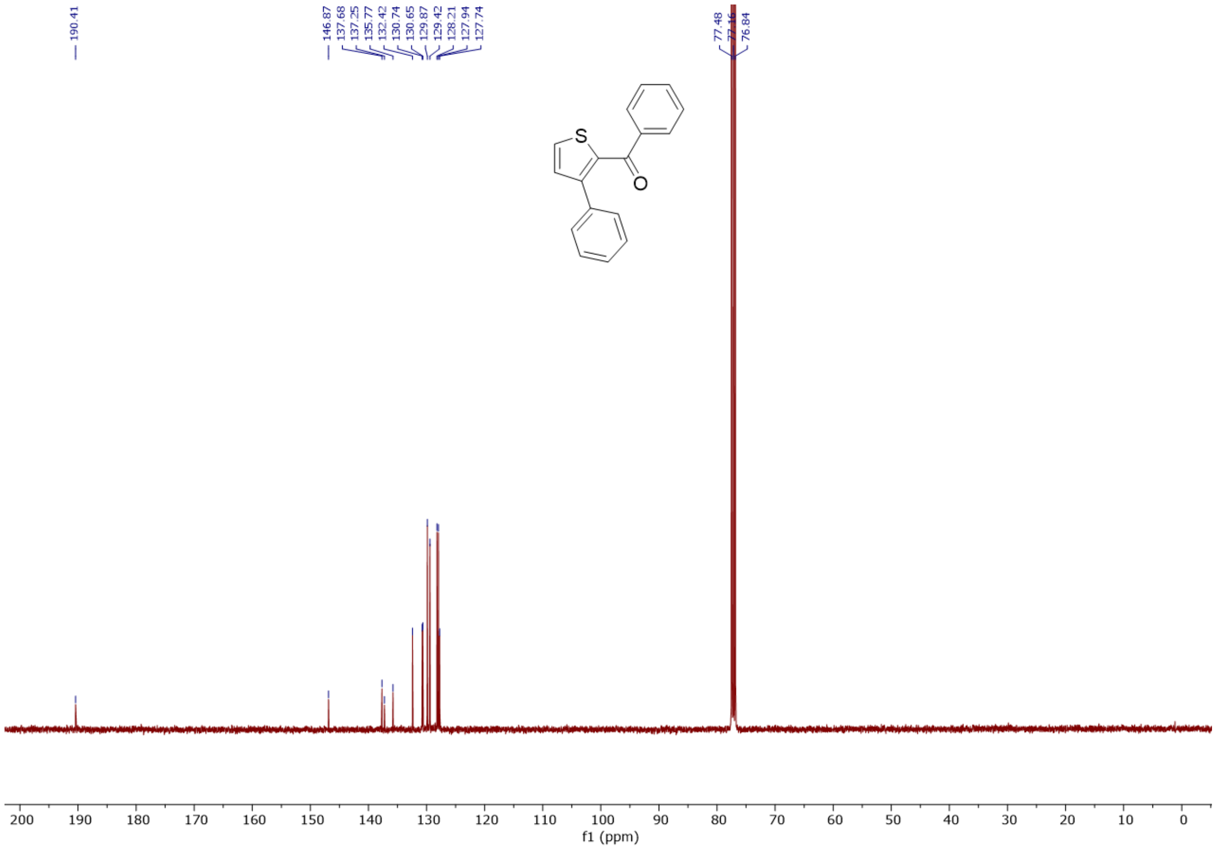
190.41
146.87
137.68
137.25
135.77
132.42
130.74
130.65
129.87
129.42
128.21
127.94
127.74
77.48
77.16
76.84
S
O
f1 (ppm)

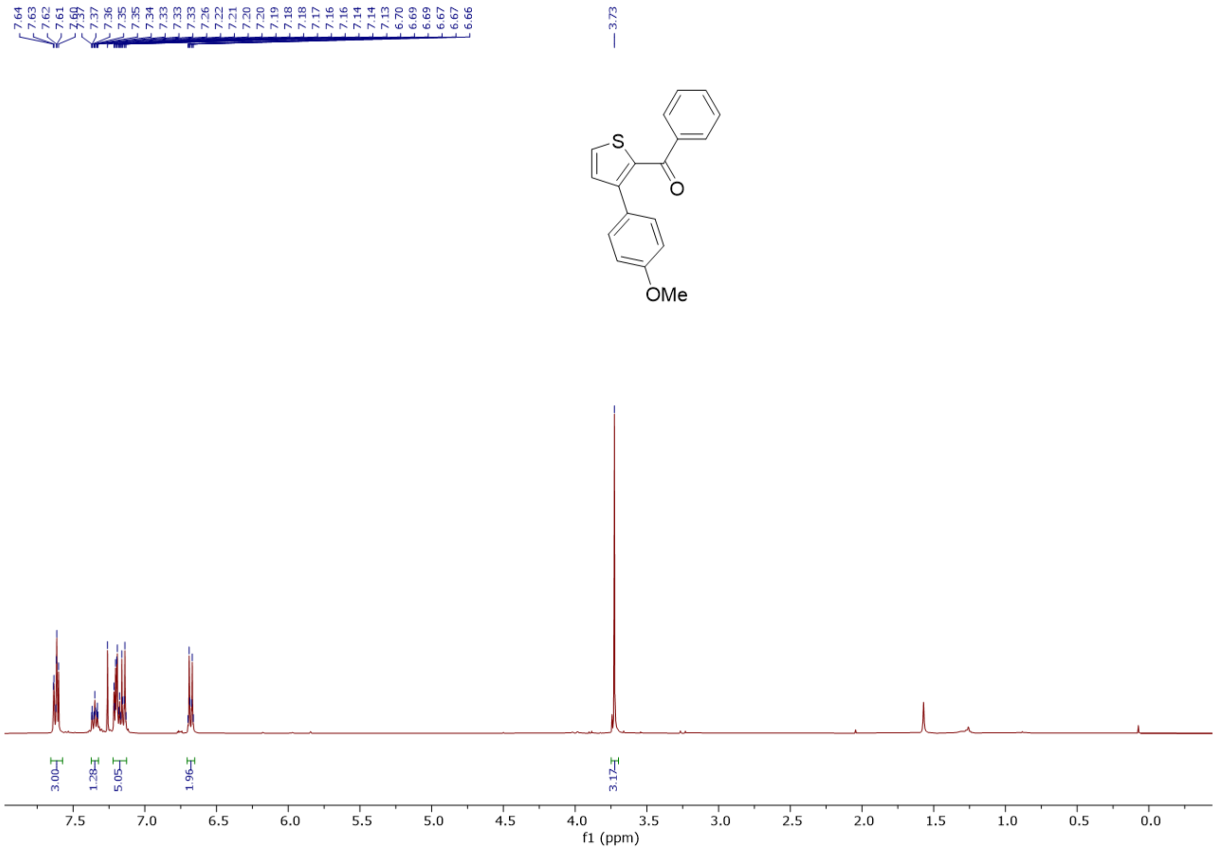
7.64
7.63
7.62
7.61
7.60
7.37
7.36
7.35
7.35
7.34
7.33
7.33
7.33
7.26
7.22
7.21
7.20
7.20
7.19
7.18
7.18
7.17
7.16
7.16
7.14
7.14
7.13
6.70
6.69
6.69
6.67
6.67
6.66
3.73
S
O
OMe
3.00
1.28
5.05
1.96
3.17
f1 (ppm)

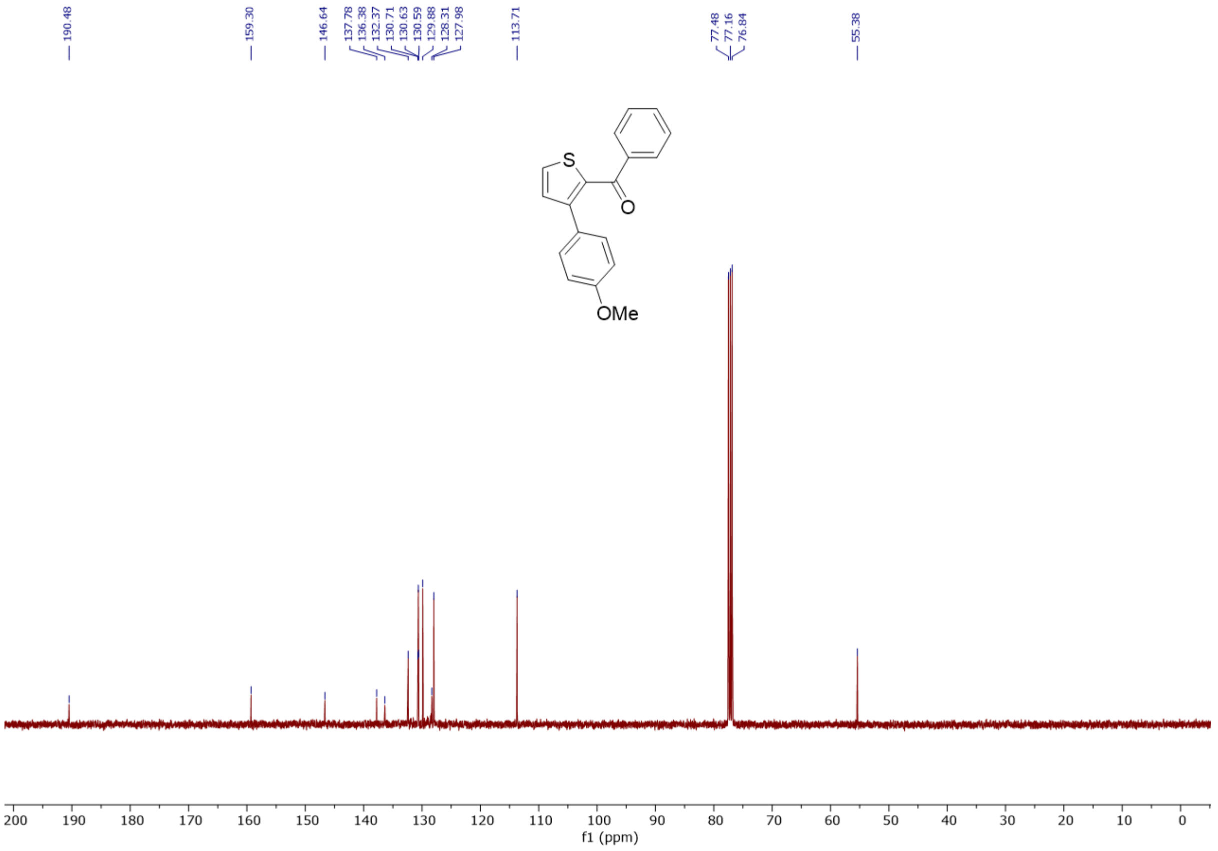
190.48
159.30
146.64
137.78
136.38
132.37
130.71
130.63
130.59
129.88
128.31
127.98
113.71
77.48
77.16
76.84
55.38
S
O
OMe
f1 (ppm)

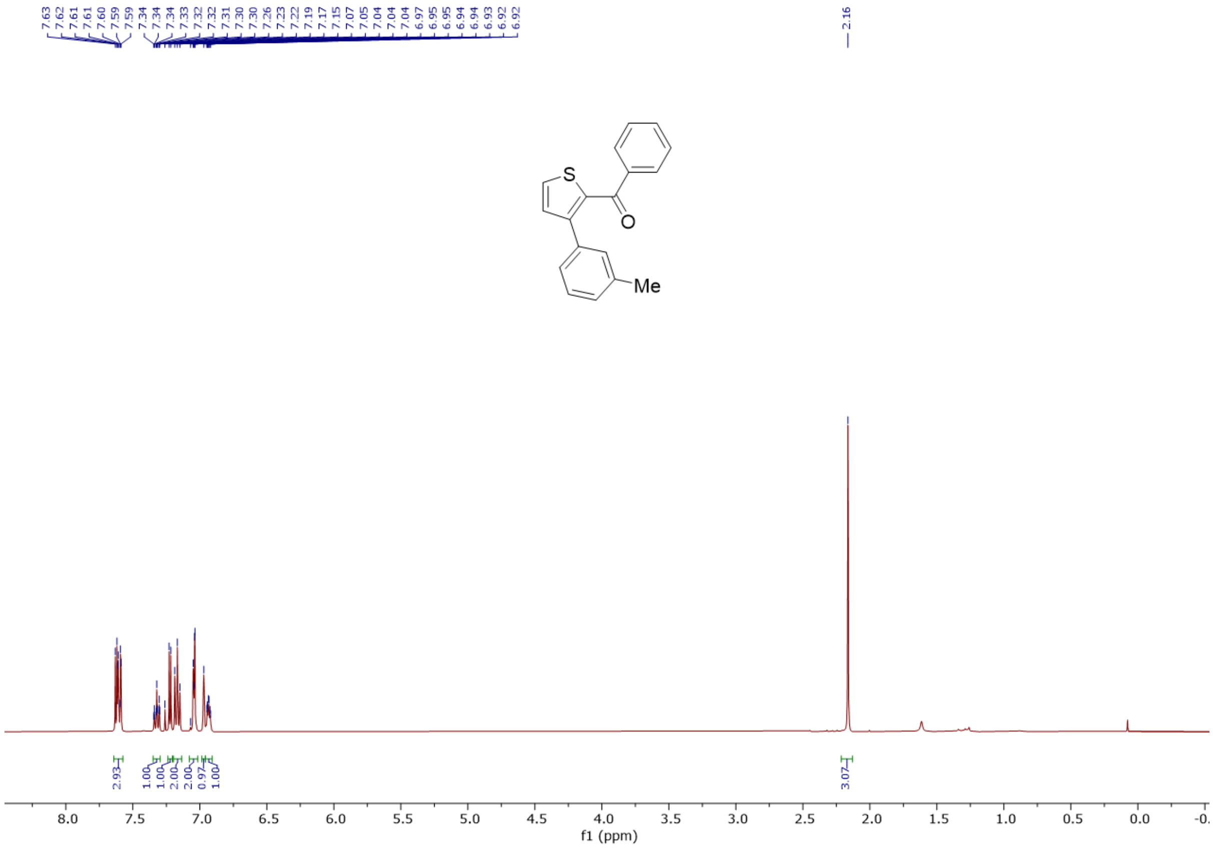

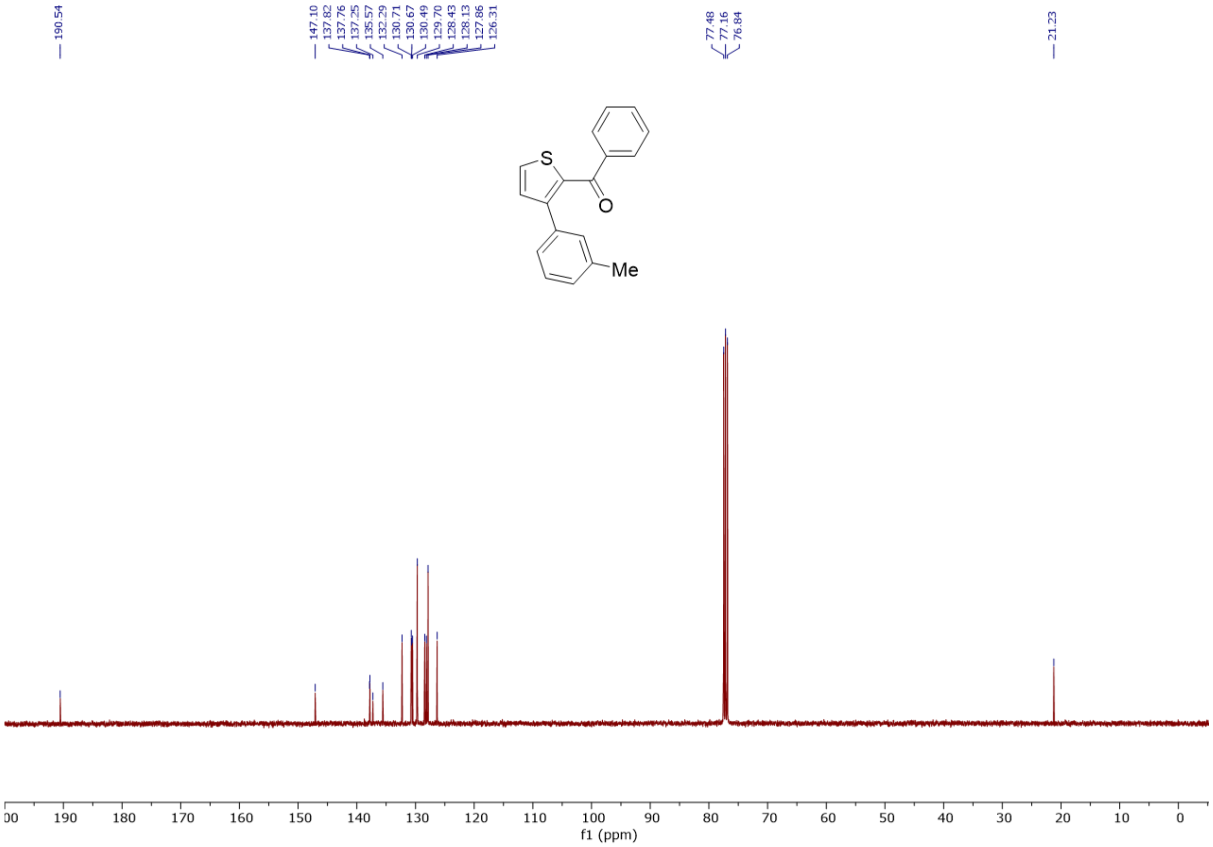

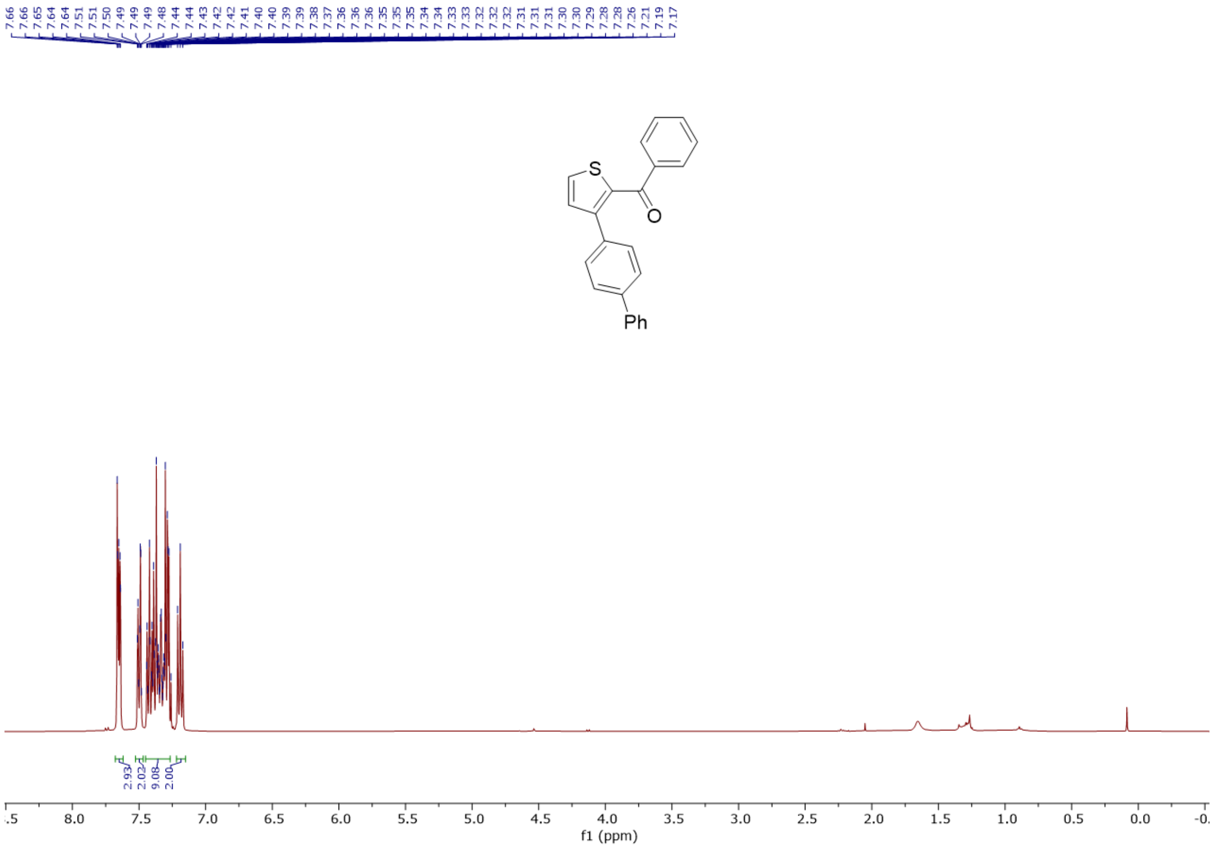
7.66 7.66 7.65 7.64 7.64 7.51 7.51 7.50 7.49 7.49 7.49 7.48 7.44 7.44 7.43 7.42 7.42 7.41 7.40 7.40 7.39 7.39 7.38 7.37 7.36 7.36 7.36 7.35 7.35 7.35 7.34 7.34 7.33 7.33 7.32 7.32 7.32 7.31 7.31 7.31 7.30 7.30 7.29 7.28 7.28 7.26 7.21 7.19 7.17
S
O
Ph
2.93
2.02
9.08
2.00
f1 (ppm)

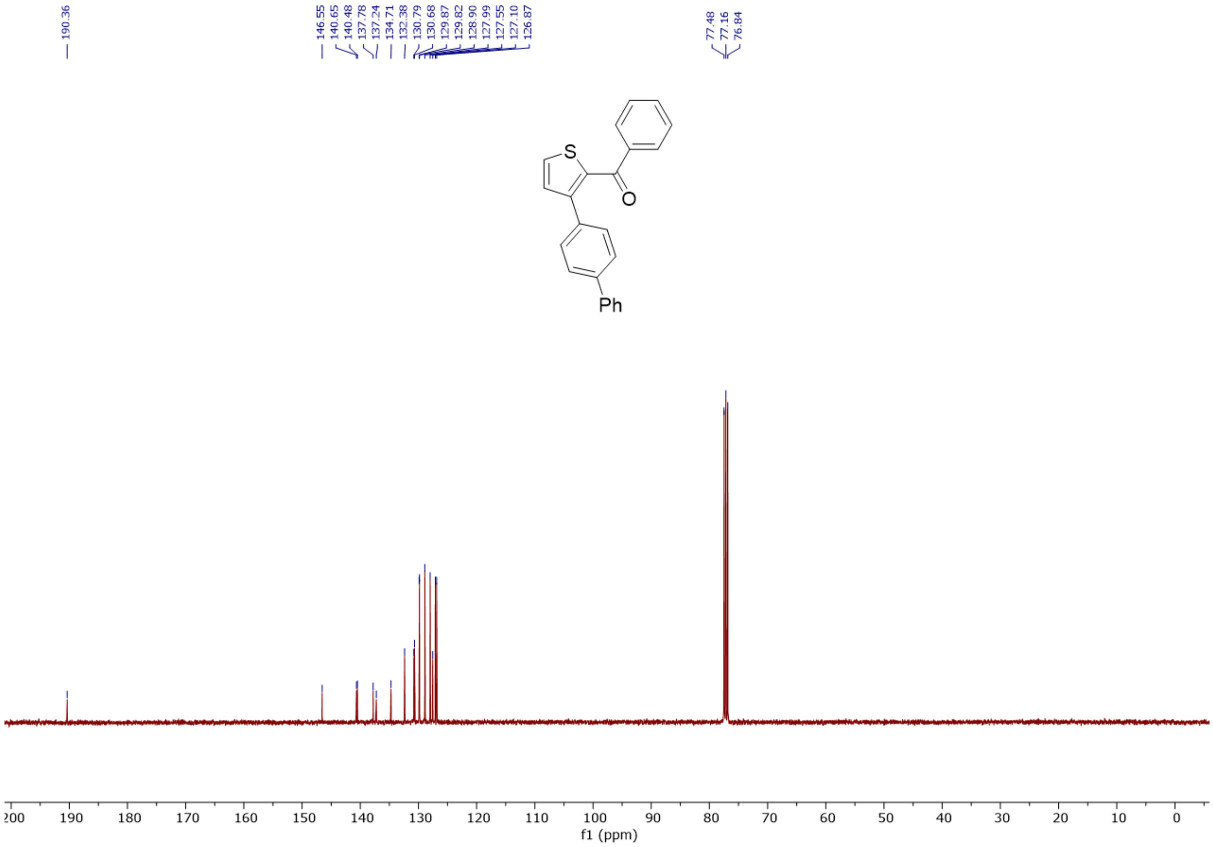
190.36
146.55 140.65 140.48 137.78 137.24 134.71 132.38 130.79 130.68 129.87 129.82 128.90 127.99 127.55 127.10 126.87
77.48 77.16 76.84
S
O
Ph
f1 (ppm)

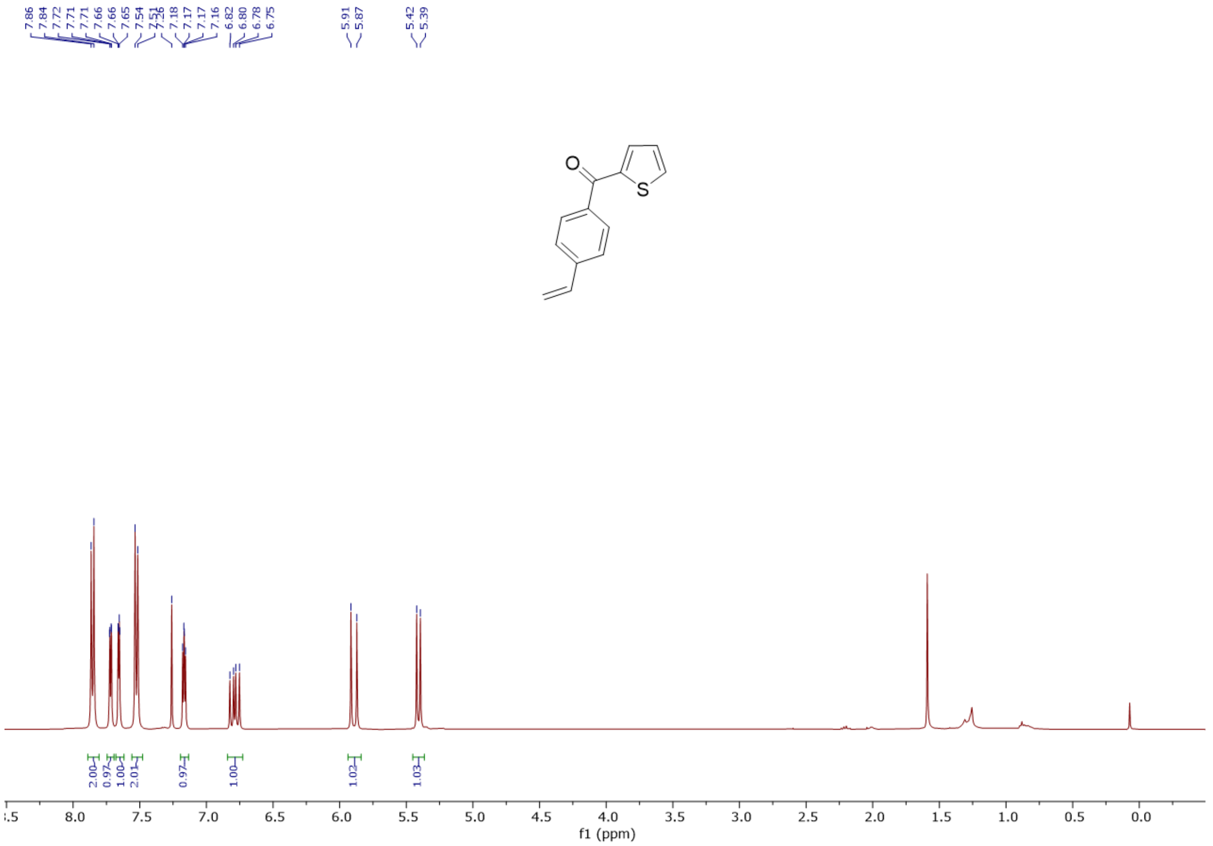
7.86
7.84
7.72
7.71
7.71
7.66
7.66
7.65
7.54
7.51
7.26
7.18
7.17
7.17
7.16
6.82
6.80
6.78
6.75
5.91
5.87
5.42
5.39
O
S
2.00
0.97
1.00
2.01
0.97
1.00
1.02
1.03
8.0
7.5
7.0
6.5
6.0
5.5
5.0
4.5
4.0
3.5
3.0
2.5
2.0
1.5
1.0
0.5
0.0
f1 (ppm)

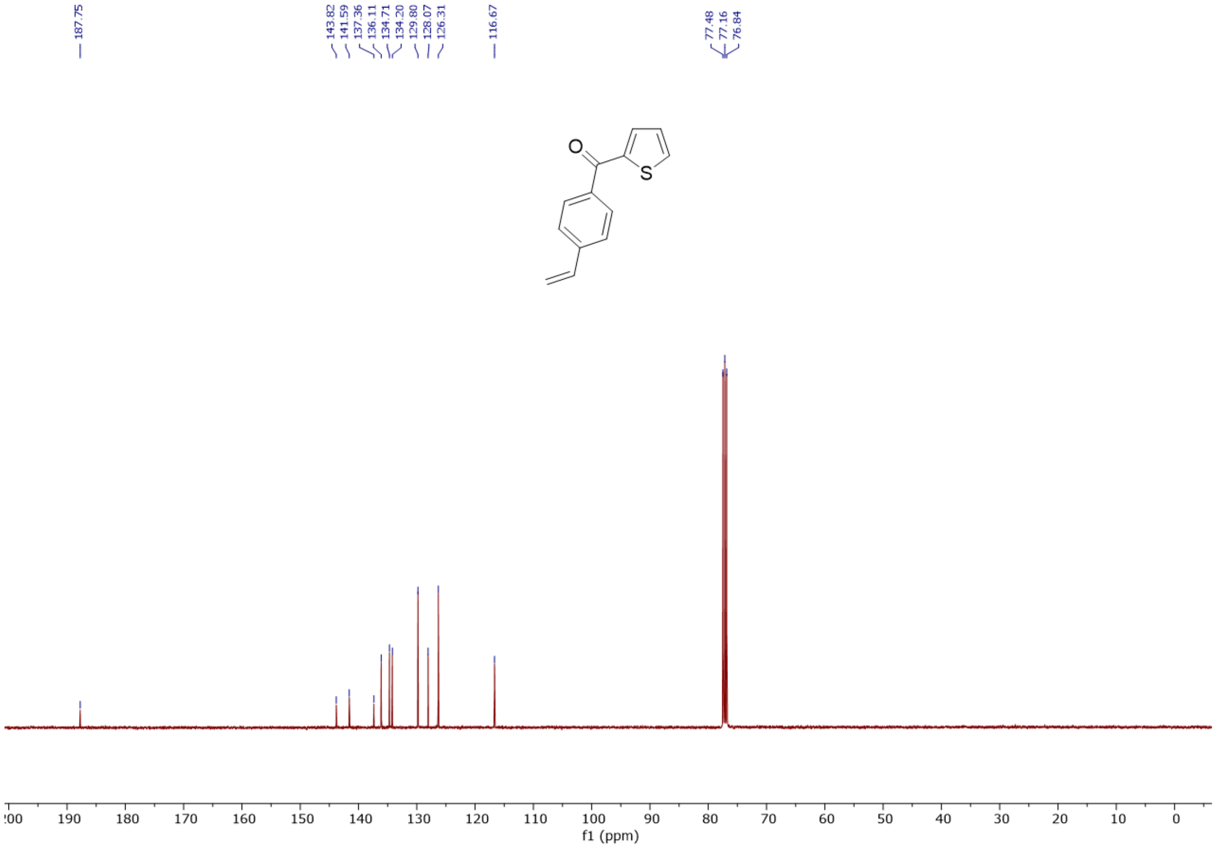
187.75
143.82
141.59
137.36
136.11
134.71
134.20
129.80
128.07
126.31
116.67
77.48
77.16
76.84
O
S
190
180
170
160
150
140
130
120
110
100
90
80
70
60
50
40
30
20
10
0
f1 (ppm)

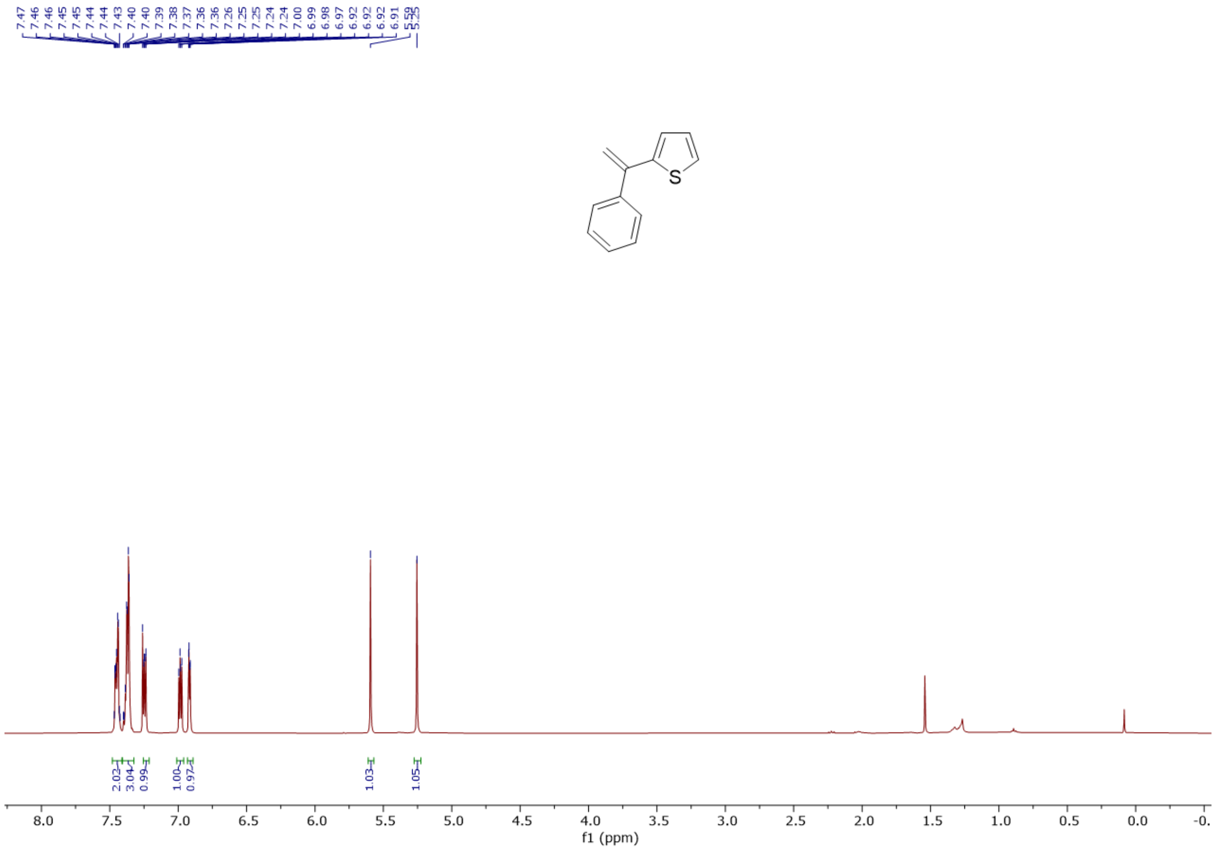
7.47
7.46
7.46
7.45
7.45
7.44
7.44
7.43
7.40
7.40
7.39
7.38
7.37
7.36
7.36
7.26
7.25
7.25
7.24
7.24
7.00
6.99
6.98
6.97
6.92
6.92
6.92
6.91
5.59
5.25
S
2.02
3.04
0.99
1.00
0.97
1.03
1.05
f1 (ppm)

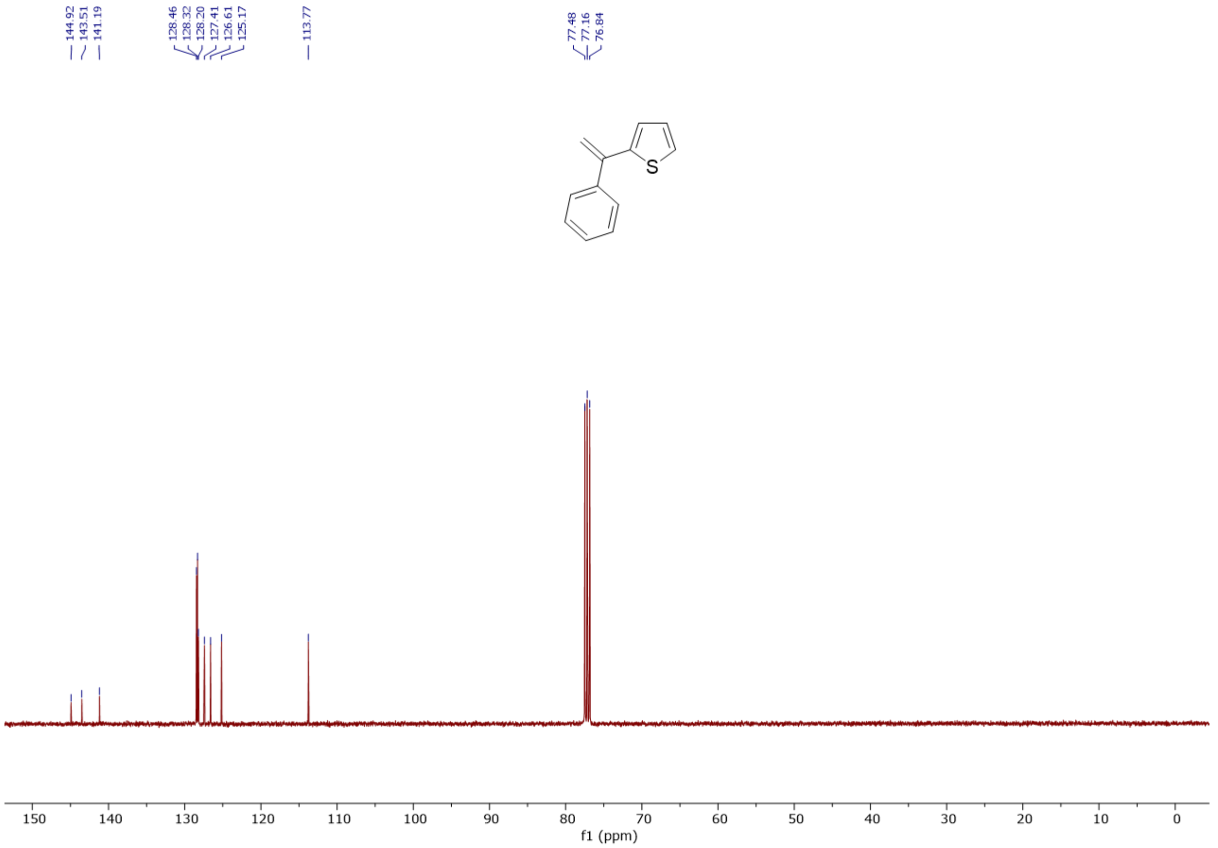
144.92
143.51
141.19
128.46
128.32
128.20
127.41
126.61
125.17
113.77
77.48
77.16
76.84
S
f1 (ppm)

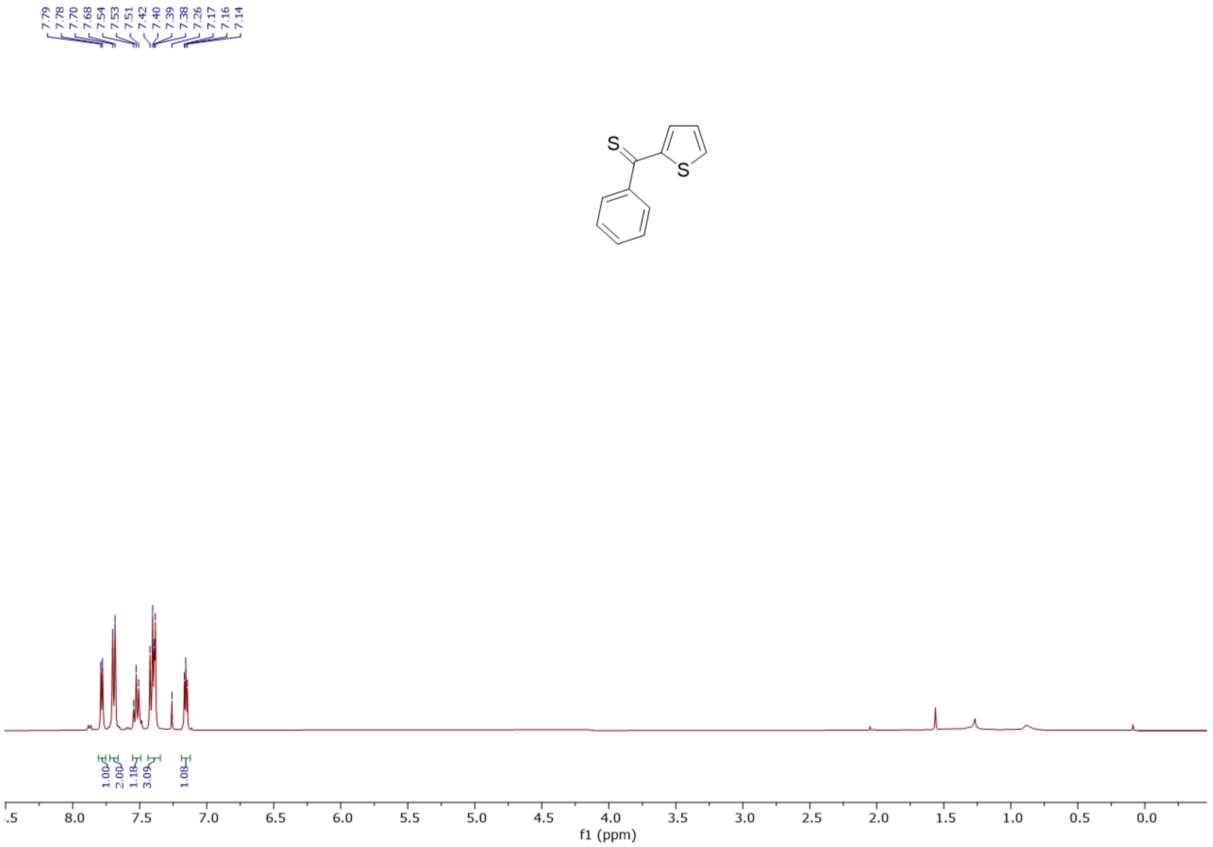
7.79
7.78
7.70
7.68
7.54
7.53
7.51
7.42
7.40
7.39
7.38
7.26
7.17
7.16
7.14
S
S
1.00
2.00
1.18
3.09
1.08
f1 (ppm)

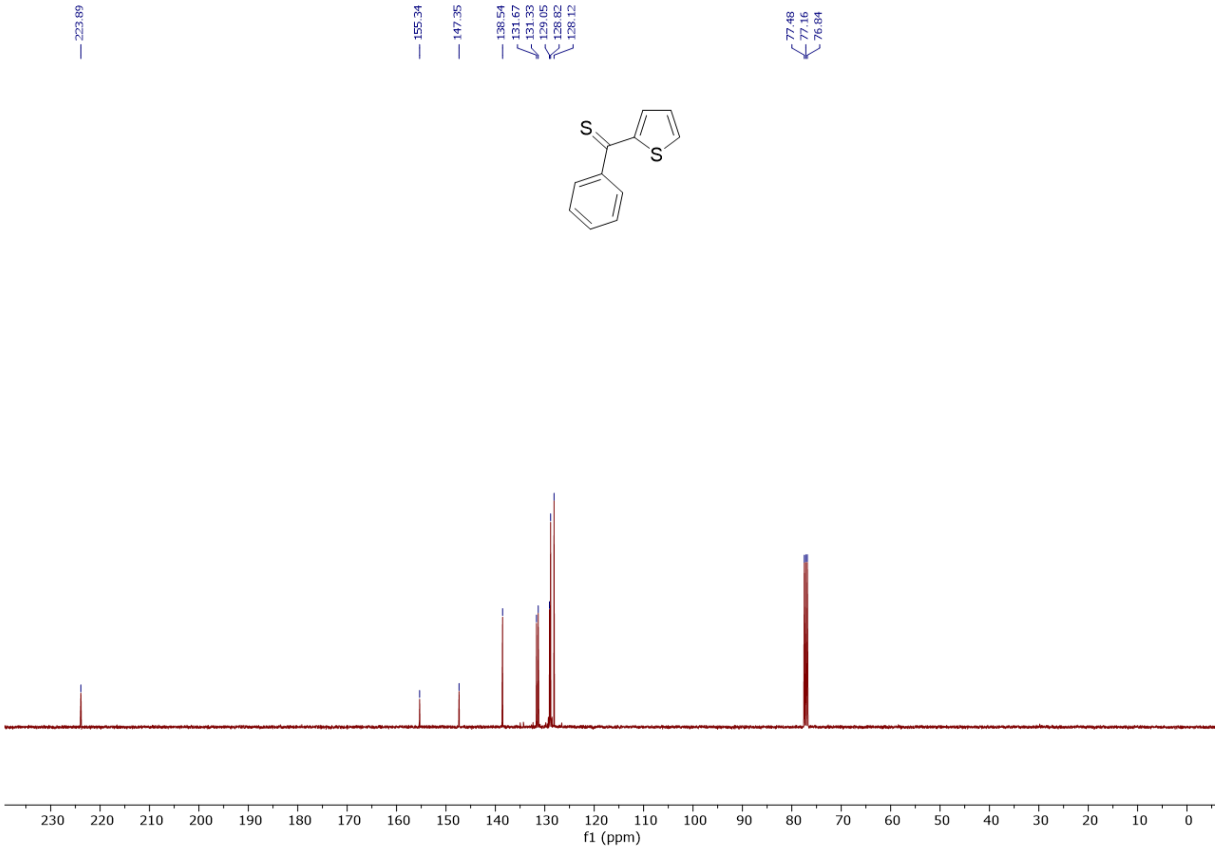
223.89
155.34
147.35
138.54
131.67
131.33
129.05
128.82
128.12
77.48
77.16
76.84
S
S
f1 (ppm)

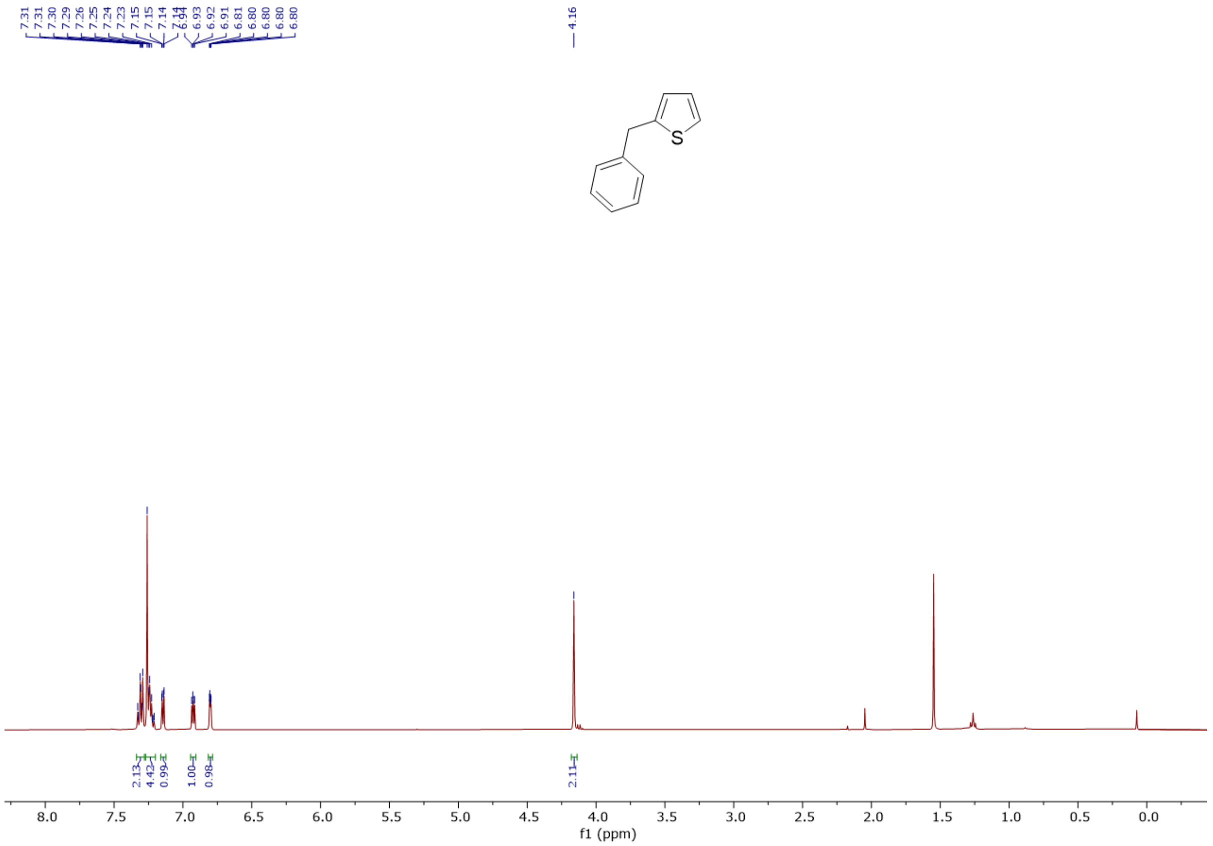
7.31
7.31
7.30
7.29
7.26
7.25
7.24
7.23
7.15
7.15
7.14
7.14
6.94
6.93
6.92
6.91
6.81
6.80
6.80
6.80
6.80
4.16
S
2.13
4.42
0.99
1.00
0.98
2.11
8.0
7.5
7.0
6.5
6.0
5.5
5.0
4.5
4.0
3.5
3.0
2.5
2.0
1.5
1.0
0.5
0.0
f1 (ppm)

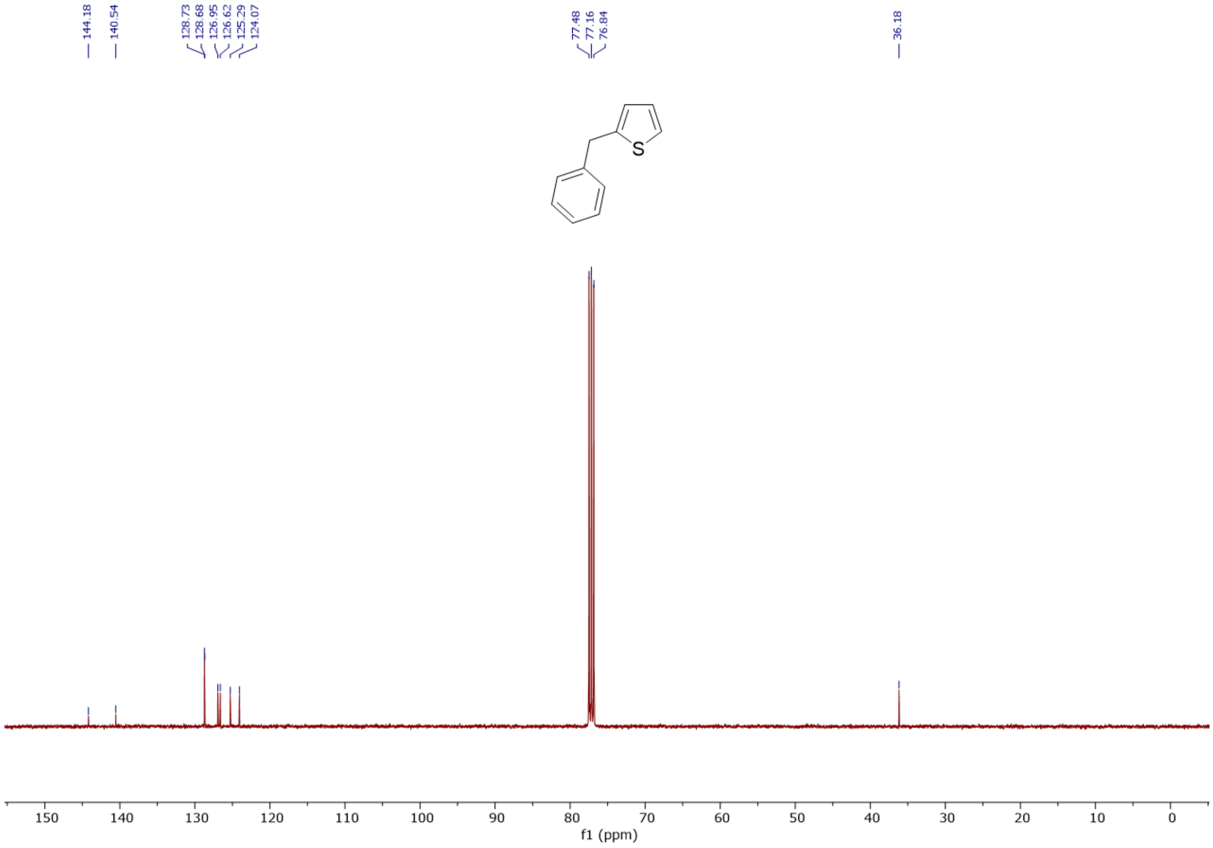
144.18
140.54
128.73
128.68
126.95
126.62
125.29
124.07
77.48
77.16
76.84
36.18
S
150
140
130
120
110
100
90
80
70
60
50
40
30
20
10
0
f1 (ppm)

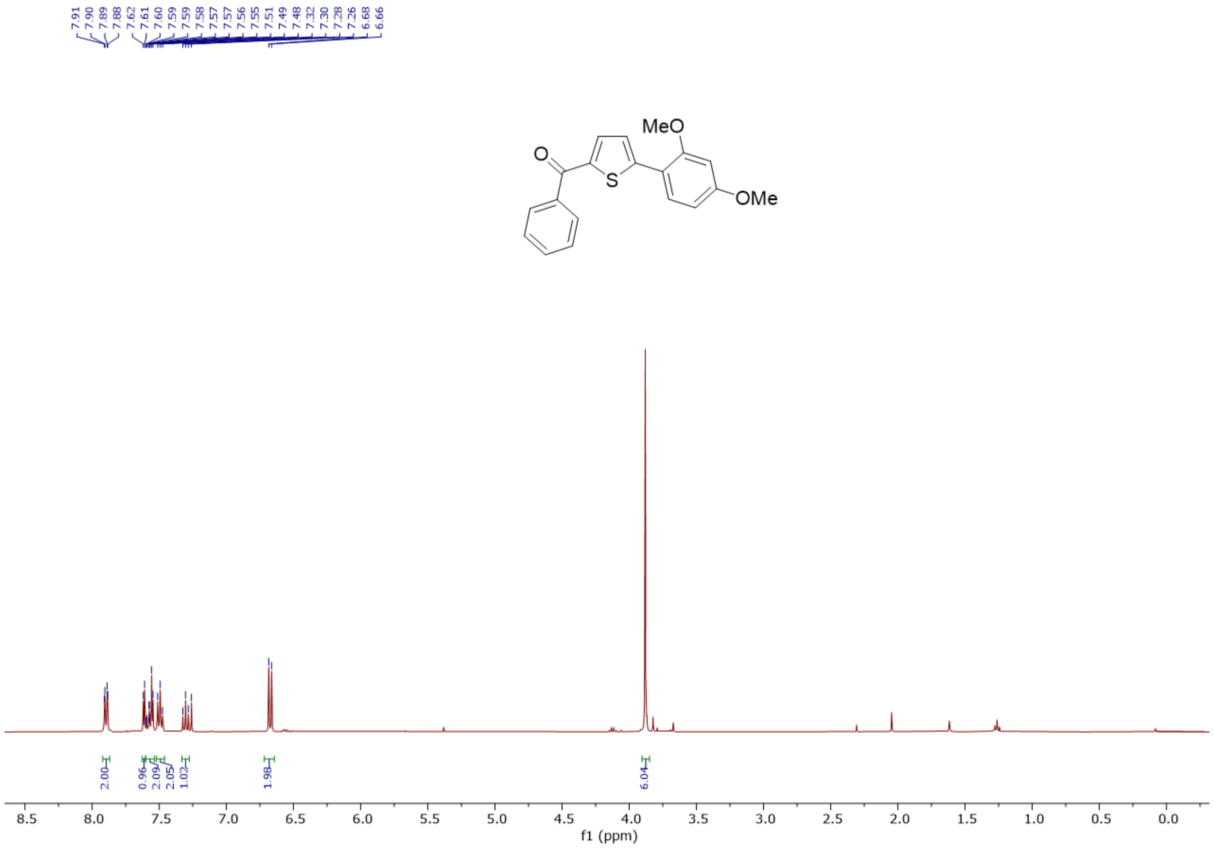
7.91
7.90
7.89
7.88
7.62
7.61
7.60
7.59
7.59
7.58
7.57
7.57
7.56
7.55
7.51
7.49
7.48
7.32
7.30
7.28
7.26
6.68
6.66
MeO
O
S
OMe
2.00
0.96
2.09
2.05
1.02
1.98
6.04
f1 (ppm)

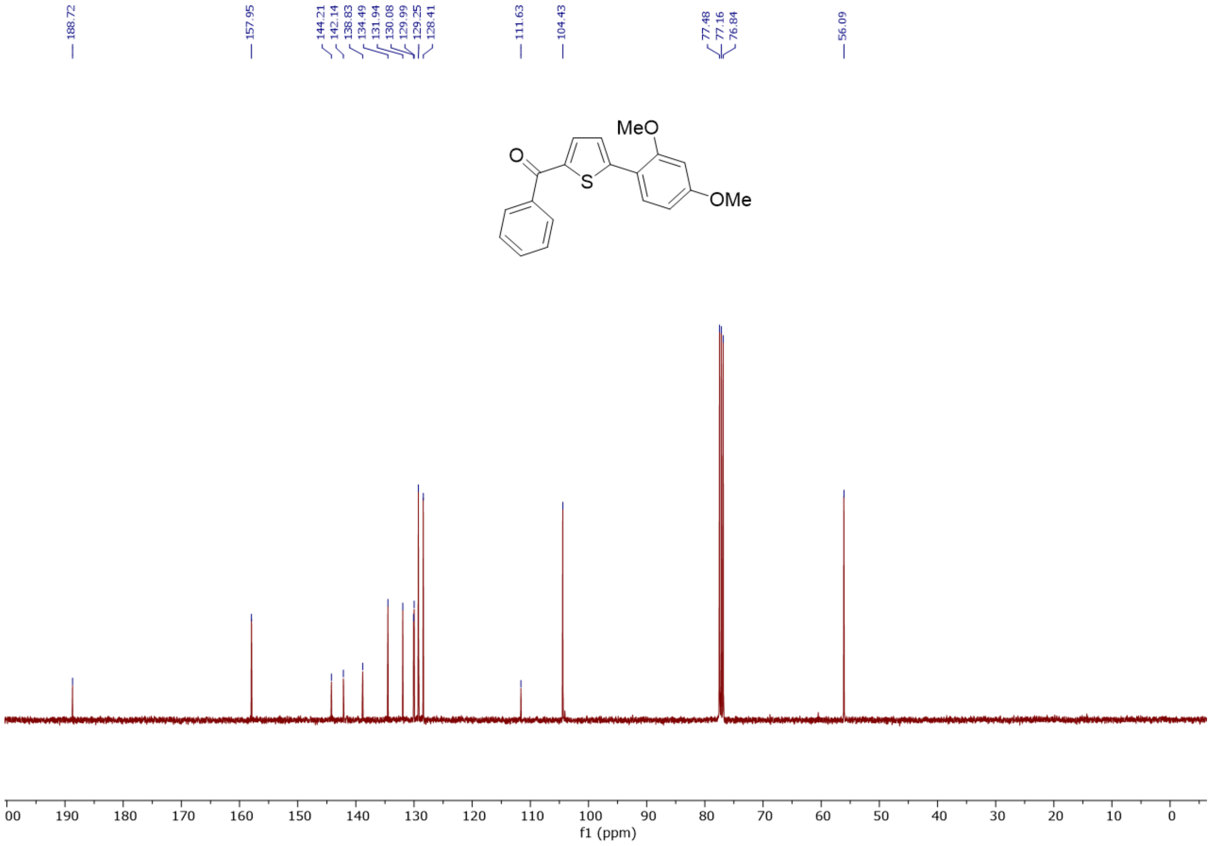
188.72
157.95
144.21
142.14
138.83
134.49
131.94
130.08
129.99
129.25
128.41
111.63
104.43
77.48
77.16
76.84
56.09
MeO
O
S
OMe
f1 (ppm)
